# Pediatric Intensive Care Unit Length of Stay Prediction by Machine Learning

**DOI:** 10.3390/bioengineering11100962

**Published:** 2024-09-26

**Authors:** Hammad A. Ganatra, Samir Q. Latifi, Orkun Baloglu

**Affiliations:** 1 Division of Pediatric Critical Care, Cleveland Clinic Children’s, Cleveland, OH 44195, USA; latifis@ccf.org (S.Q.L.); baloglo@ccf.org (O.B.); 2 Division of Cardiology and Cardiovascular Medicine, Cleveland Clinic Children’s, Cleveland, OH 44195, USA

**Keywords:** length of stay, machine learning, artificial intelligence, intensive care units, pediatric, deep learning

## Abstract

**Purpose**: To develop and validate machine learning models for predicting the length of stay (LOS) in the Pediatric Intensive Care Unit (PICU) using data from the Virtual Pediatric Systems (VPS) database. **Methods**: A retrospective study was conducted utilizing machine learning (ML) algorithms to analyze and predict PICU LOS based on historical patient data from the VPS database. The study included data from over 100 North American PICUs spanning the years 2015–2020. After excluding entries with missing variables and those indicating recovery from cardiac surgery, the dataset comprised 123,354 patient encounters. Various ML models, including Support Vector Machine, Stochastic Gradient Descent Classifier, K-Nearest Neighbors, Decision Tree, Gradient Boosting, CatBoost, and Recurrent Neural Networks (RNNs), were evaluated for their accuracy in predicting PICU LOS at thresholds of 24 h, 36 h, 48 h, 72 h, 5 days, and 7 days. **Results**: Gradient Boosting, CatBoost, and RNN models demonstrated the highest accuracy, particularly at the 36 h and 48 h thresholds, with accuracy rates between 70 and 73%. These results far outperform traditional statistical and existing prediction methods that report accuracy of only around 50%, which is effectively unusable in the practical setting. These models also exhibited balanced performance between sensitivity (up to 74%) and specificity (up to 82%) at these thresholds. **Conclusions**: ML models, particularly Gradient Boosting, CatBoost, and RNNs, show moderate effectiveness in predicting PICU LOS with accuracy slightly over 70%, outperforming previously reported human predictions. This suggests potential utility in enhancing resource and staffing management in PICUs. However, further improvements through training on specialized databases can potentially achieve better accuracy and clinical applicability.

## 1. Introduction

Machine learning (ML) and artificial intelligence (AI) are revolutionizing healthcare, with potential to transform patient care [1], enhance diagnostic accuracy [2], and optimize treatment pathways [3]. From assisting in interpreting radiological studies [4,5] to personalizing oncology therapy plans [2], these technologies offer significant potential across various healthcare fields. Analyzing electronic health records (EHRs) and large databases using ML and AI has also shown promise in improving patient outcomes and operational efficiency [6], particularly in critical care settings.

Pediatric intensive care units (PICUs) are resource-intensive environments within healthcare, necessitating specialized staffing and advanced medical equipment to cater to the complex needs of critically ill children. The nature of care in these units is compounded by the variability in patient ages, underlying conditions, and the critical interventions required. Consequently, PICUs face significant challenges in resource allocation, including the need for precise staffing levels and the availability of specialized equipment.

In this context, predicting the length of stay (LOS) in PICUs holds significant potential for improving resource allocation and staff scheduling. Accurate LOS predictions can lead to more efficient bed management, staffing decisions, and equipment planning, ultimately enhancing patient care and operational efficiency within the unit. Additionally, it can help better prepare patients and their families for the duration of their stay. Applying ML to predict LOS in PICUs presents a novel approach to these challenges. By utilizing the vast amounts of data generated in PICUs, ML models can identify patterns and predictors of LOS that might not be evident through traditional analytical methods.

To this end, the objective of this study is to use the Virtual Pediatric Systems (VPS, LLC http://www.myvps.org/ accessed on 23 September 2024) database, a comprehensive repository of pediatric critical care data, to develop and validate a machine learning model for predicting LOS in the PICU. The VPS database compiles clinical and administrative data on pediatric critical care admissions from over 100 North American PICUs and provides a readily available dataset for exploring the application of ML in this context.

## 2. Materials and Methods

### 2.1. Data Acquisition and Preprocessing

The study was reviewed by the Cleveland Clinic Institutional Review Board and approved as IRB-exempt research. It was not registered, and formal protocol was not prepared. We sourced our dataset by exporting the VPS database in CSV file format. The data extracted spans from the year 2015 to 2020, encompassing a broad temporal range to ensure development of a comprehensive ML model. We utilized Jupyter Notebooks version 7.2.2 as our integrated development environment (IDE), offering the ability to execute code blocks incrementally for data exploration. Additionally, its support for inline documentation and visualization makes it an ideal tool for collaborative data science projects, facilitating clear communication of methodologies and findings [7].

We employed Pandas version 2.2.3 [8] for preliminary data cleanup and analysis and removed patient records with essential missing variables. We consolidated certain variables (e.g., age groups, patient origin) to create more discrete and useful classes, such as recoding 28 categories of patient origin into 4: Emergency Department, Operating Room or Procedure Room, Lower Level of Care, and Another ICU. This processing made it easier to correlate patient demographics, initial care settings, and LOS in the PICU. Additionally, we simplified model development by focusing only on the primary organ system involved, streamlining the dataset for further processing and ensuring the quality and reliability of our predictive models. The above tasks were assisted by PandasAI version 2.2.15, a Python library that adds generative AI capabilities to Pandas, allowing analysis through natural language queries.

### 2.2. Data Transformation and Scaling

The LOS variable, originally continuous, was recoded into binary variables for different model thresholds. These binary variables represented whether LOS was less than or more than specific cutoffs: 24 h, 36 h, 48 h, 72 h, 96 h, 5 days, and 7 days, all clinically relevant thresholds for resource planning in PICUs. We utilized Scikit Learn’s (SKL version 1.6.0) OrdinalEncoder [9] to transform non-numeric categorical data into integers, ensuring that all features, regardless of their original format, were included in the predictive model. We preferred ordinal encoding for our categorical data since the main categories (e.g., LOS) have an inherent order that we wanted to preserve by converting them into integer values. This is important for models like gradient boosting and SVM, which benefit from maintaining the rank without assuming exact numeric distances. Unlike one-hot encoding, which loses ordinal information, and target encoding, which risks bias or leakage, OrdinalEncoder keeps the ordinal structure intact, aligning with model assumptions and capturing relationships more accurately. For other models such as KNN or RNN, ordinal encoding may not always provide a distinct advantage because these models may not inherently leverage the order of categories in the same way. However, it does not offer a disadvantage either.

**Figure 1:**
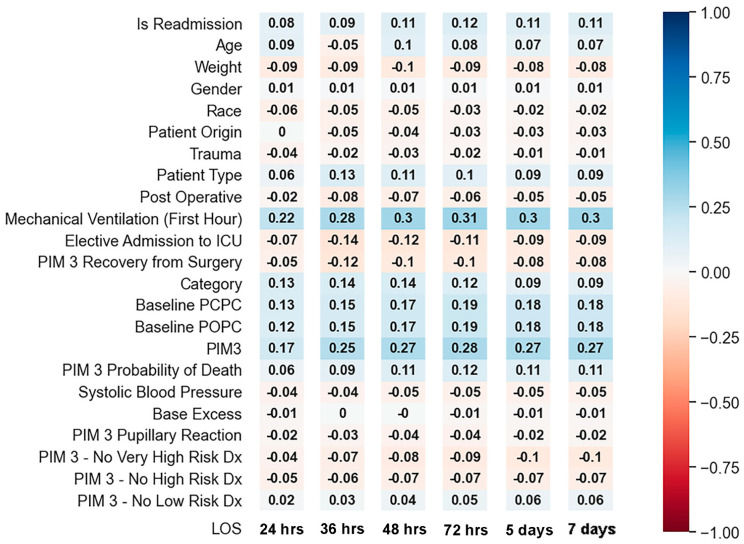
Input variables and their Pearson correlation heatmaps at each LOS threshold.

We created input and output vectors for the ML models, with the output vector representing binary recoded LOS and the input vector including patient demographics, physiological, and diagnostic variables (listed in Figure 1). Data normalization was performed using the MinMaxScaler from SKL [9], scaling values to 0–1, which is essential for effective ML model training and consistent performance.

### 2.3. Model Development and Evaluation

For each LOS cutoff iteration, we generated heatmaps using the Pearson coefficient to visualize variable correlations with LOS. These heatmaps provided a comprehensive, color-coded view of potential predictors, showing the complex and multifactorial nature of LOS determinants. This process is useful when initiating a ML project like ours, highlighting the limitations of using a single variable to predict LOS due to patient variability and the dynamic nature of critical care. The moderate to weak correlations observed for individual variables underscore the need for a multivariate approach in developing predictive ML models.

For each LOS cutoff, we trained and tested multiple well-established shallow learning algorithms: Support Vector Machine (SVM), Stochastic Gradient Descent Classifier (SGDC), K nearest neighbor (KNN), Decision Tree classifier, Gradient Boost, and Cat Boost. Each algorithm has specific strengths, and we wanted to explore which best served our healthcare dataset complexities. SVM is suited for high-dimensional spaces, robust against overfitting, and ideal for datasets rich in patient metrics. SGDC offers efficiency in linear classification and is beneficial for large-scale and sparse data that can be typical in healthcare datasets. KNN adapts well to new data, using a similarity vote among nearest neighbors, while Decision Tree Classifier provides easy-to-understand visualizations, crucial for explaining predictions in medical settings. Gradient Boosting effectively minimizes errors and improves accuracy by sequentially learning from past mistakes. Finally, Cat Boost is optimized for categorical variables that are common in healthcare and requires minimal data preprocessing.

We excluded other algorithms because they were not as well suited to the characteristics of our dataset or the goals of our analysis. For example, Naive Bayes assumes conditional independence between features, which is unrealistic in complex healthcare datasets where patient metrics are often correlated. Random Forest, while useful, tends to be less effective than Gradient Boosting or CatBoost in reducing bias and handling categorical variables in high-dimensional data. We also did not select Logistic Regression because it is too simplistic for multi-class healthcare data and does not capture non-linear relationships as effectively as decision trees or boosting methods. Lastly, AdaBoost, while similar to Gradient Boosting, can struggle with noisy data and is sensitive to outliers, making it less robust for our dataset. By choosing SVM, SGDC, KNN, Decision Trees, Gradient Boost, and CatBoost, we focused on models that offer a balance between performance, interpretability, and efficiency, making them better suited for addressing the complexities of healthcare data.

All shallow algorithms were imported from the SKL open access library [9]. The data were randomly split 80:20, with 80% for training and 20% for testing each algorithm’s LOS predictive performance. Additionally, we developed and tested a recurrent neural network (RNN) model using Keras version 3.5.0 [10] and Tensorflow 2.16.1 [11] framework developed by Google^TM^. For this model, the data were split 60:20:20 for training, validation, and testing, facilitating the tuning and evaluation of the RNN’s performance [10].

We evaluated these models using various metrics: accuracy, sensitivity (true positive rate), specificity (true negative rate), negative predictive value (NPV), positive predictive value (PPV, also known as “precision” in ML parlance), and the area under the receiver operating characteristic curve (AUC-ROC). The model’s accuracy was the outcome of primary interest, but it was put into perspective by the AUC-ROC, which weighs the true positive against the false positive rate. These metrics provided insights into the models’ classification accuracy, balance between true and false positives, and overall diagnostic ability in predicting LOS outcomes.

The Jupyter Notebook file with complete Python code and markdown is available as Supplementary Data in PDF (read-only) format.

## 3. Results

**Figure 2:**
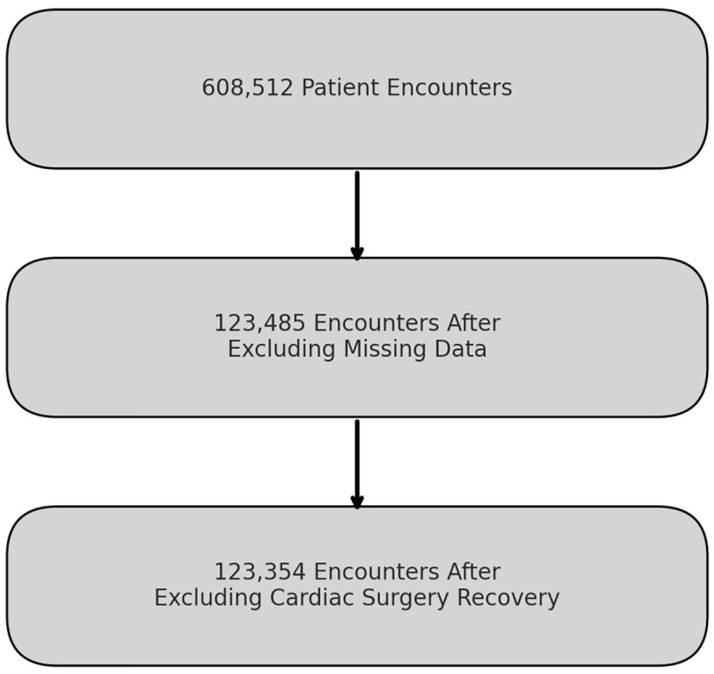
Flowchart depicting the sequential filtering process of patient encounters for inclusion in the machine learning models.

Our study analyzed data from 608,512 ICU patient encounters, treating repeat PICU admissions during the same hospitalization as separate encounters. After rigorous data processing, we retained 123,485 encounters, excluding those with missing variables like the Pediatric Overall Performance Category (POPC) and Pediatric Cerebral Performance Category (PCPC) scores. Further refinement removed encounters involving cardiac surgery recovery based on the Pediatric Index of Mortality 3 (PIM3) score, culminating in a final dataset comprising 123,354 encounters (Figure 2). Salient patient characteristics are detailed in Table 1.

**Figure 3:**
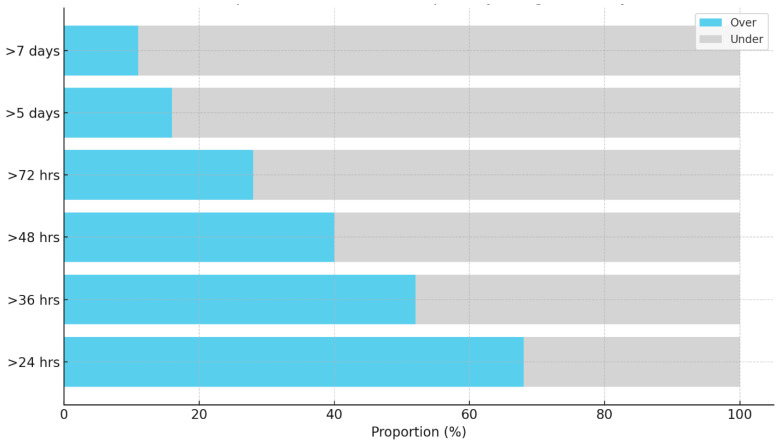
Proportion of patient samples by length of stay.

In evaluating the efficacy of various machine learning models to predict the LOS for PICU patients, this study systematically compared the performance across multiple LOS duration cutoffs: 24 h, 36 h, 48 h, 72 h, 5 days, and 7 days. The percentages of samples exceeding these durations were 68%, 52%, 40%, 28%, 16%, and 11%, respectively (Figure 3).

The Pearson coefficient heatmaps generated at each LOS cutoff are displayed in Figure 1. Correlations approaching +1 or −1 indicate strong positive or strong negative correlations; however, we found that no single input variable had a strong or even moderate correlation with LOS. Mechanical ventilation during the first hour and the PIM3 score were the only variables that showed weak correlation for predicting LOS at most time cutoffs (Pearson coefficient 0.2–0.3).

At the 24 h LOS threshold, Gradient boost and CatBoost demonstrated highest accuracy at 73%, followed by RNN with 72% and KNN with 70% accuracy (Table 2). Most models demonstrated high sensitivity in predicting LOS greater than 24 h (74% to 100%) but very low specificity (6–46%). Similarly, PPVs for LOS greater than 24 h were generally higher, reaching 75% for most models, but the NPVs remained low (45–62%). Finally, AUC-ROC for all ML models was persistently low (0.5–0.63) when attempting to predict LOS less than or greater than 24 h.

Running the models with a 36 h LOS cutoff yielded the best results. Gradient Boosting, CatBoost, and RNN showed robust performance, with accuracy rates of 70–72%, AUC-ROC of 0.70–0.72, and balanced sensitivity (71–74%) and specificity (69–70%), NPV (68–70%) and PPV (72–73%). At the 48 h cutoff, these models had similar accuracy (71–73%) and AUC-ROC (0.70–0.71), but higher specificity (79–82%) and NPV (75–76%), and lower sensitivity (59–62%) and PPV (65–68%).

When predicting longer LOS timeframes (72 h, 5 days, 7 days), all models demonstrated increasing accuracy, up to 89% (Supplemental Table S1). However, this was also associated with significantly increasing bias towards high specificity (84–99%) and high NPV (80–91%), and very low sensitivity (5–47%) and PPV (27–65%). Accordingly, the AUC-ROC scores were as low as 0.50.

## 4. Discussion

Our findings indicate that several ML models (particularly CatBoost, Gradient Boost, and RNNs) can achieve significant success in predicting PICU LOS, particularly for thresholds of 36 and 48 h (70–73% accuracy, with precision approaching 73%). While an accuracy of 73% may not seem very impressive at first glance, a review of the literature would suggest otherwise. Data show that ICU physicians are not successful at predicting LOS, approaching only 53% accuracy [12]. Similarly, emergency department physicians achieve 46% accuracy when predicting inpatient LOS [13], and intensivists and anesthesiologists successfully predicted ICU LOS after cardiac surgery in roughly 50% cases [14]. Given the perspective where human prediction is tantamount to a coin toss, we would argue that the 70–73% accuracy accomplished by our ML models is a significant improvement.

We believe our findings represent a successful initial exploration, and one of the first documented attempts to utilize AI/ML for LOS prediction in the PICU environment. The high costs of pediatric intensive care underscore the need for accurate predictions of staffing and resource allocation [15], allowing for cost-effective resource use and reduced wastage, which is essential in today’s financially constrained healthcare environment. Additionally, the COVID-19 pandemic has intensified staffing challenges for nurses and respiratory therapists [16,17,18], heightening the importance of reliable LOS predictions that allow healthcare administrators to better optimize staffing. Accurately forecasting LOS through ML models, therefore, addresses both economic and staffing challenges, enhancing patient care and resource management in PICUs.

Our ML models predict LOS more accurately at the 36 h and 48 h thresholds (vs. longer timeframes) due to a more balanced distribution of LOS at these points. As shown in Figure 3, our study sample has a near 50:50 split for LOS under or over 36 h and a 60:40 split for under or over 48 h. Other thresholds have skewed distributions, potentially resulting in ML models adopting a more computationally efficient strategy to “learn” the most common outcome. While this appears to increase accuracy, it tends to reduce precision (PPV) and sensitivity. For instance, predicting LOS greater/less than 7 days yields up to 89% accuracy but only 5% sensitivity and 27% PPV, rendering the prediction essentially unusable. This aligns with studies showing ML classifiers perform better with balanced datasets and tend to favor frequent outcomes in imbalanced data [19]. Such computational efficiency can be beneficial in certain contexts, but also highlights ML limitations in complex environments like the PICU.

Interestingly, in our iterative process we ended up using the PIM3-ROM and PIM3 score, as well as its constituent variables to formulate our best model. Although this may seem counterintuitive initially, it is recognized that inclusion of composite variables in a ML model can enhance its performance by integrating multiple dimensions, reducing noise, and encapsulating interaction effects [20,21]. Also, the PIM3 score was originally designed to predict risk of mortality and is calculated using a formula that assigns fixed coefficients to the component variables. It can be theorized that by including its constituent variables within the model, the model was able to learn and assign different coefficients and weightage to these variables to predict LOS.

A literature review reveals scarce data on using ML to predict LOS, but our results align with a recent Taiwanese investigation using “TabNet” to predict inpatient LOS from a single center’s emergency room admissions [22]. Despite their different patient population, their results were similar, with 71% accuracy and 78% precision. Another study using shallow ML models (logistic regression, random forest, and XGBoost) reported 66–77% accuracy for emergency department LOS predictions [23], paralleling our optimal ML model. The consistency across these studies and ours, highlights AI and ML’s potential in critical care predictions.

In our study, we explored both deep and shallow learning algorithms to predict the LOS. Shallow learning algorithms like decision trees, linear regression, and support vector machines, with limited layers, suit problems with less complex input–output relationships. Deep learning, with multiple layers, processes large data hierarchically, capturing complex patterns useful in tasks like image and speech recognition [24]. Interestingly, our best results were achieved equally by shallow (Gradient Boost and CatBoost) and deep learning (RNN) models. This suggests that shallow learning algorithms can perform effectively despite the potential of deep learning, emphasizing the need to choose the right approach based on the data and problem characteristics rather than defaulting to more complex models that may be computationally wasteful.

Our moderately effective ML models were trained on the VPS database, which is exceptionally large, encompassing over 600,000 unique encounters. This allowed us to liberally exclude patient encounters lacking complete information, while still retaining more than 120,000 patient encounters for model development and testing. Had we started with a smaller dataset, we would have been forced to handle missing data through less-than-ideal methods like imputation or limit ourselves to only using machine learning algorithms capable of dealing with incomplete data. Of note, in earlier iterations of this project (not published) we utilized statistical imputation to utilize all 600,000 patient encounters. However, this approach resulted in very low accuracy, and we therefore chose to forego imputation and used the best quality data for our modeling. While it may be disconcerting that we excluded almost 80% of the available VPS database, our approach is supported by Kariluoto et al., who have emphasized that the significance of ML models lies in the quality of data rather than the quantity [25].

We would like to emphasize that VPS was not originally designed for predicting LOS, and it has limitations when used in this setting. Although it is comprehensive, it includes optional data elements (e.g., disability scores) that some sites do not collect, or data that might be missing for logistical reasons (e.g., base excess in PIM3 scoring if no arterial or capillary blood gas was taken). Furthermore, clinicians looking to predict LOS might use variables that are not present in VPS. For example, while VPS includes systolic blood pressure (SBP) at admission, it lacks other early vital signs, creating a gap between available data and clinical insights needed for accurate LOS prediction. The first hours of admission offer crucial information affecting a patient’s trajectory, and the absence of this data likely limited our model’s prediction accuracy.

Moreover, it could be argued that certain variables within VPS are coded in ways that render them less suitable for machine learning modeling. For instance, VPS uses ICD codes or proprietary STAR codes for diagnoses. While these codes standardize diagnoses, they can lack the specificity needed for precise LOS predictions. For instance, septic shock in an immunocompromised oncologic patient differs significantly from that in an immunocompetent child, leading to different clinical courses and PICU LOS. Additionally, the VPS database contains 1533 unique STAR codes, and this large number of classes can weaken model predictive capability. To mitigate some of these challenges, we chose not to rely on specific ICD or STAR diagnoses, and instead categorized by the primary organ system involved (as documented in VPS), thereby reducing the classes from 1533 to just 25.

While traditional statistical methods have been used to identify factors that may be associated with PICU LOS or to produce benchmarks for PICU LOS, these methods are limited to population inferences and lack the ability to make personalized predictions [26]. Machine learning approaches such as ours are practical and implementable from an operational standpoint and can make predictions for an individual patient’s LOS by utilizing variables that are easily obtained within an hour of PICU admission. Our findings emphasize the need for databases tailored to support predictive analyses for LOS and similar outcomes. These databases should include a broader range of detailed clinical variables to enhance machine learning models’ accuracy, bolstering the case for AI and ML in clinical decisions, and we encourage the pediatric critical care medicine organizations such as SCCM and ESPNIC to facilitate such efforts. Despite challenges, our study shows the feasibility of using ML models to predict LOS in the PICU, which is crucial amid nursing and staffing shortages. By improving census prediction accuracy, our work suggests a path toward more efficient staffing and resource allocation in PICUs. Once adequately developed with more refined datasets, such models can be deployed within the electronic medical record to automatically extract data from a patient’s chart within the first few hours of admission. Automatic notifications can be sent to PICU managers about the predicted LOS for all patients in the PICU, and plans can then be updated to adjust nurse staffing and material resources such as ventilators.

## 5. Conclusions

Our study investigates machine learning models for predicting PICU LOS, marking an early effort in this area. CatBoost, Gradient Boost, and Recurrent Neural Networks showed moderate effectiveness, with accuracy slightly over 70%. Despite the limitations of the VPS database, our research highlights ML’s potential in healthcare and the need for specialized databases to improve model accuracy and utility. While not suitable as standalone AI agents yet, we believe future models trained with specialized datasets hold promise as tools for resource and staffing management in PICUs, addressing financial and staffing issues.

## Figures and Tables

**Table 1:** Demographic characteristics of sample utilized for ML modeling.

Characteristic	Values	Count	Percentage (%)
**Age**	0–2 years	39,018	31.63%
	Child 2 years to <6 years	23,923	19.40%
	Child 6 years to <12 years	22,927	18.59%
	12 years and up	37,486	30.38%
**Gender**	Male	67,785	54.96%
	Female	55,567	45.05%
	Ambiguous	2	0.002%
**Race**	White	57,002	46.22%
	Hispanic or Latino	25,688	20.83%
	Black or African American	21,539	17.46%
	Other/Mixed	7686	6.23%
	Unspecified	5569	4.51%
	Asian	2487	2.02%
	Asian/Indian/Pacific Islander	2367	1.92%
	American Indian or Alaska Native	669	0.54%
	Native Hawaiian or Other Pacific Islander	347	0.28%
**Patient Origin**	Emergency Department	69,441	56.30%
	OR/Procedure	28,013	22.71%
	Lower level of care	23,736	19.25%
	Another ICU	2164	1.75%
**Readmission**	No	86,939	70.48%
	Yes	36,415	29.52%
**Trauma**	No	111,927	90.75%
	Yes	11,427	9.25%
**Patient Type**	Unscheduled	100,194	81.22%
	Scheduled (>12 h in Advance)	23,160	18.78%
**Post Operative**	No	91,546	74.23%
	Yes	31,808	25.77%
**Mechanical Ventilation (First Hour)**	No	92,863	75.28%
	Yes	30,491	24.72%

**Table 2:** Summary of evaluation metrics for ML models at selected LOS cutoffs.

ML Model	Accuracy	Sensitivity	Specificity	NPV	PPV	AUC
	**24 h**					
**SVM**	68.60%	97%	6%	52%	69%	0.52
**SGDC**	68.40%	100%	0%	0%	68%	0.50
**KNN**	70.60%	86%	37%	55%	75%	0.62
**Decision Tree**	65%	74%	46%	45%	75%	0.60
**Gradient boost**	73%	90%	36%	62%	75%	0.63
**Cat boost**	73%	91%	32%	62%	74%	0.62
**RNN**	72%	90%	33%	60%	74%	0.61
	**36 h**					
**SVM**	66%	62%	70%	63%	69%	0.66
**SGDC**	65%	54%	77%	60%	72%	0.65
**KNN**	68%	71%	65%	67%	69%	0.68
**Decision Tree**	63%	65%	62%	61%	65%	0.63
**Gradient boost**	72%	74%	69%	71%	73%	0.72
**Cat boost**	71%	73%	69%	70%	72%	0.71
**RNN**	70%	71%	70%	68%	72%	0.70
	**48 h**					
**SVM**	68%	45%	85%	70%	66%	0.65
**SGDC**	68%	41%	86%	69%	66%	0.63
**KNN**	69%	56%	79%	73%	64%	0.67
**Decision Tree**	64%	55%	70%	70%	55%	0.63
**Gradient boost**	73%	62%	81%	76%	68%	0.71
**Cat boost**	73%	59%	82%	75%	68%	0.70
**RNN**	71%	61%	79%	75%	65%	0.70
	**72 h**					
**SVM**	75%	30%	93%	78%	60%	0.61
**SGDC**	74%	46%	84%	80%	53%	0.65
**KNN**	75%	37%	90%	79%	58%	0.63
**Decision Tree**	69%	47%	78%	79%	45%	0.63
**Gradient boost**	78%	42%	91%	81%	65%	0.67
**Cat boost**	77%	39%	92%	80%	65%	0.65
**RNN**	76%	43%	89%	80%	61%	0.66

SVM: Support Vector Machine, SGDC: Stochastic Gradient Descent Classifier, KNN: K-Nearest Neighbor, RNN: Recurrent Neural network, NPV: negative predictive value, PPV: positive predictive value, AUC: area under the curve.

## Data Availability

The database can be obtained through VPS, LLC. The machine learning source code is included in Supplementary Data.

## Supplementary Materials

The following supporting information can be downloaded at: https://www.mdpi.com/article/10.3390/bioengineering11100962/s1. The source code and extended results are included within the Supplementary Data file.
