# Supplementary material for: Pediatric Intensive Care Unit Length of Stay Prediction by Machine Learning
Source: Bioengineering (Basel). 2024 Sep 26;11(10):962. doi: 10.3390/bioengineering11100962 (PMC11504172; doi:10.3390/bioengineering11100962)
Supplement: Supplementary file 1 [file bioengineering-11-00962-s001.zip › bioengineering-3203847-supplementary.pdf]

Pediatric Intensive Care Unit Length of Stay Prediction by Machine Learning

Supplementary Data File

Table of Contents

- Supplemental Table 1. Summary of evaluation metrics for ML models at different LOS cutoffs - - - - - - - - - - - - - - - - - - - Page 3

- Jupyter Notebook with Python code for machine learning model execution - - - - - - - - - - - - - - - - - - - Page 4

Supplemental Table 1. Summary of evaluation metrics for ML models at different LOS cutoffs

ML Model	Accuracy	Sensitivity	Specificity	NPV	PPV	AUC	Accuracy	Sensitivity	Specificity	NPV	PPV	AUC
			**24 hrs**						**72 hrs**			
**SVM**	68.60%	97%	6%	52%	69%	0.52	75%	30%	93%	78%	60%	0.61
**SGDC**	68.40%	100%	0%	0%	68%	0.50	74%	46%	84%	80%	53%	0.65
**KNN**	70.60%	86%	37%	55%	75%	0.62	75%	37%	90%	79%	58%	0.63
**Decision Tree**	65%	74%	46%	45%	75%	0.60	69%	47%	78%	79%	45%	0.63
**Gradient boost**	73%	90%	36%	62%	75%	0.63	78%	42%	91%	81%	65%	0.67
**Cat boost**	73%	91%	32%	62%	74%	0.62	77%	39%	92%	80%	65%	0.65
**RNN**	72%	90%	33%	60%	74%	0.61	76%	43%	89%	80%	61%	0.66
			**36 hrs**						**5 days**			
**SVM**	66%	62%	70%	63%	69%	0.66	83%	5%	99%	84%	51%	0.52
**SGDC**	65%	54%	77%	60%	72%	0.65	83%	0%	100%	84%	0%	0.50
**KNN**	68%	71%	65%	67%	69%	0.68	83%	18%	96%	86%	49%	0.57
**Decision Tree**	63%	65%	62%	61%	65%	0.63	78%	37%	86%	87%	35%	0.62
**Gradient boost**	72%	74%	69%	71%	73%	0.72	85%	22%	97%	86%	59%	0.59
**Cat boost**	71%	73%	69%	70%	72%	0.71	85%	18%	98%	86%	61%	0.58
**RNN**	70%	71%	70%	68%	72%	0.70	84%	15%	98%	85%	57%	0.56
			**48 hrs**						**7 days**			
**SVM**	68%	45%	85%	70%	66%	0.65	88%	0%	100%	89%	47%	0.50
**SGDC**	68%	41%	86%	69%	66%	0.63	88%	0%	100%	89%	0%	0.50
**KNN**	69%	56%	79%	73%	64%	0.67	88%	0%	99%	89%	43%	0.53
**Decision Tree**	64%	55%	70%	70%	55%	0.63	83%	29%	90%	91%	27%	0.59
**Gradient boost**	73%	62%	81%	76%	68%	0.71	89%	9%	99%	89%	57%	0.54
**Cat boost**	73%	59%	82%	75%	68%	0.70	89%	6%	99%	89%	62%	0.53
**RNN**	71%	61%	79%	75%	65%	0.70	89%	5%	99%	89%	54%	0.52

SVM: Support Vector Machine, SGDC: Stochastic Gradient Descent Classifier, KNN: K-Nearest Neighbor, RNN: Recurrent Neural network, NPV: negative predictive value, PPV: positive predictive value, AUC: area under the curve

In [3]:
```
### IMPORT LIBRARIES ###

import pandas as pd
import numpy as np

from pandasai import SmartDataframe
from pandasai.llm.openai import OpenAI

from sklearn.preprocessing import MinMaxScaler, OrdinalEncoder
from sklearn.model_selection import train_test_split
from sklearn.metrics import (mean_absolute_percentage_error, mean_absolute_error,
                             accuracy_score, confusion_matrix, ConfusionMatrixDisplay,
                             RocCurveDisplay)

from tensorflow.keras import models, layers, optimizers

from matplotlib import pyplot as plt
import seaborn as sns

pd.set_option('display.max_columns', None)
#pd.set_option("display.max_rows", None)
```

In [8]:
```
## Import LLM from OpenAI for use with PandasAI ##
llm = OpenAI(api_token=[redacted]")
```

In [4]:
```
## Import the Diagnosis file and PIM file and merge dataframes ##

df5 = pd.read_csv('/Users/ganatrh/Downloads/DataWithPrimaryDiagnosis2.csv')
df70 = pd.read_csv('/Users/ganatrh/Downloads/PIM.csv')
```

In [5]:
```
df5 = df5.merge(df70, on='Case Index Id', how='left' )
```

In [6]:
```
df5.info()
```

```
<class 'pandas.core.frame.DataFrame'>
RangeIndex: 608512 entries, 0 to 608511
Data columns (total 78 columns):
 #   Column                                          Non-Null Count   Dtype  
---  ------                                          --------------   -----  
 0   Unit Id                                         608512 non-null  int64  
 1   Case Id                                         608512 non-null  int64  
 2   Case Index Id                                   608512 non-null  object 
 3   Discharge Year                                  608512 non-null  int64  
 4   Is Readmission                                  608512 non-null  int64  
 5   Age                                             608512 non-null  object 
 6   Weight (kg)                                     608512 non-null  float64
 7   Flag - Age/Weight                               608512 non-null  int64  
 8   Collects height                                 608512 non-null  int64  
 9   Height (cm)                                     309701 non-null  float64
 10  Gender                                          608512 non-null  object 
 11  Collects Race                                   608512 non-null  int64  
 12  Race                                            550714 non-null  object 
 13  Patient Origin                                  608512 non-null  object 
 14  Trauma                                          608512 non-null  int64  
 15  Patient Type                                    608512 non-null  object 
 16  Collects Transport Team                         608512 non-null  int64  
 17  Transport Team                                  371934 non-null  object 
 18  Collects Transport Vehicle                      608512 non-null  int64  
 19  Transport Vehicle                               347485 non-null  object 
 20  Post Operative                                  608512 non-null  int64  
 21  Collects Baseline PCPC/POPC                     608512 non-null  int64  
 22  Baseline PCPC                                   127543 non-null  object 
 23  Baseline POPC                                   127464 non-null  object 
 24  Collects FSS                                    608512 non-null  int64  
 25  Baseline FSS                                    59273 non-null   float64
 26  Cardiac Patient                                 608512 non-null  int64  
 27  Cardiac Procedure directly prior to or during stay  608512 non-null  int64  
 28  Medical Length of Stay (days)                   584327 non-null  float64
 29  Physical Length of Stay (days)                  608512 non-null  float64
 30  Hospital LOS                                    606176 non-null  float64
 31  Outcome                                         608512 non-null  object 
 32  Disposition                                     608512 non-null  object 
 33  Collects Limitation on Care                     608512 non-null  int64  
 34  Limitation on Care                              523209 non-null  float64
 35  Collects Brain Dead                             608512 non-null  int64  
 36  Is Brain Dead                                   4922 non-null    float64
 37  Collects Altered Code                           608512 non-null  int64  
 38  Is Altered Code                                 523227 non-null  float64
 39  Collects Withdrawal of Support                  608512 non-null  int64  
 40  Withdrawal of Support                           497357 non-null  float64
 41  Collects Autopsy                                608512 non-null  int64  
 42  Was Autopsy Performed                           11602 non-null   object 
 43  Collects Organ Donation                         608512 non-null  int64  
 44  Is Organ Donor                                  2572 non-null    float64
 45  Collects Tissue Donation                        608512 non-null  int64  
 46  Is Tissue Donor                                 3443 non-null    float64
 47  Collects Donation after Cardiac Death           608512 non-null  int64  
 48  Donation after Cardiac Death                    3563 non-null    float64
 49  Discharge PCPC                                  123029 non-null  object 
 50  Discharge POPC                                  122965 non-null  object 
 51  Discharge FSS                                   58085 non-null   float64
 52  Hospital Outcome                                604949 non-null  object 
 53  Collects Diagnosis STS Codes                    608512 non-null  int64  
 54  Collects Diagnosis ICD-9 Codes                  608512 non-null  int64  
```

```
 55  PIM 3 Score                                          608512 non-null  float64
 56  PIM 3 Probability of Death                           608512 non-null  float64
 57  Mechanical Ventilation (First Hour)                  608511 non-null  float64
 58  Elective Admission to ICU                            608511 non-null  float64
 59  PIM 3 Recovery from Surgery                          608511 non-null  object
 60  Category                                             608475 non-null  object
 61  Systolic Blood Pressure                              588367 non-null  float64
 62  Systolic Blood Pressure Unknown                      608511 non-null  float64
 63  PaO2 (mmHg)                                          13722 non-null   float64
 64  PaO2 (kPa)                                           13722 non-null   float64
 65  PaO2 Unknown                                         608511 non-null  float64
 66  FiO2                                                 13722 non-null   float64
 67  FiO2 Unknown                                         608511 non-null  float64
 68  Base Excess                                          51063 non-null   float64
 69  Base Excess Unknown                                  608511 non-null  float64
 70  PIM 3 Pupillary Reaction                             608511 non-null  object
 71  PIM 3 Pupillary Reaction Unknown                     608511 non-null  float64
 72  Admitted following Cardiac Bypass - PIM2/PIM3        608511 non-null  float64
 73  Admitted following Cardiac Bypass - PIM3 Baseline    608511 non-null  float64
 74  Admitted following Cardiac Bypass - PIM3 PostIndex   166 non-null     float64
 75  PIM 3 - No Very High Risk Dx                         608512 non-null  int64
 76  PIM 3 - No High Risk Dx                              608512 non-null  int64
 77  PIM 3 - No Low Risk Dx                               608512 non-null  int64
dtypes: float64(31), int64(28), object(19)
memory usage: 362.1+ MB
```

Remove rows that have flagged inaccurate weights

In [9]:

```python
df6 = SmartDataframe(df5, config={"llm": llm})
df7 = df6.chat("Remove rows that have the value '1' in column 'Flag - Age/Weight' and
df7.info()
```

```
<class 'pandas.core.frame.DataFrame'>
RangeIndex: 607901 entries, 0 to 607900
Data columns (total 78 columns):
 #   Column                                           Non-Null Count   Dtype  
---  ------                                           --------------   -----  
 0   Unit Id                                          607901 non-null  int64  
 1   Case Id                                          607901 non-null  int64  
 2   Case Index Id                                    607901 non-null  object 
 3   Discharge Year                                   607901 non-null  int64  
 4   Is Readmission                                   607901 non-null  int64  
 5   Age                                              607901 non-null  object 
 6   Weight (kg)                                      607901 non-null  float64
 7   Flag - Age/Weight                                607901 non-null  int64  
 8   Collects height                                  607901 non-null  int64  
 9   Height (cm)                                      309407 non-null  float64
 10  Gender                                           607901 non-null  object 
 11  Collects Race                                    607901 non-null  int64  
 12  Race                                             550155 non-null  object 
 13  Patient Origin                                   607901 non-null  object 
 14  Trauma                                           607901 non-null  int64  
 15  Patient Type                                     607901 non-null  object 
 16  Collects Transport Team                          607901 non-null  int64  
 17  Transport Team                                   371523 non-null  object 
 18  Collects Transport Vehicle                       607901 non-null  int64  
 19  Transport Vehicle                                347101 non-null  object 
 20  Post Operative                                   607901 non-null  int64  
 21  Collects Baseline PCPC/POPC                      607901 non-null  int64  
 22  Baseline PCPC                                    127387 non-null  object 
 23  Baseline POPC                                    127309 non-null  object 
 24  Collects FSS                                     607901 non-null  int64  
 25  Baseline FSS                                     59184 non-null   float64
 26  Cardiac Patient                                  607901 non-null  int64  
 27  Cardiac Procedure directly prior to or during stay  607901 non-null  int64  
 28  Medical Length of Stay (days)                    583736 non-null  float64
 29  Physical Length of Stay (days)                   607901 non-null  float64
 30  Hospital LOS                                     605567 non-null  float64
 31  Outcome                                          607901 non-null  object 
 32  Disposition                                      607901 non-null  object 
 33  Collects Limitation on Care                      607901 non-null  int64  
 34  Limitation on Care                               522675 non-null  float64
 35  Collects Brain Dead                              607901 non-null  int64  
 36  Is Brain Dead                                    4918 non-null    float64
 37  Collects Altered Code                            607901 non-null  int64  
 38  Is Altered Code                                  522693 non-null  float64
 39  Collects Withdrawal of Support                   607901 non-null  int64  
 40  Withdrawal of Support                            496859 non-null  float64
 41  Collects Autopsy                                 607901 non-null  int64  
 42  Was Autopsy Performed                            11595 non-null   object 
 43  Collects Organ Donation                          607901 non-null  int64  
 44  Is Organ Donor                                   2572 non-null    float64
 45  Collects Tissue Donation                         607901 non-null  int64  
 46  Is Tissue Donor                                  3442 non-null    float64
 47  Collects Donation after Cardiac Death            607901 non-null  int64  
 48  Donation after Cardiac Death                     3562 non-null    float64
 49  Discharge PCPC                                   122879 non-null  object 
 50  Discharge POPC                                   122815 non-null  object 
 51  Discharge FSS                                    58000 non-null   float64
 52  Hospital Outcome                                 604344 non-null  object 
 53  Collects Diagnosis STS Codes                     607901 non-null  int64  
 54  Collects Diagnosis ICD-9 Codes                   607901 non-null  int64  
```

```
 55  PIM 3 Score                                        607901 non-null  float64
 56  PIM 3 Probability of Death                         607901 non-null  float64
 57  Mechanical Ventilation (First Hour)                607900 non-null  float64
 58  Elective Admission to ICU                          607900 non-null  float64
 59  PIM 3 Recovery from Surgery                        607900 non-null  object
 60  Category                                           607864 non-null  object
 61  Systolic Blood Pressure                            587779 non-null  float64
 62  Systolic Blood Pressure Unknown                    607900 non-null  float64
 63  PaO2 (mmHg)                                        13708 non-null   float64
 64  PaO2 (kPa)                                         13708 non-null   float64
 65  PaO2 Unknown                                       607900 non-null  float64
 66  FiO2                                               13708 non-null   float64
 67  FiO2 Unknown                                       607900 non-null  float64
 68  Base Excess                                        50991 non-null   float64
 69  Base Excess Unknown                                607900 non-null  float64
 70  PIM 3 Pupillary Reaction                           607900 non-null  object
 71  PIM 3 Pupillary Reaction Unknown                   607900 non-null  float64
 72  Admitted following Cardiac Bypass - PIM2/PIM3      607900 non-null  float64
 73  Admitted following Cardiac Bypass - PIM3 Baseline  607900 non-null  float64
 74  Admitted following Cardiac Bypass - PIM3 PostIndex 166 non-null     float64
 75  PIM 3 - No Very High Risk Dx                       607901 non-null  int64
 76  PIM 3 - No High Risk Dx                            607901 non-null  int64
 77  PIM 3 - No Low Risk Dx                             607901 non-null  int64
dtypes: float64(31), int64(28), object(19)
memory usage: 361.8+ MB
```

Create dataframe with Columns of interest

In [11]:
```
df8 = SmartDataframe(df7, config={"llm": llm})
df9 = df8.chat("Select the columns 'Case Index Id', 'Is Readmission', 'Age', 'Weight (
```

In [12]:
```
df9.info()
```

```
<class 'pandas.core.frame.DataFrame'>
RangeIndex: 607901 entries, 0 to 607900
Data columns (total 41 columns):
 #   Column                                              Non-Null Count   Dtype  
---  ------                                              --------------   -----  
 0   Case Index Id                                       607901 non-null  object 
 1   Is Readmission                                      607901 non-null  int64  
 2   Age                                                 607901 non-null  object 
 3   Weight (kg)                                         607901 non-null  float64
 4   Gender                                              607901 non-null  object 
 5   Race                                                550155 non-null  object 
 6   Patient Origin                                      607901 non-null  object 
 7   Trauma                                              607901 non-null  int64  
 8   Patient Type                                        607901 non-null  object 
 9   Transport Team                                      371523 non-null  object 
 10  Transport Vehicle                                   347101 non-null  object 
 11  Post Operative                                      607901 non-null  int64  
 12  Baseline PCPC                                       127387 non-null  object 
 13  Baseline POPC                                       127309 non-null  object 
 14  Baseline FSS                                        59184 non-null   float64
 15  Cardiac Patient                                     607901 non-null  int64  
 16  Cardiac Procedure directly prior to or during stay  607901 non-null  int64  
 17  Medical Length of Stay (days)                       583736 non-null  float64
 18  Physical Length of Stay (days)                      607901 non-null  float64
 19  Hospital LOS                                        605567 non-null  float64
 20  Outcome                                             607901 non-null  object 
 21  Disposition                                         607901 non-null  object 
 22  Is Altered Code                                     522693 non-null  float64
 23  Discharge PCPC                                      122879 non-null  object 
 24  Discharge POPC                                      122815 non-null  object 
 25  Discharge FSS                                       58000 non-null   float64
 26  Hospital Outcome                                    604344 non-null  object 
 27  PIM 3 Score                                         607901 non-null  float64
 28  PIM 3 Probability of Death                          607901 non-null  float64
 29  Mechanical Ventilation (First Hour)                 607900 non-null  float64
 30  Elective Admission to ICU                           607900 non-null  float64
 31  PIM 3 Recovery from Surgery                         607900 non-null  object 
 32  Systolic Blood Pressure                             587779 non-null  float64
 33  PaO2 (mmHg)                                         13708 non-null   float64
 34  FiO2                                                13708 non-null   float64
 35  Base Excess                                         50991 non-null   float64
 36  PIM 3 Pupillary Reaction                            607900 non-null  object 
 37  PIM 3 - No Very High Risk Dx                        607901 non-null  int64  
 38  PIM 3 - No High Risk Dx                             607901 non-null  int64  
 39  PIM 3 - No Low Risk Dx                              607901 non-null  int64  
 40  Category                                            607864 non-null  object 
dtypes: float64(15), int64(8), object(18)
memory usage: 190.2+ MB
```

Remove rows that have NaN for "elective ICU admission" and for "POPC/PCPC". This comcomitantly removes NaN for other variables too

In [14]:

```
df9.dropna(subset=['Elective Admission to ICU', 'Baseline POPC', 'Baseline PCPC', 'Sys
df9.info()
```

```
<class 'pandas.core.frame.DataFrame'>
RangeIndex: 123485 entries, 0 to 123484
Data columns (total 41 columns):
 #   Column                                               Non-Null Count   Dtype  
---  ------                                               --------------   -----  
 0   Case Index Id                                        123485 non-null  object 
 1   Is Readmission                                       123485 non-null  int64  
 2   Age                                                  123485 non-null  object 
 3   Weight (kg)                                          123485 non-null  float64
 4   Gender                                               123485 non-null  object 
 5   Race                                                 121785 non-null  object 
 6   Patient Origin                                       123485 non-null  object 
 7   Trauma                                               123485 non-null  int64  
 8   Patient Type                                         123485 non-null  object 
 9   Transport Team                                       98498 non-null   object 
 10  Transport Vehicle                                    104581 non-null  object 
 11  Post Operative                                       123485 non-null  int64  
 12  Baseline PCPC                                        123485 non-null  object 
 13  Baseline POPC                                        123485 non-null  object 
 14  Baseline FSS                                         16690 non-null   float64
 15  Cardiac Patient                                      123485 non-null  int64  
 16  Cardiac Procedure directly prior to or during stay  123485 non-null  int64  
 17  Medical Length of Stay (days)                        118321 non-null  float64
 18  Physical Length of Stay (days)                       123485 non-null  float64
 19  Hospital LOS                                         123245 non-null  float64
 20  Outcome                                              123485 non-null  object 
 21  Disposition                                          123485 non-null  object 
 22  Is Altered Code                                      122991 non-null  float64
 23  Discharge PCPC                                       118721 non-null  object 
 24  Discharge POPC                                       118695 non-null  object 
 25  Discharge FSS                                        16383 non-null   float64
 26  Hospital Outcome                                     122734 non-null  object 
 27  PIM 3 Score                                          123485 non-null  float64
 28  PIM 3 Probability of Death                           123485 non-null  float64
 29  Mechanical Ventilation (First Hour)                  123485 non-null  float64
 30  Elective Admission to ICU                            123485 non-null  float64
 31  PIM 3 Recovery from Surgery                          123485 non-null  object 
 32  Systolic Blood Pressure                              123485 non-null  float64
 33  PaO2 (mmHg)                                          3205 non-null    float64
 34  FiO2                                                 3205 non-null    float64
 35  Base Excess                                          12155 non-null   float64
 36  PIM 3 Pupillary Reaction                             123485 non-null  object 
 37  PIM 3 - No Very High Risk Dx                         123485 non-null  int64  
 38  PIM 3 - No High Risk Dx                              123485 non-null  int64  
 39  PIM 3 - No Low Risk Dx                               123485 non-null  int64  
 40  Category                                             123485 non-null  object 
dtypes: float64(15), int64(8), object(18)
memory usage: 38.6+ MB
```

In [15]:
```
df9["Base Excess"] = df9["Base Excess"].replace(np.nan, 0)
df9["Base Excess"].value_counts()
```

Out[15]:
```
Base Excess
 0.0     111514
-3.0        358
-4.0        342
-5.0        329
-2.0        327
          ...  
-16.5         1
 9.7          1
 23.3         1
-34.1         1
 11.3         1
Name: count, Length: 472, dtype: int64
```

The cells below consolidate all patients > 18 into one group

In [16]:
```
df9["Age"].value_counts()
```

Out[16]:
```
Age
Infant 29 days to < 2 years                    36705
Adolescent 12 years to < 18 years              32266
Child 2 years to < 6 years                     23946
Child 6 years to < 12 years                    22954
Adolescent (late) 18 years to < 21 years        4241
Neonate Birth to 29 days                        2355
Adult 21 years and up                           1018
Name: count, dtype: int64
```

In [17]:
```
df10 = df9.copy()
df10.Age.replace(['Adult 21 years and up','Adolescent (late) 18 years to < 21 years'],
```

In [18]:
```
df10["Age"].value_counts()
```

Out[18]:
```
Age
Infant 29 days to < 2 years          36705
Adolescent 12 years to < 18 years    32266
Child 2 years to < 6 years           23946
Child 6 years to < 12 years          22954
Adult 18 years and up                 5259
Neonate Birth to 29 days              2355
Name: count, dtype: int64
```

The cells below consolidate "Patient origin" into more discrete categories

In [19]:
```
df10["Patient Origin"].value_counts()
```

Out[19]:
```
Patient Origin
Emergency Department                                                      47563
Another Hospital's Emergency Department                                   21884
General Care Floor                                                        18033
Operating Room (Direct to ICU)                                            17792
Recovery Room (PACU)                                                       9248
Another Hospital's ICU                                                     1796
Another Hospital's General Care Floor                                      1753
Step-Down Unit/Intermediate Care Unit                                      1374
Home                                                                       1120
Physician's Office/Clinic                                                   959
Inpatient Procedure Suite (not cath lab)                                    558
Outpatient Procedure Suite                                                  255
NICU (in this hospital)                                                     229
Another Hospital's OR                                                       205
Other                                                                       151
Another ICU in this hospital (except NICU)                                  129
Transitional Care/Skilled Nursing/Chronic Care Facility                     122
Dedicated technology dependent unit (transitional/progressive care unit)    104
Physical Rehab Center                                                        64
Cath lab                                                                     57
Another hospital's cath lab                                                  17
Another hospital's Step-Down Unit/Intermediate Care Unit                     16
Psychiatric/Substance Abuse/Chemical Dependence Rehab Center                 13
Another Hospital's dedicated home ventilator unit                            13
Delivery Room (including delivery room in another hospital)                  12
Telemetry Unit                                                               10
Pulmonary Rehab Center                                                        7
Another hospital's Telemetry Unit                                             1
Name: count, dtype: int64
```

In [20]:
```
df11 = df10.copy()
df11["Patient Origin"].replace(["Another Hospital's General Care Floor", "Transitional
df11["Patient Origin"].replace(["Another Hospital's ICU", "Another Hospital's OR", "Ar
df11["Patient Origin"].replace(["Home", "Physician's Office/Clinic"], "Home/Clinic",
df11["Patient Origin"].replace(["Inpatient Procedure Suite (not cath lab)", "Outpatier
df11["Patient Origin"].replace(["Step-Down Unit/Intermediate Care Unit", "Telemetry Ur
```

In [21]:
```
df11["Patient Origin"].value_counts()
```

Out[21]:
```
Patient Origin
Emergency Department                                           47563
Another Hospital's Emergency Department                        21884
General Care Floor                                             18033
Operating Room (Direct to ICU)                                 17792
Recovery Room (PACU)                                            9248
Home/Clinic                                                     2079
Another Hospital ICU/OR/Cath                                    2018
Another Hospital Non-ED Non-ICU unit                            1989
Step down/Intermediate/Telemetry                                1488
Procedure Suite                                                  813
NICU (in this hospital)                                          229
Other                                                            151
Another ICU in this hospital (except NICU)                       129
Cath lab                                                          57
Delivery Room (including delivery room in another hospital)       12
Name: count, dtype: int64
```

Remove rows with PIM3 recovery from surgery showing a recovery from cardiac surgery

In [22]:
```
df11["PIM 3 Recovery from Surgery"].value_counts()
```

Out[22]:
```
PIM 3 Recovery from Surgery
No                                               99362
Yes, Recovery from non-cardiac procedure         23992
Yes, Recovery from non-bypass cardiac procedure     98
Yes, Recovery from bypass cardiac procedure         33
Name: count, dtype: int64
```

In [23]:
```
df12 = df11.copy()
df12 = df12.drop(df12[df12['PIM 3 Recovery from Surgery'] == 'Yes, Recovery from non-b
df12 = df12.drop(df12[df12['PIM 3 Recovery from Surgery'] == 'Yes, Recovery from bypas
df12 = df12.reset_index(drop=True)
```

In [24]:
```
df12["PIM 3 Recovery from Surgery"].value_counts()
```

Out[24]:
```
PIM 3 Recovery from Surgery
No                                          99362
Yes, Recovery from non-cardiac procedure    23992
Name: count, dtype: int64
```

Fill Race NaN with "Unspecified"

In [25]:
```
df12.Race.fillna('Unspecified', inplace=True)
df12.Race.unique()
```

Out[25]:
```
array(['White', 'Other/Mixed', 'Hispanic or Latino',
       'Black or African American', 'Asian/Indian/Pacific Islander',
       'Unspecified', 'Asian', 'American Indian or Alaska Native',
       'Native Hawaiian or Other Pacific Islander'], dtype=object)
```

Consolidate Pupillary reactions to Fixed>3mm and others/unknown

In [26]:
```
df12["PIM 3 Pupillary Reaction"].value_counts()
```

Out[26]:
```
PIM 3 Pupillary Reaction
Other                                   116524
Unknown                                   5040
>3mm and both fixed                        927
Pupilary Assessment Invalid - Drugs        651
Pupilary Assessment Invalid - Injury       141
Pupilary Assessment Invalid - Toxins        71
Name: count, dtype: int64
```

In [27]:
```
df13 = df12.copy()
df13["PIM 3 Pupillary Reaction"].replace(["Other", "Unknown", "Pupilary Assessment Inv
df13["PIM 3 Pupillary Reaction"].value_counts()
```

Out[27]:
```
PIM 3 Pupillary Reaction
Other/Unknown          122427
>3mm and both fixed       927
Name: count, dtype: int64
```

Divide data into LOS - 24hrs

In [28]:
```python
chatBot = SmartDataframe(df13, config={"llm": llm})
```

In [29]:
```python
chatBot.chat("For the column 'Physical Length of Stay (days)', what percentage of rows
```

Out[29]:
```
68.62363603936637
```

In [30]:
```python
df14 = chatBot.chat("Create a new column 'LOS', and if the value in column 'Physical L
df14.LOS.value_counts()
```

Out[30]:
```
LOS
1    84650
0    38704
Name: count, dtype: int64
```

In [31]:
```python
df14.info()
```

```
<class 'pandas.core.frame.DataFrame'>
RangeIndex: 123354 entries, 0 to 123353
Data columns (total 42 columns):
 #   Column                                              Non-Null Count   Dtype  
---  ------                                              --------------   -----  
 0   Case Index Id                                       123354 non-null  object 
 1   Is Readmission                                      123354 non-null  int64  
 2   Age                                                 123354 non-null  object 
 3   Weight (kg)                                         123354 non-null  float64
 4   Gender                                              123354 non-null  object 
 5   Race                                                123354 non-null  object 
 6   Patient Origin                                      123354 non-null  object 
 7   Trauma                                              123354 non-null  int64  
 8   Patient Type                                        123354 non-null  object 
 9   Transport Team                                      98391 non-null   object 
 10  Transport Vehicle                                   104468 non-null  object 
 11  Post Operative                                      123354 non-null  int64  
 12  Baseline PCPC                                       123354 non-null  object 
 13  Baseline POPC                                       123354 non-null  object 
 14  Baseline FSS                                        16666 non-null   float64
 15  Cardiac Patient                                     123354 non-null  int64  
 16  Cardiac Procedure directly prior to or during stay  123354 non-null  int64  
 17  Medical Length of Stay (days)                       118191 non-null  float64
 18  Physical Length of Stay (days)                      123354 non-null  float64
 19  Hospital LOS                                        123115 non-null  float64
 20  Outcome                                             123354 non-null  object 
 21  Disposition                                         123354 non-null  object 
 22  Is Altered Code                                     122862 non-null  float64
 23  Discharge PCPC                                      118592 non-null  object 
 24  Discharge POPC                                      118566 non-null  object 
 25  Discharge FSS                                       16359 non-null   float64
 26  Hospital Outcome                                    122609 non-null  object 
 27  PIM 3 Score                                         123354 non-null  float64
 28  PIM 3 Probability of Death                          123354 non-null  float64
 29  Mechanical Ventilation (First Hour)                 123354 non-null  float64
 30  Elective Admission to ICU                           123354 non-null  float64
 31  PIM 3 Recovery from Surgery                         123354 non-null  object 
 32  Systolic Blood Pressure                             123354 non-null  float64
 33  PaO2 (mmHg)                                         3179 non-null    float64
 34  FiO2                                                3179 non-null    float64
 35  Base Excess                                         123354 non-null  float64
 36  PIM 3 Pupillary Reaction                            123354 non-null  object 
 37  PIM 3 - No Very High Risk Dx                        123354 non-null  int64  
 38  PIM 3 - No High Risk Dx                             123354 non-null  int64  
 39  PIM 3 - No Low Risk Dx                              123354 non-null  int64  
 40  Category                                            123354 non-null  object 
 41  LOS                                                 123354 non-null  int64  
dtypes: float64(15), int64(9), object(18)
memory usage: 39.5+ MB
```

Apply Ordinal Encoder to convert Object datatype to Integers

In [33]:
```
toEncode = df14[['Is Readmission', 'Age', 'Gender', 'Race', 'Patient Origin', 'Trauma'
                'Post Operative', 'Mechanical Ventilation (First Hour)', 'Elective Adm
                'PIM 3 Recovery from Surgery', 'Category', 'Baseline PCPC', 'Baseline 
                 'PIM 3 Pupillary Reaction']].copy()
columnNames = list(toEncode.columns)

enc = OrdinalEncoder()
```

```
df15 = pd.DataFrame(enc.fit_transform(toEncode), columns= columnNames)

for i in range(len(columnNames)):
    print (columnNames[i] , enc.categories_[i])
```

```
Is Readmission [0 1]
Age ['Adolescent 12 years to < 18 years' 'Adult 18 years and up'
 'Child 2 years to < 6 years' 'Child 6 years to < 12 years'
 'Infant 29 days to < 2 years' 'Neonate Birth to 29 days']
Gender ['Ambiguous' 'Female' 'Male']
Race ['American Indian or Alaska Native' 'Asian'
 'Asian/Indian/Pacific Islander' 'Black or African American'
 'Hispanic or Latino' 'Native Hawaiian or Other Pacific Islander'
 'Other/Mixed' 'Unspecified' 'White']
Patient Origin ['Another Hospital ICU/OR/Cath' 'Another Hospital Non-ED Non-ICU unit'
 "Another Hospital's Emergency Department"
 'Another ICU in this hospital (except NICU)' 'Cath lab'
 'Delivery Room (including delivery room in another hospital)'
 'Emergency Department' 'General Care Floor' 'Home/Clinic'
 'NICU (in this hospital)' 'Operating Room (Direct to ICU)' 'Other'
 'Procedure Suite' 'Recovery Room (PACU)'
 'Step down/Intermediate/Telemetry']
Trauma [0 1]
Patient Type ['Scheduled (> or = 12 Hours in Advance)' 'Unscheduled']
Post Operative [0 1]
Mechanical Ventilation (First Hour) [0. 1.]
Elective Admission to ICU [0. 1.]
PIM 3 Recovery from Surgery ['No' 'Yes, Recovery from non-cardiac procedure']
Category ['Cardiovascular' 'Dermatologic' 'Endocrine' 'Factors Influencing Health'
 'Gastrointestinal' 'Genetic' 'Gynecologic' 'Hematologic' 'Immunologic'
 'Infectious' 'Injury/Poisoning/Adverse Effects' 'Metabolic' 'Neurologic'
 'Newborn/Perinatal' 'Oncologic' 'Ophthalmologic' 'Orthopedic'
 'Psychiatric' 'Renal/Genitourinary' 'Respiratory' 'Respiratory/ENT'
 'Rheumatologic' 'Symptoms' 'Transplant' 'Ungroupable']
Baseline PCPC ['1 - Normal' '2 - Mild disability' '3 - Moderate disability'
 '4 - Severe disability' '5 - Coma or vegetative state']
Baseline POPC ['1 - Good overall performance' '2 - Mild overall disability'
 '3 - Moderate overall disability' '4 - Severe overall disability'
 '5 - Coma or vegetative state']
PIM 3 Pupillary Reaction ['>3mm and both fixed' 'Other/Unknown']
```

In [34]:
```
df15.info()
```

```
<class 'pandas.core.frame.DataFrame'>
RangeIndex: 123354 entries, 0 to 123353
Data columns (total 15 columns):
 #   Column                                Non-Null Count   Dtype  
---  ------                                --------------   -----  
 0   Is Readmission                        123354 non-null  float64
 1   Age                                   123354 non-null  float64
 2   Gender                                123354 non-null  float64
 3   Race                                  123354 non-null  float64
 4   Patient Origin                        123354 non-null  float64
 5   Trauma                                123354 non-null  float64
 6   Patient Type                          123354 non-null  float64
 7   Post Operative                        123354 non-null  float64
 8   Mechanical Ventilation (First Hour)  123354 non-null  float64
 9   Elective Admission to ICU             123354 non-null  float64
 10  PIM 3 Recovery from Surgery           123354 non-null  float64
 11  Category                              123354 non-null  float64
 12  Baseline PCPC                         123354 non-null  float64
 13  Baseline POPC                         123354 non-null  float64
 14  PIM 3 Pupillary Reaction              123354 non-null  float64
dtypes: float64(15)
memory usage: 14.1 MB
```

Add "weight" and "PIM3" Score into the dataframe

In [35]:

```
df16 = df15.copy()

df16.insert(2, "Weight", 0)
df16.insert(15, "PIM3", 0)
df16.insert(16, "PIM 3 Probability of Death", 0)
df16.insert(17, "Systolic Blood Pressure", 0)
df16.insert(18, "Base Excess", 0)
df16.insert(20, "PIM 3 - No Very High Risk Dx", 0)
df16.insert(21, "PIM 3 - No High Risk Dx", 0)
df16.insert(22, "PIM 3 - No Low Risk Dx", 0)




df16.Weight = df14["Weight (kg)"].copy()
df16.PIM3 = df14["PIM 3 Score"].copy()
df16["PIM 3 Probability of Death"] = df14["PIM 3 Probability of Death"].copy()
df16["Systolic Blood Pressure"] = df14["Systolic Blood Pressure"].copy()
df16["Base Excess"] = df14["Base Excess"].copy()
df16["PIM 3 - No Very High Risk Dx"] = df14["PIM 3 - No Very High Risk Dx"].copy()
df16["PIM 3 - No High Risk Dx"] = df14["PIM 3 - No High Risk Dx"].copy()
df16["PIM 3 - No Low Risk Dx"] = df14["PIM 3 - No Low Risk Dx"].copy()


df16.info()
```

```
<class 'pandas.core.frame.DataFrame'>
RangeIndex: 123354 entries, 0 to 123353
Data columns (total 23 columns):
 #   Column                              Non-Null Count   Dtype  
---  ------                              --------------   -----  
 0   Is Readmission                      123354 non-null  float64
 1   Age                                 123354 non-null  float64
 2   Weight                              123354 non-null  float64
 3   Gender                              123354 non-null  float64
 4   Race                                123354 non-null  float64
 5   Patient Origin                      123354 non-null  float64
 6   Trauma                              123354 non-null  float64
 7   Patient Type                        123354 non-null  float64
 8   Post Operative                      123354 non-null  float64
 9   Mechanical Ventilation (First Hour) 123354 non-null  float64
 10  Elective Admission to ICU           123354 non-null  float64
 11  PIM 3 Recovery from Surgery         123354 non-null  float64
 12  Category                            123354 non-null  float64
 13  Baseline PCPC                       123354 non-null  float64
 14  Baseline POPC                       123354 non-null  float64
 15  PIM3                                123354 non-null  float64
 16  PIM 3 Probability of Death          123354 non-null  float64
 17  Systolic Blood Pressure             123354 non-null  float64
 18  Base Excess                         123354 non-null  float64
 19  PIM 3 Pupillary Reaction            123354 non-null  float64
 20  PIM 3 - No Very High Risk Dx        123354 non-null  int64  
 21  PIM 3 - No High Risk Dx             123354 non-null  int64  
 22  PIM 3 - No Low Risk Dx              123354 non-null  int64  
dtypes: float64(20), int64(3)
memory usage: 21.6 MB
```

Add "LOS" into the dataframe and generate HEATMAP

In [36]:

```
df17 = df16.copy()

df17.insert(23, "LOS", 0)
df17.LOS = df14.LOS.copy()
```

In [37]:

```
df17.info()
```

```
<class 'pandas.core.frame.DataFrame'>
RangeIndex: 123354 entries, 0 to 123353
Data columns (total 24 columns):
 #   Column                                Non-Null Count   Dtype  
---  ------                                --------------   -----  
 0   Is Readmission                        123354 non-null  float64
 1   Age                                   123354 non-null  float64
 2   Weight                                123354 non-null  float64
 3   Gender                                123354 non-null  float64
 4   Race                                  123354 non-null  float64
 5   Patient Origin                        123354 non-null  float64
 6   Trauma                                123354 non-null  float64
 7   Patient Type                          123354 non-null  float64
 8   Post Operative                        123354 non-null  float64
 9   Mechanical Ventilation (First Hour)   123354 non-null  float64
 10  Elective Admission to ICU             123354 non-null  float64
 11  PIM 3 Recovery from Surgery           123354 non-null  float64
 12  Category                              123354 non-null  float64
 13  Baseline PCPC                         123354 non-null  float64
 14  Baseline POPC                         123354 non-null  float64
 15  PIM3                                  123354 non-null  float64
 16  PIM 3 Probability of Death            123354 non-null  float64
 17  Systolic Blood Pressure               123354 non-null  float64
 18  Base Excess                           123354 non-null  float64
 19  PIM 3 Pupillary Reaction              123354 non-null  float64
 20  PIM 3 - No Very High Risk Dx          123354 non-null  int64  
 21  PIM 3 - No High Risk Dx               123354 non-null  int64  
 22  PIM 3 - No Low Risk Dx                123354 non-null  int64  
 23  LOS                                   123354 non-null  int64  
dtypes: float64(20), int64(4)
memory usage: 22.6 MB
```

In [38]:

```
def heatMap(list, fontSize):
    corr = list.corr()
    corr= corr.round(decimals=2)
    plt.figure(figsize=(20, 8))
    sns.set(font_scale = 1.2)
    sns.heatmap(corr, cmap='RdBu', vmin=-1, vmax=1, annot=True, annot_kws={'fontsize':
```

In [39]:

```
heatMap(df17, 12)
```

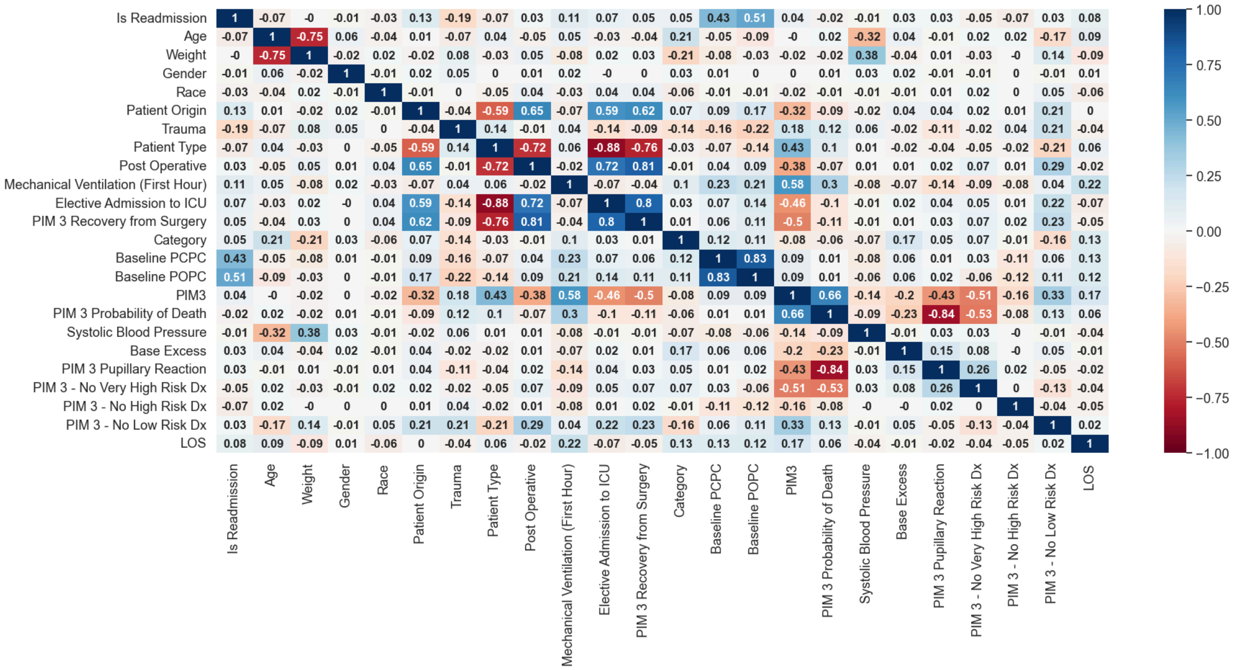

Create INPUT and OUTPUT NP arrays

In [78]:

```
input = df16.copy().to_numpy()
output = df17["LOS"].copy().to_numpy()

#input.head()
print('Input sample 25: \n' , input[27] ,  "\n \n Output sample 25: " , output[27])
```

```
Input sample 25: 
 [  0.      4.     10.8     2.      7.      1.      0.      1.      1.      1.
   0.      0.    625.      1.      2.     -1.06   25.68   89.      0.      1.
   0.      1.      1.   ]

 Output sample 25:  1
```

Apply SKLearn train/test split to Input and Output for 80:20 split

In [79]:

```
X_train, X_test, y_train, y_test = train_test_split(input, output, test_size=0.2, rand
```

In [38]:

```
print(X_train.shape, "\t", y_train.shape, "\n")
print(X_test.shape, "\t", y_test.shape, "\n")
```

```
(98685, 23)      (98685,)

(24672, 23)      (24672,)
```

Fit MinMaxScaler on Training data, and then apply transformation to training and test set

In [39]:

```
scaler = MinMaxScaler()
scaler.fit(X_train)
```

Out[39]:

```
▾ MinMaxScaler
MinMaxScaler()
```

In [40]:

```
X_train = scaler.transform(X_train)
X_test = scaler.transform(X_test)
```

In [41]:

```
print(X_test[27], '\n' '\n', y_test[27])
```

```
[1.         0.4        0.03930435 0.5        0.75       0.92857143
 0.         0.         1.         1.         1.         1.
 0.31406045 0.75       0.75       0.17475124 0.00410287 0.42918455
 0.38844847 1.         1.         1.         1.        ]

 1
```

Import and apply LinearSVC - results appear Encouraging

Attempted standard SVC with RBF kernel too but computationally exorbitant

In [42]:

```
from sklearn.svm import LinearSVC
```

In [43]:

```
svc = LinearSVC(dual="auto", random_state=0, tol=1e-5)
svc.fit(X_train, y_train)
```

Out[43]:

```
▾ LinearSVC
LinearSVC(dual='auto', random_state=0, tol=1e-05)
```

In [44]:

```
svc_predictions = svc.predict(X_test)
```

In [45]:

```
def plotCM(test, predictions):

    print("accuracy Score is: " + str(accuracy_score(test, predictions)))

    cm = confusion_matrix(test, predictions, normalize='true')
    fig, ax = plt.subplots(figsize=(2,2))
    ConfusionMatrixDisplay(cm).plot(ax=ax, colorbar=False)
    plt.title('Sens/Spec')
    plt.show()

    cm2 = confusion_matrix(test, predictions, normalize='pred')
    fig, ax = plt.subplots(figsize=(2,2))
    ConfusionMatrixDisplay(cm2).plot(ax=ax, colorbar=False)
    plt.title('NPV/PPV')
    plt.show()

    RocCurveDisplay.from_predictions(test, predictions)
    plt.show()
```

In [46]:

```
plotCM(y_test, svc_predictions)
```

```
accuracy Score is: 0.6859192607003891
```

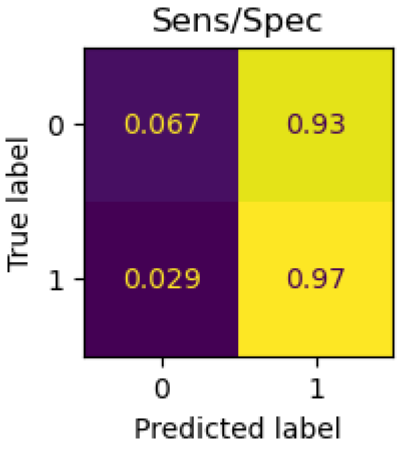

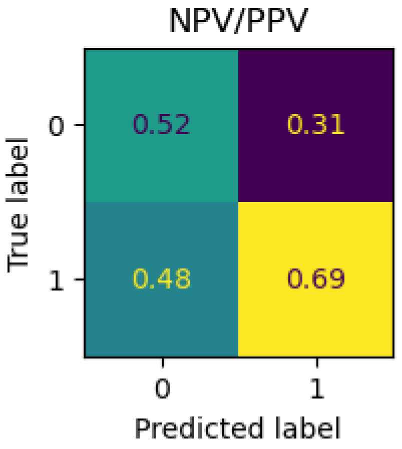

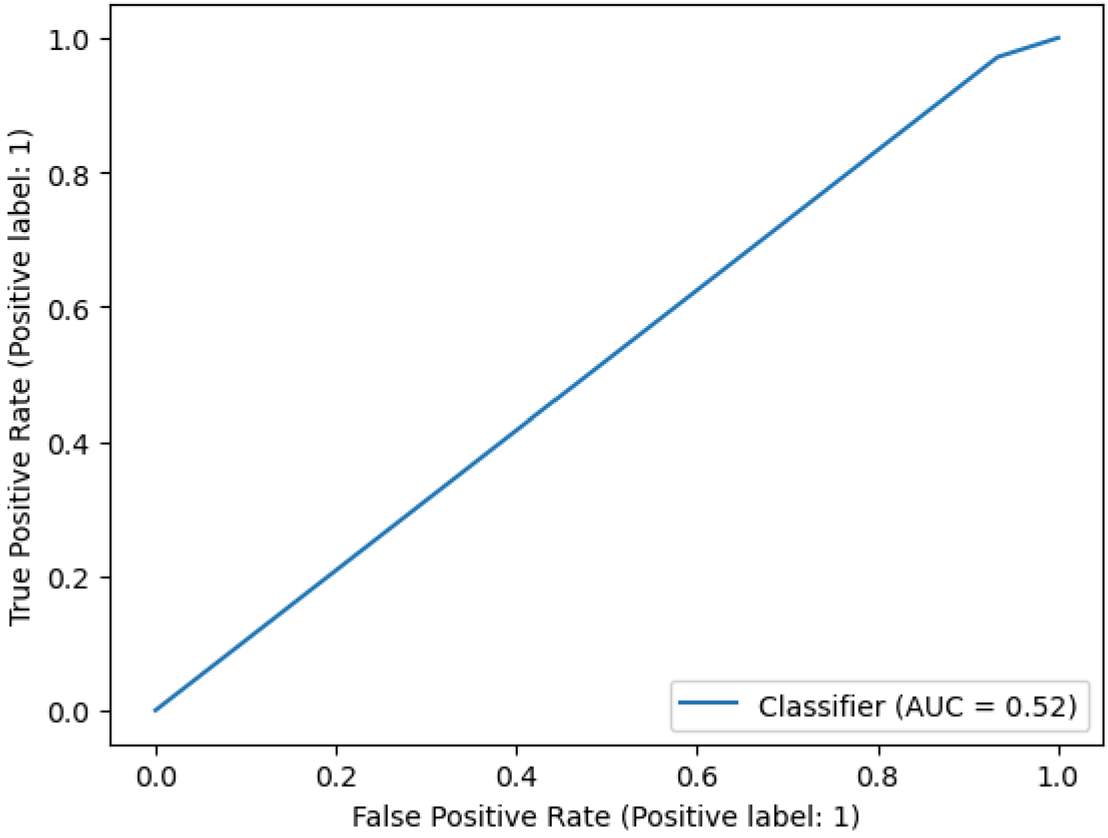

Import and apply Stochastic Gradient Descent Classification - results seen

In [47]:
```
from sklearn.linear_model import SGDClassifier
```

In [48]:
```
sgdc = SGDClassifier(loss="hinge", penalty="l2")
sgdc.fit(X_train, y_train)
```

Out[48]:
```
▾ SGDClassifier
SGDClassifier()
```

In [49]:
```
sgdc_predictions = sgdc.predict(X_test)
```

In [50]:
```
plotCM(y_test, sgdc_predictions)
```

```
accuracy Score is: 0.684257457846952
```

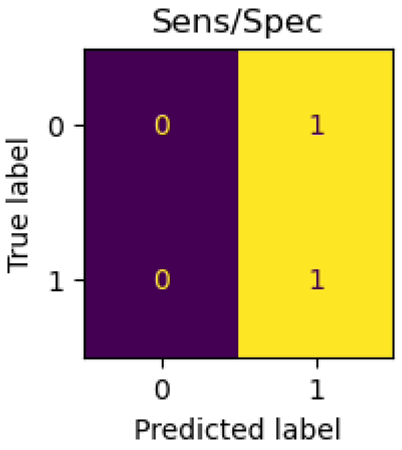

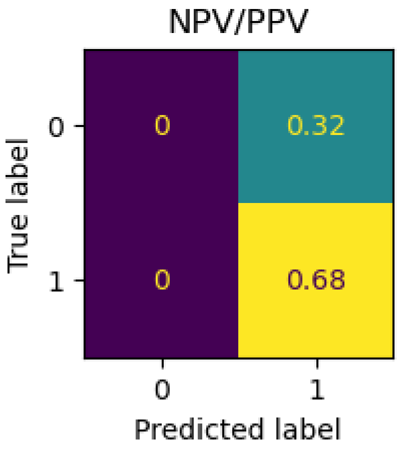

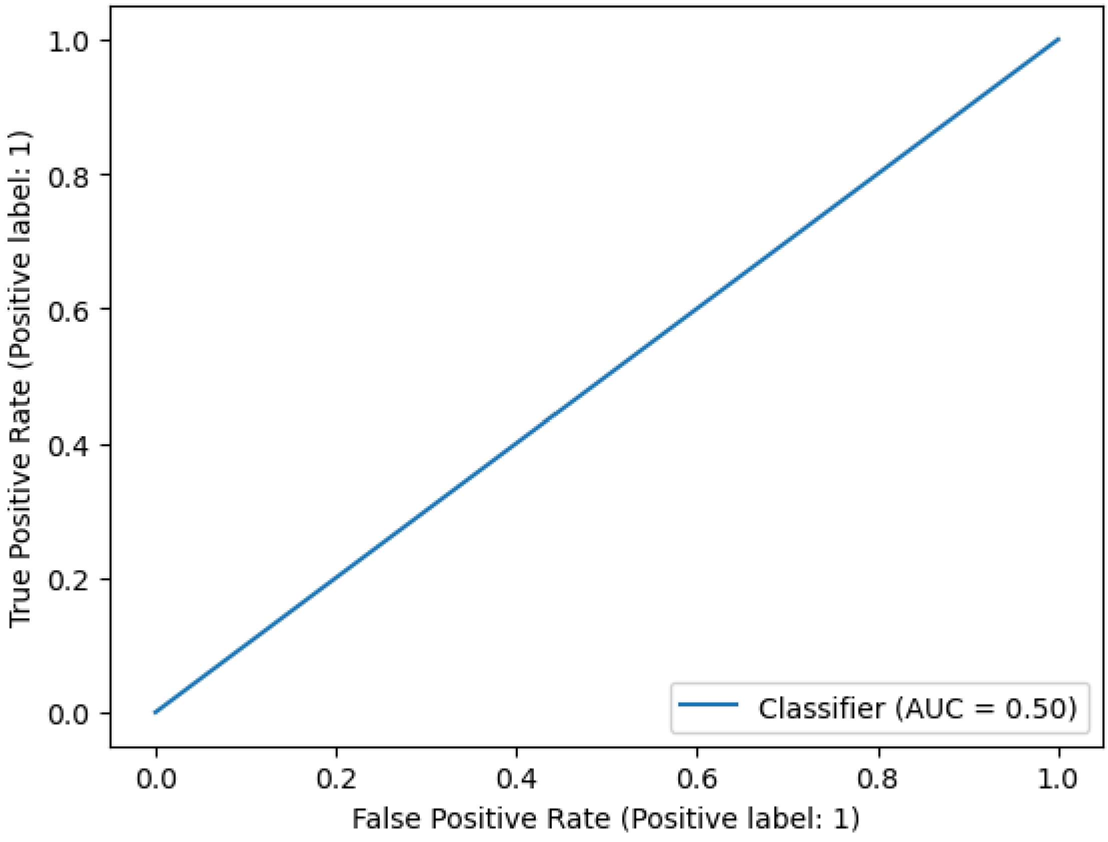

Import and apply KNN Classifier - results

In [51]:
```
from sklearn.neighbors import KNeighborsClassifier
```

In [52]:
```
knn = KNeighborsClassifier(n_neighbors=15)
knn.fit(X_train, y_train)
```

Out[52]:
```
▾ KNeighborsClassifier
KNeighborsClassifier(n_neighbors=15)
```

In [53]:
```
knn_predictions = knn.predict(X_test)
```

In [54]:
```
plotCM(y_test, knn_predictions)
```

```
accuracy Score is: 0.7064283398184177
```

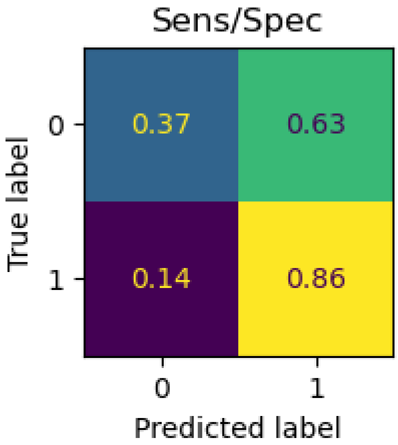

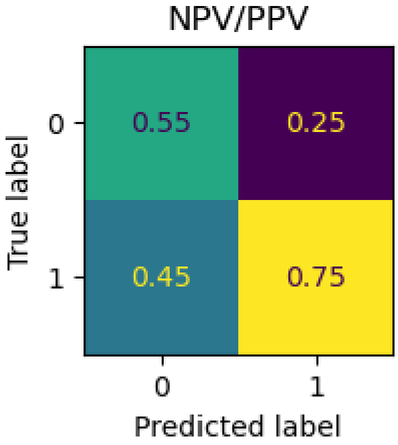

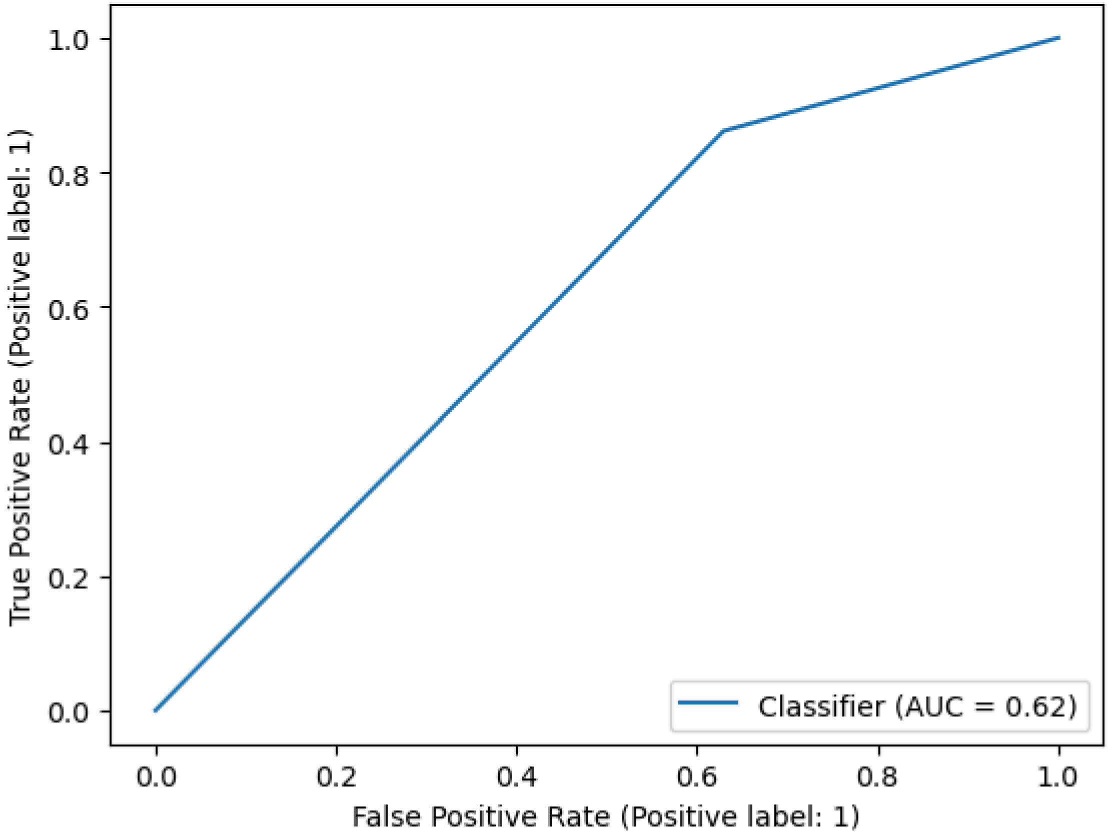

Import and apply Decision Tree Regression - results appear to be GARBAGE

In [55]:
```
from sklearn.tree import DecisionTreeClassifier
```

In [56]:
```
dtree = DecisionTreeClassifier(random_state=30)
dtree.fit(X_train, y_train)
```

Out[56]:
```
▾ DecisionTreeClassifier
DecisionTreeClassifier(random_state=30)
```

In [57]:
```
dtree_predictions = dtree.predict(X_test)
```

In [58]:
```
plotCM(y_test, dtree_predictions)
```

```
accuracy Score is: 0.6487110894941635
```

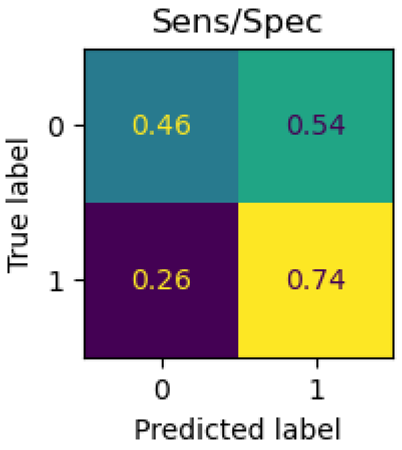

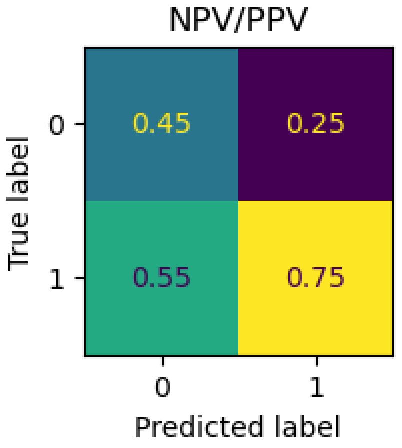

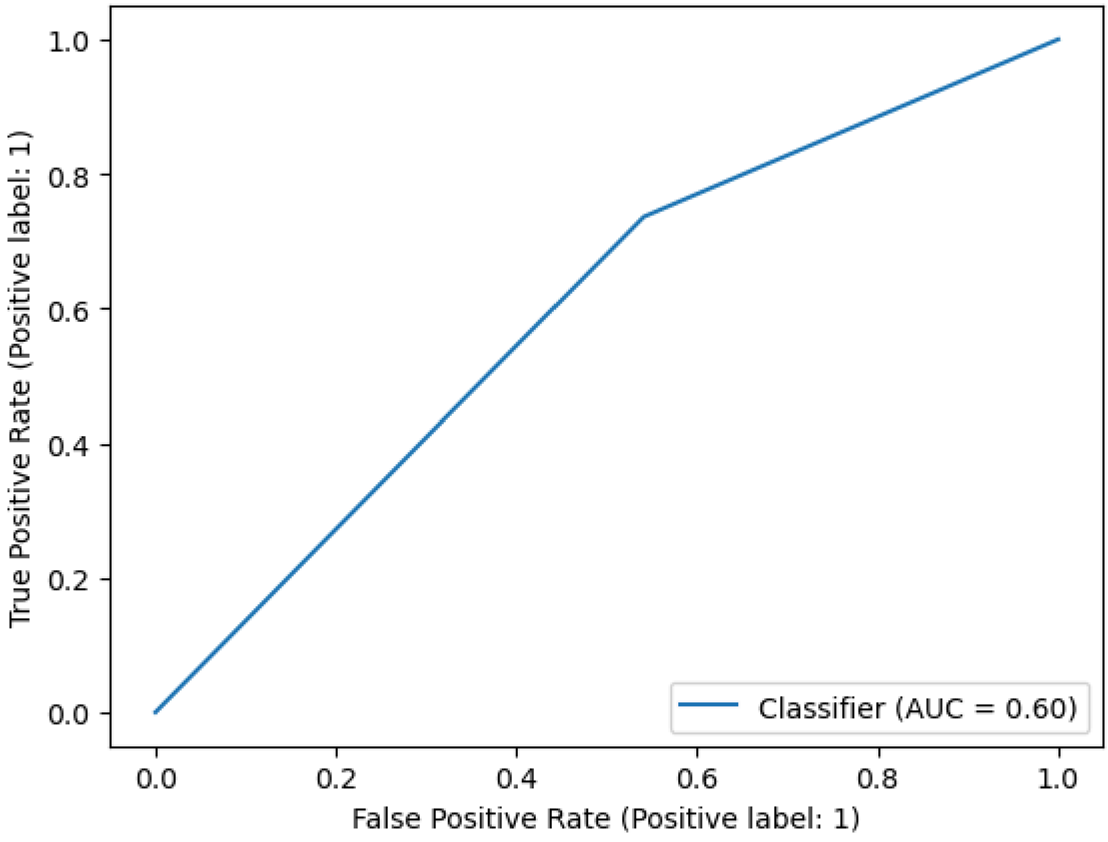

Attempt Gradient Boosting

In [59]:
```
from sklearn.ensemble import HistGradientBoostingClassifier
```

In [60]:
```
boost = HistGradientBoostingClassifier()
boost.fit(X_train, y_train)
```

Out[60]:
```
▾ HistGradientBoostingClassifier
HistGradientBoostingClassifier()
```

In [61]:
```
boost_predictions = boost.predict(X_test)
```

In [62]:
```
plotCM(y_test, boost_predictions)
```

```
accuracy Score is: 0.7282749675745784
```

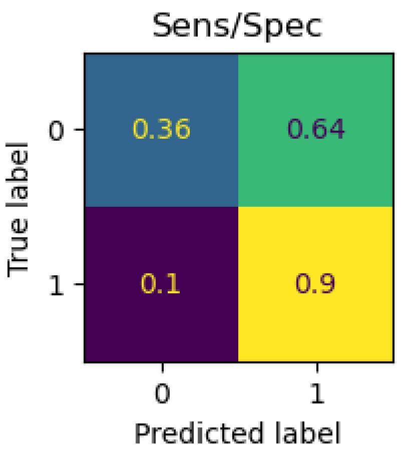

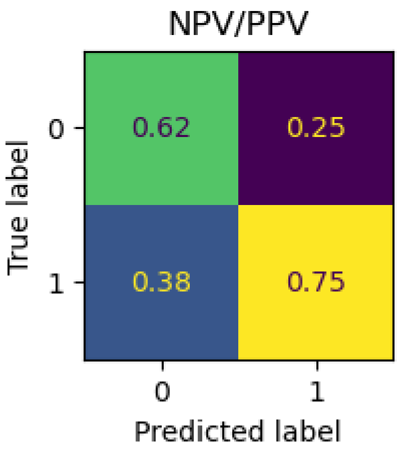

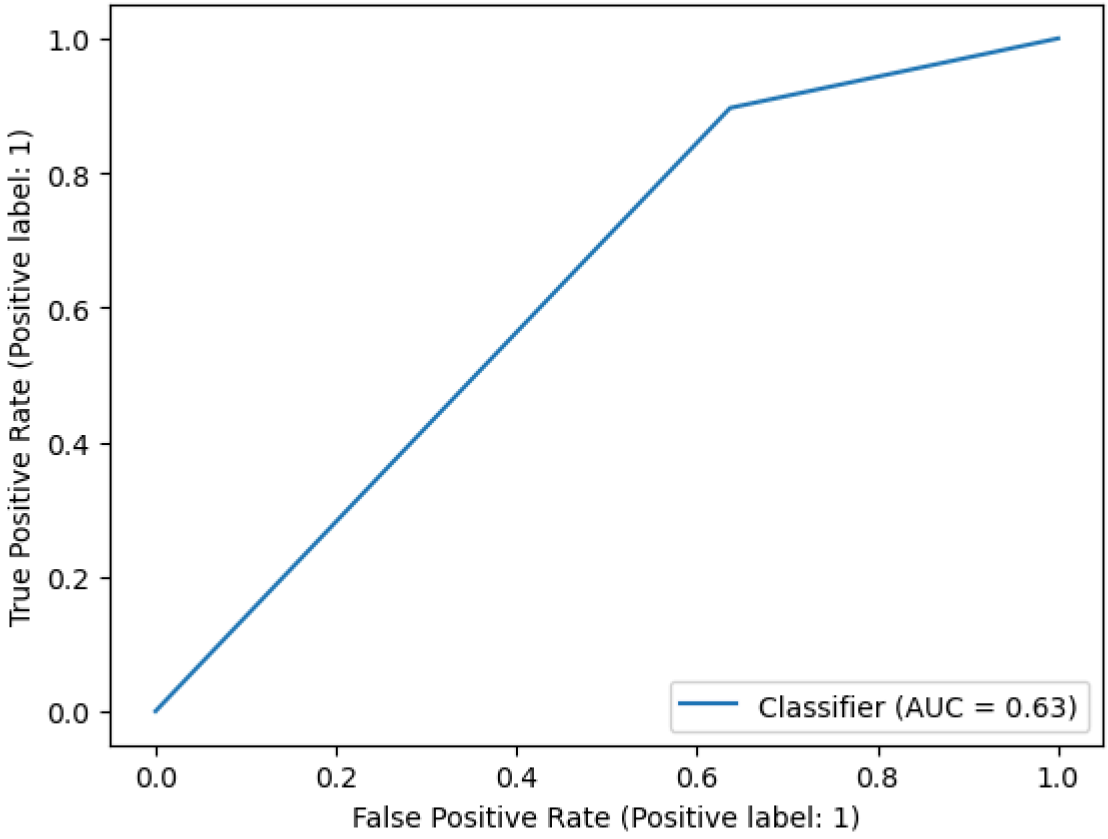

Attempt CatBoost

In [63]:
```
from catboost import CatBoostClassifier
import warnings
warnings.filterwarnings("ignore")
```

In [64]:
```
# Define the hyperparameters for the CatBoost algorithm
params = {'learning_rate': 0.1, 'depth': 6,
          'l2_leaf_reg': 3, 'iterations': 100}

# Initialize the CatBoostClassifier object
# with the defined hyperparameters and fit it on the training set
model = CatBoostClassifier(**params)
model.fit(X_train, y_train)
```

```
0:	learn: 0.6656172	total: 161ms	remaining: 15.9s
1:	learn: 0.6441216	total: 180ms	remaining: 8.81s
2:	learn: 0.6287034	total: 201ms	remaining: 6.5s
3:	learn: 0.6155649	total: 222ms	remaining: 5.33s
4:	learn: 0.6043451	total: 243ms	remaining: 4.61s
5:	learn: 0.5961066	total: 263ms	remaining: 4.12s
6:	learn: 0.5883128	total: 282ms	remaining: 3.75s
7:	learn: 0.5822448	total: 307ms	remaining: 3.53s
8:	learn: 0.5786047	total: 323ms	remaining: 3.27s
9:	learn: 0.5749065	total: 343ms	remaining: 3.08s
10:	learn: 0.5711309	total: 368ms	remaining: 2.98s
11:	learn: 0.5678365	total: 393ms	remaining: 2.88s
12:	learn: 0.5644850	total: 415ms	remaining: 2.78s
13:	learn: 0.5622424	total: 437ms	remaining: 2.68s
14:	learn: 0.5600662	total: 457ms	remaining: 2.59s
15:	learn: 0.5582233	total: 475ms	remaining: 2.49s
16:	learn: 0.5566732	total: 498ms	remaining: 2.43s
17:	learn: 0.5555896	total: 520ms	remaining: 2.37s
18:	learn: 0.5542079	total: 543ms	remaining: 2.31s
19:	learn: 0.5529211	total: 566ms	remaining: 2.27s
20:	learn: 0.5519199	total: 590ms	remaining: 2.22s
21:	learn: 0.5509396	total: 616ms	remaining: 2.18s
22:	learn: 0.5500828	total: 639ms	remaining: 2.14s
23:	learn: 0.5493715	total: 662ms	remaining: 2.1s
24:	learn: 0.5487110	total: 682ms	remaining: 2.05s
25:	learn: 0.5480874	total: 705ms	remaining: 2s
26:	learn: 0.5473230	total: 727ms	remaining: 1.97s
27:	learn: 0.5465291	total: 755ms	remaining: 1.94s
28:	learn: 0.5459477	total: 784ms	remaining: 1.92s
29:	learn: 0.5453840	total: 824ms	remaining: 1.92s
30:	learn: 0.5445688	total: 860ms	remaining: 1.91s
31:	learn: 0.5440429	total: 892ms	remaining: 1.9s
32:	learn: 0.5433219	total: 929ms	remaining: 1.89s
33:	learn: 0.5430052	total: 967ms	remaining: 1.88s
34:	learn: 0.5425785	total: 998ms	remaining: 1.85s
35:	learn: 0.5422308	total: 1.03s	remaining: 1.83s
36:	learn: 0.5417312	total: 1.06s	remaining: 1.81s
37:	learn: 0.5413722	total: 1.1s	remaining: 1.79s
38:	learn: 0.5407876	total: 1.12s	remaining: 1.76s
39:	learn: 0.5405931	total: 1.15s	remaining: 1.73s
40:	learn: 0.5402257	total: 1.18s	remaining: 1.69s
41:	learn: 0.5399617	total: 1.2s	remaining: 1.66s
42:	learn: 0.5396332	total: 1.23s	remaining: 1.63s
43:	learn: 0.5392112	total: 1.26s	remaining: 1.61s
44:	learn: 0.5389881	total: 1.29s	remaining: 1.58s
45:	learn: 0.5386188	total: 1.32s	remaining: 1.55s
46:	learn: 0.5383275	total: 1.35s	remaining: 1.53s
47:	learn: 0.5379114	total: 1.38s	remaining: 1.5s
48:	learn: 0.5376693	total: 1.43s	remaining: 1.48s
49:	learn: 0.5374767	total: 1.45s	remaining: 1.45s
50:	learn: 0.5372688	total: 1.48s	remaining: 1.43s
51:	learn: 0.5370606	total: 1.51s	remaining: 1.39s
52:	learn: 0.5368211	total: 1.54s	remaining: 1.36s
53:	learn: 0.5366902	total: 1.56s	remaining: 1.33s
54:	learn: 0.5364824	total: 1.59s	remaining: 1.3s
55:	learn: 0.5360395	total: 1.61s	remaining: 1.27s
56:	learn: 0.5357664	total: 1.63s	remaining: 1.23s
57:	learn: 0.5356169	total: 1.66s	remaining: 1.2s
58:	learn: 0.5354206	total: 1.68s	remaining: 1.17s
59:	learn: 0.5352781	total: 1.71s	remaining: 1.14s
```

```
60:	learn: 0.5351332	total: 1.74s	remaining: 1.11s
61:	learn: 0.5349110	total: 1.76s	remaining: 1.08s
62:	learn: 0.5346770	total: 1.8s	remaining: 1.06s
63:	learn: 0.5344514	total: 1.83s	remaining: 1.03s
64:	learn: 0.5343071	total: 1.86s	remaining: 1s
65:	learn: 0.5341750	total: 1.89s	remaining: 972ms
66:	learn: 0.5340043	total: 1.91s	remaining: 943ms
67:	learn: 0.5338816	total: 1.94s	remaining: 913ms
68:	learn: 0.5337319	total: 1.96s	remaining: 882ms
69:	learn: 0.5334418	total: 1.99s	remaining: 852ms
70:	learn: 0.5331919	total: 2.01s	remaining: 822ms
71:	learn: 0.5329861	total: 2.04s	remaining: 792ms
72:	learn: 0.5327404	total: 2.06s	remaining: 761ms
73:	learn: 0.5324622	total: 2.08s	remaining: 730ms
74:	learn: 0.5323235	total: 2.1s	remaining: 701ms
75:	learn: 0.5322257	total: 2.13s	remaining: 672ms
76:	learn: 0.5320197	total: 2.15s	remaining: 643ms
77:	learn: 0.5317864	total: 2.17s	remaining: 613ms
78:	learn: 0.5315788	total: 2.2s	remaining: 584ms
79:	learn: 0.5314988	total: 2.22s	remaining: 555ms
80:	learn: 0.5312469	total: 2.24s	remaining: 525ms
81:	learn: 0.5310927	total: 2.26s	remaining: 497ms
82:	learn: 0.5309195	total: 2.28s	remaining: 468ms
83:	learn: 0.5308103	total: 2.3s	remaining: 439ms
84:	learn: 0.5307211	total: 2.32s	remaining: 410ms
85:	learn: 0.5305007	total: 2.35s	remaining: 383ms
86:	learn: 0.5304245	total: 2.37s	remaining: 355ms
87:	learn: 0.5302886	total: 2.4s	remaining: 327ms
88:	learn: 0.5301906	total: 2.42s	remaining: 299ms
89:	learn: 0.5301171	total: 2.44s	remaining: 271ms
90:	learn: 0.5299657	total: 2.46s	remaining: 243ms
91:	learn: 0.5297738	total: 2.48s	remaining: 216ms
92:	learn: 0.5296761	total: 2.5s	remaining: 188ms
93:	learn: 0.5295585	total: 2.52s	remaining: 161ms
94:	learn: 0.5293815	total: 2.55s	remaining: 134ms
95:	learn: 0.5292699	total: 2.58s	remaining: 107ms
96:	learn: 0.5291769	total: 2.61s	remaining: 80.6ms
97:	learn: 0.5289708	total: 2.64s	remaining: 53.8ms
98:	learn: 0.5288920	total: 2.66s	remaining: 26.9ms
99:	learn: 0.5287461	total: 2.69s	remaining: 0us
```

Out[64]:
```
<catboost.core.CatBoostClassifier at 0x2322820d610>
```

In [65]:
```
y_pred = model.predict(X_test)
```

In [66]:
```
plotCM(y_test, y_pred)
```

```
accuracy Score is: 0.723613813229572
```

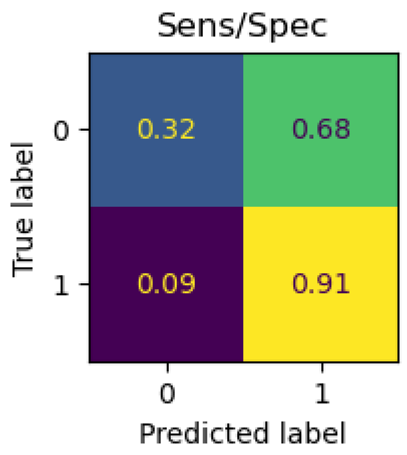

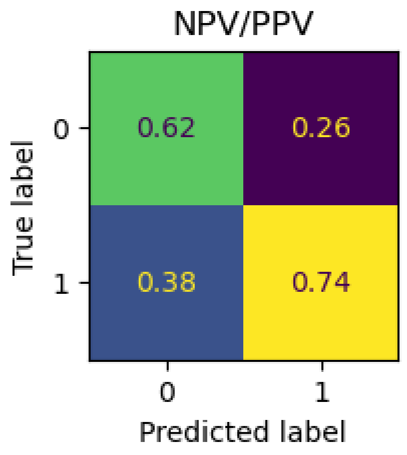

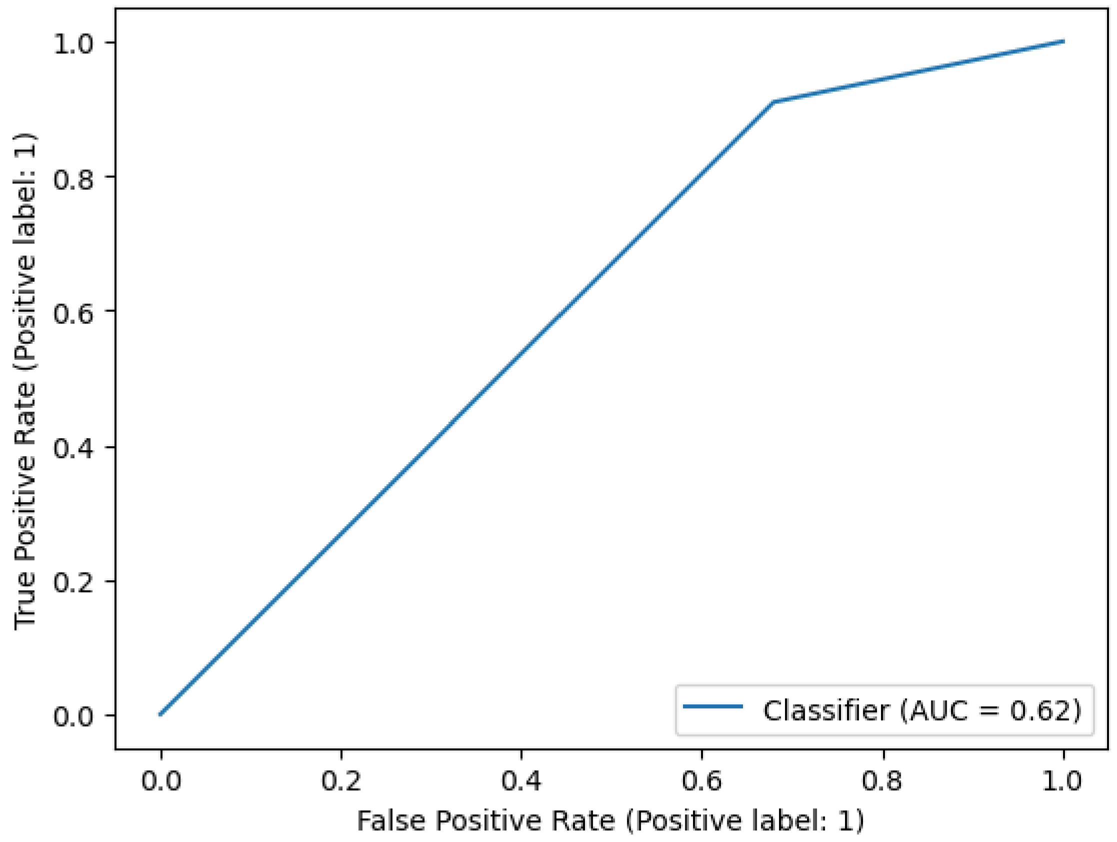

Neural Network

split Training data again in 75:25 ration to generate a total 60:20:20 split for Train:Validate:Test

In [67]:
```
X_train, X_val, y_train, y_val = train_test_split(X_train, y_train, test_size=0.25, ra
```

In [68]:
```
# Define function to plot Training Loss vs Validation Loss across epochs
def drawPlot():
    plt.plot(history.history['loss'])
    plt.plot(history.history['val_loss'])
    plt.title('model Loss')
    plt.ylabel('Loss')
    plt.xlabel('Epoch')
    #plt.xticks(np.arange(0, 21, 1.0))
    plt.legend(['train', 'val'], loc='upper left')
    plt.show()
```

In [71]:
```
drawPlot()
```

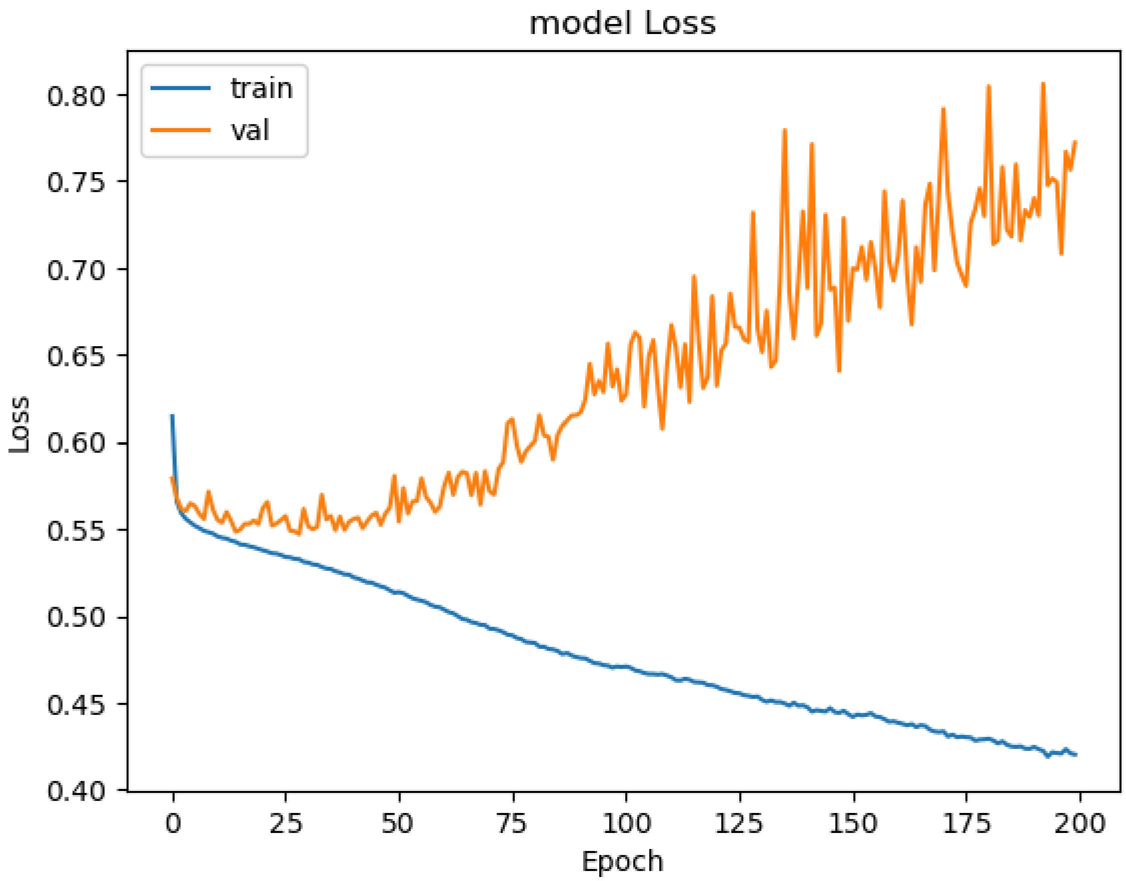

In [75]:
```
network = models.Sequential()
network.add(layers.Dense(512, activation='relu', input_shape=(23, )))
network.add(layers.Dense(256, activation='relu'))
network.add(layers.Dense(128, activation='relu'))
network.add(layers.Dense(64, activation='relu'))
network.add(layers.Dense(32, activation='relu'))
network.add(layers.Dense(16, activation='relu'))
network.add(layers.Dense(8, activation='relu'))
network.add(layers.Dense(4, activation='relu'))
network.add(layers.Dense(2, activation='relu'))
network.add(layers.Dense(1, activation='sigmoid'))
network.compile(optimizer=optimizers.RMSprop(),
```

```
                 loss='binary_crossentropy',
                 metrics=['accuracy'])
```

In [70]:
```
# fit model
history = network.fit(X_train, y_train,
                      batch_size=128, epochs=200,
                      validation_data = (X_val, y_val))
```

```
Epoch 1/200
579/579 [==============================] - 3s 4ms/step - loss: 0.6147 - accuracy: 0.6
861 - val_loss: 0.5792 - val_accuracy: 0.6982
Epoch 2/200
579/579 [==============================] - 3s 5ms/step - loss: 0.5659 - accuracy: 0.7
034 - val_loss: 0.5679 - val_accuracy: 0.6989
Epoch 3/200
579/579 [==============================] - 3s 4ms/step - loss: 0.5591 - accuracy: 0.7
105 - val_loss: 0.5614 - val_accuracy: 0.7099
Epoch 4/200
579/579 [==============================] - 3s 5ms/step - loss: 0.5558 - accuracy: 0.7
144 - val_loss: 0.5605 - val_accuracy: 0.7109
Epoch 5/200
579/579 [==============================] - 3s 5ms/step - loss: 0.5538 - accuracy: 0.7
167 - val_loss: 0.5648 - val_accuracy: 0.7170
Epoch 6/200
579/579 [==============================] - 3s 5ms/step - loss: 0.5519 - accuracy: 0.7
178 - val_loss: 0.5632 - val_accuracy: 0.7091
Epoch 7/200
579/579 [==============================] - 3s 5ms/step - loss: 0.5505 - accuracy: 0.7
181 - val_loss: 0.5588 - val_accuracy: 0.7137
Epoch 8/200
579/579 [==============================] - 3s 4ms/step - loss: 0.5490 - accuracy: 0.7
189 - val_loss: 0.5558 - val_accuracy: 0.7156
Epoch 9/200
579/579 [==============================] - 3s 5ms/step - loss: 0.5482 - accuracy: 0.7
185 - val_loss: 0.5715 - val_accuracy: 0.6940
Epoch 10/200
579/579 [==============================] - 2s 4ms/step - loss: 0.5474 - accuracy: 0.7
193 - val_loss: 0.5611 - val_accuracy: 0.7151
Epoch 11/200
579/579 [==============================] - 2s 4ms/step - loss: 0.5458 - accuracy: 0.7
216 - val_loss: 0.5553 - val_accuracy: 0.7189
Epoch 12/200
579/579 [==============================] - 3s 4ms/step - loss: 0.5450 - accuracy: 0.7
200 - val_loss: 0.5537 - val_accuracy: 0.7172
Epoch 13/200
579/579 [==============================] - 3s 5ms/step - loss: 0.5445 - accuracy: 0.7
208 - val_loss: 0.5598 - val_accuracy: 0.7117
Epoch 14/200
579/579 [==============================] - 3s 5ms/step - loss: 0.5433 - accuracy: 0.7
220 - val_loss: 0.5546 - val_accuracy: 0.7138
Epoch 15/200
579/579 [==============================] - 2s 4ms/step - loss: 0.5426 - accuracy: 0.7
219 - val_loss: 0.5484 - val_accuracy: 0.7178
Epoch 16/200
579/579 [==============================] - 2s 4ms/step - loss: 0.5412 - accuracy: 0.7
227 - val_loss: 0.5497 - val_accuracy: 0.7168
Epoch 17/200
579/579 [==============================] - 3s 4ms/step - loss: 0.5409 - accuracy: 0.7
230 - val_loss: 0.5530 - val_accuracy: 0.7168
Epoch 18/200
579/579 [==============================] - 2s 4ms/step - loss: 0.5401 - accuracy: 0.7
247 - val_loss: 0.5529 - val_accuracy: 0.7166
Epoch 19/200
579/579 [==============================] - 3s 4ms/step - loss: 0.5395 - accuracy: 0.7
238 - val_loss: 0.5549 - val_accuracy: 0.7178
Epoch 20/200
579/579 [==============================] - 3s 5ms/step - loss: 0.5386 - accuracy: 0.7
246 - val_loss: 0.5528 - val_accuracy: 0.7170
```

```
Epoch 21/200
579/579 [==============================] - 2s 4ms/step - loss: 0.5379 - accuracy: 0.7
245 - val_loss: 0.5620 - val_accuracy: 0.7188
Epoch 22/200
579/579 [==============================] - 2s 4ms/step - loss: 0.5370 - accuracy: 0.7
246 - val_loss: 0.5656 - val_accuracy: 0.7107
Epoch 23/200
579/579 [==============================] - 2s 4ms/step - loss: 0.5361 - accuracy: 0.7
242 - val_loss: 0.5520 - val_accuracy: 0.7153
Epoch 24/200
579/579 [==============================] - 2s 4ms/step - loss: 0.5359 - accuracy: 0.7
249 - val_loss: 0.5532 - val_accuracy: 0.7123
Epoch 25/200
579/579 [==============================] - 2s 4ms/step - loss: 0.5350 - accuracy: 0.7
261 - val_loss: 0.5552 - val_accuracy: 0.7172
Epoch 26/200
579/579 [==============================] - 3s 4ms/step - loss: 0.5340 - accuracy: 0.7
272 - val_loss: 0.5575 - val_accuracy: 0.7184
Epoch 27/200
579/579 [==============================] - 2s 4ms/step - loss: 0.5337 - accuracy: 0.7
267 - val_loss: 0.5491 - val_accuracy: 0.7172
Epoch 28/200
579/579 [==============================] - 3s 5ms/step - loss: 0.5328 - accuracy: 0.7
265 - val_loss: 0.5487 - val_accuracy: 0.7180
Epoch 29/200
579/579 [==============================] - 2s 4ms/step - loss: 0.5327 - accuracy: 0.7
271 - val_loss: 0.5471 - val_accuracy: 0.7204
Epoch 30/200
579/579 [==============================] - 3s 5ms/step - loss: 0.5311 - accuracy: 0.7
286 - val_loss: 0.5616 - val_accuracy: 0.7206
Epoch 31/200
579/579 [==============================] - 3s 5ms/step - loss: 0.5307 - accuracy: 0.7
286 - val_loss: 0.5516 - val_accuracy: 0.7170
Epoch 32/200
579/579 [==============================] - 3s 4ms/step - loss: 0.5297 - accuracy: 0.7
288 - val_loss: 0.5499 - val_accuracy: 0.7192
Epoch 33/200
579/579 [==============================] - 3s 5ms/step - loss: 0.5294 - accuracy: 0.7
302 - val_loss: 0.5514 - val_accuracy: 0.7221
Epoch 34/200
579/579 [==============================] - 3s 5ms/step - loss: 0.5282 - accuracy: 0.7
298 - val_loss: 0.5697 - val_accuracy: 0.7189
Epoch 35/200
579/579 [==============================] - 3s 4ms/step - loss: 0.5273 - accuracy: 0.7
297 - val_loss: 0.5556 - val_accuracy: 0.7088
Epoch 36/200
579/579 [==============================] - 3s 4ms/step - loss: 0.5270 - accuracy: 0.7
297 - val_loss: 0.5575 - val_accuracy: 0.7219
Epoch 37/200
579/579 [==============================] - 3s 5ms/step - loss: 0.5257 - accuracy: 0.7
313 - val_loss: 0.5494 - val_accuracy: 0.7192
Epoch 38/200
579/579 [==============================] - 2s 4ms/step - loss: 0.5250 - accuracy: 0.7
306 - val_loss: 0.5572 - val_accuracy: 0.7146
Epoch 39/200
579/579 [==============================] - 2s 4ms/step - loss: 0.5239 - accuracy: 0.7
319 - val_loss: 0.5495 - val_accuracy: 0.7171
Epoch 40/200
579/579 [==============================] - 3s 5ms/step - loss: 0.5238 - accuracy: 0.7
326 - val_loss: 0.5541 - val_accuracy: 0.7141
```

```
Epoch 41/200
579/579 [==============================] - 2s 4ms/step - loss: 0.5222 - accuracy: 0.7
331 - val_loss: 0.5557 - val_accuracy: 0.7199
Epoch 42/200
579/579 [==============================] - 3s 4ms/step - loss: 0.5215 - accuracy: 0.7
337 - val_loss: 0.5565 - val_accuracy: 0.7186
Epoch 43/200
579/579 [==============================] - 3s 4ms/step - loss: 0.5205 - accuracy: 0.7
339 - val_loss: 0.5505 - val_accuracy: 0.7189
Epoch 44/200
579/579 [==============================] - 3s 4ms/step - loss: 0.5194 - accuracy: 0.7
347 - val_loss: 0.5544 - val_accuracy: 0.7169
Epoch 45/200
579/579 [==============================] - 3s 5ms/step - loss: 0.5192 - accuracy: 0.7
336 - val_loss: 0.5578 - val_accuracy: 0.7180
Epoch 46/200
579/579 [==============================] - 3s 5ms/step - loss: 0.5180 - accuracy: 0.7
352 - val_loss: 0.5593 - val_accuracy: 0.7133
Epoch 47/200
579/579 [==============================] - 4s 6ms/step - loss: 0.5170 - accuracy: 0.7
367 - val_loss: 0.5525 - val_accuracy: 0.7165
Epoch 48/200
579/579 [==============================] - 4s 6ms/step - loss: 0.5163 - accuracy: 0.7
370 - val_loss: 0.5588 - val_accuracy: 0.7124
Epoch 49/200
579/579 [==============================] - 3s 6ms/step - loss: 0.5147 - accuracy: 0.7
378 - val_loss: 0.5623 - val_accuracy: 0.7143
Epoch 50/200
579/579 [==============================] - 3s 6ms/step - loss: 0.5134 - accuracy: 0.7
368 - val_loss: 0.5805 - val_accuracy: 0.7144
Epoch 51/200
579/579 [==============================] - 3s 5ms/step - loss: 0.5137 - accuracy: 0.7
385 - val_loss: 0.5543 - val_accuracy: 0.7179
Epoch 52/200
579/579 [==============================] - 3s 5ms/step - loss: 0.5131 - accuracy: 0.7
387 - val_loss: 0.5736 - val_accuracy: 0.7160
Epoch 53/200
579/579 [==============================] - 4s 6ms/step - loss: 0.5115 - accuracy: 0.7
396 - val_loss: 0.5589 - val_accuracy: 0.7179
Epoch 54/200
579/579 [==============================] - 3s 6ms/step - loss: 0.5102 - accuracy: 0.7
399 - val_loss: 0.5658 - val_accuracy: 0.7134
Epoch 55/200
579/579 [==============================] - 4s 7ms/step - loss: 0.5094 - accuracy: 0.7
401 - val_loss: 0.5662 - val_accuracy: 0.7046
Epoch 56/200
579/579 [==============================] - 3s 6ms/step - loss: 0.5087 - accuracy: 0.7
415 - val_loss: 0.5792 - val_accuracy: 0.7057
Epoch 57/200
579/579 [==============================] - 3s 6ms/step - loss: 0.5079 - accuracy: 0.7
423 - val_loss: 0.5684 - val_accuracy: 0.7099
Epoch 58/200
579/579 [==============================] - 3s 6ms/step - loss: 0.5063 - accuracy: 0.7
433 - val_loss: 0.5650 - val_accuracy: 0.7185
Epoch 59/200
579/579 [==============================] - 3s 6ms/step - loss: 0.5054 - accuracy: 0.7
437 - val_loss: 0.5598 - val_accuracy: 0.7163
Epoch 60/200
579/579 [==============================] - 4s 6ms/step - loss: 0.5050 - accuracy: 0.7
426 - val_loss: 0.5628 - val_accuracy: 0.7194
```

```
Epoch 61/200
579/579 [==============================] - 4s 6ms/step - loss: 0.5036 - accuracy: 0.7
447 - val_loss: 0.5748 - val_accuracy: 0.7170
Epoch 62/200
579/579 [==============================] - 4s 7ms/step - loss: 0.5022 - accuracy: 0.7
444 - val_loss: 0.5824 - val_accuracy: 0.7175
Epoch 63/200
579/579 [==============================] - 4s 6ms/step - loss: 0.5015 - accuracy: 0.7
452 - val_loss: 0.5697 - val_accuracy: 0.7135
Epoch 64/200
579/579 [==============================] - 4s 7ms/step - loss: 0.5000 - accuracy: 0.7
451 - val_loss: 0.5799 - val_accuracy: 0.7110
Epoch 65/200
579/579 [==============================] - 4s 6ms/step - loss: 0.4983 - accuracy: 0.7
469 - val_loss: 0.5830 - val_accuracy: 0.7186
Epoch 66/200
579/579 [==============================] - 4s 6ms/step - loss: 0.4978 - accuracy: 0.7
476 - val_loss: 0.5820 - val_accuracy: 0.7090
Epoch 67/200
579/579 [==============================] - 4s 7ms/step - loss: 0.4965 - accuracy: 0.7
486 - val_loss: 0.5695 - val_accuracy: 0.7156
Epoch 68/200
579/579 [==============================] - 4s 6ms/step - loss: 0.4959 - accuracy: 0.7
482 - val_loss: 0.5823 - val_accuracy: 0.7110
Epoch 69/200
579/579 [==============================] - 4s 6ms/step - loss: 0.4949 - accuracy: 0.7
496 - val_loss: 0.5642 - val_accuracy: 0.7136
Epoch 70/200
579/579 [==============================] - 4s 7ms/step - loss: 0.4947 - accuracy: 0.7
494 - val_loss: 0.5833 - val_accuracy: 0.7138
Epoch 71/200
579/579 [==============================] - 4s 6ms/step - loss: 0.4927 - accuracy: 0.7
504 - val_loss: 0.5718 - val_accuracy: 0.7142
Epoch 72/200
579/579 [==============================] - 3s 6ms/step - loss: 0.4925 - accuracy: 0.7
506 - val_loss: 0.5699 - val_accuracy: 0.7120
Epoch 73/200
579/579 [==============================] - 3s 5ms/step - loss: 0.4918 - accuracy: 0.7
513 - val_loss: 0.5845 - val_accuracy: 0.7104
Epoch 74/200
579/579 [==============================] - 3s 6ms/step - loss: 0.4907 - accuracy: 0.7
506 - val_loss: 0.5884 - val_accuracy: 0.7115
Epoch 75/200
579/579 [==============================] - 3s 6ms/step - loss: 0.4892 - accuracy: 0.7
524 - val_loss: 0.6107 - val_accuracy: 0.7134
Epoch 76/200
579/579 [==============================] - 3s 5ms/step - loss: 0.4889 - accuracy: 0.7
531 - val_loss: 0.6130 - val_accuracy: 0.7088
Epoch 77/200
579/579 [==============================] - 4s 6ms/step - loss: 0.4873 - accuracy: 0.7
531 - val_loss: 0.5982 - val_accuracy: 0.7063
Epoch 78/200
579/579 [==============================] - 3s 6ms/step - loss: 0.4867 - accuracy: 0.7
537 - val_loss: 0.5886 - val_accuracy: 0.7121
Epoch 79/200
579/579 [==============================] - 4s 6ms/step - loss: 0.4850 - accuracy: 0.7
543 - val_loss: 0.5946 - val_accuracy: 0.7102
Epoch 80/200
579/579 [==============================] - 3s 6ms/step - loss: 0.4848 - accuracy: 0.7
555 - val_loss: 0.5978 - val_accuracy: 0.7123
```

```
Epoch 81/200
579/579 [==============================] - 4s 8ms/step - loss: 0.4845 - accuracy: 0.7
560 - val_loss: 0.6002 - val_accuracy: 0.7130
Epoch 82/200
579/579 [==============================] - 4s 7ms/step - loss: 0.4823 - accuracy: 0.7
563 - val_loss: 0.6153 - val_accuracy: 0.7147
Epoch 83/200
579/579 [==============================] - 4s 7ms/step - loss: 0.4823 - accuracy: 0.7
563 - val_loss: 0.6036 - val_accuracy: 0.7136
Epoch 84/200
579/579 [==============================] - 4s 7ms/step - loss: 0.4810 - accuracy: 0.7
568 - val_loss: 0.6028 - val_accuracy: 0.7131
Epoch 85/200
579/579 [==============================] - 4s 7ms/step - loss: 0.4807 - accuracy: 0.7
568 - val_loss: 0.5899 - val_accuracy: 0.7110
Epoch 86/200
579/579 [==============================] - 4s 7ms/step - loss: 0.4799 - accuracy: 0.7
568 - val_loss: 0.6038 - val_accuracy: 0.7142
Epoch 87/200
579/579 [==============================] - 4s 7ms/step - loss: 0.4781 - accuracy: 0.7
591 - val_loss: 0.6090 - val_accuracy: 0.7117
Epoch 88/200
579/579 [==============================] - 4s 7ms/step - loss: 0.4787 - accuracy: 0.7
582 - val_loss: 0.6122 - val_accuracy: 0.7146
Epoch 89/200
579/579 [==============================] - 4s 7ms/step - loss: 0.4772 - accuracy: 0.7
596 - val_loss: 0.6151 - val_accuracy: 0.7107
Epoch 90/200
579/579 [==============================] - 3s 5ms/step - loss: 0.4764 - accuracy: 0.7
601 - val_loss: 0.6151 - val_accuracy: 0.7098
Epoch 91/200
579/579 [==============================] - 3s 6ms/step - loss: 0.4758 - accuracy: 0.7
611 - val_loss: 0.6169 - val_accuracy: 0.7054
Epoch 92/200
579/579 [==============================] - 3s 6ms/step - loss: 0.4756 - accuracy: 0.7
597 - val_loss: 0.6243 - val_accuracy: 0.7044
Epoch 93/200
579/579 [==============================] - 4s 7ms/step - loss: 0.4743 - accuracy: 0.7
612 - val_loss: 0.6448 - val_accuracy: 0.7068
Epoch 94/200
579/579 [==============================] - 4s 6ms/step - loss: 0.4729 - accuracy: 0.7
621 - val_loss: 0.6272 - val_accuracy: 0.7042
Epoch 95/200
579/579 [==============================] - 4s 6ms/step - loss: 0.4726 - accuracy: 0.7
625 - val_loss: 0.6350 - val_accuracy: 0.7020
Epoch 96/200
579/579 [==============================] - 4s 6ms/step - loss: 0.4717 - accuracy: 0.7
628 - val_loss: 0.6286 - val_accuracy: 0.6992
Epoch 97/200
579/579 [==============================] - 4s 6ms/step - loss: 0.4714 - accuracy: 0.7
626 - val_loss: 0.6563 - val_accuracy: 0.7138
Epoch 98/200
579/579 [==============================] - 4s 6ms/step - loss: 0.4703 - accuracy: 0.7
641 - val_loss: 0.6317 - val_accuracy: 0.7039
Epoch 99/200
579/579 [==============================] - 3s 6ms/step - loss: 0.4710 - accuracy: 0.7
636 - val_loss: 0.6417 - val_accuracy: 0.7103
Epoch 100/200
579/579 [==============================] - 3s 6ms/step - loss: 0.4706 - accuracy: 0.7
637 - val_loss: 0.6236 - val_accuracy: 0.7098
```

```
Epoch 101/200
579/579 [==============================] - 4s 7ms/step - loss: 0.4710 - accuracy: 0.7
624 - val_loss: 0.6273 - val_accuracy: 0.7090
Epoch 102/200
579/579 [==============================] - 3s 6ms/step - loss: 0.4702 - accuracy: 0.7
646 - val_loss: 0.6560 - val_accuracy: 0.7133
Epoch 103/200
579/579 [==============================] - 3s 6ms/step - loss: 0.4687 - accuracy: 0.7
635 - val_loss: 0.6627 - val_accuracy: 0.7190
Epoch 104/200
579/579 [==============================] - 3s 5ms/step - loss: 0.4682 - accuracy: 0.7
654 - val_loss: 0.6597 - val_accuracy: 0.7080
Epoch 105/200
579/579 [==============================] - 3s 5ms/step - loss: 0.4672 - accuracy: 0.7
651 - val_loss: 0.6202 - val_accuracy: 0.7092
Epoch 106/200
579/579 [==============================] - 3s 5ms/step - loss: 0.4666 - accuracy: 0.7
643 - val_loss: 0.6490 - val_accuracy: 0.7094
Epoch 107/200
579/579 [==============================] - 3s 5ms/step - loss: 0.4666 - accuracy: 0.7
672 - val_loss: 0.6584 - val_accuracy: 0.7139
Epoch 108/200
579/579 [==============================] - 3s 5ms/step - loss: 0.4663 - accuracy: 0.7
659 - val_loss: 0.6315 - val_accuracy: 0.6965
Epoch 109/200
579/579 [==============================] - 3s 5ms/step - loss: 0.4666 - accuracy: 0.7
657 - val_loss: 0.6074 - val_accuracy: 0.7104
Epoch 110/200
579/579 [==============================] - 3s 5ms/step - loss: 0.4658 - accuracy: 0.7
668 - val_loss: 0.6431 - val_accuracy: 0.7021
Epoch 111/200
579/579 [==============================] - 3s 5ms/step - loss: 0.4647 - accuracy: 0.7
666 - val_loss: 0.6671 - val_accuracy: 0.7052
Epoch 112/200
579/579 [==============================] - 3s 5ms/step - loss: 0.4630 - accuracy: 0.7
667 - val_loss: 0.6537 - val_accuracy: 0.7056
Epoch 113/200
579/579 [==============================] - 3s 6ms/step - loss: 0.4629 - accuracy: 0.7
684 - val_loss: 0.6313 - val_accuracy: 0.7153
Epoch 114/200
579/579 [==============================] - 3s 6ms/step - loss: 0.4639 - accuracy: 0.7
669 - val_loss: 0.6560 - val_accuracy: 0.7028
Epoch 115/200
579/579 [==============================] - 4s 6ms/step - loss: 0.4633 - accuracy: 0.7
674 - val_loss: 0.6228 - val_accuracy: 0.6964
Epoch 116/200
579/579 [==============================] - 3s 6ms/step - loss: 0.4620 - accuracy: 0.7
682 - val_loss: 0.6950 - val_accuracy: 0.6994
Epoch 117/200
579/579 [==============================] - 3s 6ms/step - loss: 0.4618 - accuracy: 0.7
675 - val_loss: 0.6582 - val_accuracy: 0.7035
Epoch 118/200
579/579 [==============================] - 5s 8ms/step - loss: 0.4616 - accuracy: 0.7
691 - val_loss: 0.6309 - val_accuracy: 0.7010
Epoch 119/200
579/579 [==============================] - 5s 9ms/step - loss: 0.4603 - accuracy: 0.7
674 - val_loss: 0.6370 - val_accuracy: 0.7082
Epoch 120/200
579/579 [==============================] - 5s 8ms/step - loss: 0.4601 - accuracy: 0.7
695 - val_loss: 0.6836 - val_accuracy: 0.7107
```

```
Epoch 121/200
579/579 [==============================] - 4s 6ms/step - loss: 0.4593 - accuracy: 0.7
697 - val_loss: 0.6322 - val_accuracy: 0.7085
Epoch 122/200
579/579 [==============================] - 3s 6ms/step - loss: 0.4579 - accuracy: 0.7
707 - val_loss: 0.6524 - val_accuracy: 0.6991
Epoch 123/200
579/579 [==============================] - 4s 7ms/step - loss: 0.4575 - accuracy: 0.7
701 - val_loss: 0.6564 - val_accuracy: 0.6963
Epoch 124/200
579/579 [==============================] - 4s 8ms/step - loss: 0.4567 - accuracy: 0.7
711 - val_loss: 0.6851 - val_accuracy: 0.7120
Epoch 125/200
579/579 [==============================] - 4s 7ms/step - loss: 0.4557 - accuracy: 0.7
706 - val_loss: 0.6661 - val_accuracy: 0.7041
Epoch 126/200
579/579 [==============================] - 4s 8ms/step - loss: 0.4555 - accuracy: 0.7
712 - val_loss: 0.6656 - val_accuracy: 0.7076
Epoch 127/200
579/579 [==============================] - 4s 7ms/step - loss: 0.4544 - accuracy: 0.7
728 - val_loss: 0.6590 - val_accuracy: 0.7021
Epoch 128/200
579/579 [==============================] - 4s 7ms/step - loss: 0.4540 - accuracy: 0.7
726 - val_loss: 0.6574 - val_accuracy: 0.7050
Epoch 129/200
579/579 [==============================] - 4s 7ms/step - loss: 0.4534 - accuracy: 0.7
727 - val_loss: 0.7317 - val_accuracy: 0.7005
Epoch 130/200
579/579 [==============================] - 4s 7ms/step - loss: 0.4537 - accuracy: 0.7
721 - val_loss: 0.6649 - val_accuracy: 0.6968
Epoch 131/200
579/579 [==============================] - 4s 6ms/step - loss: 0.4517 - accuracy: 0.7
741 - val_loss: 0.6515 - val_accuracy: 0.7003
Epoch 132/200
579/579 [==============================] - 3s 6ms/step - loss: 0.4506 - accuracy: 0.7
741 - val_loss: 0.6753 - val_accuracy: 0.6997
Epoch 133/200
579/579 [==============================] - 4s 6ms/step - loss: 0.4515 - accuracy: 0.7
730 - val_loss: 0.6429 - val_accuracy: 0.7033
Epoch 134/200
579/579 [==============================] - 3s 6ms/step - loss: 0.4504 - accuracy: 0.7
740 - val_loss: 0.6467 - val_accuracy: 0.6989
Epoch 135/200
579/579 [==============================] - 4s 6ms/step - loss: 0.4505 - accuracy: 0.7
737 - val_loss: 0.6944 - val_accuracy: 0.7074
Epoch 136/200
579/579 [==============================] - 3s 6ms/step - loss: 0.4496 - accuracy: 0.7
752 - val_loss: 0.7792 - val_accuracy: 0.7085
Epoch 137/200
579/579 [==============================] - 3s 6ms/step - loss: 0.4483 - accuracy: 0.7
767 - val_loss: 0.6859 - val_accuracy: 0.7048
Epoch 138/200
579/579 [==============================] - 3s 6ms/step - loss: 0.4501 - accuracy: 0.7
754 - val_loss: 0.6594 - val_accuracy: 0.7080
Epoch 139/200
579/579 [==============================] - 3s 6ms/step - loss: 0.4483 - accuracy: 0.7
747 - val_loss: 0.6928 - val_accuracy: 0.7083
Epoch 140/200
579/579 [==============================] - 3s 5ms/step - loss: 0.4486 - accuracy: 0.7
760 - val_loss: 0.7324 - val_accuracy: 0.7067
```

```
Epoch 141/200
579/579 [==============================] - 3s 5ms/step - loss: 0.4475 - accuracy: 0.7
761 - val_loss: 0.6885 - val_accuracy: 0.7000
Epoch 142/200
579/579 [==============================] - 3s 5ms/step - loss: 0.4450 - accuracy: 0.7
766 - val_loss: 0.7711 - val_accuracy: 0.7081
Epoch 143/200
579/579 [==============================] - 3s 5ms/step - loss: 0.4458 - accuracy: 0.7
775 - val_loss: 0.6610 - val_accuracy: 0.6965
Epoch 144/200
579/579 [==============================] - 3s 6ms/step - loss: 0.4453 - accuracy: 0.7
786 - val_loss: 0.6678 - val_accuracy: 0.7040
Epoch 145/200
579/579 [==============================] - 3s 6ms/step - loss: 0.4450 - accuracy: 0.7
783 - val_loss: 0.7305 - val_accuracy: 0.7068
Epoch 146/200
579/579 [==============================] - 3s 6ms/step - loss: 0.4470 - accuracy: 0.7
773 - val_loss: 0.6874 - val_accuracy: 0.6820
Epoch 147/200
579/579 [==============================] - 4s 7ms/step - loss: 0.4447 - accuracy: 0.7
777 - val_loss: 0.6886 - val_accuracy: 0.7070
Epoch 148/200
579/579 [==============================] - 3s 6ms/step - loss: 0.4443 - accuracy: 0.7
779 - val_loss: 0.6406 - val_accuracy: 0.7061
Epoch 149/200
579/579 [==============================] - 3s 6ms/step - loss: 0.4454 - accuracy: 0.7
768 - val_loss: 0.7285 - val_accuracy: 0.7077
Epoch 150/200
579/579 [==============================] - 3s 6ms/step - loss: 0.4435 - accuracy: 0.7
790 - val_loss: 0.6695 - val_accuracy: 0.6997
Epoch 151/200
579/579 [==============================] - 3s 6ms/step - loss: 0.4420 - accuracy: 0.7
802 - val_loss: 0.6997 - val_accuracy: 0.6988
Epoch 152/200
579/579 [==============================] - 3s 5ms/step - loss: 0.4433 - accuracy: 0.7
781 - val_loss: 0.6990 - val_accuracy: 0.6981
Epoch 153/200
579/579 [==============================] - 2s 4ms/step - loss: 0.4428 - accuracy: 0.7
777 - val_loss: 0.7119 - val_accuracy: 0.6904
Epoch 154/200
579/579 [==============================] - 3s 5ms/step - loss: 0.4431 - accuracy: 0.7
798 - val_loss: 0.6931 - val_accuracy: 0.7014
Epoch 155/200
579/579 [==============================] - 3s 5ms/step - loss: 0.4441 - accuracy: 0.7
774 - val_loss: 0.7148 - val_accuracy: 0.6938
Epoch 156/200
579/579 [==============================] - 3s 5ms/step - loss: 0.4422 - accuracy: 0.7
799 - val_loss: 0.6993 - val_accuracy: 0.6985
Epoch 157/200
579/579 [==============================] - 3s 5ms/step - loss: 0.4418 - accuracy: 0.7
791 - val_loss: 0.6773 - val_accuracy: 0.6960
Epoch 158/200
579/579 [==============================] - 3s 5ms/step - loss: 0.4406 - accuracy: 0.7
806 - val_loss: 0.7440 - val_accuracy: 0.6989
Epoch 159/200
579/579 [==============================] - 3s 5ms/step - loss: 0.4391 - accuracy: 0.7
809 - val_loss: 0.7044 - val_accuracy: 0.7043
Epoch 160/200
579/579 [==============================] - 3s 5ms/step - loss: 0.4394 - accuracy: 0.7
796 - val_loss: 0.6926 - val_accuracy: 0.7025
```

```
Epoch 161/200
579/579 [==============================] - 3s 5ms/step - loss: 0.4386 - accuracy: 0.7
809 - val_loss: 0.7065 - val_accuracy: 0.7061
Epoch 162/200
579/579 [==============================] - 3s 5ms/step - loss: 0.4379 - accuracy: 0.7
813 - val_loss: 0.7387 - val_accuracy: 0.6911
Epoch 163/200
579/579 [==============================] - 3s 5ms/step - loss: 0.4371 - accuracy: 0.7
819 - val_loss: 0.6952 - val_accuracy: 0.6948
Epoch 164/200
579/579 [==============================] - 3s 5ms/step - loss: 0.4378 - accuracy: 0.7
820 - val_loss: 0.6675 - val_accuracy: 0.6939
Epoch 165/200
579/579 [==============================] - 3s 5ms/step - loss: 0.4361 - accuracy: 0.7
827 - val_loss: 0.7118 - val_accuracy: 0.6840
Epoch 166/200
579/579 [==============================] - 3s 5ms/step - loss: 0.4372 - accuracy: 0.7
815 - val_loss: 0.6920 - val_accuracy: 0.7006
Epoch 167/200
579/579 [==============================] - 3s 6ms/step - loss: 0.4367 - accuracy: 0.7
823 - val_loss: 0.7364 - val_accuracy: 0.6901
Epoch 168/200
579/579 [==============================] - 3s 6ms/step - loss: 0.4346 - accuracy: 0.7
829 - val_loss: 0.7485 - val_accuracy: 0.6950
Epoch 169/200
579/579 [==============================] - 3s 6ms/step - loss: 0.4335 - accuracy: 0.7
838 - val_loss: 0.6986 - val_accuracy: 0.6948
Epoch 170/200
579/579 [==============================] - 3s 6ms/step - loss: 0.4332 - accuracy: 0.7
829 - val_loss: 0.7427 - val_accuracy: 0.7018
Epoch 171/200
579/579 [==============================] - 3s 5ms/step - loss: 0.4336 - accuracy: 0.7
847 - val_loss: 0.7914 - val_accuracy: 0.7034
Epoch 172/200
579/579 [==============================] - 3s 5ms/step - loss: 0.4307 - accuracy: 0.7
851 - val_loss: 0.7432 - val_accuracy: 0.6968
Epoch 173/200
579/579 [==============================] - 3s 5ms/step - loss: 0.4318 - accuracy: 0.7
829 - val_loss: 0.7205 - val_accuracy: 0.6900
Epoch 174/200
579/579 [==============================] - 4s 6ms/step - loss: 0.4303 - accuracy: 0.7
854 - val_loss: 0.7031 - val_accuracy: 0.6949
Epoch 175/200
579/579 [==============================] - 4s 6ms/step - loss: 0.4306 - accuracy: 0.7
836 - val_loss: 0.6959 - val_accuracy: 0.7010
Epoch 176/200
579/579 [==============================] - 3s 5ms/step - loss: 0.4303 - accuracy: 0.7
851 - val_loss: 0.6896 - val_accuracy: 0.6926
Epoch 177/200
579/579 [==============================] - 3s 4ms/step - loss: 0.4301 - accuracy: 0.7
839 - val_loss: 0.7258 - val_accuracy: 0.6916
Epoch 178/200
579/579 [==============================] - 2s 4ms/step - loss: 0.4283 - accuracy: 0.7
867 - val_loss: 0.7337 - val_accuracy: 0.7050
Epoch 179/200
579/579 [==============================] - 3s 5ms/step - loss: 0.4289 - accuracy: 0.7
864 - val_loss: 0.7457 - val_accuracy: 0.7040
Epoch 180/200
579/579 [==============================] - 3s 5ms/step - loss: 0.4290 - accuracy: 0.7
867 - val_loss: 0.7298 - val_accuracy: 0.6898
```

```
Epoch 181/200
579/579 [==============================] - 3s 5ms/step - loss: 0.4294 - accuracy: 0.7
861 - val_loss: 0.8044 - val_accuracy: 0.6928
Epoch 182/200
579/579 [==============================] - 3s 5ms/step - loss: 0.4284 - accuracy: 0.7
856 - val_loss: 0.7137 - val_accuracy: 0.7011
Epoch 183/200
579/579 [==============================] - 3s 4ms/step - loss: 0.4267 - accuracy: 0.7
868 - val_loss: 0.7158 - val_accuracy: 0.6935
Epoch 184/200
579/579 [==============================] - 3s 5ms/step - loss: 0.4279 - accuracy: 0.7
863 - val_loss: 0.7580 - val_accuracy: 0.7070
Epoch 185/200
579/579 [==============================] - 3s 5ms/step - loss: 0.4259 - accuracy: 0.7
874 - val_loss: 0.7220 - val_accuracy: 0.6950
Epoch 186/200
579/579 [==============================] - 3s 5ms/step - loss: 0.4249 - accuracy: 0.7
878 - val_loss: 0.7180 - val_accuracy: 0.6970
Epoch 187/200
579/579 [==============================] - 3s 5ms/step - loss: 0.4247 - accuracy: 0.7
884 - val_loss: 0.7595 - val_accuracy: 0.7005
Epoch 188/200
579/579 [==============================] - 3s 5ms/step - loss: 0.4250 - accuracy: 0.7
878 - val_loss: 0.7158 - val_accuracy: 0.6848
Epoch 189/200
579/579 [==============================] - 2s 4ms/step - loss: 0.4237 - accuracy: 0.7
887 - val_loss: 0.7332 - val_accuracy: 0.6999
Epoch 190/200
579/579 [==============================] - 3s 5ms/step - loss: 0.4236 - accuracy: 0.7
888 - val_loss: 0.7292 - val_accuracy: 0.6910
Epoch 191/200
579/579 [==============================] - 3s 5ms/step - loss: 0.4247 - accuracy: 0.7
878 - val_loss: 0.7401 - val_accuracy: 0.7058
Epoch 192/200
579/579 [==============================] - 3s 5ms/step - loss: 0.4234 - accuracy: 0.7
879 - val_loss: 0.7302 - val_accuracy: 0.7006
Epoch 193/200
579/579 [==============================] - 3s 5ms/step - loss: 0.4224 - accuracy: 0.7
892 - val_loss: 0.8058 - val_accuracy: 0.6969
Epoch 194/200
579/579 [==============================] - 2s 4ms/step - loss: 0.4190 - accuracy: 0.7
913 - val_loss: 0.7475 - val_accuracy: 0.6944
Epoch 195/200
579/579 [==============================] - 3s 5ms/step - loss: 0.4216 - accuracy: 0.7
899 - val_loss: 0.7514 - val_accuracy: 0.6865
Epoch 196/200
579/579 [==============================] - 3s 5ms/step - loss: 0.4211 - accuracy: 0.7
894 - val_loss: 0.7493 - val_accuracy: 0.6817
Epoch 197/200
579/579 [==============================] - 4s 6ms/step - loss: 0.4208 - accuracy: 0.7
900 - val_loss: 0.7081 - val_accuracy: 0.6975
Epoch 198/200
579/579 [==============================] - 4s 7ms/step - loss: 0.4234 - accuracy: 0.7
888 - val_loss: 0.7668 - val_accuracy: 0.6917
Epoch 199/200
579/579 [==============================] - 3s 5ms/step - loss: 0.4210 - accuracy: 0.7
911 - val_loss: 0.7564 - val_accuracy: 0.6935
Epoch 200/200
579/579 [==============================] - 3s 6ms/step - loss: 0.4203 - accuracy: 0.7
882 - val_loss: 0.7720 - val_accuracy: 0.6974
```

In [72]:
```
# Concatenate Train and Validation Data
X_training = np.concatenate((X_train, X_val), axis=0)
y_training = np.concatenate((y_train, y_val), axis=0)
```

In [76]:
```
# Re-train network on complete Training Data, with Epochs set at 25
network.fit(X_training, y_training,
            batch_size=128, epochs=40)
```

```
Epoch 1/40
771/771 [==============================] - 5s 5ms/step - loss: 0.5785 - accuracy: 0.6
931
Epoch 2/40
771/771 [==============================] - 4s 5ms/step - loss: 0.5639 - accuracy: 0.7
073
Epoch 3/40
771/771 [==============================] - 4s 6ms/step - loss: 0.5601 - accuracy: 0.7
107
Epoch 4/40
771/771 [==============================] - 4s 5ms/step - loss: 0.5574 - accuracy: 0.7
150
Epoch 5/40
771/771 [==============================] - 4s 5ms/step - loss: 0.5552 - accuracy: 0.7
163
Epoch 6/40
771/771 [==============================] - 4s 5ms/step - loss: 0.5539 - accuracy: 0.7
175
Epoch 7/40
771/771 [==============================] - 4s 6ms/step - loss: 0.5525 - accuracy: 0.7
184
Epoch 8/40
771/771 [==============================] - 3s 4ms/step - loss: 0.5513 - accuracy: 0.7
186
Epoch 9/40
771/771 [==============================] - 3s 4ms/step - loss: 0.5499 - accuracy: 0.7
199
Epoch 10/40
771/771 [==============================] - 4s 5ms/step - loss: 0.5492 - accuracy: 0.7
197
Epoch 11/40
771/771 [==============================] - 4s 5ms/step - loss: 0.5475 - accuracy: 0.7
207
Epoch 12/40
771/771 [==============================] - 4s 5ms/step - loss: 0.5472 - accuracy: 0.7
200
Epoch 13/40
771/771 [==============================] - 4s 5ms/step - loss: 0.5461 - accuracy: 0.7
212
Epoch 14/40
771/771 [==============================] - 4s 6ms/step - loss: 0.5451 - accuracy: 0.7
217
Epoch 15/40
771/771 [==============================] - 4s 5ms/step - loss: 0.5443 - accuracy: 0.7
217
Epoch 16/40
771/771 [==============================] - 4s 5ms/step - loss: 0.5434 - accuracy: 0.7
226
Epoch 17/40
771/771 [==============================] - 4s 5ms/step - loss: 0.5425 - accuracy: 0.7
221
Epoch 18/40
771/771 [==============================] - 4s 5ms/step - loss: 0.5420 - accuracy: 0.7
223
Epoch 19/40
771/771 [==============================] - 4s 5ms/step - loss: 0.5413 - accuracy: 0.7
222
Epoch 20/40
771/771 [==============================] - 4s 5ms/step - loss: 0.5402 - accuracy: 0.7
232
```

```
Epoch 21/40
771/771 [==============================] - 3s 4ms/step - loss: 0.5396 - accuracy: 0.7
239
Epoch 22/40
771/771 [==============================] - 3s 4ms/step - loss: 0.5388 - accuracy: 0.7
236
Epoch 23/40
771/771 [==============================] - 3s 4ms/step - loss: 0.5379 - accuracy: 0.7
243
Epoch 24/40
771/771 [==============================] - 3s 4ms/step - loss: 0.5372 - accuracy: 0.7
253
Epoch 25/40
771/771 [==============================] - 3s 4ms/step - loss: 0.5371 - accuracy: 0.7
238
Epoch 26/40
771/771 [==============================] - 3s 4ms/step - loss: 0.5362 - accuracy: 0.7
247
Epoch 27/40
771/771 [==============================] - 3s 4ms/step - loss: 0.5355 - accuracy: 0.7
263
Epoch 28/40
771/771 [==============================] - 3s 4ms/step - loss: 0.5348 - accuracy: 0.7
265
Epoch 29/40
771/771 [==============================] - 4s 5ms/step - loss: 0.5341 - accuracy: 0.7
263
Epoch 30/40
771/771 [==============================] - 4s 5ms/step - loss: 0.5335 - accuracy: 0.7
264
Epoch 31/40
771/771 [==============================] - 3s 4ms/step - loss: 0.5334 - accuracy: 0.7
269
Epoch 32/40
771/771 [==============================] - 3s 4ms/step - loss: 0.5322 - accuracy: 0.7
273
Epoch 33/40
771/771 [==============================] - 4s 5ms/step - loss: 0.5319 - accuracy: 0.7
276
Epoch 34/40
771/771 [==============================] - 3s 4ms/step - loss: 0.5309 - accuracy: 0.7
278
Epoch 35/40
771/771 [==============================] - 4s 5ms/step - loss: 0.5308 - accuracy: 0.7
276
Epoch 36/40
771/771 [==============================] - 4s 5ms/step - loss: 0.5302 - accuracy: 0.7
295
Epoch 37/40
771/771 [==============================] - 4s 5ms/step - loss: 0.5295 - accuracy: 0.7
298
Epoch 38/40
771/771 [==============================] - 4s 5ms/step - loss: 0.5284 - accuracy: 0.7
299
Epoch 39/40
771/771 [==============================] - 4s 5ms/step - loss: 0.5281 - accuracy: 0.7
298
Epoch 40/40
771/771 [==============================] - 4s 5ms/step - loss: 0.5273 - accuracy: 0.7
307
```

Out[76]:
```
<keras.src.callbacks.History at 0x2323dfca950>
```

In [77]:
```
y_prediction = network.predict(X_test)
```

```
771/771 [==============================] - 2s 2ms/step
```

In [78]:
```
network.evaluate(X_test, y_test)
```

```
771/771 [==============================] - 2s 2ms/step - loss: 0.5475 - accuracy: 0.7
194
```

Out[78]:
```
[0.547500729560852, 0.7193984985351562]
```

In [79]:
```
y_prediction[y_prediction >= 0.5] = 1
y_prediction[y_prediction < 0.5] = 0
```

In [80]:
```
nnDF= pd.DataFrame(y_prediction)
```

In [81]:
```
nnDF.value_counts()
```

Out[81]:
```
1.0    20415
0.0     4257
Name: count, dtype: int64
```

In [82]:
```
plotCM(y_test, y_prediction)
```

```
accuracy Score is: 0.7193985084306096
```

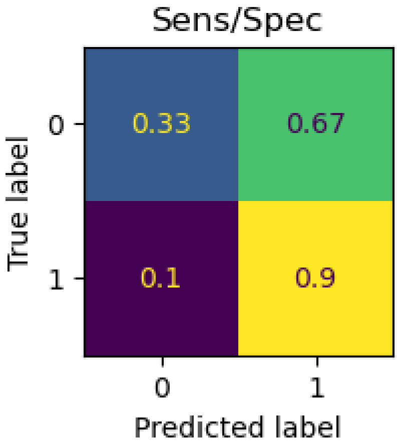

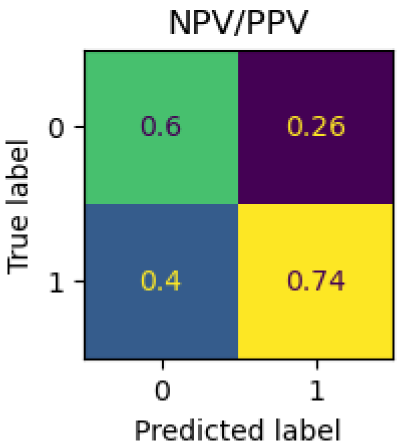

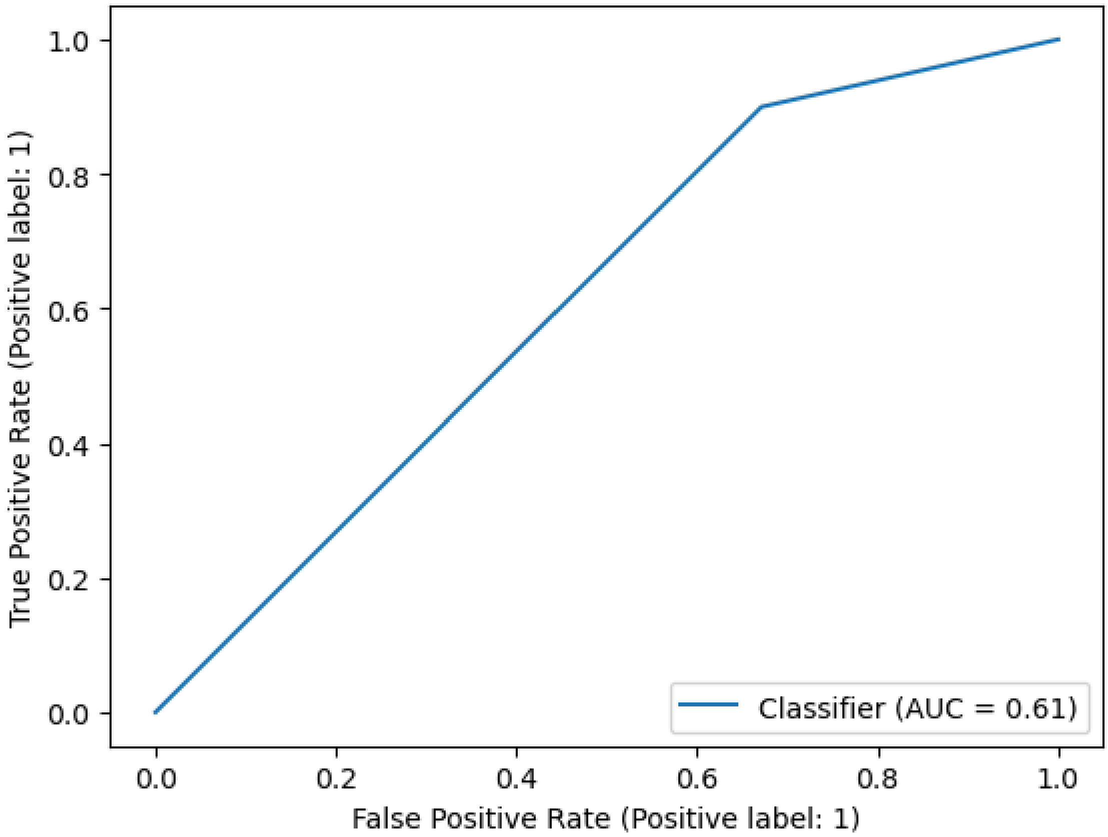
True Positive Rate (Positive label: 1)
False Positive Rate (Positive label: 1)
Classifier (AUC = 0.61)
0.0
0.2
0.4
0.6
0.8
1.0

In [ ]:

Reimport files and re-process for 36 hour threshold modeling

In [38]:
```
df5 = pd.read_csv('/Users/ganatrh/Downloads/DataWithPrimaryDiagnosis2.csv')
df70 = pd.read_csv('/Users/ganatrh/Downloads/PIM.csv')
```

In [39]:
```
df5 = df5.merge(df70, on='Case Index Id', how='left' )
```

In [40]:
```
df5.info()
```

```
<class 'pandas.core.frame.DataFrame'>
RangeIndex: 608512 entries, 0 to 608511
Data columns (total 78 columns):
 #   Column                                          Non-Null Count   Dtype  
---  ------                                          --------------   -----  
 0   Unit Id                                         608512 non-null  int64  
 1   Case Id                                         608512 non-null  int64  
 2   Case Index Id                                   608512 non-null  object 
 3   Discharge Year                                  608512 non-null  int64  
 4   Is Readmission                                  608512 non-null  int64  
 5   Age                                             608512 non-null  object 
 6   Weight (kg)                                     608512 non-null  float64
 7   Flag - Age/Weight                               608512 non-null  int64  
 8   Collects height                                 608512 non-null  int64  
 9   Height (cm)                                     309701 non-null  float64
 10  Gender                                          608512 non-null  object 
 11  Collects Race                                   608512 non-null  int64  
 12  Race                                            550714 non-null  object 
 13  Patient Origin                                  608512 non-null  object 
 14  Trauma                                          608512 non-null  int64  
 15  Patient Type                                    608512 non-null  object 
 16  Collects Transport Team                         608512 non-null  int64  
 17  Transport Team                                  371934 non-null  object 
 18  Collects Transport Vehicle                      608512 non-null  int64  
 19  Transport Vehicle                               347485 non-null  object 
 20  Post Operative                                  608512 non-null  int64  
 21  Collects Baseline PCPC/POPC                     608512 non-null  int64  
 22  Baseline PCPC                                   127543 non-null  object 
 23  Baseline POPC                                   127464 non-null  object 
 24  Collects FSS                                    608512 non-null  int64  
 25  Baseline FSS                                    59273 non-null   float64
 26  Cardiac Patient                                 608512 non-null  int64  
 27  Cardiac Procedure directly prior to or during stay  608512 non-null  int64  
 28  Medical Length of Stay (days)                   584327 non-null  float64
 29  Physical Length of Stay (days)                  608512 non-null  float64
 30  Hospital LOS                                    606176 non-null  float64
 31  Outcome                                         608512 non-null  object 
 32  Disposition                                     608512 non-null  object 
 33  Collects Limitation on Care                     608512 non-null  int64  
 34  Limitation on Care                              523209 non-null  float64
 35  Collects Brain Dead                             608512 non-null  int64  
 36  Is Brain Dead                                   4922 non-null    float64
 37  Collects Altered Code                           608512 non-null  int64  
 38  Is Altered Code                                 523227 non-null  float64
 39  Collects Withdrawal of Support                  608512 non-null  int64  
 40  Withdrawal of Support                           497357 non-null  float64
 41  Collects Autopsy                                608512 non-null  int64  
 42  Was Autopsy Performed                           11602 non-null   object 
 43  Collects Organ Donation                         608512 non-null  int64  
 44  Is Organ Donor                                  2572 non-null    float64
 45  Collects Tissue Donation                        608512 non-null  int64  
 46  Is Tissue Donor                                 3443 non-null    float64
 47  Collects Donation after Cardiac Death           608512 non-null  int64  
 48  Donation after Cardiac Death                    3563 non-null    float64
 49  Discharge PCPC                                  123029 non-null  object 
 50  Discharge POPC                                  122965 non-null  object 
 51  Discharge FSS                                   58085 non-null   float64
 52  Hospital Outcome                                604949 non-null  object 
 53  Collects Diagnosis STS Codes                    608512 non-null  int64  
 54  Collects Diagnosis ICD-9 Codes                  608512 non-null  int64  
```

```
 55  PIM 3 Score                                           608512 non-null  float64
 56  PIM 3 Probability of Death                            608512 non-null  float64
 57  Mechanical Ventilation (First Hour)                   608511 non-null  float64
 58  Elective Admission to ICU                             608511 non-null  float64
 59  PIM 3 Recovery from Surgery                           608511 non-null  object
 60  Category                                              608475 non-null  object
 61  Systolic Blood Pressure                               588367 non-null  float64
 62  Systolic Blood Pressure Unknown                       608511 non-null  float64
 63  PaO2 (mmHg)                                           13722 non-null   float64
 64  PaO2 (kPa)                                            13722 non-null   float64
 65  PaO2 Unknown                                          608511 non-null  float64
 66  FiO2                                                  13722 non-null   float64
 67  FiO2 Unknown                                          608511 non-null  float64
 68  Base Excess                                           51063 non-null   float64
 69  Base Excess Unknown                                   608511 non-null  float64
 70  PIM 3 Pupillary Reaction                              608511 non-null  object
 71  PIM 3 Pupillary Reaction Unknown                      608511 non-null  float64
 72  Admitted following Cardiac Bypass - PIM2/PIM3         608511 non-null  float64
 73  Admitted following Cardiac Bypass - PIM3 Baseline     608511 non-null  float64
 74  Admitted following Cardiac Bypass - PIM3 PostIndex    166 non-null     float64
 75  PIM 3 - No Very High Risk Dx                          608512 non-null  int64
 76  PIM 3 - No High Risk Dx                               608512 non-null  int64
 77  PIM 3 - No Low Risk Dx                                608512 non-null  int64
dtypes: float64(31), int64(28), object(19)
memory usage: 362.1+ MB
```

Remove rows that have flagged inaccurate weights

In [41]:

```
df6 = SmartDataframe(df5, config={"llm": llm})
df7 = df6.chat("Remove rows that have the value '1' in column 'Flag - Age/Weight' and
df7.info()
```

```
<class 'pandas.core.frame.DataFrame'>
RangeIndex: 607901 entries, 0 to 607900
Data columns (total 78 columns):
 #   Column                                          Non-Null Count   Dtype  
---  ------                                          --------------   -----  
 0   Unit Id                                         607901 non-null  int64  
 1   Case Id                                         607901 non-null  int64  
 2   Case Index Id                                   607901 non-null  object 
 3   Discharge Year                                  607901 non-null  int64  
 4   Is Readmission                                  607901 non-null  int64  
 5   Age                                             607901 non-null  object 
 6   Weight (kg)                                     607901 non-null  float64
 7   Flag - Age/Weight                               607901 non-null  int64  
 8   Collects height                                 607901 non-null  int64  
 9   Height (cm)                                     309407 non-null  float64
 10  Gender                                          607901 non-null  object 
 11  Collects Race                                   607901 non-null  int64  
 12  Race                                            550155 non-null  object 
 13  Patient Origin                                  607901 non-null  object 
 14  Trauma                                          607901 non-null  int64  
 15  Patient Type                                    607901 non-null  object 
 16  Collects Transport Team                         607901 non-null  int64  
 17  Transport Team                                  371523 non-null  object 
 18  Collects Transport Vehicle                      607901 non-null  int64  
 19  Transport Vehicle                               347101 non-null  object 
 20  Post Operative                                  607901 non-null  int64  
 21  Collects Baseline PCPC/POPC                     607901 non-null  int64  
 22  Baseline PCPC                                   127387 non-null  object 
 23  Baseline POPC                                   127309 non-null  object 
 24  Collects FSS                                    607901 non-null  int64  
 25  Baseline FSS                                    59184 non-null   float64
 26  Cardiac Patient                                 607901 non-null  int64  
 27  Cardiac Procedure directly prior to or during stay  607901 non-null  int64  
 28  Medical Length of Stay (days)                   583736 non-null  float64
 29  Physical Length of Stay (days)                  607901 non-null  float64
 30  Hospital LOS                                    605567 non-null  float64
 31  Outcome                                         607901 non-null  object 
 32  Disposition                                     607901 non-null  object 
 33  Collects Limitation on Care                     607901 non-null  int64  
 34  Limitation on Care                              522675 non-null  float64
 35  Collects Brain Dead                             607901 non-null  int64  
 36  Is Brain Dead                                   4918 non-null    float64
 37  Collects Altered Code                           607901 non-null  int64  
 38  Is Altered Code                                 522693 non-null  float64
 39  Collects Withdrawal of Support                  607901 non-null  int64  
 40  Withdrawal of Support                           496859 non-null  float64
 41  Collects Autopsy                                607901 non-null  int64  
 42  Was Autopsy Performed                           11595 non-null   object 
 43  Collects Organ Donation                         607901 non-null  int64  
 44  Is Organ Donor                                  2572 non-null    float64
 45  Collects Tissue Donation                        607901 non-null  int64  
 46  Is Tissue Donor                                 3442 non-null    float64
 47  Collects Donation after Cardiac Death           607901 non-null  int64  
 48  Donation after Cardiac Death                    3562 non-null    float64
 49  Discharge PCPC                                  122879 non-null  object 
 50  Discharge POPC                                  122815 non-null  object 
 51  Discharge FSS                                   58000 non-null   float64
 52  Hospital Outcome                                604344 non-null  object 
 53  Collects Diagnosis STS Codes                    607901 non-null  int64  
 54  Collects Diagnosis ICD-9 Codes                  607901 non-null  int64  
```

```
 55  PIM 3 Score                                            607901 non-null  float64
 56  PIM 3 Probability of Death                             607901 non-null  float64
 57  Mechanical Ventilation (First Hour)                    607900 non-null  float64
 58  Elective Admission to ICU                              607900 non-null  float64
 59  PIM 3 Recovery from Surgery                            607900 non-null  object
 60  Category                                               607864 non-null  object
 61  Systolic Blood Pressure                                587779 non-null  float64
 62  Systolic Blood Pressure Unknown                        607900 non-null  float64
 63  PaO2 (mmHg)                                            13708 non-null   float64
 64  PaO2 (kPa)                                             13708 non-null   float64
 65  PaO2 Unknown                                           607900 non-null  float64
 66  FiO2                                                   13708 non-null   float64
 67  FiO2 Unknown                                           607900 non-null  float64
 68  Base Excess                                            50991 non-null   float64
 69  Base Excess Unknown                                    607900 non-null  float64
 70  PIM 3 Pupillary Reaction                               607900 non-null  object
 71  PIM 3 Pupillary Reaction Unknown                       607900 non-null  float64
 72  Admitted following Cardiac Bypass - PIM2/PIM3          607900 non-null  float64
 73  Admitted following Cardiac Bypass - PIM3 Baseline      607900 non-null  float64
 74  Admitted following Cardiac Bypass - PIM3 PostIndex     166 non-null     float64
 75  PIM 3 - No Very High Risk Dx                           607901 non-null  int64
 76  PIM 3 - No High Risk Dx                                607901 non-null  int64
 77  PIM 3 - No Low Risk Dx                                 607901 non-null  int64
dtypes: float64(31), int64(28), object(19)
memory usage: 361.8+ MB
```

Create dataframe with Columns of interest

In [42]:
```python
df8 = SmartDataframe(df7, config={"llm": llm})
df9 = df8.chat("Select the columns 'Case Index Id', 'Is Readmission', 'Age', 'Weight (
```

In [43]:
```python
df9.info()
```

```
<class 'pandas.core.frame.DataFrame'>
RangeIndex: 607901 entries, 0 to 607900
Data columns (total 41 columns):
 #   Column                                               Non-Null Count   Dtype  
---  ------                                               --------------   -----  
 0   Case Index Id                                        607901 non-null  object 
 1   Is Readmission                                       607901 non-null  int64  
 2   Age                                                  607901 non-null  object 
 3   Weight (kg)                                          607901 non-null  float64
 4   Gender                                               607901 non-null  object 
 5   Race                                                 550155 non-null  object 
 6   Patient Origin                                       607901 non-null  object 
 7   Trauma                                               607901 non-null  int64  
 8   Patient Type                                         607901 non-null  object 
 9   Transport Team                                       371523 non-null  object 
 10  Transport Vehicle                                    347101 non-null  object 
 11  Post Operative                                       607901 non-null  int64  
 12  Baseline PCPC                                        127387 non-null  object 
 13  Baseline POPC                                        127309 non-null  object 
 14  Baseline FSS                                         59184 non-null   float64
 15  Cardiac Patient                                      607901 non-null  int64  
 16  Cardiac Procedure directly prior to or during stay  607901 non-null  int64  
 17  Medical Length of Stay (days)                        583736 non-null  float64
 18  Physical Length of Stay (days)                       607901 non-null  float64
 19  Hospital LOS                                         605567 non-null  float64
 20  Outcome                                              607901 non-null  object 
 21  Disposition                                          607901 non-null  object 
 22  Is Altered Code                                      522693 non-null  float64
 23  Discharge PCPC                                       122879 non-null  object 
 24  Discharge POPC                                       122815 non-null  object 
 25  Discharge FSS                                        58000 non-null   float64
 26  Hospital Outcome                                     604344 non-null  object 
 27  PIM 3 Score                                          607901 non-null  float64
 28  PIM 3 Probability of Death                           607901 non-null  float64
 29  Mechanical Ventilation (First Hour)                  607900 non-null  float64
 30  Elective Admission to ICU                            607900 non-null  float64
 31  PIM 3 Recovery from Surgery                          607900 non-null  object 
 32  Systolic Blood Pressure                              587779 non-null  float64
 33  PaO2 (mmHg)                                          13708 non-null   float64
 34  FiO2                                                 13708 non-null   float64
 35  Base Excess                                          50991 non-null   float64
 36  PIM 3 Pupillary Reaction                             607900 non-null  object 
 37  PIM 3 - No Very High Risk Dx                         607901 non-null  int64  
 38  PIM 3 - No High Risk Dx                              607901 non-null  int64  
 39  PIM 3 - No Low Risk Dx                               607901 non-null  int64  
 40  Category                                             607864 non-null  object 
dtypes: float64(15), int64(8), object(18)
memory usage: 190.2+ MB
```

Remove rows that have NaN for "elective ICU admission" and for "POPC/PCPC". This comcomitantly removes NaN for other variables too

In [116…

```
df9.dropna(subset=['Elective Admission to ICU', 'Baseline POPC', 'Baseline PCPC', 'Sys
df9.info()
```

```
<class 'pandas.core.frame.DataFrame'>
RangeIndex: 123485 entries, 0 to 123484
Data columns (total 41 columns):
 #   Column                                                Non-Null Count   Dtype  
---  ------                                                --------------   -----  
 0   Case Index Id                                         123485 non-null  object 
 1   Is Readmission                                        123485 non-null  int64  
 2   Age                                                   123485 non-null  object 
 3   Weight (kg)                                           123485 non-null  float64
 4   Gender                                                123485 non-null  object 
 5   Race                                                  121785 non-null  object 
 6   Patient Origin                                        123485 non-null  object 
 7   Trauma                                                123485 non-null  int64  
 8   Patient Type                                          123485 non-null  object 
 9   Transport Team                                        98498 non-null   object 
 10  Transport Vehicle                                     104581 non-null  object 
 11  Post Operative                                        123485 non-null  int64  
 12  Baseline PCPC                                         123485 non-null  object 
 13  Baseline POPC                                         123485 non-null  object 
 14  Baseline FSS                                          16690 non-null   float64
 15  Cardiac Patient                                       123485 non-null  int64  
 16  Cardiac Procedure directly prior to or during stay    123485 non-null  int64  
 17  Medical Length of Stay (days)                         118321 non-null  float64
 18  Physical Length of Stay (days)                        123485 non-null  float64
 19  Hospital LOS                                          123245 non-null  float64
 20  Outcome                                               123485 non-null  object 
 21  Disposition                                           123485 non-null  object 
 22  Is Altered Code                                       122991 non-null  float64
 23  Discharge PCPC                                        118721 non-null  object 
 24  Discharge POPC                                        118695 non-null  object 
 25  Discharge FSS                                         16383 non-null   float64
 26  Hospital Outcome                                      122734 non-null  object 
 27  PIM 3 Score                                           123485 non-null  float64
 28  PIM 3 Probability of Death                            123485 non-null  float64
 29  Mechanical Ventilation (First Hour)                   123485 non-null  float64
 30  Elective Admission to ICU                             123485 non-null  float64
 31  PIM 3 Recovery from Surgery                           123485 non-null  object 
 32  Systolic Blood Pressure                               123485 non-null  float64
 33  PaO2 (mmHg)                                           3205 non-null    float64
 34  FiO2                                                  3205 non-null    float64
 35  Base Excess                                           123485 non-null  float64
 36  PIM 3 Pupillary Reaction                              123485 non-null  object 
 37  PIM 3 - No Very High Risk Dx                          123485 non-null  int64  
 38  PIM 3 - No High Risk Dx                               123485 non-null  int64  
 39  PIM 3 - No Low Risk Dx                                123485 non-null  int64  
 40  Category                                              123485 non-null  object 
dtypes: float64(15), int64(8), object(18)
memory usage: 38.6+ MB
```

In [117…

```
df9["Base Excess"] = df9["Base Excess"].replace(np.nan, 0)
df9["Base Excess"].value_counts()
```

Out[117]:
```
Base Excess
 0.0     111514
-3.0        358
-4.0        342
-5.0        329
-2.0        327
          ...
-16.5         1
 9.7          1
 23.3         1
-34.1         1
 11.3         1
Name: count, Length: 472, dtype: int64
```

The cells below consolidate all patients > 18 into one group

In [118…
```
df9["Age"].value_counts()
```

Out[118]:
```
Age
Infant 29 days to < 2 years                  36705
Adolescent 12 years to < 18 years            32266
Child 2 years to < 6 years                   23946
Child 6 years to < 12 years                  22954
Adolescent (late) 18 years to < 21 years      4241
Neonate Birth to 29 days                      2355
Adult 21 years and up                         1018
Name: count, dtype: int64
```

In [119…
```
df10 = df9.copy()
df10.Age.replace(['Adolescent 12 years to < 18 years', 'Adult 21 years and up','Adoles
df10.Age.replace(['Neonate Birth to 29 days', 'Infant 29 days to < 2 years'], "0-2 yea
```

In [124…
```
df10["Age"].value_counts()
```

Out[124]:
```
Age
0-2 years                      39060
12 years and up                37525
Child 2 years to < 6 years     23946
Child 6 years to < 12 years    22954
Name: count, dtype: int64
```

The cells below consolidate "Patient origin" into more discrete categories

In [125…
```
df10["Patient Origin"].value_counts()
```

Out[125]:
```
Patient Origin
Emergency Department                                                        47563
Another Hospital's Emergency Department                                     21884
General Care Floor                                                          18033
Operating Room (Direct to ICU)                                              17792
Recovery Room (PACU)                                                         9248
Another Hospital's ICU                                                       1796
Another Hospital's General Care Floor                                        1753
Step-Down Unit/Intermediate Care Unit                                        1374
Home                                                                         1120
Physician's Office/Clinic                                                     959
Inpatient Procedure Suite (not cath lab)                                      558
Outpatient Procedure Suite                                                    255
NICU (in this hospital)                                                       229
Another Hospital's OR                                                         205
Other                                                                         151
Another ICU in this hospital (except NICU)                                    129
Transitional Care/Skilled Nursing/Chronic Care Facility                       122
Dedicated technology dependent unit (transitional/progressive care unit)      104
Physical Rehab Center                                                          64
Cath lab                                                                       57
Another hospital's cath lab                                                    17
Another hospital's Step-Down Unit/Intermediate Care Unit                       16
Psychiatric/Substance Abuse/Chemical Dependence Rehab Center                   13
Another Hospital's dedicated home ventilator unit                              13
Delivery Room (including delivery room in another hospital)                    12
Telemetry Unit                                                                 10
Pulmonary Rehab Center                                                          7
Another hospital's Telemetry Unit                                               1
Name: count, dtype: int64
```

In [126…
```python
df11 = df10.copy()
df11["Patient Origin"].replace(["Another Hospital's Emergency Department"], "Emergency
df11["Patient Origin"].replace(["Other", "Step-Down Unit/Intermediate Care Unit", "Tel
df11["Patient Origin"].replace(["Delivery Room (including delivery room in another hos
df11["Patient Origin"].replace(["Another Hospital's OR", "Another hospital's cath lab"
```

In [127…
```python
df11["Patient Origin"].value_counts()
```

Out[127]:
```
Patient Origin
Emergency Department    69447
OR/Procedure            28132
Lower level of care     23740
Another ICU              2166
Name: count, dtype: int64
```

Remove rows with PIM3 recovery from surgery showing a recovery from cardiac surgery

In [128…
```python
df11["PIM 3 Recovery from Surgery"].value_counts()
```

Out[128]:
```
PIM 3 Recovery from Surgery
No                                                99362
Yes, Recovery from non-cardiac procedure          23992
Yes, Recovery from non-bypass cardiac procedure      98
Yes, Recovery from bypass cardiac procedure          33
Name: count, dtype: int64
```

In [129…
```
df12 = df11.copy()
df12 = df12.drop(df12[df12['PIM 3 Recovery from Surgery'] == 'Yes, Recovery from non-b
df12 = df12.drop(df12[df12['PIM 3 Recovery from Surgery'] == 'Yes, Recovery from bypas
df12 = df12.reset_index(drop=True)
```

In [130…
```
df12["PIM 3 Recovery from Surgery"].value_counts()
```

Out[130]:
```
PIM 3 Recovery from Surgery
No                                       99362
Yes, Recovery from non-cardiac procedure    23992
Name: count, dtype: int64
```

Fill Race NaN with "Unspecified"

In [131…
```
df12.Race.fillna('Unspecified', inplace=True)
df12.Race.unique()
```

Out[131]:
```
array(['White', 'Other/Mixed', 'Hispanic or Latino',
       'Black or African American', 'Asian/Indian/Pacific Islander',
       'Unspecified', 'Asian', 'American Indian or Alaska Native',
       'Native Hawaiian or Other Pacific Islander'], dtype=object)
```

Consolidate Pupillary reactions to Fixed>3mm and others/unknown

In [132…
```
df12["PIM 3 Pupillary Reaction"].value_counts()
```

Out[132]:
```
PIM 3 Pupillary Reaction
Other                                 116524
Unknown                                 5040
>3mm and both fixed                      927
Pupilary Assessment Invalid - Drugs      651
Pupilary Assessment Invalid - Injury     141
Pupilary Assessment Invalid - Toxins      71
Name: count, dtype: int64
```

In [133…
```
df13 = df12.copy()
df13["PIM 3 Pupillary Reaction"].replace(["Other", "Unknown", "Pupilary Assessment Inv
df13["PIM 3 Pupillary Reaction"].value_counts()
```

Out[133]:
```
PIM 3 Pupillary Reaction
Other/Unknown          122427
>3mm and both fixed       927
Name: count, dtype: int64
```

In [134…
```
chatBot = SmartDataframe(df13, config={"llm": llm})
```

In [135…
```
chatBot.chat("For the column 'Physical Length of Stay (days)', what percentage of rows
```

Out[135]:
```
"The percentage of rows with 'Physical Length of Stay (days)' more than 1.5 is: 52.7
2%"
```

In [69]:
```
chatBot.chat("For the column 'Physical Length of Stay (days)', what percentage of rows
```

Out[69]:
```
40.21012184148447
```

In [138…
```
LOS = chatBot.chat("Create a new dataframe with column'LOS', and if the value in colum
LOS.info()
```

```
<class 'pandas.core.frame.DataFrame'>
RangeIndex: 123354 entries, 0 to 123353
Data columns (total 1 columns):
 #   Column  Non-Null Count   Dtype
---  ------  --------------   -----
 0   LOS     123354 non-null  int64
dtypes: int64(1)
memory usage: 963.8 KB
```

In [139…

```
df14 = pd.concat([df13, LOS], axis=1)
df14.info()
```

```
<class 'pandas.core.frame.DataFrame'>
RangeIndex: 123354 entries, 0 to 123353
Data columns (total 42 columns):
 #   Column                                             Non-Null Count   Dtype  
---  ------                                             --------------   -----  
 0   Case Index Id                                      123354 non-null  object 
 1   Is Readmission                                     123354 non-null  int64  
 2   Age                                                123354 non-null  object 
 3   Weight (kg)                                        123354 non-null  float64
 4   Gender                                             123354 non-null  object 
 5   Race                                               123354 non-null  object 
 6   Patient Origin                                     123354 non-null  object 
 7   Trauma                                             123354 non-null  int64  
 8   Patient Type                                       123354 non-null  object 
 9   Transport Team                                     98391 non-null   object 
 10  Transport Vehicle                                  104468 non-null  object 
 11  Post Operative                                     123354 non-null  int64  
 12  Baseline PCPC                                      123354 non-null  object 
 13  Baseline POPC                                      123354 non-null  object 
 14  Baseline FSS                                       16666 non-null   float64
 15  Cardiac Patient                                    123354 non-null  int64  
 16  Cardiac Procedure directly prior to or during stay 123354 non-null  int64  
 17  Medical Length of Stay (days)                      118191 non-null  float64
 18  Physical Length of Stay (days)                     123354 non-null  float64
 19  Hospital LOS                                       123115 non-null  float64
 20  Outcome                                            123354 non-null  object 
 21  Disposition                                        123354 non-null  object 
 22  Is Altered Code                                    122862 non-null  float64
 23  Discharge PCPC                                     118592 non-null  object 
 24  Discharge POPC                                     118566 non-null  object 
 25  Discharge FSS                                      16359 non-null   float64
 26  Hospital Outcome                                   122609 non-null  object 
 27  PIM 3 Score                                        123354 non-null  float64
 28  PIM 3 Probability of Death                         123354 non-null  float64
 29  Mechanical Ventilation (First Hour)                123354 non-null  float64
 30  Elective Admission to ICU                          123354 non-null  float64
 31  PIM 3 Recovery from Surgery                        123354 non-null  object 
 32  Systolic Blood Pressure                            123354 non-null  float64
 33  PaO2 (mmHg)                                        3179 non-null    float64
 34  FiO2                                               3179 non-null    float64
 35  Base Excess                                        123354 non-null  float64
 36  PIM 3 Pupillary Reaction                           123354 non-null  object 
 37  PIM 3 - No Very High Risk Dx                       123354 non-null  int64  
 38  PIM 3 - No High Risk Dx                            123354 non-null  int64  
 39  PIM 3 - No Low Risk Dx                             123354 non-null  int64  
 40  Category                                           123354 non-null  object 
 41  LOS                                                123354 non-null  int64  
dtypes: float64(15), int64(9), object(18)
memory usage: 39.5+ MB
```

Apply Ordinal Encoder to convert Object datatype to Integers

In [140…

```
toEncode = df14[['Is Readmission', 'Age', 'Gender', 'Race', 'Patient Origin', 'Trauma'
                'Post Operative', 'Mechanical Ventilation (First Hour)', 'Elective Adm
                'PIM 3 Recovery from Surgery', 'Category', 'Baseline PCPC', 'Baseline 
                 'PIM 3 Pupillary Reaction']].copy()
columnNames = list(toEncode.columns)

enc = OrdinalEncoder()
```

```
df15 = pd.DataFrame(enc.fit_transform(toEncode), columns= columnNames)

for i in range(len(columnNames)):
    print (columnNames[i] , enc.categories_[i])
```

```
Is Readmission [0 1]
Age ['0-2 years' '12 years and up' 'Child 2 years to < 6 years'
 'Child 6 years to < 12 years']
Gender ['Ambiguous' 'Female' 'Male']
Race ['American Indian or Alaska Native' 'Asian'
 'Asian/Indian/Pacific Islander' 'Black or African American'
 'Hispanic or Latino' 'Native Hawaiian or Other Pacific Islander'
 'Other/Mixed' 'Unspecified' 'White']
Patient Origin ['Another ICU' 'Emergency Department' 'Lower level of care' 'OR/Proced
ure']
Trauma [0 1]
Patient Type ['Scheduled (> or = 12 Hours in Advance)' 'Unscheduled']
Post Operative [0 1]
Mechanical Ventilation (First Hour) [0. 1.]
Elective Admission to ICU [0. 1.]
PIM 3 Recovery from Surgery ['No' 'Yes, Recovery from non-cardiac procedure']
Category ['Cardiovascular' 'Dermatologic' 'Endocrine' 'Factors Influencing Health'
 'Gastrointestinal' 'Genetic' 'Gynecologic' 'Hematologic' 'Immunologic'
 'Infectious' 'Injury/Poisoning/Adverse Effects' 'Metabolic' 'Neurologic'
 'Newborn/Perinatal' 'Oncologic' 'Ophthalmologic' 'Orthopedic'
 'Psychiatric' 'Renal/Genitourinary' 'Respiratory' 'Respiratory/ENT'
 'Rheumatologic' 'Symptoms' 'Transplant' 'Ungroupable']
Baseline PCPC ['1 - Normal' '2 - Mild disability' '3 - Moderate disability'
 '4 - Severe disability' '5 - Coma or vegetative state']
Baseline POPC ['1 - Good overall performance' '2 - Mild overall disability'
 '3 - Moderate overall disability' '4 - Severe overall disability'
 '5 - Coma or vegetative state']
PIM 3 Pupillary Reaction ['>3mm and both fixed' 'Other/Unknown']
```

In [141…

```
df15.info()
```

```
<class 'pandas.core.frame.DataFrame'>
RangeIndex: 123354 entries, 0 to 123353
Data columns (total 15 columns):
 #   Column                               Non-Null Count   Dtype  
---  ------                               --------------   -----  
 0   Is Readmission                       123354 non-null  float64
 1   Age                                  123354 non-null  float64
 2   Gender                               123354 non-null  float64
 3   Race                                 123354 non-null  float64
 4   Patient Origin                       123354 non-null  float64
 5   Trauma                               123354 non-null  float64
 6   Patient Type                         123354 non-null  float64
 7   Post Operative                       123354 non-null  float64
 8   Mechanical Ventilation (First Hour)  123354 non-null  float64
 9   Elective Admission to ICU            123354 non-null  float64
 10  PIM 3 Recovery from Surgery          123354 non-null  float64
 11  Category                             123354 non-null  float64
 12  Baseline PCPC                        123354 non-null  float64
 13  Baseline POPC                        123354 non-null  float64
 14  PIM 3 Pupillary Reaction             123354 non-null  float64
dtypes: float64(15)
memory usage: 14.1 MB
```

In [142…

```
for column in toEncode.columns:
    print(toEncode.value_counts(column))
```

```
Is Readmission
0    86939
1    36415
Name: count, dtype: int64
Age
0-2 years                       39018
12 years and up                 37486
Child 2 years to < 6 years      23923
Child 6 years to < 12 years     22927
Name: count, dtype: int64
Gender
Male         67785
Female       55567
Ambiguous        2
Name: count, dtype: int64
Race
White                                          57002
Hispanic or Latino                             25688
Black or African American                      21539
Other/Mixed                                     7686
Unspecified                                     5569
Asian                                           2487
Asian/Indian/Pacific Islander                   2367
American Indian or Alaska Native                 669
Native Hawaiian or Other Pacific Islander        347
Name: count, dtype: int64
Patient Origin
Emergency Department    69441
OR/Procedure            28013
Lower level of care     23736
Another ICU              2164
Name: count, dtype: int64
Trauma
0    111927
1     11427
Name: count, dtype: int64
Patient Type
Unscheduled                                 100194
Scheduled (> or = 12 Hours in Advance)     23160
Name: count, dtype: int64
Post Operative
0    91546
1    31808
Name: count, dtype: int64
Mechanical Ventilation (First Hour)
0.0    92863
1.0    30491
Name: count, dtype: int64
Elective Admission to ICU
0.0    100313
1.0     23041
Name: count, dtype: int64
PIM 3 Recovery from Surgery
No                                          99362
Yes, Recovery from non-cardiac procedure    23992
Name: count, dtype: int64
Category
Respiratory                         32884
Neurologic                          17803
Injury/Poisoning/Adverse Effects    17383
```

```
Infectious                           9619
Respiratory/ENT                      8361
Endocrine                            7587
Oncologic                            5614
Orthopedic                           3668
Gastrointestinal                     3569
Symptoms                             3296
Genetic                              3255
Cardiovascular                       2760
Metabolic                            1745
Renal/Genitourinary                  1682
Hematologic                           932
Ungroupable                           904
Psychiatric                           576
Dermatologic                          397
Factors Influencing Health            335
Transplant                            316
Rheumatologic                         266
Newborn/Perinatal                     213
Ophthalmologic                         92
Gynecologic                            49
Immunologic                            48
Name: count, dtype: int64
Baseline PCPC
1 - Normal                       86709
2 - Mild disability              13613
3 - Moderate disability          12376
4 - Severe disability            10391
5 - Coma or vegetative state       265
Name: count, dtype: int64
Baseline POPC
1 - Good overall performance        66573
2 - Mild overall disability         22301
3 - Moderate overall disability     22294
4 - Severe overall disability       11915
5 - Coma or vegetative state          271
Name: count, dtype: int64
PIM 3 Pupillary Reaction
Other/Unknown            122427
>3mm and both fixed         927
Name: count, dtype: int64
```

Add "weight" and "PIM3" Score into the dataframe

In [143…

```
df16 = df15.copy()

df16.insert(2, "Weight", 0)
df16.insert(15, "PIM3", 0)
df16.insert(16, "PIM 3 Probability of Death", 0)
df16.insert(17, "Systolic Blood Pressure", 0)
df16.insert(18, "Base Excess", 0)
df16.insert(20, "PIM 3 - No Very High Risk Dx", 0)
df16.insert(21, "PIM 3 - No High Risk Dx", 0)
df16.insert(22, "PIM 3 - No Low Risk Dx", 0)




df16.Weight = df14["Weight (kg)"].copy()
```

```
df16.PIM3 = df14["PIM 3 Score"].copy()
df16["PIM 3 Probability of Death"] = df14["PIM 3 Probability of Death"].copy()
df16["Systolic Blood Pressure"] = df14["Systolic Blood Pressure"].copy()
df16["Base Excess"] = df14["Base Excess"].copy()
df16["PIM 3 - No Very High Risk Dx"] = df14["PIM 3 - No Very High Risk Dx"].copy()
df16["PIM 3 - No High Risk Dx"] = df14["PIM 3 - No High Risk Dx"].copy()
df16["PIM 3 - No Low Risk Dx"] = df14["PIM 3 - No Low Risk Dx"].copy()


df16.info()
```

```
<class 'pandas.core.frame.DataFrame'>
RangeIndex: 123354 entries, 0 to 123353
Data columns (total 23 columns):
 #   Column                               Non-Null Count   Dtype
---  ------                               --------------   -----
 0   Is Readmission                       123354 non-null  float64
 1   Age                                  123354 non-null  float64
 2   Weight                               123354 non-null  float64
 3   Gender                               123354 non-null  float64
 4   Race                                 123354 non-null  float64
 5   Patient Origin                       123354 non-null  float64
 6   Trauma                               123354 non-null  float64
 7   Patient Type                         123354 non-null  float64
 8   Post Operative                       123354 non-null  float64
 9   Mechanical Ventilation (First Hour)  123354 non-null  float64
 10  Elective Admission to ICU            123354 non-null  float64
 11  PIM 3 Recovery from Surgery          123354 non-null  float64
 12  Category                             123354 non-null  float64
 13  Baseline PCPC                        123354 non-null  float64
 14  Baseline POPC                        123354 non-null  float64
 15  PIM3                                 123354 non-null  float64
 16  PIM 3 Probability of Death           123354 non-null  float64
 17  Systolic Blood Pressure              123354 non-null  float64
 18  Base Excess                          123354 non-null  float64
 19  PIM 3 Pupillary Reaction             123354 non-null  float64
 20  PIM 3 - No Very High Risk Dx         123354 non-null  int64
 21  PIM 3 - No High Risk Dx              123354 non-null  int64
 22  PIM 3 - No Low Risk Dx               123354 non-null  int64
dtypes: float64(20), int64(3)
memory usage: 21.6 MB
```

Add "LOS" into the dataframe and generate HEATMAP

In [144…

```
df17 = df16.copy()

df17.insert(23, "LOS", 0)
df17.LOS = df14.LOS.copy()
```

In [145…

```
df17.info()
```

```
<class 'pandas.core.frame.DataFrame'>
RangeIndex: 123354 entries, 0 to 123353
Data columns (total 24 columns):
 #   Column                                Non-Null Count   Dtype  
---  ------                                --------------   -----  
 0   Is Readmission                        123354 non-null  float64
 1   Age                                   123354 non-null  float64
 2   Weight                                123354 non-null  float64
 3   Gender                                123354 non-null  float64
 4   Race                                  123354 non-null  float64
 5   Patient Origin                        123354 non-null  float64
 6   Trauma                                123354 non-null  float64
 7   Patient Type                          123354 non-null  float64
 8   Post Operative                        123354 non-null  float64
 9   Mechanical Ventilation (First Hour)   123354 non-null  float64
 10  Elective Admission to ICU             123354 non-null  float64
 11  PIM 3 Recovery from Surgery           123354 non-null  float64
 12  Category                              123354 non-null  float64
 13  Baseline PCPC                         123354 non-null  float64
 14  Baseline POPC                         123354 non-null  float64
 15  PIM3                                  123354 non-null  float64
 16  PIM 3 Probability of Death            123354 non-null  float64
 17  Systolic Blood Pressure               123354 non-null  float64
 18  Base Excess                           123354 non-null  float64
 19  PIM 3 Pupillary Reaction              123354 non-null  float64
 20  PIM 3 - No Very High Risk Dx          123354 non-null  int64  
 21  PIM 3 - No High Risk Dx               123354 non-null  int64  
 22  PIM 3 - No Low Risk Dx                123354 non-null  int64  
 23  LOS                                   123354 non-null  int64  
dtypes: float64(20), int64(4)
memory usage: 22.6 MB
```

In [146…

```
df17.describe()
```

Out[146]:

	Is Readmission	Age	Weight	Gender	Race	Patient Origin	
**count**	123354.000000	123354.000000	123354.000000	123354.000000	123354.000000	123354.000000	12[illegible]
**mean**	0.295207	1.249356	30.388527	1.549500	5.816106	1.629068	
**std**	0.456138	1.091573	26.382641	0.497578	2.296383	0.850072	
**min**	0.000000	0.000000	0.000000	0.000000	0.000000	0.000000	
**25%**	0.000000	0.000000	10.200000	1.000000	4.000000	1.000000	
**50%**	0.000000	1.000000	19.700000	2.000000	7.000000	1.000000	
**75%**	1.000000	2.000000	47.100000	2.000000	8.000000	2.000000	
**max**	1.000000	3.000000	287.500000	2.000000	8.000000	3.000000	

In [147…

```
def heatMap(list, fontSize):
    corr = list.corr()
    corr= corr.round(decimals=2)
    plt.figure(figsize=(20, 8))
```

```
    sns.set(font_scale = 1.2)
    sns.heatmap(corr, cmap='RdBu', vmin=-1, vmax=1, annot=True, annot_kws={'fontsize':
```

In [148…

```
heatMap(df17, 12)
```

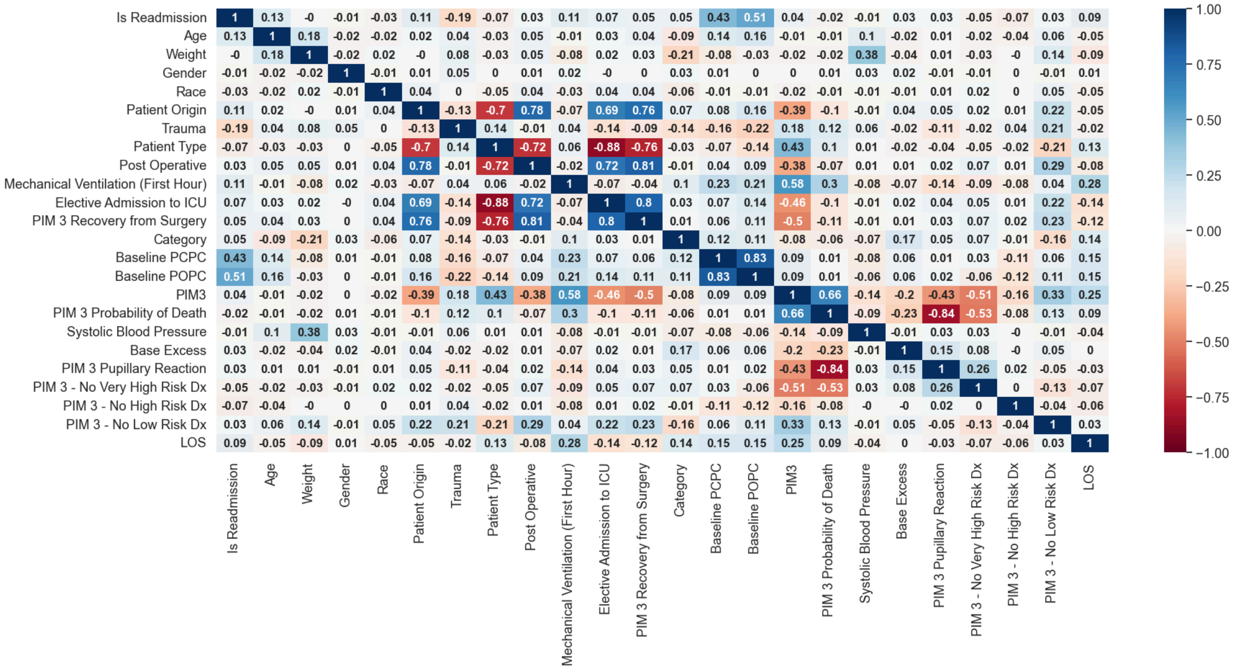

Create INPUT and OUTPUT NP arrays

In [149…

```
input = df16.copy().to_numpy()
output = df17["LOS"].copy().to_numpy()

#input.head()
print('Input sample 25: \n' , input[27] ,  "\n \n Output sample 25: " , output[27])
```

```
Input sample 25:
 [ 0.     0.    10.8    2.     7.     2.     0.     1.     1.     1.     0.     0.
  4.     1.     2.    -1.06  25.68  89.     0.     1.     0.     1.     1.   ]

 Output sample 25:  1
```

Apply SKLearn train/test split to Input and Output for 80:20 split

In [150…

```
X_train, X_test, y_train, y_test = train_test_split(input, output, test_size=0.2, ranc
```

In [151…

```
print(X_train.shape, "\t", y_train.shape, "\n")
print(X_test.shape, "\t", y_test.shape, "\n")
```

```
(98683, 23)      (98683,)

(24671, 23)      (24671,)
```

Fit MinMaxScaler on Training data, and then apply transformation to training and test set

In [152…

```
scaler = MinMaxScaler()
scaler.fit(X_train)
```

Out[152]:

```
▾ MinMaxScaler
MinMaxScaler()
```

In [153…

```
X_train = scaler.transform(X_train)
X_test = scaler.transform(X_test)
```

In [154…

```
print(X_test[27], '\n' '\n', y_test[27])
```

```
[1.         0.66666667 0.03930435 0.5        0.75       1.
 0.         0.         1.         1.         1.         1.
 0.5        0.75       0.75       0.17475124 0.00410287 0.42918455
 0.38844847 1.         1.         1.         1.        ]

 1
```

Import and apply LinearSVC - results appear Encouraging

Attempted standard SVC with RBF kernel too but computationally exorbitant

In [43]:

```
from sklearn.svm import LinearSVC
```

In [44]:

```
svc = LinearSVC(dual="auto", random_state=0, tol=1e-5)
svc.fit(X_train, y_train)
```

Out[44]:

```
▾ LinearSVC
LinearSVC(dual='auto', random_state=0, tol=1e-05)
```

In [45]:

```
svc_predictions = svc.predict(X_test)
```

In [46]:

```
def plotCM(test, predictions):

    print("accuracy Score is: " + str(accuracy_score(test, predictions)))

    cm = confusion_matrix(test, predictions, normalize='true')
    fig, ax = plt.subplots(figsize=(2,2))
    ConfusionMatrixDisplay(cm).plot(ax=ax, colorbar=False)
    plt.title('Sens/Spec')
    plt.show()

    cm2 = confusion_matrix(test, predictions, normalize='pred')
    fig, ax = plt.subplots(figsize=(2,2))
    ConfusionMatrixDisplay(cm2).plot(ax=ax, colorbar=False)
    plt.title('NPV/PPV')
    plt.show()

    RocCurveDisplay.from_predictions(test, predictions)
    plt.show()
```

In [47]:

```
plotCM(y_test, svc_predictions)
```

```
accuracy Score is: 0.668044747081712
```

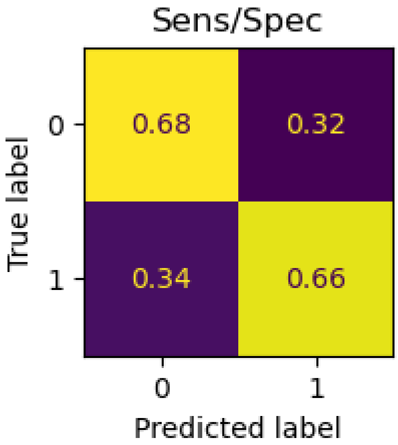

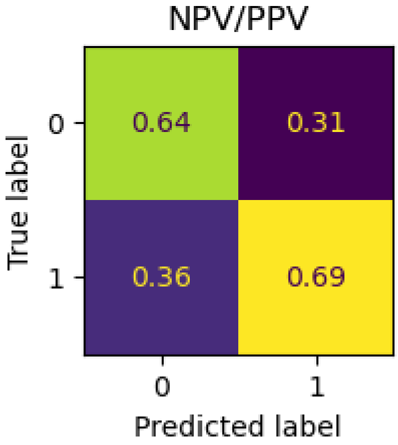

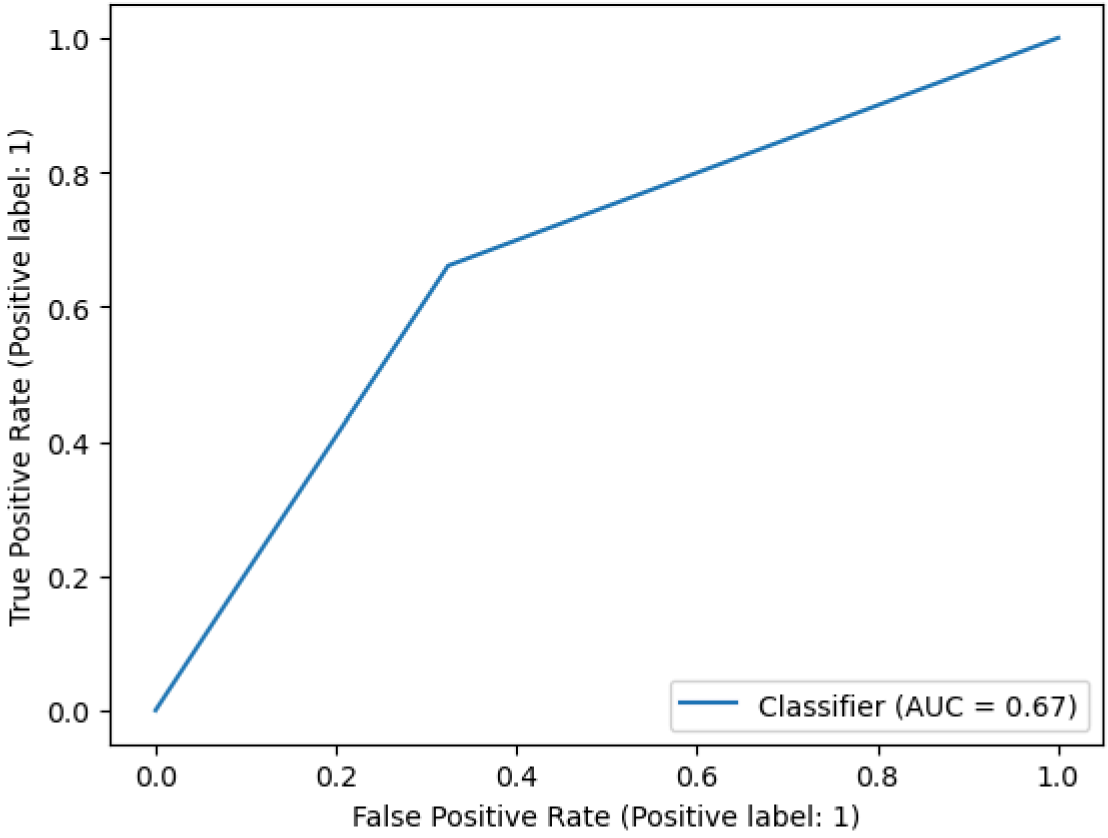

Import and apply Stochastic Gradient Descent Classification - results seen

In [48]:
```
from sklearn.linear_model import SGDClassifier
```

In [49]:
```
sgdc = SGDClassifier(loss="hinge", penalty="l2")
sgdc.fit(X_train, y_train)
```

Out[49]:
```
▾ SGDClassifier
SGDClassifier()
```

In [50]:
```
sgdc_predictions = sgdc.predict(X_test)
```

In [51]:
```
plotCM(y_test, sgdc_predictions)
```

```
accuracy Score is: 0.6626134889753567
```

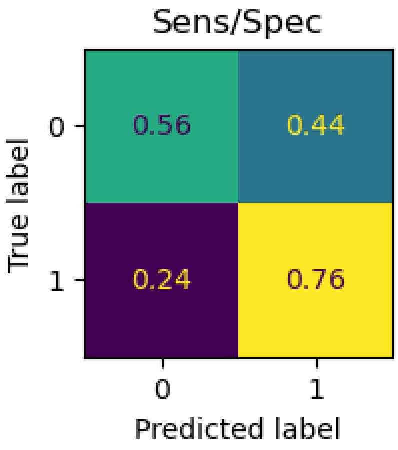

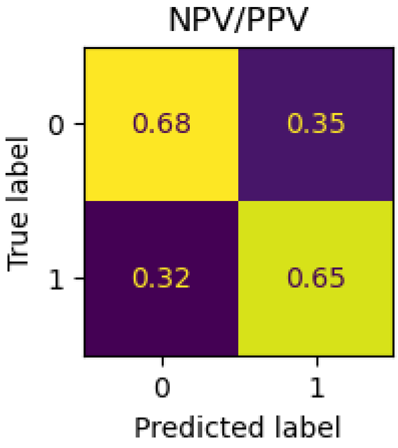

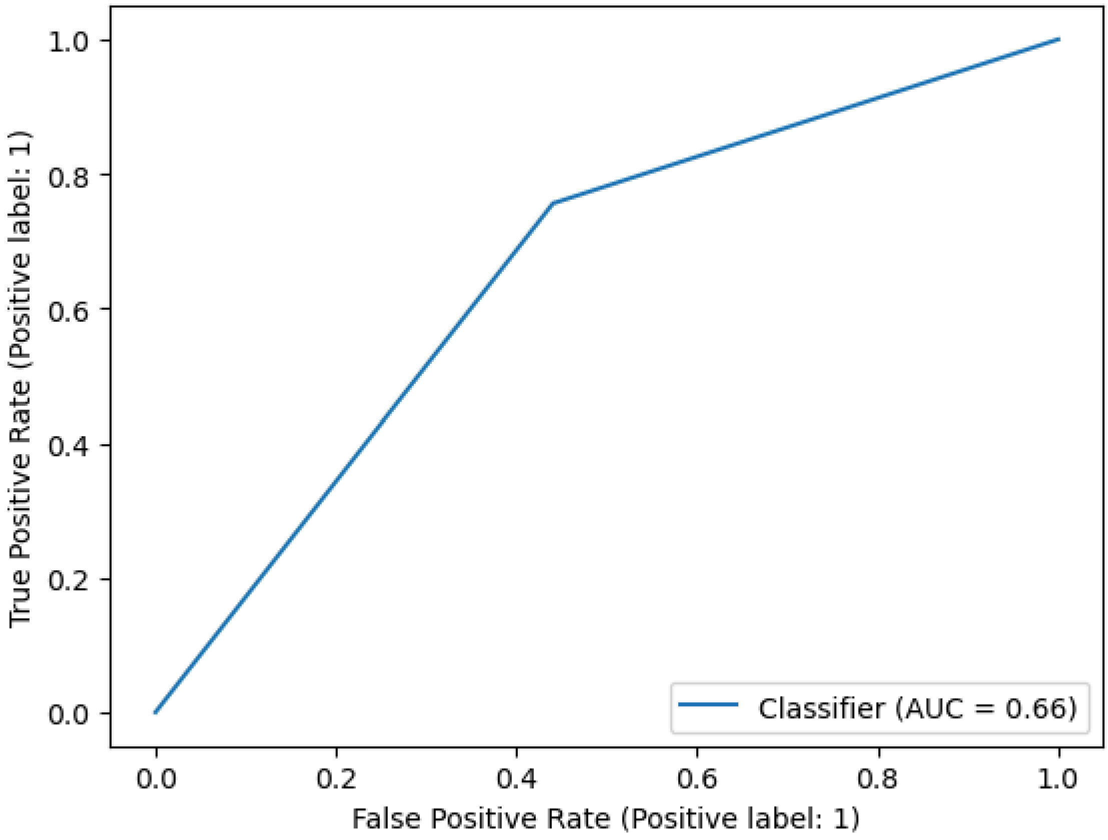

Import and apply KNN Classifier - results

In [52]:
```
from sklearn.neighbors import KNeighborsClassifier
```

In [53]:
```
knn = KNeighborsClassifier(n_neighbors=15)
knn.fit(X_train, y_train)
```

Out[53]:
```
▾ KNeighborsClassifier
KNeighborsClassifier(n_neighbors=15)
```

In [54]:
```
knn_predictions = knn.predict(X_test)
```

In [55]:
```
plotCM(y_test, knn_predictions)
```

```
accuracy Score is: 0.6817444876783398
```

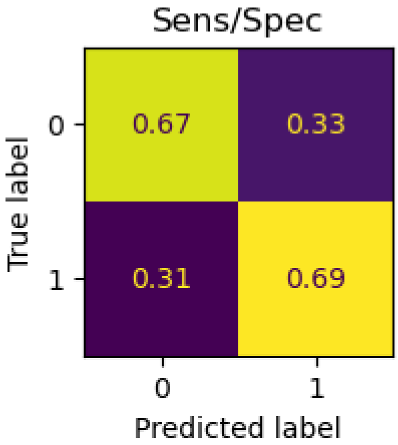

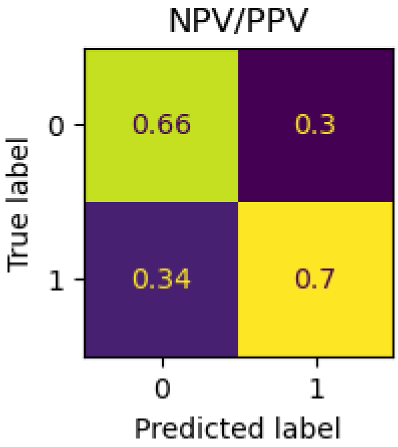

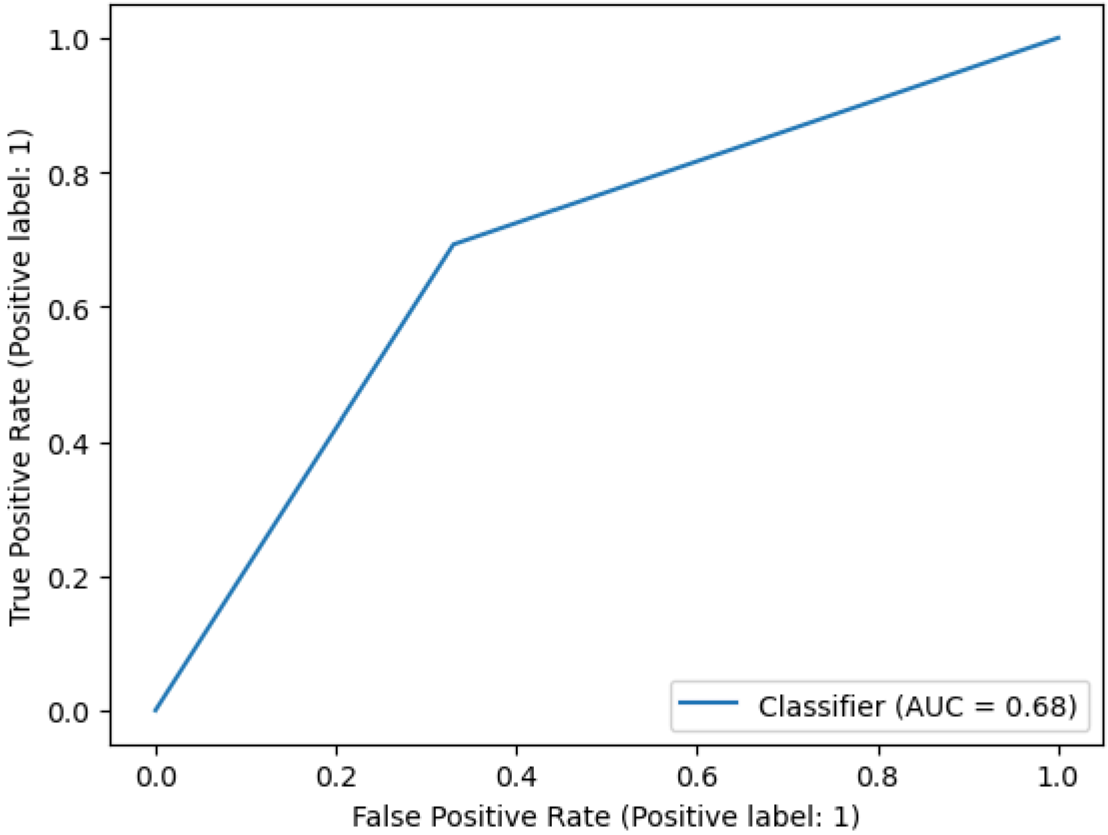

Import and apply Decision Tree Regression

In [56]:
```
from sklearn.tree import DecisionTreeClassifier
```

In [57]:
```
dtree = DecisionTreeClassifier(random_state=30)
dtree.fit(X_train, y_train)
```

Out[57]:
```
▾ DecisionTreeClassifier
DecisionTreeClassifier(random_state=30)
```

In [58]:
```
dtree_predictions = dtree.predict(X_test)
```

In [59]:
```
plotCM(y_test, dtree_predictions)
```

```
accuracy Score is: 0.6139753566796369
```

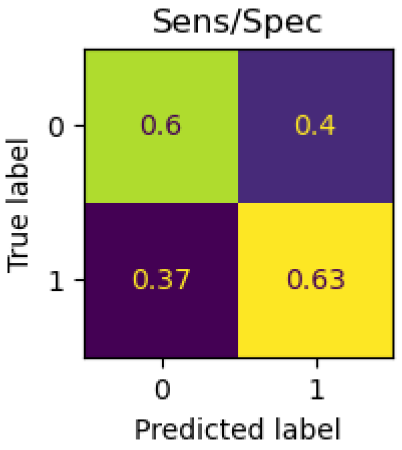

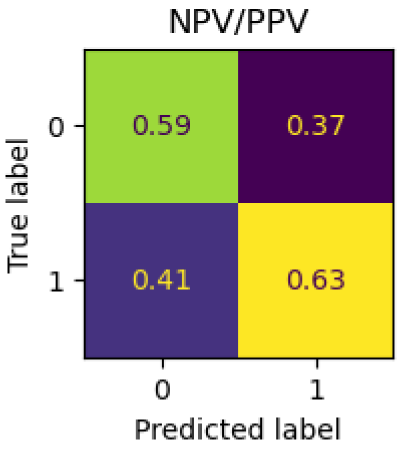

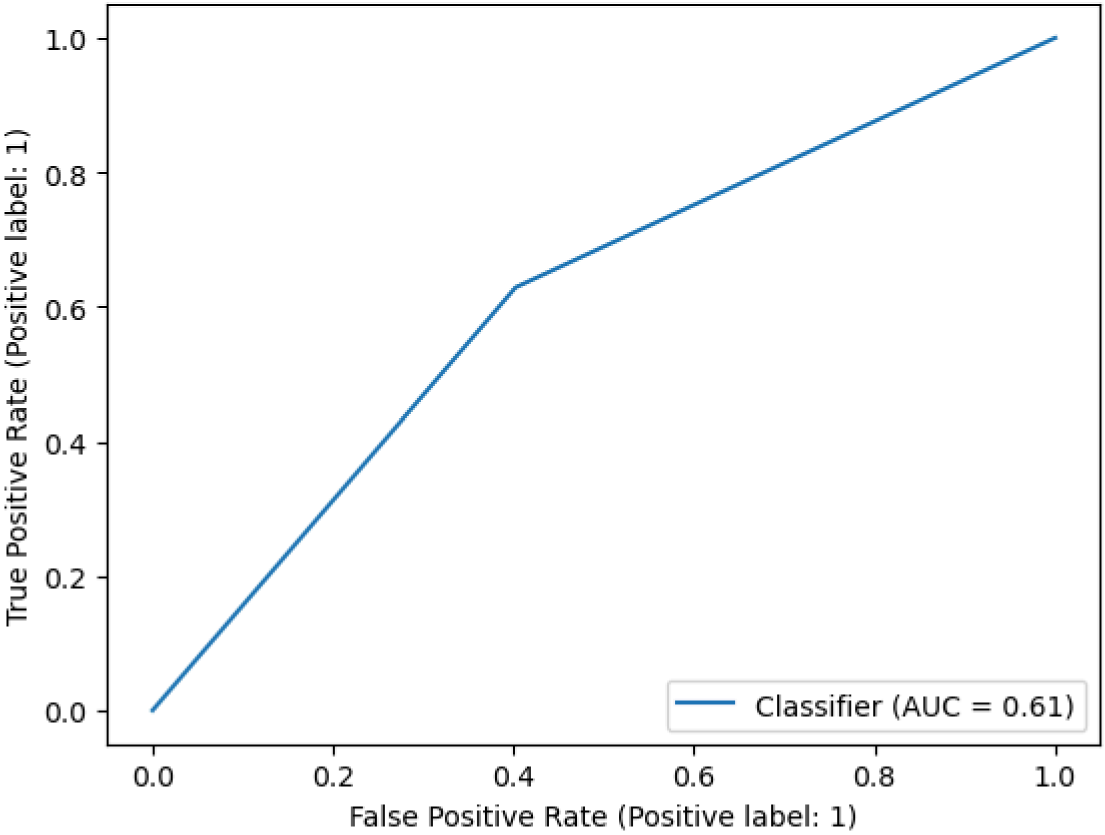

Attempt Gradient Boosting

In [60]:
```
from sklearn.ensemble import HistGradientBoostingClassifier
```

In [61]:
```
boost = HistGradientBoostingClassifier()
boost.fit(X_train, y_train)
```

Out[61]:
```
▾ HistGradientBoostingClassifier
HistGradientBoostingClassifier()
```

In [62]:
```
boost_predictions = boost.predict(X_test)
```

In [63]:
```
plotCM(y_test, boost_predictions)
```

```
accuracy Score is: 0.7088197146562906
```

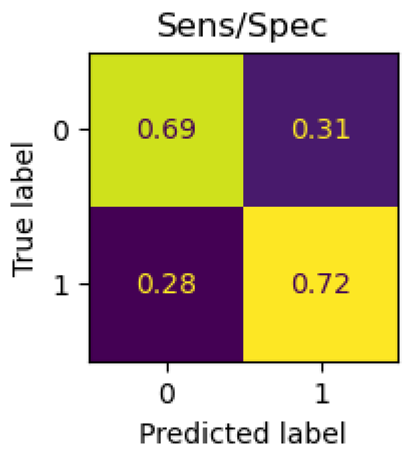

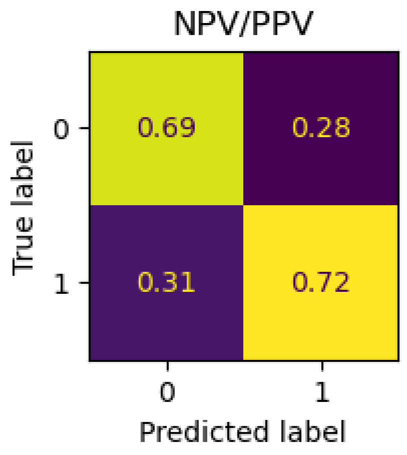

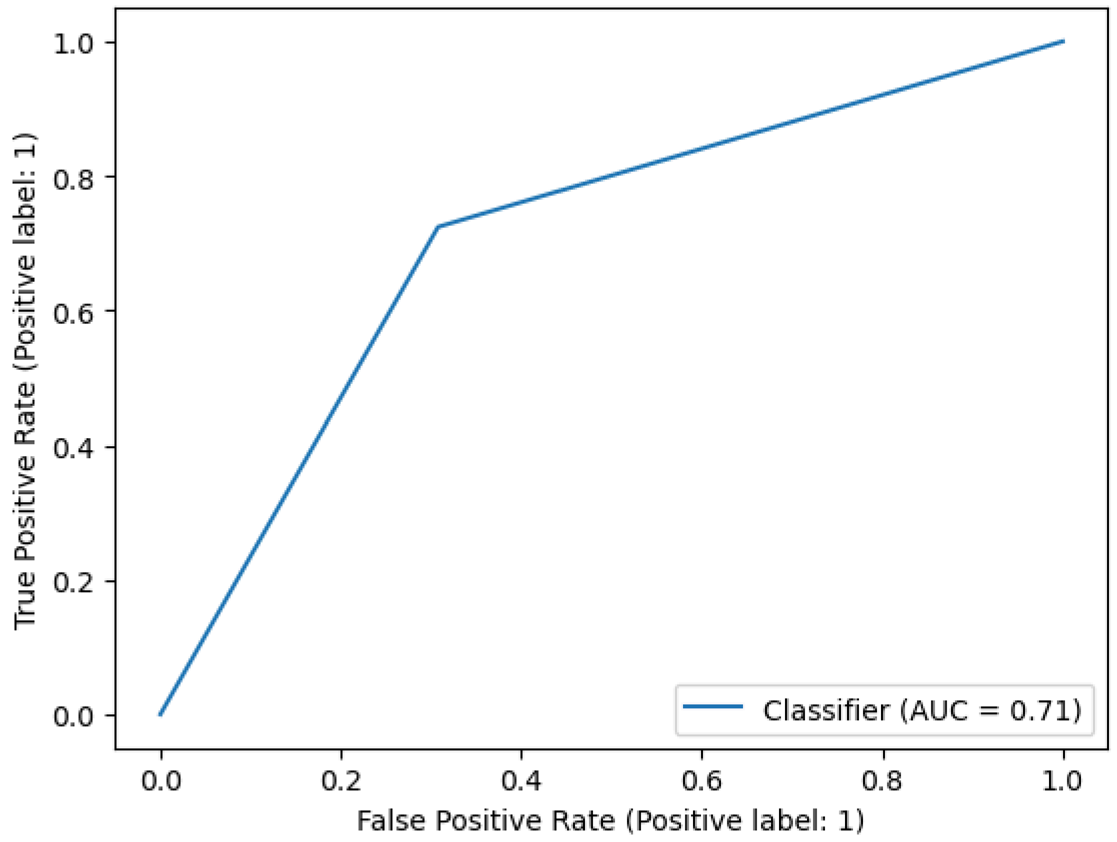

Attempt CatBoost

In [64]:
```
from catboost import CatBoostClassifier
import warnings
warnings.filterwarnings("ignore")
```

In [65]:
```
# Define the hyperparameters for the CatBoost algorithm
params = {'learning_rate': 0.1, 'depth': 6,
          'l2_leaf_reg': 3, 'iterations': 100}

# Initialize the CatBoostClassifier object
# with the defined hyperparameters and fit it on the training set
model = CatBoostClassifier(**params)
model.fit(X_train, y_train)
```

```
0:	learn: 0.6759380	total: 167ms	remaining: 16.6s
1:	learn: 0.6624980	total: 195ms	remaining: 9.58s
2:	learn: 0.6506852	total: 231ms	remaining: 7.45s
3:	learn: 0.6411415	total: 267ms	remaining: 6.4s
4:	learn: 0.6330452	total: 301ms	remaining: 5.72s
5:	learn: 0.6258763	total: 335ms	remaining: 5.25s
6:	learn: 0.6202392	total: 365ms	remaining: 4.85s
7:	learn: 0.6148481	total: 398ms	remaining: 4.58s
8:	learn: 0.6114958	total: 431ms	remaining: 4.36s
9:	learn: 0.6082475	total: 460ms	remaining: 4.14s
10:	learn: 0.6050706	total: 489ms	remaining: 3.96s
11:	learn: 0.6025151	total: 518ms	remaining: 3.8s
12:	learn: 0.6002888	total: 546ms	remaining: 3.65s
13:	learn: 0.5985273	total: 573ms	remaining: 3.52s
14:	learn: 0.5967239	total: 603ms	remaining: 3.42s
15:	learn: 0.5952956	total: 649ms	remaining: 3.4s
16:	learn: 0.5939082	total: 679ms	remaining: 3.31s
17:	learn: 0.5923581	total: 712ms	remaining: 3.24s
18:	learn: 0.5910080	total: 743ms	remaining: 3.17s
19:	learn: 0.5899067	total: 773ms	remaining: 3.09s
20:	learn: 0.5885535	total: 800ms	remaining: 3.01s
21:	learn: 0.5877166	total: 837ms	remaining: 2.97s
22:	learn: 0.5868793	total: 870ms	remaining: 2.91s
23:	learn: 0.5861203	total: 909ms	remaining: 2.88s
24:	learn: 0.5853985	total: 953ms	remaining: 2.86s
25:	learn: 0.5845819	total: 991ms	remaining: 2.82s
26:	learn: 0.5838812	total: 1.02s	remaining: 2.77s
27:	learn: 0.5830770	total: 1.06s	remaining: 2.73s
28:	learn: 0.5820559	total: 1.1s	remaining: 2.69s
29:	learn: 0.5815630	total: 1.13s	remaining: 2.64s
30:	learn: 0.5810238	total: 1.17s	remaining: 2.61s
31:	learn: 0.5805621	total: 1.21s	remaining: 2.57s
32:	learn: 0.5799005	total: 1.24s	remaining: 2.52s
33:	learn: 0.5793818	total: 1.28s	remaining: 2.49s
34:	learn: 0.5787558	total: 1.32s	remaining: 2.45s
35:	learn: 0.5784076	total: 1.36s	remaining: 2.41s
36:	learn: 0.5780406	total: 1.38s	remaining: 2.36s
37:	learn: 0.5774869	total: 1.41s	remaining: 2.31s
38:	learn: 0.5771921	total: 1.44s	remaining: 2.25s
39:	learn: 0.5769511	total: 1.47s	remaining: 2.2s
40:	learn: 0.5766178	total: 1.49s	remaining: 2.15s
41:	learn: 0.5761478	total: 1.53s	remaining: 2.11s
42:	learn: 0.5757176	total: 1.55s	remaining: 2.06s
43:	learn: 0.5753638	total: 1.58s	remaining: 2.01s
44:	learn: 0.5749988	total: 1.61s	remaining: 1.97s
45:	learn: 0.5747005	total: 1.64s	remaining: 1.92s
46:	learn: 0.5744176	total: 1.67s	remaining: 1.88s
47:	learn: 0.5741617	total: 1.7s	remaining: 1.84s
48:	learn: 0.5739468	total: 1.73s	remaining: 1.8s
49:	learn: 0.5736993	total: 1.76s	remaining: 1.76s
50:	learn: 0.5734815	total: 1.79s	remaining: 1.72s
51:	learn: 0.5733285	total: 1.82s	remaining: 1.68s
52:	learn: 0.5731779	total: 1.85s	remaining: 1.64s
53:	learn: 0.5727506	total: 1.88s	remaining: 1.6s
54:	learn: 0.5725278	total: 1.91s	remaining: 1.56s
55:	learn: 0.5724124	total: 1.94s	remaining: 1.53s
56:	learn: 0.5722501	total: 1.97s	remaining: 1.49s
57:	learn: 0.5721167	total: 2s	remaining: 1.45s
58:	learn: 0.5718530	total: 2.02s	remaining: 1.4s
59:	learn: 0.5716565	total: 2.05s	remaining: 1.36s
```

```
60:	learn: 0.5714589	total: 2.08s	remaining: 1.33s
61:	learn: 0.5712130	total: 2.1s	remaining: 1.29s
62:	learn: 0.5710602	total: 2.13s	remaining: 1.25s
63:	learn: 0.5708808	total: 2.16s	remaining: 1.22s
64:	learn: 0.5706436	total: 2.19s	remaining: 1.18s
65:	learn: 0.5704087	total: 2.23s	remaining: 1.15s
66:	learn: 0.5699991	total: 2.25s	remaining: 1.11s
67:	learn: 0.5696536	total: 2.28s	remaining: 1.07s
68:	learn: 0.5692881	total: 2.31s	remaining: 1.04s
69:	learn: 0.5689352	total: 2.33s	remaining: 1s
70:	learn: 0.5687140	total: 2.36s	remaining: 966ms
71:	learn: 0.5683918	total: 2.39s	remaining: 930ms
72:	learn: 0.5682411	total: 2.42s	remaining: 894ms
73:	learn: 0.5679700	total: 2.44s	remaining: 858ms
74:	learn: 0.5678661	total: 2.47s	remaining: 823ms
75:	learn: 0.5675793	total: 2.49s	remaining: 787ms
76:	learn: 0.5673331	total: 2.52s	remaining: 752ms
77:	learn: 0.5671200	total: 2.54s	remaining: 718ms
78:	learn: 0.5667889	total: 2.58s	remaining: 685ms
79:	learn: 0.5666646	total: 2.6s	remaining: 650ms
80:	learn: 0.5665622	total: 2.62s	remaining: 615ms
81:	learn: 0.5664570	total: 2.65s	remaining: 581ms
82:	learn: 0.5663470	total: 2.67s	remaining: 548ms
83:	learn: 0.5662283	total: 2.7s	remaining: 515ms
84:	learn: 0.5660183	total: 2.73s	remaining: 481ms
85:	learn: 0.5658741	total: 2.75s	remaining: 448ms
86:	learn: 0.5656390	total: 2.78s	remaining: 416ms
87:	learn: 0.5655537	total: 2.81s	remaining: 383ms
88:	learn: 0.5654250	total: 2.83s	remaining: 350ms
89:	learn: 0.5652344	total: 2.86s	remaining: 317ms
90:	learn: 0.5651191	total: 2.88s	remaining: 285ms
91:	learn: 0.5650298	total: 2.91s	remaining: 253ms
92:	learn: 0.5649105	total: 2.93s	remaining: 221ms
93:	learn: 0.5648206	total: 2.96s	remaining: 189ms
94:	learn: 0.5646914	total: 2.98s	remaining: 157ms
95:	learn: 0.5646088	total: 3.01s	remaining: 125ms
96:	learn: 0.5644176	total: 3.04s	remaining: 93.9ms
97:	learn: 0.5642808	total: 3.06s	remaining: 62.5ms
98:	learn: 0.5641394	total: 3.09s	remaining: 31.2ms
99:	learn: 0.5639856	total: 3.12s	remaining: 0us
```

Out[65]:

```
<catboost.core.CatBoostClassifier at 0x1ce562d6050>
```

In [66]:

```
y_pred = model.predict(X_test)
```

In [67]:

```
plotCM(y_test, y_pred)
```

```
accuracy Score is: 0.704523346303502
```

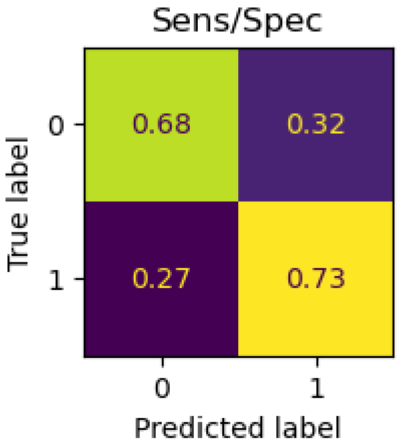

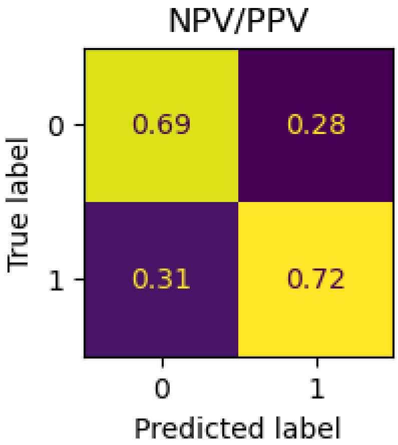

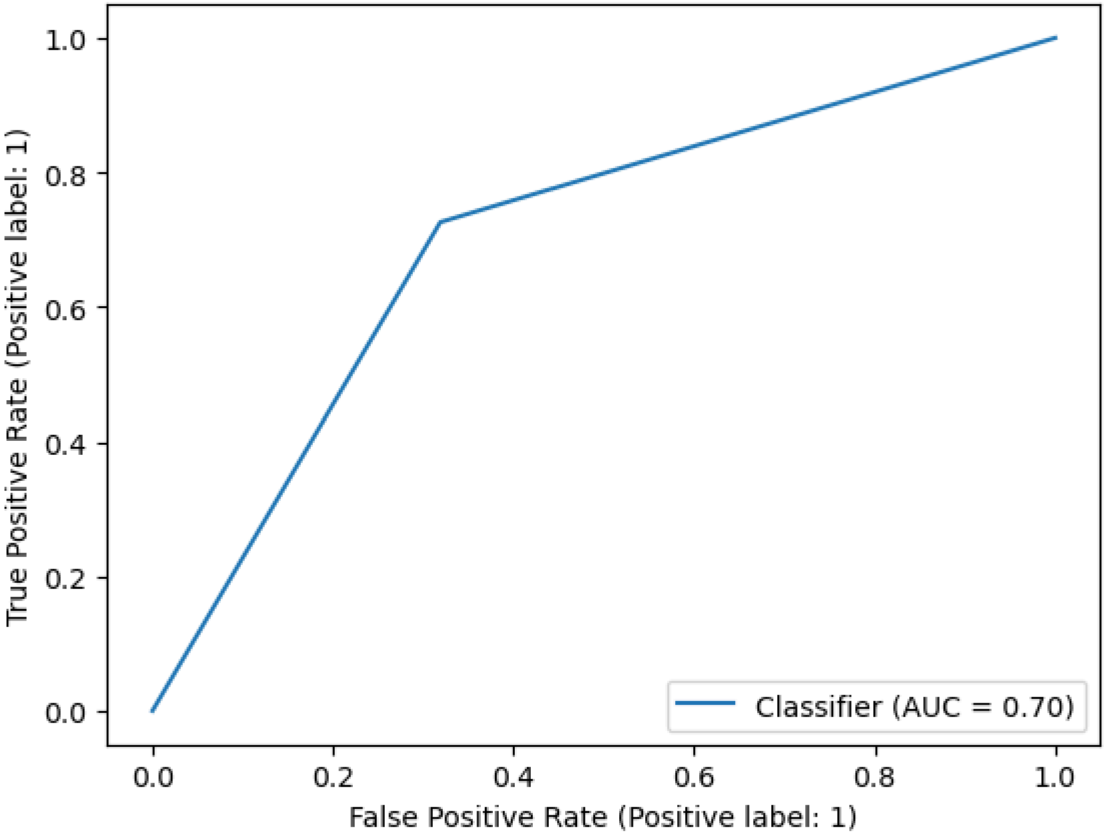

Neural Network

split Training data again in 75:25 ration to generate a total 60:20:20 split for Train:Validate:Test

In [155…
```
X_train, X_val, y_train, y_val = train_test_split(X_train, y_train, test_size=0.25, ra
```

In [156…
```
# Define function to plot Training Loss vs Validation Loss across epochs
def drawPlot():
    plt.figure(figsize=(6.4, 4.8))
    plt.plot(history.history['loss'])
    plt.plot(history.history['val_loss'])
    plt.title('model Loss')
    plt.ylabel('Loss')
    plt.xlabel('Epoch')
    #plt.xticks(np.arange(0, 21, 1.0))
    plt.legend(['train', 'val'], loc='upper left')
    plt.show()
```

In [159…
```
network = models.Sequential()
network.add(layers.Dense(512, activation='relu', input_shape=(23, )))
network.add(layers.Dense(256, activation='relu'))
network.add(layers.Dense(128, activation='relu'))
network.add(layers.Dense(64, activation='relu'))
network.add(layers.Dense(32, activation='relu'))
network.add(layers.Dense(16, activation='relu'))
network.add(layers.Dense(8, activation='relu'))
network.add(layers.Dense(4, activation='relu'))
network.add(layers.Dense(2, activation='relu'))
network.add(layers.Dense(1, activation='sigmoid'))
network.compile(optimizer=optimizers.RMSprop(),
                loss='binary_crossentropy',
                metrics=['accuracy'])
```

In [160…
```
# fit model
history = network.fit(X_train, y_train,
                      batch_size=128, epochs=200,
                      validation_data = (X_val, y_val))
```

```
Epoch 1/200
579/579 [==============================] - 5s 6ms/step - loss: 0.6339 - accuracy: 0.6
635 - val_loss: 0.6294 - val_accuracy: 0.6550
Epoch 2/200
579/579 [==============================] - 3s 5ms/step - loss: 0.6113 - accuracy: 0.6
829 - val_loss: 0.6056 - val_accuracy: 0.6866
Epoch 3/200
579/579 [==============================] - 3s 5ms/step - loss: 0.6041 - accuracy: 0.6
853 - val_loss: 0.6096 - val_accuracy: 0.6841
Epoch 4/200
579/579 [==============================] - 3s 5ms/step - loss: 0.6003 - accuracy: 0.6
884 - val_loss: 0.5979 - val_accuracy: 0.6892
Epoch 5/200
579/579 [==============================] - 3s 6ms/step - loss: 0.5975 - accuracy: 0.6
887 - val_loss: 0.5941 - val_accuracy: 0.6906
Epoch 6/200
579/579 [==============================] - 4s 6ms/step - loss: 0.5947 - accuracy: 0.6
901 - val_loss: 0.5937 - val_accuracy: 0.6905
Epoch 7/200
579/579 [==============================] - 3s 6ms/step - loss: 0.5926 - accuracy: 0.6
906 - val_loss: 0.6304 - val_accuracy: 0.6676
Epoch 8/200
579/579 [==============================] - 3s 6ms/step - loss: 0.5911 - accuracy: 0.6
913 - val_loss: 0.6049 - val_accuracy: 0.6751
Epoch 9/200
579/579 [==============================] - 4s 6ms/step - loss: 0.5892 - accuracy: 0.6
928 - val_loss: 0.5927 - val_accuracy: 0.6917
Epoch 10/200
579/579 [==============================] - 4s 6ms/step - loss: 0.5871 - accuracy: 0.6
930 - val_loss: 0.5897 - val_accuracy: 0.6944
Epoch 11/200
579/579 [==============================] - 3s 6ms/step - loss: 0.5854 - accuracy: 0.6
942 - val_loss: 0.5920 - val_accuracy: 0.6933
Epoch 12/200
579/579 [==============================] - 3s 6ms/step - loss: 0.5840 - accuracy: 0.6
949 - val_loss: 0.5915 - val_accuracy: 0.6919
Epoch 13/200
579/579 [==============================] - 4s 6ms/step - loss: 0.5824 - accuracy: 0.6
949 - val_loss: 0.5870 - val_accuracy: 0.6940
Epoch 14/200
579/579 [==============================] - 4s 7ms/step - loss: 0.5817 - accuracy: 0.6
960 - val_loss: 0.5912 - val_accuracy: 0.6922
Epoch 15/200
579/579 [==============================] - 4s 7ms/step - loss: 0.5804 - accuracy: 0.6
984 - val_loss: 0.5855 - val_accuracy: 0.6954
Epoch 16/200
579/579 [==============================] - 3s 6ms/step - loss: 0.5785 - accuracy: 0.6
991 - val_loss: 0.5890 - val_accuracy: 0.6936
Epoch 17/200
579/579 [==============================] - 3s 6ms/step - loss: 0.5776 - accuracy: 0.7
002 - val_loss: 0.5851 - val_accuracy: 0.6972
Epoch 18/200
579/579 [==============================] - 4s 6ms/step - loss: 0.5762 - accuracy: 0.6
991 - val_loss: 0.5887 - val_accuracy: 0.6992
Epoch 19/200
579/579 [==============================] - 4s 8ms/step - loss: 0.5751 - accuracy: 0.7
015 - val_loss: 0.5921 - val_accuracy: 0.6924
Epoch 20/200
579/579 [==============================] - 4s 7ms/step - loss: 0.5742 - accuracy: 0.7
021 - val_loss: 0.5866 - val_accuracy: 0.6924
```

```
Epoch 21/200
579/579 [==============================] - 5s 9ms/step - loss: 0.5733 - accuracy: 0.7
020 - val_loss: 0.5841 - val_accuracy: 0.6953
Epoch 22/200
579/579 [==============================] - 5s 8ms/step - loss: 0.5717 - accuracy: 0.7
031 - val_loss: 0.5876 - val_accuracy: 0.6892
Epoch 23/200
579/579 [==============================] - 4s 8ms/step - loss: 0.5712 - accuracy: 0.7
052 - val_loss: 0.5850 - val_accuracy: 0.6954
Epoch 24/200
579/579 [==============================] - 4s 7ms/step - loss: 0.5697 - accuracy: 0.7
045 - val_loss: 0.5809 - val_accuracy: 0.6996
Epoch 25/200
579/579 [==============================] - 4s 7ms/step - loss: 0.5690 - accuracy: 0.7
055 - val_loss: 0.5852 - val_accuracy: 0.6966
Epoch 26/200
579/579 [==============================] - 4s 6ms/step - loss: 0.5684 - accuracy: 0.7
065 - val_loss: 0.5827 - val_accuracy: 0.6939
Epoch 27/200
579/579 [==============================] - 3s 5ms/step - loss: 0.5672 - accuracy: 0.7
062 - val_loss: 0.6243 - val_accuracy: 0.6795
Epoch 28/200
579/579 [==============================] - 3s 6ms/step - loss: 0.5667 - accuracy: 0.7
070 - val_loss: 0.5797 - val_accuracy: 0.6999
Epoch 29/200
579/579 [==============================] - 3s 6ms/step - loss: 0.5657 - accuracy: 0.7
087 - val_loss: 0.5834 - val_accuracy: 0.6959
Epoch 30/200
579/579 [==============================] - 4s 6ms/step - loss: 0.5648 - accuracy: 0.7
086 - val_loss: 0.5906 - val_accuracy: 0.6902
Epoch 31/200
579/579 [==============================] - 4s 6ms/step - loss: 0.5632 - accuracy: 0.7
095 - val_loss: 0.5851 - val_accuracy: 0.6975
Epoch 32/200
579/579 [==============================] - 4s 7ms/step - loss: 0.5635 - accuracy: 0.7
089 - val_loss: 0.5904 - val_accuracy: 0.6928
Epoch 33/200
579/579 [==============================] - 4s 7ms/step - loss: 0.5621 - accuracy: 0.7
098 - val_loss: 0.5862 - val_accuracy: 0.6989
Epoch 34/200
579/579 [==============================] - 4s 7ms/step - loss: 0.5608 - accuracy: 0.7
107 - val_loss: 0.5797 - val_accuracy: 0.7028
Epoch 35/200
579/579 [==============================] - 4s 7ms/step - loss: 0.5605 - accuracy: 0.7
120 - val_loss: 0.5854 - val_accuracy: 0.6991
Epoch 36/200
579/579 [==============================] - 4s 6ms/step - loss: 0.5593 - accuracy: 0.7
115 - val_loss: 0.5879 - val_accuracy: 0.6975
Epoch 37/200
579/579 [==============================] - 3s 6ms/step - loss: 0.5582 - accuracy: 0.7
124 - val_loss: 0.5801 - val_accuracy: 0.7010
Epoch 38/200
579/579 [==============================] - 4s 6ms/step - loss: 0.5576 - accuracy: 0.7
124 - val_loss: 0.5887 - val_accuracy: 0.7007
Epoch 39/200
579/579 [==============================] - 4s 7ms/step - loss: 0.5571 - accuracy: 0.7
144 - val_loss: 0.5899 - val_accuracy: 0.7002
Epoch 40/200
579/579 [==============================] - 3s 6ms/step - loss: 0.5556 - accuracy: 0.7
151 - val_loss: 0.6068 - val_accuracy: 0.6959
```

```
Epoch 41/200
579/579 [==============================] - 3s 6ms/step - loss: 0.5545 - accuracy: 0.7
163 - val_loss: 0.5903 - val_accuracy: 0.6933
Epoch 42/200
579/579 [==============================] - 4s 7ms/step - loss: 0.5533 - accuracy: 0.7
171 - val_loss: 0.5848 - val_accuracy: 0.6947
Epoch 43/200
579/579 [==============================] - 3s 6ms/step - loss: 0.5529 - accuracy: 0.7
183 - val_loss: 0.5926 - val_accuracy: 0.6976
Epoch 44/200
579/579 [==============================] - 3s 6ms/step - loss: 0.5520 - accuracy: 0.7
179 - val_loss: 0.5972 - val_accuracy: 0.6956
Epoch 45/200
579/579 [==============================] - 3s 6ms/step - loss: 0.5507 - accuracy: 0.7
191 - val_loss: 0.5981 - val_accuracy: 0.6943
Epoch 46/200
579/579 [==============================] - 3s 6ms/step - loss: 0.5500 - accuracy: 0.7
197 - val_loss: 0.5891 - val_accuracy: 0.6978
Epoch 47/200
579/579 [==============================] - 3s 5ms/step - loss: 0.5488 - accuracy: 0.7
191 - val_loss: 0.5963 - val_accuracy: 0.6989
Epoch 48/200
579/579 [==============================] - 3s 5ms/step - loss: 0.5481 - accuracy: 0.7
205 - val_loss: 0.5854 - val_accuracy: 0.6985
Epoch 49/200
579/579 [==============================] - 3s 5ms/step - loss: 0.5464 - accuracy: 0.7
206 - val_loss: 0.5884 - val_accuracy: 0.6971
Epoch 50/200
579/579 [==============================] - 3s 5ms/step - loss: 0.5449 - accuracy: 0.7
236 - val_loss: 0.5959 - val_accuracy: 0.6871
Epoch 51/200
579/579 [==============================] - 3s 5ms/step - loss: 0.5448 - accuracy: 0.7
229 - val_loss: 0.5926 - val_accuracy: 0.6948
Epoch 52/200
579/579 [==============================] - 3s 5ms/step - loss: 0.5432 - accuracy: 0.7
242 - val_loss: 0.5918 - val_accuracy: 0.6994
Epoch 53/200
579/579 [==============================] - 3s 5ms/step - loss: 0.5429 - accuracy: 0.7
246 - val_loss: 0.6006 - val_accuracy: 0.6923
Epoch 54/200
579/579 [==============================] - 3s 5ms/step - loss: 0.5417 - accuracy: 0.7
254 - val_loss: 0.6026 - val_accuracy: 0.6960
Epoch 55/200
579/579 [==============================] - 3s 5ms/step - loss: 0.5417 - accuracy: 0.7
257 - val_loss: 0.5959 - val_accuracy: 0.6962
Epoch 56/200
579/579 [==============================] - 3s 5ms/step - loss: 0.5405 - accuracy: 0.7
261 - val_loss: 0.5890 - val_accuracy: 0.7030
Epoch 57/200
579/579 [==============================] - 3s 6ms/step - loss: 0.5400 - accuracy: 0.7
261 - val_loss: 0.5998 - val_accuracy: 0.7002
Epoch 58/200
579/579 [==============================] - 3s 5ms/step - loss: 0.5390 - accuracy: 0.7
263 - val_loss: 0.5985 - val_accuracy: 0.6894
Epoch 59/200
579/579 [==============================] - 3s 6ms/step - loss: 0.5381 - accuracy: 0.7
276 - val_loss: 0.6023 - val_accuracy: 0.6998
Epoch 60/200
579/579 [==============================] - 3s 5ms/step - loss: 0.5361 - accuracy: 0.7
293 - val_loss: 0.6247 - val_accuracy: 0.6852
```

```
Epoch 61/200
579/579 [==============================] - 3s 5ms/step - loss: 0.5357 - accuracy: 0.7
282 - val_loss: 0.6195 - val_accuracy: 0.6806
Epoch 62/200
579/579 [==============================] - 3s 6ms/step - loss: 0.5352 - accuracy: 0.7
298 - val_loss: 0.6065 - val_accuracy: 0.6841
Epoch 63/200
579/579 [==============================] - 3s 5ms/step - loss: 0.5334 - accuracy: 0.7
300 - val_loss: 0.5987 - val_accuracy: 0.6955
Epoch 64/200
579/579 [==============================] - 4s 6ms/step - loss: 0.5324 - accuracy: 0.7
308 - val_loss: 0.6035 - val_accuracy: 0.6943
Epoch 65/200
579/579 [==============================] - 4s 6ms/step - loss: 0.5326 - accuracy: 0.7
326 - val_loss: 0.5999 - val_accuracy: 0.6927
Epoch 66/200
579/579 [==============================] - 3s 6ms/step - loss: 0.5312 - accuracy: 0.7
314 - val_loss: 0.6022 - val_accuracy: 0.6956
Epoch 67/200
579/579 [==============================] - 4s 6ms/step - loss: 0.5298 - accuracy: 0.7
335 - val_loss: 0.6202 - val_accuracy: 0.6958
Epoch 68/200
579/579 [==============================] - 3s 6ms/step - loss: 0.5295 - accuracy: 0.7
331 - val_loss: 0.6213 - val_accuracy: 0.6933
Epoch 69/200
579/579 [==============================] - 3s 6ms/step - loss: 0.5280 - accuracy: 0.7
341 - val_loss: 0.6041 - val_accuracy: 0.6969
Epoch 70/200
579/579 [==============================] - 3s 5ms/step - loss: 0.5279 - accuracy: 0.7
355 - val_loss: 0.6164 - val_accuracy: 0.6877
Epoch 71/200
579/579 [==============================] - 4s 6ms/step - loss: 0.5274 - accuracy: 0.7
360 - val_loss: 0.6113 - val_accuracy: 0.6919
Epoch 72/200
579/579 [==============================] - 4s 6ms/step - loss: 0.5259 - accuracy: 0.7
356 - val_loss: 0.6407 - val_accuracy: 0.6988
Epoch 73/200
579/579 [==============================] - 3s 6ms/step - loss: 0.5254 - accuracy: 0.7
367 - val_loss: 0.6243 - val_accuracy: 0.6959
Epoch 74/200
579/579 [==============================] - 4s 6ms/step - loss: 0.5240 - accuracy: 0.7
361 - val_loss: 0.6180 - val_accuracy: 0.6937
Epoch 75/200
579/579 [==============================] - 3s 5ms/step - loss: 0.5238 - accuracy: 0.7
364 - val_loss: 0.6128 - val_accuracy: 0.6978
Epoch 76/200
579/579 [==============================] - 3s 5ms/step - loss: 0.5227 - accuracy: 0.7
379 - val_loss: 0.6164 - val_accuracy: 0.6919
Epoch 77/200
579/579 [==============================] - 3s 6ms/step - loss: 0.5223 - accuracy: 0.7
387 - val_loss: 0.6116 - val_accuracy: 0.6886
Epoch 78/200
579/579 [==============================] - 4s 6ms/step - loss: 0.5221 - accuracy: 0.7
391 - val_loss: 0.6259 - val_accuracy: 0.6898
Epoch 79/200
579/579 [==============================] - 4s 7ms/step - loss: 0.5214 - accuracy: 0.7
386 - val_loss: 0.6360 - val_accuracy: 0.6941
Epoch 80/200
579/579 [==============================] - 4s 7ms/step - loss: 0.5200 - accuracy: 0.7
388 - val_loss: 0.6357 - val_accuracy: 0.6849
```

```
Epoch 81/200
579/579 [==============================] - 4s 6ms/step - loss: 0.5194 - accuracy: 0.7
410 - val_loss: 0.6264 - val_accuracy: 0.6930
Epoch 82/200
579/579 [==============================] - 3s 6ms/step - loss: 0.5184 - accuracy: 0.7
408 - val_loss: 0.6416 - val_accuracy: 0.6913
Epoch 83/200
579/579 [==============================] - 4s 7ms/step - loss: 0.5171 - accuracy: 0.7
418 - val_loss: 0.6181 - val_accuracy: 0.6953
Epoch 84/200
579/579 [==============================] - 3s 6ms/step - loss: 0.5163 - accuracy: 0.7
429 - val_loss: 0.6613 - val_accuracy: 0.6942
Epoch 85/200
579/579 [==============================] - 3s 5ms/step - loss: 0.5174 - accuracy: 0.7
416 - val_loss: 0.6526 - val_accuracy: 0.6736
Epoch 86/200
579/579 [==============================] - 3s 5ms/step - loss: 0.5152 - accuracy: 0.7
424 - val_loss: 0.6841 - val_accuracy: 0.6948
Epoch 87/200
579/579 [==============================] - 3s 5ms/step - loss: 0.5144 - accuracy: 0.7
427 - val_loss: 0.6405 - val_accuracy: 0.6949
Epoch 88/200
579/579 [==============================] - 4s 7ms/step - loss: 0.5143 - accuracy: 0.7
444 - val_loss: 0.6386 - val_accuracy: 0.6914
Epoch 89/200
579/579 [==============================] - 3s 6ms/step - loss: 0.5137 - accuracy: 0.7
442 - val_loss: 0.6429 - val_accuracy: 0.6939
Epoch 90/200
579/579 [==============================] - 3s 5ms/step - loss: 0.5132 - accuracy: 0.7
446 - val_loss: 0.6552 - val_accuracy: 0.6909
Epoch 91/200
579/579 [==============================] - 3s 6ms/step - loss: 0.5125 - accuracy: 0.7
454 - val_loss: 0.6683 - val_accuracy: 0.6894
Epoch 92/200
579/579 [==============================] - 4s 6ms/step - loss: 0.5102 - accuracy: 0.7
458 - val_loss: 0.6415 - val_accuracy: 0.6856
Epoch 93/200
579/579 [==============================] - 4s 7ms/step - loss: 0.5107 - accuracy: 0.7
466 - val_loss: 0.6358 - val_accuracy: 0.6878
Epoch 94/200
579/579 [==============================] - 3s 6ms/step - loss: 0.5108 - accuracy: 0.7
461 - val_loss: 0.6503 - val_accuracy: 0.6838
Epoch 95/200
579/579 [==============================] - 3s 5ms/step - loss: 0.5099 - accuracy: 0.7
465 - val_loss: 0.6491 - val_accuracy: 0.6905
Epoch 96/200
579/579 [==============================] - 3s 6ms/step - loss: 0.5098 - accuracy: 0.7
466 - val_loss: 0.6357 - val_accuracy: 0.6885
Epoch 97/200
579/579 [==============================] - 3s 6ms/step - loss: 0.5099 - accuracy: 0.7
464 - val_loss: 0.6402 - val_accuracy: 0.6909
Epoch 98/200
579/579 [==============================] - 3s 5ms/step - loss: 0.5096 - accuracy: 0.7
457 - val_loss: 0.7094 - val_accuracy: 0.6931
Epoch 99/200
579/579 [==============================] - 3s 6ms/step - loss: 0.5088 - accuracy: 0.7
467 - val_loss: 0.6359 - val_accuracy: 0.6936
Epoch 100/200
579/579 [==============================] - 4s 7ms/step - loss: 0.5082 - accuracy: 0.7
468 - val_loss: 0.6426 - val_accuracy: 0.6899
```

```
Epoch 101/200
579/579 [==============================] - 3s 5ms/step - loss: 0.5073 - accuracy: 0.7
478 - val_loss: 0.6485 - val_accuracy: 0.6880
Epoch 102/200
579/579 [==============================] - 3s 5ms/step - loss: 0.5077 - accuracy: 0.7
478 - val_loss: 0.6312 - val_accuracy: 0.6831
Epoch 103/200
579/579 [==============================] - 3s 5ms/step - loss: 0.5052 - accuracy: 0.7
483 - val_loss: 0.6314 - val_accuracy: 0.6862
Epoch 104/200
579/579 [==============================] - 3s 6ms/step - loss: 0.5059 - accuracy: 0.7
476 - val_loss: 0.6655 - val_accuracy: 0.6941
Epoch 105/200
579/579 [==============================] - 3s 6ms/step - loss: 0.5052 - accuracy: 0.7
496 - val_loss: 0.6454 - val_accuracy: 0.6811
Epoch 106/200
579/579 [==============================] - 4s 6ms/step - loss: 0.5048 - accuracy: 0.7
500 - val_loss: 0.6300 - val_accuracy: 0.6787
Epoch 107/200
579/579 [==============================] - 4s 7ms/step - loss: 0.5050 - accuracy: 0.7
500 - val_loss: 0.6396 - val_accuracy: 0.6838
Epoch 108/200
579/579 [==============================] - 3s 6ms/step - loss: 0.5051 - accuracy: 0.7
496 - val_loss: 0.6831 - val_accuracy: 0.6891
Epoch 109/200
579/579 [==============================] - 3s 6ms/step - loss: 0.5041 - accuracy: 0.7
497 - val_loss: 0.6513 - val_accuracy: 0.6751
Epoch 110/200
579/579 [==============================] - 3s 5ms/step - loss: 0.5025 - accuracy: 0.7
499 - val_loss: 0.6903 - val_accuracy: 0.6896
Epoch 111/200
579/579 [==============================] - 4s 6ms/step - loss: 0.5034 - accuracy: 0.7
504 - val_loss: 0.6676 - val_accuracy: 0.6863
Epoch 112/200
579/579 [==============================] - 3s 6ms/step - loss: 0.5024 - accuracy: 0.7
516 - val_loss: 0.6775 - val_accuracy: 0.6870
Epoch 113/200
579/579 [==============================] - 3s 6ms/step - loss: 0.5003 - accuracy: 0.7
520 - val_loss: 0.6580 - val_accuracy: 0.6793
Epoch 114/200
579/579 [==============================] - 3s 6ms/step - loss: 0.5018 - accuracy: 0.7
512 - val_loss: 0.6711 - val_accuracy: 0.6885
Epoch 115/200
579/579 [==============================] - 3s 6ms/step - loss: 0.5004 - accuracy: 0.7
507 - val_loss: 0.6423 - val_accuracy: 0.6873
Epoch 116/200
579/579 [==============================] - 3s 6ms/step - loss: 0.4998 - accuracy: 0.7
528 - val_loss: 0.6454 - val_accuracy: 0.6911
Epoch 117/200
579/579 [==============================] - 3s 6ms/step - loss: 0.4984 - accuracy: 0.7
537 - val_loss: 0.6350 - val_accuracy: 0.6881
Epoch 118/200
579/579 [==============================] - 3s 6ms/step - loss: 0.4977 - accuracy: 0.7
539 - val_loss: 0.7087 - val_accuracy: 0.6863
Epoch 119/200
579/579 [==============================] - 4s 6ms/step - loss: 0.4981 - accuracy: 0.7
529 - val_loss: 0.6549 - val_accuracy: 0.6821
Epoch 120/200
579/579 [==============================] - 4s 7ms/step - loss: 0.4979 - accuracy: 0.7
528 - val_loss: 0.6625 - val_accuracy: 0.6835
```

```
Epoch 121/200
579/579 [==============================] - 4s 6ms/step - loss: 0.4976 - accuracy: 0.7
540 - val_loss: 0.6623 - val_accuracy: 0.6829
Epoch 122/200
579/579 [==============================] - 4s 7ms/step - loss: 0.4982 - accuracy: 0.7
549 - val_loss: 0.6618 - val_accuracy: 0.6829
Epoch 123/200
579/579 [==============================] - 3s 6ms/step - loss: 0.4962 - accuracy: 0.7
556 - val_loss: 0.6641 - val_accuracy: 0.6875
Epoch 124/200
579/579 [==============================] - 3s 5ms/step - loss: 0.4956 - accuracy: 0.7
562 - val_loss: 0.6575 - val_accuracy: 0.6881
Epoch 125/200
579/579 [==============================] - 3s 5ms/step - loss: 0.4948 - accuracy: 0.7
557 - val_loss: 0.6458 - val_accuracy: 0.6786
Epoch 126/200
579/579 [==============================] - 3s 5ms/step - loss: 0.4957 - accuracy: 0.7
562 - val_loss: 0.7289 - val_accuracy: 0.6829
Epoch 127/200
579/579 [==============================] - 3s 6ms/step - loss: 0.4940 - accuracy: 0.7
565 - val_loss: 0.6636 - val_accuracy: 0.6858
Epoch 128/200
579/579 [==============================] - 3s 6ms/step - loss: 0.4940 - accuracy: 0.7
575 - val_loss: 0.6910 - val_accuracy: 0.6881
Epoch 129/200
579/579 [==============================] - 3s 5ms/step - loss: 0.4949 - accuracy: 0.7
567 - val_loss: 0.6678 - val_accuracy: 0.6700
Epoch 130/200
579/579 [==============================] - 3s 5ms/step - loss: 0.4935 - accuracy: 0.7
559 - val_loss: 0.6746 - val_accuracy: 0.6870
Epoch 131/200
579/579 [==============================] - 3s 5ms/step - loss: 0.4930 - accuracy: 0.7
557 - val_loss: 0.6860 - val_accuracy: 0.6866
Epoch 132/200
579/579 [==============================] - 4s 7ms/step - loss: 0.4915 - accuracy: 0.7
578 - val_loss: 0.6862 - val_accuracy: 0.6823
Epoch 133/200
579/579 [==============================] - 4s 6ms/step - loss: 0.4917 - accuracy: 0.7
568 - val_loss: 0.7250 - val_accuracy: 0.6861
Epoch 134/200
579/579 [==============================] - 4s 6ms/step - loss: 0.4921 - accuracy: 0.7
570 - val_loss: 0.7559 - val_accuracy: 0.6868
Epoch 135/200
579/579 [==============================] - 4s 6ms/step - loss: 0.4887 - accuracy: 0.7
592 - val_loss: 0.7465 - val_accuracy: 0.6867
Epoch 136/200
579/579 [==============================] - 3s 6ms/step - loss: 0.4894 - accuracy: 0.7
600 - val_loss: 0.6555 - val_accuracy: 0.6825
Epoch 137/200
579/579 [==============================] - 3s 5ms/step - loss: 0.4897 - accuracy: 0.7
585 - val_loss: 0.7244 - val_accuracy: 0.6844
Epoch 138/200
579/579 [==============================] - 3s 5ms/step - loss: 0.4892 - accuracy: 0.7
586 - val_loss: 0.6946 - val_accuracy: 0.6795
Epoch 139/200
579/579 [==============================] - 3s 5ms/step - loss: 0.4881 - accuracy: 0.7
598 - val_loss: 0.7030 - val_accuracy: 0.6855
Epoch 140/200
579/579 [==============================] - 3s 5ms/step - loss: 0.4872 - accuracy: 0.7
605 - val_loss: 0.7492 - val_accuracy: 0.6831
```

```
Epoch 141/200
579/579 [==============================] - 3s 6ms/step - loss: 0.4862 - accuracy: 0.7
609 - val_loss: 0.6975 - val_accuracy: 0.6804
Epoch 142/200
579/579 [==============================] - 4s 6ms/step - loss: 0.4872 - accuracy: 0.7
596 - val_loss: 0.6703 - val_accuracy: 0.6832
Epoch 143/200
579/579 [==============================] - 3s 6ms/step - loss: 0.4869 - accuracy: 0.7
606 - val_loss: 0.6623 - val_accuracy: 0.6837
Epoch 144/200
579/579 [==============================] - 3s 5ms/step - loss: 0.4869 - accuracy: 0.7
608 - val_loss: 0.6599 - val_accuracy: 0.6776
Epoch 145/200
579/579 [==============================] - 3s 6ms/step - loss: 0.4859 - accuracy: 0.7
598 - val_loss: 0.6933 - val_accuracy: 0.6894
Epoch 146/200
579/579 [==============================] - 4s 6ms/step - loss: 0.4835 - accuracy: 0.7
619 - val_loss: 0.6975 - val_accuracy: 0.6865
Epoch 147/200
579/579 [==============================] - 4s 7ms/step - loss: 0.4851 - accuracy: 0.7
625 - val_loss: 0.8026 - val_accuracy: 0.6838
Epoch 148/200
579/579 [==============================] - 3s 6ms/step - loss: 0.4831 - accuracy: 0.7
624 - val_loss: 0.6937 - val_accuracy: 0.6874
Epoch 149/200
579/579 [==============================] - 4s 6ms/step - loss: 0.4833 - accuracy: 0.7
620 - val_loss: 0.6593 - val_accuracy: 0.6758
Epoch 150/200
579/579 [==============================] - 4s 6ms/step - loss: 0.4828 - accuracy: 0.7
630 - val_loss: 0.6772 - val_accuracy: 0.6846
Epoch 151/200
579/579 [==============================] - 4s 7ms/step - loss: 0.4826 - accuracy: 0.7
636 - val_loss: 0.7401 - val_accuracy: 0.6900
Epoch 152/200
579/579 [==============================] - 4s 6ms/step - loss: 0.4833 - accuracy: 0.7
628 - val_loss: 0.6849 - val_accuracy: 0.6819
Epoch 153/200
579/579 [==============================] - 3s 6ms/step - loss: 0.4821 - accuracy: 0.7
632 - val_loss: 0.6894 - val_accuracy: 0.6758
Epoch 154/200
579/579 [==============================] - 4s 6ms/step - loss: 0.4821 - accuracy: 0.7
635 - val_loss: 0.7058 - val_accuracy: 0.6838
Epoch 155/200
579/579 [==============================] - 3s 6ms/step - loss: 0.4821 - accuracy: 0.7
636 - val_loss: 0.7230 - val_accuracy: 0.6906
Epoch 156/200
579/579 [==============================] - 4s 6ms/step - loss: 0.4795 - accuracy: 0.7
650 - val_loss: 0.6674 - val_accuracy: 0.6808
Epoch 157/200
579/579 [==============================] - 3s 6ms/step - loss: 0.4781 - accuracy: 0.7
653 - val_loss: 0.6921 - val_accuracy: 0.6868
Epoch 158/200
579/579 [==============================] - 3s 6ms/step - loss: 0.4793 - accuracy: 0.7
650 - val_loss: 0.7081 - val_accuracy: 0.6849
Epoch 159/200
579/579 [==============================] - 3s 6ms/step - loss: 0.4787 - accuracy: 0.7
630 - val_loss: 0.7057 - val_accuracy: 0.6844
Epoch 160/200
579/579 [==============================] - 3s 6ms/step - loss: 0.4781 - accuracy: 0.7
661 - val_loss: 0.7360 - val_accuracy: 0.6851
```

```
Epoch 161/200
579/579 [==============================] - 4s 6ms/step - loss: 0.4788 - accuracy: 0.7
647 - val_loss: 0.6684 - val_accuracy: 0.6845
Epoch 162/200
579/579 [==============================] - 3s 6ms/step - loss: 0.4775 - accuracy: 0.7
653 - val_loss: 0.6652 - val_accuracy: 0.6785
Epoch 163/200
579/579 [==============================] - 4s 7ms/step - loss: 0.4768 - accuracy: 0.7
666 - val_loss: 0.6493 - val_accuracy: 0.6840
Epoch 164/200
579/579 [==============================] - 4s 6ms/step - loss: 0.4759 - accuracy: 0.7
663 - val_loss: 0.7366 - val_accuracy: 0.6867
Epoch 165/200
579/579 [==============================] - 4s 7ms/step - loss: 0.4755 - accuracy: 0.7
662 - val_loss: 0.7412 - val_accuracy: 0.6864
Epoch 166/200
579/579 [==============================] - 4s 7ms/step - loss: 0.4753 - accuracy: 0.7
675 - val_loss: 0.7133 - val_accuracy: 0.6783
Epoch 167/200
579/579 [==============================] - 4s 7ms/step - loss: 0.4738 - accuracy: 0.7
672 - val_loss: 0.7343 - val_accuracy: 0.6703
Epoch 168/200
579/579 [==============================] - 3s 6ms/step - loss: 0.4755 - accuracy: 0.7
662 - val_loss: 0.6686 - val_accuracy: 0.6743
Epoch 169/200
579/579 [==============================] - 3s 6ms/step - loss: 0.4755 - accuracy: 0.7
677 - val_loss: 0.7177 - val_accuracy: 0.6748
Epoch 170/200
579/579 [==============================] - 3s 5ms/step - loss: 0.4727 - accuracy: 0.7
690 - val_loss: 0.7318 - val_accuracy: 0.6852
Epoch 171/200
579/579 [==============================] - 3s 6ms/step - loss: 0.4706 - accuracy: 0.7
689 - val_loss: 0.6903 - val_accuracy: 0.6769
Epoch 172/200
579/579 [==============================] - 4s 6ms/step - loss: 0.4714 - accuracy: 0.7
694 - val_loss: 0.6642 - val_accuracy: 0.6799
Epoch 173/200
579/579 [==============================] - 3s 5ms/step - loss: 0.4707 - accuracy: 0.7
684 - val_loss: 0.7855 - val_accuracy: 0.6804
Epoch 174/200
579/579 [==============================] - 3s 5ms/step - loss: 0.4706 - accuracy: 0.7
697 - val_loss: 0.7113 - val_accuracy: 0.6841
Epoch 175/200
579/579 [==============================] - 3s 6ms/step - loss: 0.4712 - accuracy: 0.7
700 - val_loss: 0.6958 - val_accuracy: 0.6832
Epoch 176/200
579/579 [==============================] - 4s 6ms/step - loss: 0.4686 - accuracy: 0.7
705 - val_loss: 0.7070 - val_accuracy: 0.6838
Epoch 177/200
579/579 [==============================] - 4s 6ms/step - loss: 0.4681 - accuracy: 0.7
719 - val_loss: 0.7353 - val_accuracy: 0.6853
Epoch 178/200
579/579 [==============================] - 4s 6ms/step - loss: 0.4670 - accuracy: 0.7
711 - val_loss: 0.7287 - val_accuracy: 0.6832
Epoch 179/200
579/579 [==============================] - 4s 6ms/step - loss: 0.4666 - accuracy: 0.7
719 - val_loss: 0.6845 - val_accuracy: 0.6823
Epoch 180/200
579/579 [==============================] - 5s 8ms/step - loss: 0.4679 - accuracy: 0.7
712 - val_loss: 0.7036 - val_accuracy: 0.6737
```

```
Epoch 181/200
579/579 [==============================] - 4s 7ms/step - loss: 0.4660 - accuracy: 0.7
727 - val_loss: 0.7144 - val_accuracy: 0.6767
Epoch 182/200
579/579 [==============================] - 4s 7ms/step - loss: 0.4651 - accuracy: 0.7
739 - val_loss: 0.7157 - val_accuracy: 0.6781
Epoch 183/200
579/579 [==============================] - 3s 6ms/step - loss: 0.4648 - accuracy: 0.7
736 - val_loss: 0.7166 - val_accuracy: 0.6690
Epoch 184/200
579/579 [==============================] - 4s 7ms/step - loss: 0.4622 - accuracy: 0.7
757 - val_loss: 0.7558 - val_accuracy: 0.6832
Epoch 185/200
579/579 [==============================] - 4s 7ms/step - loss: 0.4635 - accuracy: 0.7
741 - val_loss: 0.7051 - val_accuracy: 0.6792
Epoch 186/200
579/579 [==============================] - 4s 7ms/step - loss: 0.4623 - accuracy: 0.7
745 - val_loss: 0.7660 - val_accuracy: 0.6741
Epoch 187/200
579/579 [==============================] - 3s 6ms/step - loss: 0.4596 - accuracy: 0.7
759 - val_loss: 0.7396 - val_accuracy: 0.6801
Epoch 188/200
579/579 [==============================] - 3s 5ms/step - loss: 0.4600 - accuracy: 0.7
743 - val_loss: 0.7096 - val_accuracy: 0.6729
Epoch 189/200
579/579 [==============================] - 3s 5ms/step - loss: 0.4617 - accuracy: 0.7
741 - val_loss: 0.7082 - val_accuracy: 0.6750
Epoch 190/200
579/579 [==============================] - 3s 5ms/step - loss: 0.4594 - accuracy: 0.7
766 - val_loss: 0.7342 - val_accuracy: 0.6755
Epoch 191/200
579/579 [==============================] - 3s 5ms/step - loss: 0.4593 - accuracy: 0.7
770 - val_loss: 0.7103 - val_accuracy: 0.6764
Epoch 192/200
579/579 [==============================] - 3s 6ms/step - loss: 0.4609 - accuracy: 0.7
749 - val_loss: 0.7331 - val_accuracy: 0.6734
Epoch 193/200
579/579 [==============================] - 3s 5ms/step - loss: 0.4598 - accuracy: 0.7
733 - val_loss: 0.7384 - val_accuracy: 0.6694
Epoch 194/200
579/579 [==============================] - 3s 5ms/step - loss: 0.4592 - accuracy: 0.7
758 - val_loss: 0.7642 - val_accuracy: 0.6754
Epoch 195/200
579/579 [==============================] - 3s 5ms/step - loss: 0.4561 - accuracy: 0.7
778 - val_loss: 0.7723 - val_accuracy: 0.6786
Epoch 196/200
579/579 [==============================] - 3s 6ms/step - loss: 0.4567 - accuracy: 0.7
773 - val_loss: 0.7621 - val_accuracy: 0.6711
Epoch 197/200
579/579 [==============================] - 3s 6ms/step - loss: 0.4584 - accuracy: 0.7
767 - val_loss: 0.7593 - val_accuracy: 0.6751
Epoch 198/200
579/579 [==============================] - 4s 6ms/step - loss: 0.4576 - accuracy: 0.7
764 - val_loss: 0.8344 - val_accuracy: 0.6752
Epoch 199/200
579/579 [==============================] - 4s 7ms/step - loss: 0.4565 - accuracy: 0.7
780 - val_loss: 0.7926 - val_accuracy: 0.6841
Epoch 200/200
579/579 [==============================] - 4s 7ms/step - loss: 0.4551 - accuracy: 0.7
790 - val_loss: 0.7596 - val_accuracy: 0.6709
```

In [161…

```
drawPlot()
```

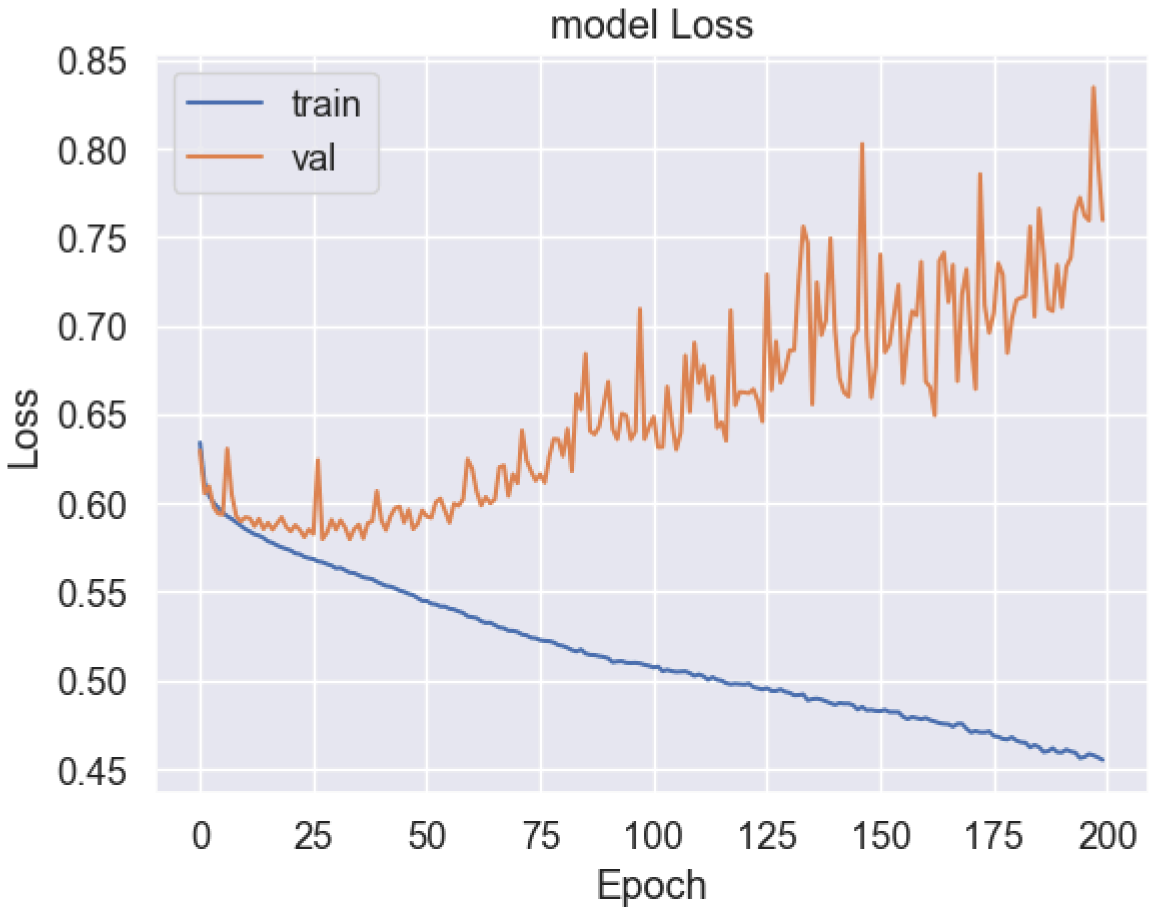

In [72]:

```
# Concatenate Train and Validation Data
X_training = np.concatenate((X_train, X_val), axis=0)
y_training = np.concatenate((y_train, y_val), axis=0)
```

In [75]:

```
# Re-train network on complete Training Data, with Epochs set at 45
network.fit(X_training, y_training,
            batch_size=128, epochs=45)
```

```
Epoch 1/45
771/771 [==============================] - 5s 5ms/step - loss: 0.6300 - accuracy: 0.6
644
Epoch 2/45
771/771 [==============================] - 3s 4ms/step - loss: 0.6086 - accuracy: 0.6
826
Epoch 3/45
771/771 [==============================] - 4s 5ms/step - loss: 0.6026 - accuracy: 0.6
855
Epoch 4/45
771/771 [==============================] - 4s 5ms/step - loss: 0.5982 - accuracy: 0.6
871
Epoch 5/45
771/771 [==============================] - 4s 5ms/step - loss: 0.5954 - accuracy: 0.6
884
Epoch 6/45
771/771 [==============================] - 4s 5ms/step - loss: 0.5927 - accuracy: 0.6
895
Epoch 7/45
771/771 [==============================] - 3s 4ms/step - loss: 0.5905 - accuracy: 0.6
915
Epoch 8/45
771/771 [==============================] - 4s 5ms/step - loss: 0.5888 - accuracy: 0.6
917
Epoch 9/45
771/771 [==============================] - 3s 4ms/step - loss: 0.5872 - accuracy: 0.6
935
Epoch 10/45
771/771 [==============================] - 4s 5ms/step - loss: 0.5857 - accuracy: 0.6
943
Epoch 11/45
771/771 [==============================] - 3s 4ms/step - loss: 0.5840 - accuracy: 0.6
942
Epoch 12/45
771/771 [==============================] - 3s 4ms/step - loss: 0.5822 - accuracy: 0.6
962
Epoch 13/45
771/771 [==============================] - 3s 4ms/step - loss: 0.5810 - accuracy: 0.6
980
Epoch 14/45
771/771 [==============================] - 3s 4ms/step - loss: 0.5790 - accuracy: 0.6
982
Epoch 15/45
771/771 [==============================] - 3s 4ms/step - loss: 0.5781 - accuracy: 0.6
999
Epoch 16/45
771/771 [==============================] - 3s 4ms/step - loss: 0.5771 - accuracy: 0.7
000
Epoch 17/45
771/771 [==============================] - 3s 4ms/step - loss: 0.5758 - accuracy: 0.7
012
Epoch 18/45
771/771 [==============================] - 3s 4ms/step - loss: 0.5752 - accuracy: 0.7
018
Epoch 19/45
771/771 [==============================] - 3s 4ms/step - loss: 0.5738 - accuracy: 0.7
029
Epoch 20/45
771/771 [==============================] - 4s 5ms/step - loss: 0.5730 - accuracy: 0.7
026
```

```
Epoch 21/45
771/771 [==============================] - 4s 5ms/step - loss: 0.5722 - accuracy: 0.7
025
Epoch 22/45
771/771 [==============================] - 3s 4ms/step - loss: 0.5717 - accuracy: 0.7
031
Epoch 23/45
771/771 [==============================] - 3s 3ms/step - loss: 0.5704 - accuracy: 0.7
050
Epoch 24/45
771/771 [==============================] - 3s 4ms/step - loss: 0.5698 - accuracy: 0.7
050
Epoch 25/45
771/771 [==============================] - 3s 4ms/step - loss: 0.5692 - accuracy: 0.7
052
Epoch 26/45
771/771 [==============================] - 3s 3ms/step - loss: 0.5681 - accuracy: 0.7
059
Epoch 27/45
771/771 [==============================] - 3s 4ms/step - loss: 0.5678 - accuracy: 0.7
060
Epoch 28/45
771/771 [==============================] - 3s 4ms/step - loss: 0.5675 - accuracy: 0.7
066
Epoch 29/45
771/771 [==============================] - 3s 4ms/step - loss: 0.5661 - accuracy: 0.7
079
Epoch 30/45
771/771 [==============================] - 3s 4ms/step - loss: 0.5655 - accuracy: 0.7
077
Epoch 31/45
771/771 [==============================] - 3s 4ms/step - loss: 0.5647 - accuracy: 0.7
090
Epoch 32/45
771/771 [==============================] - 4s 5ms/step - loss: 0.5644 - accuracy: 0.7
087
Epoch 33/45
771/771 [==============================] - 4s 5ms/step - loss: 0.5636 - accuracy: 0.7
089
Epoch 34/45
771/771 [==============================] - 4s 5ms/step - loss: 0.5632 - accuracy: 0.7
097
Epoch 35/45
771/771 [==============================] - 4s 5ms/step - loss: 0.5622 - accuracy: 0.7
117
Epoch 36/45
771/771 [==============================] - 4s 5ms/step - loss: 0.5616 - accuracy: 0.7
116
Epoch 37/45
771/771 [==============================] - 3s 4ms/step - loss: 0.5612 - accuracy: 0.7
122
Epoch 38/45
771/771 [==============================] - 3s 4ms/step - loss: 0.5602 - accuracy: 0.7
122
Epoch 39/45
771/771 [==============================] - 3s 4ms/step - loss: 0.5600 - accuracy: 0.7
125
Epoch 40/45
771/771 [==============================] - 3s 4ms/step - loss: 0.5589 - accuracy: 0.7
128
```

```
Epoch 41/45
771/771 [==============================] - 3s 4ms/step - loss: 0.5588 - accuracy: 0.7
130
Epoch 42/45
771/771 [==============================] - 3s 4ms/step - loss: 0.5572 - accuracy: 0.7
149
Epoch 43/45
771/771 [==============================] - 3s 4ms/step - loss: 0.5570 - accuracy: 0.7
151
Epoch 44/45
771/771 [==============================] - 3s 4ms/step - loss: 0.5562 - accuracy: 0.7
141
Epoch 45/45
771/771 [==============================] - 4s 5ms/step - loss: 0.5558 - accuracy: 0.7
143
```

Out[75]:

```
<keras.src.callbacks.History at 0x1ce7e143890>
```

In [76]:

```
y_prediction = network.predict(X_test)
```

```
771/771 [==============================] - 1s 1ms/step
```

In [77]:

```
network.evaluate(X_test, y_test)
```

```
771/771 [==============================] - 1s 2ms/step - loss: 0.5789 - accuracy: 0.7
010
```

Out[77]:

```
[0.5788931846618652, 0.7009565234184265]
```

In [78]:

```
y_prediction[y_prediction >= 0.5] = 1
y_prediction[y_prediction < 0.5] = 0
```

In [79]:

```
nnDF= pd.DataFrame(y_prediction)
```

In [80]:

```
nnDF.value_counts()
```

Out[80]:

```
0.0    12373
1.0    12299
Name: count, dtype: int64
```

In [81]:

```
plotCM(y_test, y_prediction)
```

```
accuracy Score is: 0.7009565499351491
```

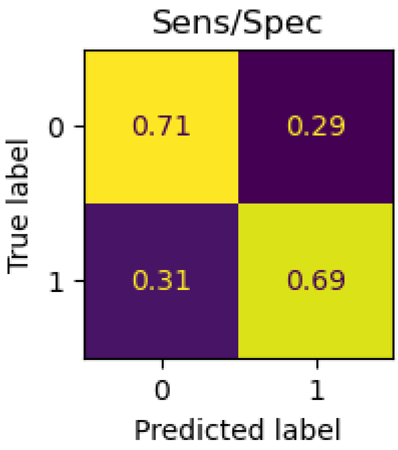

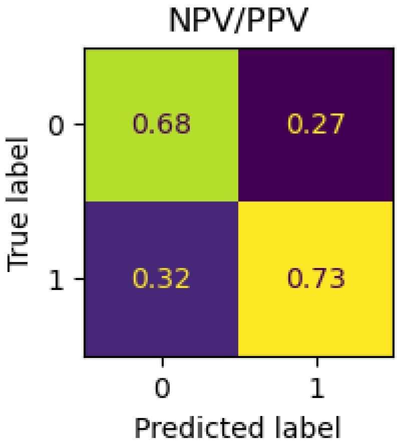
NPV/PPV
0.68
0.27
0.32
0.73
True label
0
1
0
1
Predicted label

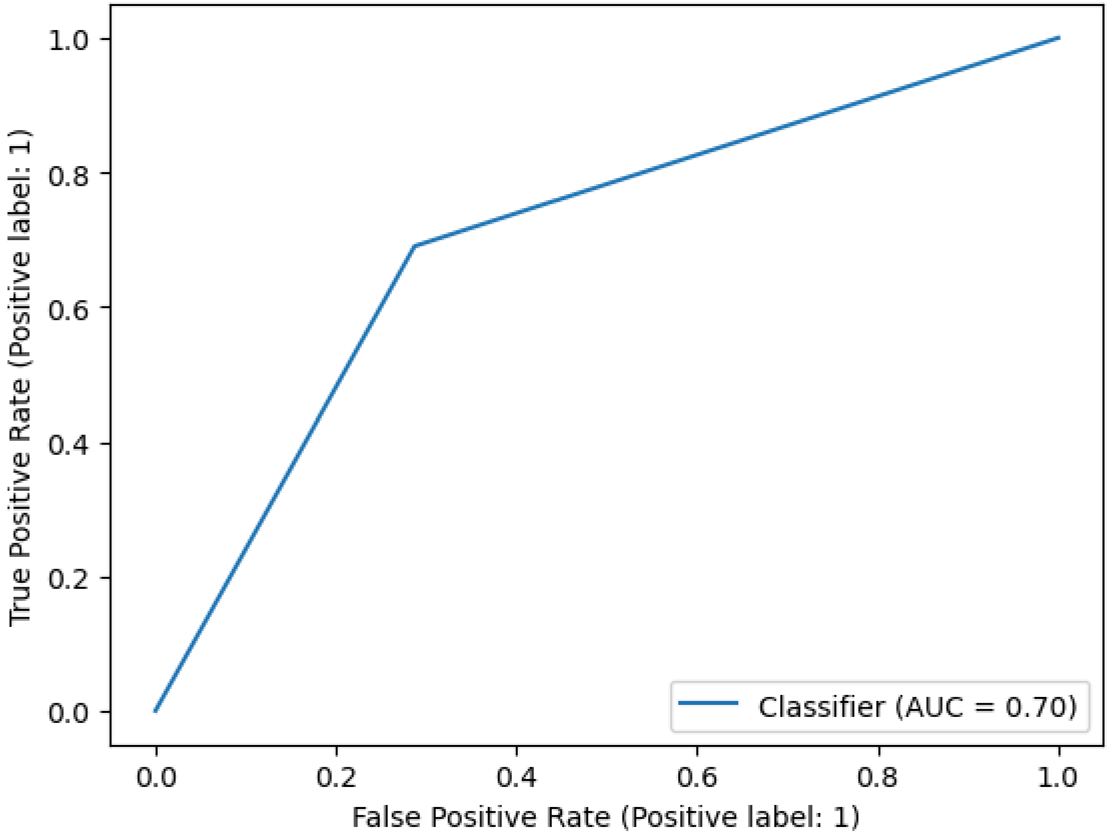
1.0
0.8
0.6
0.4
0.2
0.0
True Positive Rate (Positive label: 1)
Classifier (AUC = 0.70)
0.0
0.2
0.4
0.6
0.8
1.0
False Positive Rate (Positive label: 1)

```
In [ ]:
```

Reimport files and re-process for 48 hour threshold modeling

In [5]:
```
df5 = pd.read_csv('/Users/ganatrh/Downloads/DataWithPrimaryDiagnosis2.csv')
df70 = pd.read_csv('/Users/ganatrh/Downloads/PIM.csv')
```

In [6]:
```
df5 = df5.merge(df70, on='Case Index Id', how='left' )
```

In [7]:
```
df5.info()
```

```
<class 'pandas.core.frame.DataFrame'>
RangeIndex: 608512 entries, 0 to 608511
Data columns (total 78 columns):
 #   Column                                          Non-Null Count   Dtype
---  ------                                          --------------   -----
 0   Unit Id                                         608512 non-null  int64
 1   Case Id                                         608512 non-null  int64
 2   Case Index Id                                   608512 non-null  object
 3   Discharge Year                                  608512 non-null  int64
 4   Is Readmission                                  608512 non-null  int64
 5   Age                                             608512 non-null  object
 6   Weight (kg)                                     608512 non-null  float64
 7   Flag - Age/Weight                               608512 non-null  int64
 8   Collects height                                 608512 non-null  int64
 9   Height (cm)                                     309701 non-null  float64
 10  Gender                                          608512 non-null  object
 11  Collects Race                                   608512 non-null  int64
 12  Race                                            550714 non-null  object
 13  Patient Origin                                  608512 non-null  object
 14  Trauma                                          608512 non-null  int64
 15  Patient Type                                    608512 non-null  object
 16  Collects Transport Team                         608512 non-null  int64
 17  Transport Team                                  371934 non-null  object
 18  Collects Transport Vehicle                      608512 non-null  int64
 19  Transport Vehicle                               347485 non-null  object
 20  Post Operative                                  608512 non-null  int64
 21  Collects Baseline PCPC/POPC                     608512 non-null  int64
 22  Baseline PCPC                                   127543 non-null  object
 23  Baseline POPC                                   127464 non-null  object
 24  Collects FSS                                    608512 non-null  int64
 25  Baseline FSS                                    59273 non-null   float64
 26  Cardiac Patient                                 608512 non-null  int64
 27  Cardiac Procedure directly prior to or during stay  608512 non-null  int64
 28  Medical Length of Stay (days)                   584327 non-null  float64
 29  Physical Length of Stay (days)                  608512 non-null  float64
 30  Hospital LOS                                    606176 non-null  float64
 31  Outcome                                         608512 non-null  object
 32  Disposition                                     608512 non-null  object
 33  Collects Limitation on Care                     608512 non-null  int64
 34  Limitation on Care                              523209 non-null  float64
 35  Collects Brain Dead                             608512 non-null  int64
 36  Is Brain Dead                                   4922 non-null    float64
 37  Collects Altered Code                           608512 non-null  int64
 38  Is Altered Code                                 523227 non-null  float64
 39  Collects Withdrawal of Support                  608512 non-null  int64
 40  Withdrawal of Support                           497357 non-null  float64
 41  Collects Autopsy                                608512 non-null  int64
 42  Was Autopsy Performed                           11602 non-null   object
 43  Collects Organ Donation                         608512 non-null  int64
 44  Is Organ Donor                                  2572 non-null    float64
 45  Collects Tissue Donation                        608512 non-null  int64
 46  Is Tissue Donor                                 3443 non-null    float64
 47  Collects Donation after Cardiac Death           608512 non-null  int64
 48  Donation after Cardiac Death                    3563 non-null    float64
 49  Discharge PCPC                                  123029 non-null  object
 50  Discharge POPC                                  122965 non-null  object
 51  Discharge FSS                                   58085 non-null   float64
 52  Hospital Outcome                                604949 non-null  object
 53  Collects Diagnosis STS Codes                    608512 non-null  int64
 54  Collects Diagnosis ICD-9 Codes                  608512 non-null  int64
```

```
 55  PIM 3 Score                                            608512 non-null  float64
 56  PIM 3 Probability of Death                             608512 non-null  float64
 57  Mechanical Ventilation (First Hour)                    608511 non-null  float64
 58  Elective Admission to ICU                              608511 non-null  float64
 59  PIM 3 Recovery from Surgery                            608511 non-null  object
 60  Category                                               608475 non-null  object
 61  Systolic Blood Pressure                                588367 non-null  float64
 62  Systolic Blood Pressure Unknown                        608511 non-null  float64
 63  PaO2 (mmHg)                                            13722 non-null   float64
 64  PaO2 (kPa)                                             13722 non-null   float64
 65  PaO2 Unknown                                           608511 non-null  float64
 66  FiO2                                                   13722 non-null   float64
 67  FiO2 Unknown                                           608511 non-null  float64
 68  Base Excess                                            51063 non-null   float64
 69  Base Excess Unknown                                    608511 non-null  float64
 70  PIM 3 Pupillary Reaction                               608511 non-null  object
 71  PIM 3 Pupillary Reaction Unknown                       608511 non-null  float64
 72  Admitted following Cardiac Bypass - PIM2/PIM3          608511 non-null  float64
 73  Admitted following Cardiac Bypass - PIM3 Baseline      608511 non-null  float64
 74  Admitted following Cardiac Bypass - PIM3 PostIndex     166 non-null     float64
 75  PIM 3 - No Very High Risk Dx                           608512 non-null  int64
 76  PIM 3 - No High Risk Dx                                608512 non-null  int64
 77  PIM 3 - No Low Risk Dx                                 608512 non-null  int64
dtypes: float64(31), int64(28), object(19)
memory usage: 362.1+ MB
```

Remove rows that have flagged inaccurate weights

In [8]:

```
df6 = SmartDataframe(df5, config={"llm": llm})
df7 = df6.chat("Remove rows that have the value '1' in column 'Flag - Age/Weight' and
df7.info()
```

```
<class 'pandas.core.frame.DataFrame'>
RangeIndex: 607901 entries, 0 to 607900
Data columns (total 78 columns):
 #   Column                                            Non-Null Count   Dtype  
---  ------                                            --------------   -----  
 0   Unit Id                                           607901 non-null  int64  
 1   Case Id                                           607901 non-null  int64  
 2   Case Index Id                                     607901 non-null  object 
 3   Discharge Year                                    607901 non-null  int64  
 4   Is Readmission                                    607901 non-null  int64  
 5   Age                                               607901 non-null  object 
 6   Weight (kg)                                       607901 non-null  float64
 7   Flag - Age/Weight                                 607901 non-null  int64  
 8   Collects height                                   607901 non-null  int64  
 9   Height (cm)                                       309407 non-null  float64
 10  Gender                                            607901 non-null  object 
 11  Collects Race                                     607901 non-null  int64  
 12  Race                                              550155 non-null  object 
 13  Patient Origin                                    607901 non-null  object 
 14  Trauma                                            607901 non-null  int64  
 15  Patient Type                                      607901 non-null  object 
 16  Collects Transport Team                           607901 non-null  int64  
 17  Transport Team                                    371523 non-null  object 
 18  Collects Transport Vehicle                        607901 non-null  int64  
 19  Transport Vehicle                                 347101 non-null  object 
 20  Post Operative                                    607901 non-null  int64  
 21  Collects Baseline PCPC/POPC                       607901 non-null  int64  
 22  Baseline PCPC                                     127387 non-null  object 
 23  Baseline POPC                                     127309 non-null  object 
 24  Collects FSS                                      607901 non-null  int64  
 25  Baseline FSS                                      59184 non-null   float64
 26  Cardiac Patient                                   607901 non-null  int64  
 27  Cardiac Procedure directly prior to or during stay  607901 non-null  int64  
 28  Medical Length of Stay (days)                     583736 non-null  float64
 29  Physical Length of Stay (days)                    607901 non-null  float64
 30  Hospital LOS                                      605567 non-null  float64
 31  Outcome                                           607901 non-null  object 
 32  Disposition                                       607901 non-null  object 
 33  Collects Limitation on Care                       607901 non-null  int64  
 34  Limitation on Care                                522675 non-null  float64
 35  Collects Brain Dead                               607901 non-null  int64  
 36  Is Brain Dead                                     4918 non-null    float64
 37  Collects Altered Code                             607901 non-null  int64  
 38  Is Altered Code                                   522693 non-null  float64
 39  Collects Withdrawal of Support                    607901 non-null  int64  
 40  Withdrawal of Support                             496859 non-null  float64
 41  Collects Autopsy                                  607901 non-null  int64  
 42  Was Autopsy Performed                             11595 non-null   object 
 43  Collects Organ Donation                           607901 non-null  int64  
 44  Is Organ Donor                                    2572 non-null    float64
 45  Collects Tissue Donation                          607901 non-null  int64  
 46  Is Tissue Donor                                   3442 non-null    float64
 47  Collects Donation after Cardiac Death             607901 non-null  int64  
 48  Donation after Cardiac Death                      3562 non-null    float64
 49  Discharge PCPC                                    122879 non-null  object 
 50  Discharge POPC                                    122815 non-null  object 
 51  Discharge FSS                                     58000 non-null   float64
 52  Hospital Outcome                                  604344 non-null  object 
 53  Collects Diagnosis STS Codes                      607901 non-null  int64  
 54  Collects Diagnosis ICD-9 Codes                    607901 non-null  int64  
```

```
 55  PIM 3 Score                                             607901 non-null  float64
 56  PIM 3 Probability of Death                              607901 non-null  float64
 57  Mechanical Ventilation (First Hour)                     607900 non-null  float64
 58  Elective Admission to ICU                               607900 non-null  float64
 59  PIM 3 Recovery from Surgery                             607900 non-null  object
 60  Category                                                607864 non-null  object
 61  Systolic Blood Pressure                                 587779 non-null  float64
 62  Systolic Blood Pressure Unknown                         607900 non-null  float64
 63  PaO2 (mmHg)                                             13708 non-null   float64
 64  PaO2 (kPa)                                              13708 non-null   float64
 65  PaO2 Unknown                                            607900 non-null  float64
 66  FiO2                                                    13708 non-null   float64
 67  FiO2 Unknown                                            607900 non-null  float64
 68  Base Excess                                             50991 non-null   float64
 69  Base Excess Unknown                                     607900 non-null  float64
 70  PIM 3 Pupillary Reaction                                607900 non-null  object
 71  PIM 3 Pupillary Reaction Unknown                        607900 non-null  float64
 72  Admitted following Cardiac Bypass - PIM2/PIM3           607900 non-null  float64
 73  Admitted following Cardiac Bypass - PIM3 Baseline       607900 non-null  float64
 74  Admitted following Cardiac Bypass - PIM3 PostIndex      166 non-null     float64
 75  PIM 3 - No Very High Risk Dx                            607901 non-null  int64
 76  PIM 3 - No High Risk Dx                                 607901 non-null  int64
 77  PIM 3 - No Low Risk Dx                                  607901 non-null  int64
dtypes: float64(31), int64(28), object(19)
memory usage: 361.8+ MB
```

Create dataframe with Columns of interest

In [9]:
```
df8 = SmartDataframe(df7, config={"llm": llm})
df9 = df8.chat("Select the columns 'Case Index Id', 'Is Readmission', 'Age', 'Weight (
```

In [10]:
```
df9.info()
```

```
<class 'pandas.core.frame.DataFrame'>
RangeIndex: 607901 entries, 0 to 607900
Data columns (total 41 columns):
 #   Column                                             Non-Null Count   Dtype  
---  ------                                             --------------   -----  
 0   Case Index Id                                      607901 non-null  object 
 1   Is Readmission                                     607901 non-null  int64  
 2   Age                                                607901 non-null  object 
 3   Weight (kg)                                        607901 non-null  float64
 4   Gender                                             607901 non-null  object 
 5   Race                                               550155 non-null  object 
 6   Patient Origin                                     607901 non-null  object 
 7   Trauma                                             607901 non-null  int64  
 8   Patient Type                                       607901 non-null  object 
 9   Transport Team                                     371523 non-null  object 
 10  Transport Vehicle                                  347101 non-null  object 
 11  Post Operative                                     607901 non-null  int64  
 12  Baseline PCPC                                      127387 non-null  object 
 13  Baseline POPC                                      127309 non-null  object 
 14  Baseline FSS                                       59184 non-null   float64
 15  Cardiac Patient                                    607901 non-null  int64  
 16  Cardiac Procedure directly prior to or during stay 607901 non-null  int64  
 17  Medical Length of Stay (days)                      583736 non-null  float64
 18  Physical Length of Stay (days)                     607901 non-null  float64
 19  Hospital LOS                                       605567 non-null  float64
 20  Outcome                                            607901 non-null  object 
 21  Disposition                                        607901 non-null  object 
 22  Is Altered Code                                    522693 non-null  float64
 23  Discharge PCPC                                     122879 non-null  object 
 24  Discharge POPC                                     122815 non-null  object 
 25  Discharge FSS                                      58000 non-null   float64
 26  Hospital Outcome                                   604344 non-null  object 
 27  PIM 3 Score                                        607901 non-null  float64
 28  PIM 3 Probability of Death                         607901 non-null  float64
 29  Mechanical Ventilation (First Hour)                607900 non-null  float64
 30  Elective Admission to ICU                          607900 non-null  float64
 31  PIM 3 Recovery from Surgery                        607900 non-null  object 
 32  Systolic Blood Pressure                            587779 non-null  float64
 33  PaO2 (mmHg)                                        13708 non-null   float64
 34  FiO2                                               13708 non-null   float64
 35  Base Excess                                        50991 non-null   float64
 36  PIM 3 Pupillary Reaction                           607900 non-null  object 
 37  PIM 3 - No Very High Risk Dx                       607901 non-null  int64  
 38  PIM 3 - No High Risk Dx                            607901 non-null  int64  
 39  PIM 3 - No Low Risk Dx                             607901 non-null  int64  
 40  Category                                           607864 non-null  object 
dtypes: float64(15), int64(8), object(18)
memory usage: 190.2+ MB
```

Remove rows that have NaN for "elective ICU admission" and for "POPC/PCPC". This comcomitantly removes NaN for other variables too

In [11]:
```
df9.dropna(subset=['Elective Admission to ICU', 'Baseline POPC', 'Baseline PCPC', 'Sys
df9.info()
```

```
<class 'pandas.core.frame.DataFrame'>
RangeIndex: 123485 entries, 0 to 123484
Data columns (total 41 columns):
 #   Column                                                Non-Null Count   Dtype  
---  ------                                                --------------   -----  
 0   Case Index Id                                         123485 non-null  object 
 1   Is Readmission                                        123485 non-null  int64  
 2   Age                                                   123485 non-null  object 
 3   Weight (kg)                                           123485 non-null  float64
 4   Gender                                                123485 non-null  object 
 5   Race                                                  121785 non-null  object 
 6   Patient Origin                                        123485 non-null  object 
 7   Trauma                                                123485 non-null  int64  
 8   Patient Type                                          123485 non-null  object 
 9   Transport Team                                        98498 non-null   object 
 10  Transport Vehicle                                     104581 non-null  object 
 11  Post Operative                                        123485 non-null  int64  
 12  Baseline PCPC                                         123485 non-null  object 
 13  Baseline POPC                                         123485 non-null  object 
 14  Baseline FSS                                          16690 non-null   float64
 15  Cardiac Patient                                       123485 non-null  int64  
 16  Cardiac Procedure directly prior to or during stay    123485 non-null  int64  
 17  Medical Length of Stay (days)                         118321 non-null  float64
 18  Physical Length of Stay (days)                        123485 non-null  float64
 19  Hospital LOS                                          123245 non-null  float64
 20  Outcome                                               123485 non-null  object 
 21  Disposition                                           123485 non-null  object 
 22  Is Altered Code                                       122991 non-null  float64
 23  Discharge PCPC                                        118721 non-null  object 
 24  Discharge POPC                                        118695 non-null  object 
 25  Discharge FSS                                         16383 non-null   float64
 26  Hospital Outcome                                      122734 non-null  object 
 27  PIM 3 Score                                           123485 non-null  float64
 28  PIM 3 Probability of Death                            123485 non-null  float64
 29  Mechanical Ventilation (First Hour)                   123485 non-null  float64
 30  Elective Admission to ICU                             123485 non-null  float64
 31  PIM 3 Recovery from Surgery                           123485 non-null  object 
 32  Systolic Blood Pressure                               123485 non-null  float64
 33  PaO2 (mmHg)                                           3205 non-null    float64
 34  FiO2                                                  3205 non-null    float64
 35  Base Excess                                           12155 non-null   float64
 36  PIM 3 Pupillary Reaction                              123485 non-null  object 
 37  PIM 3 - No Very High Risk Dx                          123485 non-null  int64  
 38  PIM 3 - No High Risk Dx                               123485 non-null  int64  
 39  PIM 3 - No Low Risk Dx                                123485 non-null  int64  
 40  Category                                              123485 non-null  object 
dtypes: float64(15), int64(8), object(18)
memory usage: 38.6+ MB
```

In [12]:
```
df9["Base Excess"] = df9["Base Excess"].replace(np.nan, 0)
df9["Base Excess"].value_counts()
```

Out[12]:
```
Base Excess
 0.0     111514
-3.0        358
-4.0        342
-5.0        329
-2.0        327
          ...
-16.5         1
 9.7          1
 23.3         1
-34.1         1
 11.3         1
Name: count, Length: 472, dtype: int64
```

The cells below consolidate all patients > 18 into one group

In [13]:
```python
df9["Age"].value_counts()
```

Out[13]:
```
Age
Infant 29 days to < 2 years                   36705
Adolescent 12 years to < 18 years             32266
Child 2 years to < 6 years                    23946
Child 6 years to < 12 years                   22954
Adolescent (late) 18 years to < 21 years       4241
Neonate Birth to 29 days                       2355
Adult 21 years and up                          1018
Name: count, dtype: int64
```

In [14]:
```python
df10 = df9.copy()
df10.Age.replace(['Adult 21 years and up','Adolescent (late) 18 years to < 21 years'],
```

In [15]:
```python
df10["Age"].value_counts()
```

Out[15]:
```
Age
Infant 29 days to < 2 years          36705
Adolescent 12 years to < 18 years    32266
Child 2 years to < 6 years           23946
Child 6 years to < 12 years          22954
Adult 18 years and up                 5259
Neonate Birth to 29 days              2355
Name: count, dtype: int64
```

The cells below consolidate "Patient origin" into more discrete categories

In [16]:
```python
df10["Patient Origin"].value_counts()
```

Out[16]:

```
Patient Origin
Emergency Department                                                      47563
Another Hospital's Emergency Department                                   21884
General Care Floor                                                        18033
Operating Room (Direct to ICU)                                            17792
Recovery Room (PACU)                                                       9248
Another Hospital's ICU                                                     1796
Another Hospital's General Care Floor                                      1753
Step-Down Unit/Intermediate Care Unit                                      1374
Home                                                                       1120
Physician's Office/Clinic                                                   959
Inpatient Procedure Suite (not cath lab)                                    558
Outpatient Procedure Suite                                                  255
NICU (in this hospital)                                                     229
Another Hospital's OR                                                       205
Other                                                                       151
Another ICU in this hospital (except NICU)                                  129
Transitional Care/Skilled Nursing/Chronic Care Facility                     122
Dedicated technology dependent unit (transitional/progressive care unit)    104
Physical Rehab Center                                                        64
Cath lab                                                                     57
Another hospital's cath lab                                                  17
Another hospital's Step-Down Unit/Intermediate Care Unit                     16
Psychiatric/Substance Abuse/Chemical Dependence Rehab Center                 13
Another Hospital's dedicated home ventilator unit                            13
Delivery Room (including delivery room in another hospital)                  12
Telemetry Unit                                                               10
Pulmonary Rehab Center                                                        7
Another hospital's Telemetry Unit                                             1
Name: count, dtype: int64
```

In [17]:

```
df11 = df10.copy()
df11["Patient Origin"].replace(["Another Hospital's General Care Floor", "Transitional
df11["Patient Origin"].replace(["Another Hospital's ICU", "Another Hospital's OR", "Ar
df11["Patient Origin"].replace(["Home", "Physician's Office/Clinic"], "Home/Clinic",
df11["Patient Origin"].replace(["Inpatient Procedure Suite (not cath lab)", "Outpatier
df11["Patient Origin"].replace(["Step-Down Unit/Intermediate Care Unit", "Telemetry Ur
```

In [18]:

```
df11["Patient Origin"].value_counts()
```

Out[18]:

```
Patient Origin
Emergency Department                                           47563
Another Hospital's Emergency Department                        21884
General Care Floor                                             18033
Operating Room (Direct to ICU)                                 17792
Recovery Room (PACU)                                            9248
Home/Clinic                                                     2079
Another Hospital ICU/OR/Cath                                    2018
Another Hospital Non-ED Non-ICU unit                            1989
Step down/Intermediate/Telemetry                                1488
Procedure Suite                                                  813
NICU (in this hospital)                                          229
Other                                                            151
Another ICU in this hospital (except NICU)                       129
Cath lab                                                          57
Delivery Room (including delivery room in another hospital)       12
Name: count, dtype: int64
```

Remove rows with PIM3 recovery from surgery showing a recovery from cardiac surgery

In [19]:
```
df11["PIM 3 Recovery from Surgery"].value_counts()
```

Out[19]:
```
PIM 3 Recovery from Surgery
No                                                99362
Yes, Recovery from non-cardiac procedure          23992
Yes, Recovery from non-bypass cardiac procedure      98
Yes, Recovery from bypass cardiac procedure          33
Name: count, dtype: int64
```

In [20]:
```
df12 = df11.copy()
df12 = df12.drop(df12[df12['PIM 3 Recovery from Surgery'] == 'Yes, Recovery from non-b
df12 = df12.drop(df12[df12['PIM 3 Recovery from Surgery'] == 'Yes, Recovery from bypas
df12 = df12.reset_index(drop=True)
```

In [21]:
```
df12["PIM 3 Recovery from Surgery"].value_counts()
```

Out[21]:
```
PIM 3 Recovery from Surgery
No                                           99362
Yes, Recovery from non-cardiac procedure     23992
Name: count, dtype: int64
```

Fill Race NaN with "Unspecified"

In [22]:
```
df12.Race.fillna('Unspecified', inplace=True)
df12.Race.unique()
```

Out[22]:
```
array(['White', 'Other/Mixed', 'Hispanic or Latino',
       'Black or African American', 'Asian/Indian/Pacific Islander',
       'Unspecified', 'Asian', 'American Indian or Alaska Native',
       'Native Hawaiian or Other Pacific Islander'], dtype=object)
```

Consolidate Pupillary reactions to Fixed>3mm and others/unknown

In [23]:
```
df12["PIM 3 Pupillary Reaction"].value_counts()
```

Out[23]:
```
PIM 3 Pupillary Reaction
Other                                   116524
Unknown                                   5040
>3mm and both fixed                        927
Pupilary Assessment Invalid - Drugs        651
Pupilary Assessment Invalid - Injury       141
Pupilary Assessment Invalid - Toxins        71
Name: count, dtype: int64
```

In [24]:
```
df13 = df12.copy()
df13["PIM 3 Pupillary Reaction"].replace(["Other", "Unknown", "Pupilary Assessment Inv
df13["PIM 3 Pupillary Reaction"].value_counts()
```

Out[24]:
```
PIM 3 Pupillary Reaction
Other/Unknown          122427
>3mm and both fixed       927
Name: count, dtype: int64
```

In [25]:
```
chatBot = SmartDataframe(df13, config={"llm": llm})
```

In [26]:
```
chatBot.chat("For the column 'Physical Length of Stay (days)', what percentage of rows
```

Out[26]:
```
"The percentage of rows with 'Physical Length of Stay (days)' more than 2 is: 40.17%"
```

In [27]:
```
chatBot.chat("For the column 'Physical Length of Stay (days)', what percentage of rows
```

Out[27]:
```
40.208667736757626
```

In [28]:
```
df14 = chatBot.chat("Create a new column 'LOS', and if the value in column 'Physical L
df14.LOS.value_counts()
```

Out[28]:
```
LOS
0    73807
1    49547
Name: count, dtype: int64
```

In [29]:
```
df14.info()
```

```
<class 'pandas.core.frame.DataFrame'>
RangeIndex: 123354 entries, 0 to 123353
Data columns (total 42 columns):
 #   Column                                           Non-Null Count   Dtype  
---  ------                                           --------------   -----  
 0   Case Index Id                                    123354 non-null  object 
 1   Is Readmission                                   123354 non-null  int64  
 2   Age                                              123354 non-null  object 
 3   Weight (kg)                                      123354 non-null  float64
 4   Gender                                           123354 non-null  object 
 5   Race                                             123354 non-null  object 
 6   Patient Origin                                   123354 non-null  object 
 7   Trauma                                           123354 non-null  int64  
 8   Patient Type                                     123354 non-null  object 
 9   Transport Team                                   98391 non-null   object 
 10  Transport Vehicle                                104468 non-null  object 
 11  Post Operative                                   123354 non-null  int64  
 12  Baseline PCPC                                    123354 non-null  object 
 13  Baseline POPC                                    123354 non-null  object 
 14  Baseline FSS                                     16666 non-null   float64
 15  Cardiac Patient                                  123354 non-null  int64  
 16  Cardiac Procedure directly prior to or during stay 123354 non-null  int64  
 17  Medical Length of Stay (days)                    118191 non-null  float64
 18  Physical Length of Stay (days)                   123354 non-null  float64
 19  Hospital LOS                                     123115 non-null  float64
 20  Outcome                                          123354 non-null  object 
 21  Disposition                                      123354 non-null  object 
 22  Is Altered Code                                  122862 non-null  float64
 23  Discharge PCPC                                   118592 non-null  object 
 24  Discharge POPC                                   118566 non-null  object 
 25  Discharge FSS                                    16359 non-null   float64
 26  Hospital Outcome                                 122609 non-null  object 
 27  PIM 3 Score                                      123354 non-null  float64
 28  PIM 3 Probability of Death                       123354 non-null  float64
 29  Mechanical Ventilation (First Hour)              123354 non-null  float64
 30  Elective Admission to ICU                        123354 non-null  float64
 31  PIM 3 Recovery from Surgery                      123354 non-null  object 
 32  Systolic Blood Pressure                          123354 non-null  float64
 33  PaO2 (mmHg)                                      3179 non-null    float64
 34  FiO2                                             3179 non-null    float64
 35  Base Excess                                      123354 non-null  float64
 36  PIM 3 Pupillary Reaction                         123354 non-null  object 
 37  PIM 3 - No Very High Risk Dx                     123354 non-null  int64  
 38  PIM 3 - No High Risk Dx                          123354 non-null  int64  
 39  PIM 3 - No Low Risk Dx                           123354 non-null  int64  
 40  Category                                         123354 non-null  object 
 41  LOS                                              123354 non-null  int64  
dtypes: float64(15), int64(9), object(18)
memory usage: 39.5+ MB
```

Apply Ordinal Encoder to convert Object datatype to Integers

In [30]:

```
toEncode = df14[['Is Readmission', 'Age', 'Gender', 'Race', 'Patient Origin', 'Trauma'
                'Post Operative', 'Mechanical Ventilation (First Hour)', 'Elective Adm
                'PIM 3 Recovery from Surgery', 'Category', 'Baseline PCPC', 'Baseline 
                 'PIM 3 Pupillary Reaction']].copy()
columnNames = list(toEncode.columns)

enc = OrdinalEncoder()
```

```
df15 = pd.DataFrame(enc.fit_transform(toEncode), columns= columnNames)

for i in range(len(columnNames)):
    print (columnNames[i] , enc.categories_[i])
```

```
Is Readmission [0 1]
Age ['Adolescent 12 years to < 18 years' 'Adult 18 years and up'
 'Child 2 years to < 6 years' 'Child 6 years to < 12 years'
 'Infant 29 days to < 2 years' 'Neonate Birth to 29 days']
Gender ['Ambiguous' 'Female' 'Male']
Race ['American Indian or Alaska Native' 'Asian'
 'Asian/Indian/Pacific Islander' 'Black or African American'
 'Hispanic or Latino' 'Native Hawaiian or Other Pacific Islander'
 'Other/Mixed' 'Unspecified' 'White']
Patient Origin ['Another Hospital ICU/OR/Cath' 'Another Hospital Non-ED Non-ICU unit'
 "Another Hospital's Emergency Department"
 'Another ICU in this hospital (except NICU)' 'Cath lab'
 'Delivery Room (including delivery room in another hospital)'
 'Emergency Department' 'General Care Floor' 'Home/Clinic'
 'NICU (in this hospital)' 'Operating Room (Direct to ICU)' 'Other'
 'Procedure Suite' 'Recovery Room (PACU)'
 'Step down/Intermediate/Telemetry']
Trauma [0 1]
Patient Type ['Scheduled (> or = 12 Hours in Advance)' 'Unscheduled']
Post Operative [0 1]
Mechanical Ventilation (First Hour) [0. 1.]
Elective Admission to ICU [0. 1.]
PIM 3 Recovery from Surgery ['No' 'Yes, Recovery from non-cardiac procedure']
Category ['Cardiovascular' 'Dermatologic' 'Endocrine' 'Factors Influencing Health'
 'Gastrointestinal' 'Genetic' 'Gynecologic' 'Hematologic' 'Immunologic'
 'Infectious' 'Injury/Poisoning/Adverse Effects' 'Metabolic' 'Neurologic'
 'Newborn/Perinatal' 'Oncologic' 'Ophthalmologic' 'Orthopedic'
 'Psychiatric' 'Renal/Genitourinary' 'Respiratory' 'Respiratory/ENT'
 'Rheumatologic' 'Symptoms' 'Transplant' 'Ungroupable']
Baseline PCPC ['1 - Normal' '2 - Mild disability' '3 - Moderate disability'
 '4 - Severe disability' '5 - Coma or vegetative state']
Baseline POPC ['1 - Good overall performance' '2 - Mild overall disability'
 '3 - Moderate overall disability' '4 - Severe overall disability'
 '5 - Coma or vegetative state']
PIM 3 Pupillary Reaction ['>3mm and both fixed' 'Other/Unknown']
```

In [31]:
```
df15.info()
```

```
<class 'pandas.core.frame.DataFrame'>
RangeIndex: 123354 entries, 0 to 123353
Data columns (total 15 columns):
 #   Column                               Non-Null Count   Dtype  
---  ------                               --------------   -----  
 0   Is Readmission                       123354 non-null  float64
 1   Age                                  123354 non-null  float64
 2   Gender                               123354 non-null  float64
 3   Race                                 123354 non-null  float64
 4   Patient Origin                       123354 non-null  float64
 5   Trauma                               123354 non-null  float64
 6   Patient Type                         123354 non-null  float64
 7   Post Operative                       123354 non-null  float64
 8   Mechanical Ventilation (First Hour)  123354 non-null  float64
 9   Elective Admission to ICU            123354 non-null  float64
 10  PIM 3 Recovery from Surgery          123354 non-null  float64
 11  Category                             123354 non-null  float64
 12  Baseline PCPC                        123354 non-null  float64
 13  Baseline POPC                        123354 non-null  float64
 14  PIM 3 Pupillary Reaction             123354 non-null  float64
dtypes: float64(15)
memory usage: 14.1 MB
```

Add "weight" and "PIM3" Score into the dataframe

In [32]:

```
df16 = df15.copy()

df16.insert(2, "Weight", 0)
df16.insert(15, "PIM3", 0)
df16.insert(16, "PIM 3 Probability of Death", 0)
df16.insert(17, "Systolic Blood Pressure", 0)
df16.insert(18, "Base Excess", 0)
df16.insert(20, "PIM 3 - No Very High Risk Dx", 0)
df16.insert(21, "PIM 3 - No High Risk Dx", 0)
df16.insert(22, "PIM 3 - No Low Risk Dx", 0)




df16.Weight = df14["Weight (kg)"].copy()
df16.PIM3 = df14["PIM 3 Score"].copy()
df16["PIM 3 Probability of Death"] = df14["PIM 3 Probability of Death"].copy()
df16["Systolic Blood Pressure"] = df14["Systolic Blood Pressure"].copy()
df16["Base Excess"] = df14["Base Excess"].copy()
df16["PIM 3 - No Very High Risk Dx"] = df14["PIM 3 - No Very High Risk Dx"].copy()
df16["PIM 3 - No High Risk Dx"] = df14["PIM 3 - No High Risk Dx"].copy()
df16["PIM 3 - No Low Risk Dx"] = df14["PIM 3 - No Low Risk Dx"].copy()


df16.info()
```

```
<class 'pandas.core.frame.DataFrame'>
RangeIndex: 123354 entries, 0 to 123353
Data columns (total 23 columns):
 #   Column                              Non-Null Count   Dtype  
---  ------                              --------------   -----  
 0   Is Readmission                      123354 non-null  float64
 1   Age                                 123354 non-null  float64
 2   Weight                              123354 non-null  float64
 3   Gender                              123354 non-null  float64
 4   Race                                123354 non-null  float64
 5   Patient Origin                      123354 non-null  float64
 6   Trauma                              123354 non-null  float64
 7   Patient Type                        123354 non-null  float64
 8   Post Operative                      123354 non-null  float64
 9   Mechanical Ventilation (First Hour) 123354 non-null  float64
 10  Elective Admission to ICU           123354 non-null  float64
 11  PIM 3 Recovery from Surgery         123354 non-null  float64
 12  Category                            123354 non-null  float64
 13  Baseline PCPC                       123354 non-null  float64
 14  Baseline POPC                       123354 non-null  float64
 15  PIM3                                123354 non-null  float64
 16  PIM 3 Probability of Death          123354 non-null  float64
 17  Systolic Blood Pressure             123354 non-null  float64
 18  Base Excess                         123354 non-null  float64
 19  PIM 3 Pupillary Reaction            123354 non-null  float64
 20  PIM 3 - No Very High Risk Dx        123354 non-null  int64  
 21  PIM 3 - No High Risk Dx             123354 non-null  int64  
 22  PIM 3 - No Low Risk Dx              123354 non-null  int64  
dtypes: float64(20), int64(3)
memory usage: 21.6 MB
```

Add "LOS" into the dataframe and generate HEATMAP

In [33]:

```
df17 = df16.copy()

df17.insert(23, "LOS", 0)
df17.LOS = df14.LOS.copy()
```

In [34]:

```
df17.info()
```

```
<class 'pandas.core.frame.DataFrame'>
RangeIndex: 123354 entries, 0 to 123353
Data columns (total 24 columns):
 #   Column                                Non-Null Count   Dtype  
---  ------                                --------------   -----  
 0   Is Readmission                        123354 non-null  float64
 1   Age                                   123354 non-null  float64
 2   Weight                                123354 non-null  float64
 3   Gender                                123354 non-null  float64
 4   Race                                  123354 non-null  float64
 5   Patient Origin                        123354 non-null  float64
 6   Trauma                                123354 non-null  float64
 7   Patient Type                          123354 non-null  float64
 8   Post Operative                        123354 non-null  float64
 9   Mechanical Ventilation (First Hour)   123354 non-null  float64
 10  Elective Admission to ICU             123354 non-null  float64
 11  PIM 3 Recovery from Surgery           123354 non-null  float64
 12  Category                              123354 non-null  float64
 13  Baseline PCPC                         123354 non-null  float64
 14  Baseline POPC                         123354 non-null  float64
 15  PIM3                                  123354 non-null  float64
 16  PIM 3 Probability of Death            123354 non-null  float64
 17  Systolic Blood Pressure               123354 non-null  float64
 18  Base Excess                           123354 non-null  float64
 19  PIM 3 Pupillary Reaction              123354 non-null  float64
 20  PIM 3 - No Very High Risk Dx          123354 non-null  int64  
 21  PIM 3 - No High Risk Dx               123354 non-null  int64  
 22  PIM 3 - No Low Risk Dx                123354 non-null  int64  
 23  LOS                                   123354 non-null  int64  
dtypes: float64(20), int64(4)
memory usage: 22.6 MB
```

In [35]:

```
def heatMap(list, fontSize):
    corr = list.corr()
    corr= corr.round(decimals=2)
    plt.figure(figsize=(20, 8))
    sns.set(font_scale = 1.2)
    sns.heatmap(corr, cmap='RdBu', vmin=-1, vmax=1, annot=True, annot_kws={'fontsize':
```

In [37]:

```
heatMap(df17, 12)
```

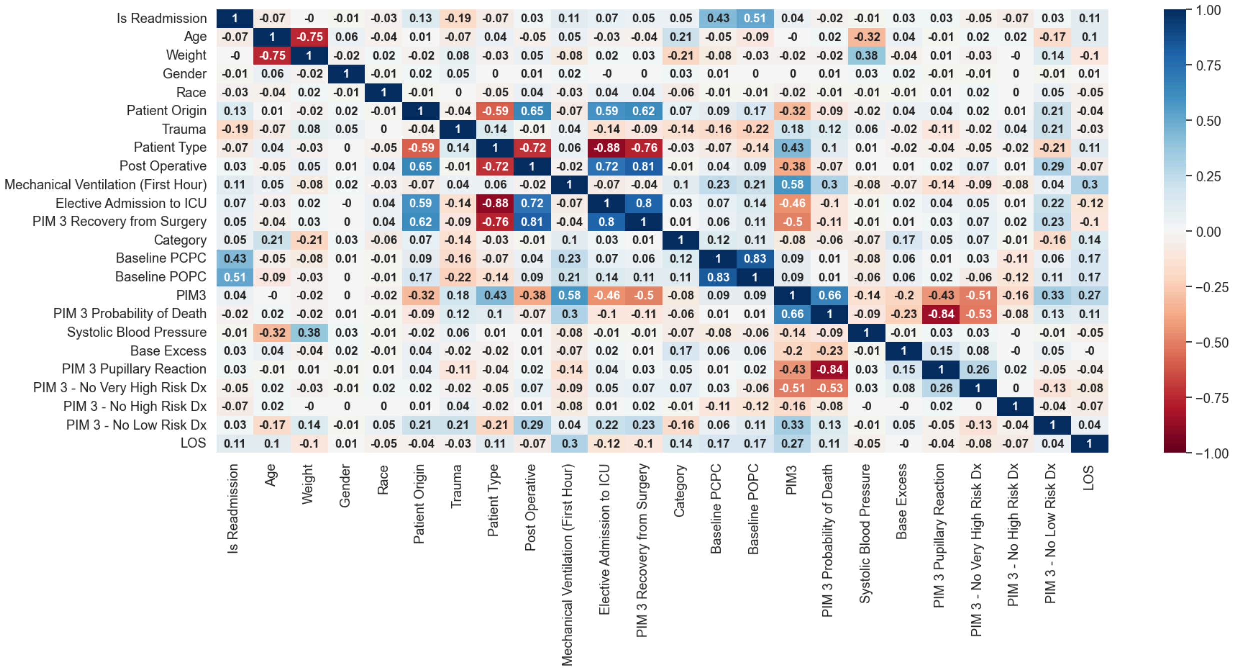

Create INPUT and OUTPUT NP arrays

In [35]:
```
input = df16.copy().to_numpy()
output = df17["LOS"].copy().to_numpy()

#input.head()
print('Input sample 25: \n' , input[27] ,  "\n \n Output sample 25: " , output[27])
```

```
Input sample 25: 
 [  0.      4.     10.8     2.      7.      1.      0.      1.      1.      1.
   0.      0.    625.      1.      2.     -1.06   25.68   89.      0.      1.
   0.      1.      1.   ]

 Output sample 25:  1
```

Apply SKLearn train/test split to Input and Output for 80:20 split

In [36]:
```
X_train, X_test, y_train, y_test = train_test_split(input, output, test_size=0.2, ranc
```

In [37]:
```
print(X_train.shape, "\t", y_train.shape, "\n")
print(X_test.shape, "\t", y_test.shape, "\n")
```

```
(98685, 23)      (98685,)

(24672, 23)      (24672,)
```

Fit MinMaxScaler on Training data, and then apply transformation to training and test set

In [38]:
```
scaler = MinMaxScaler()
scaler.fit(X_train)
```

Out[38]:

```
▾ MinMaxScaler
MinMaxScaler()
```

In [39]:

```
X_train = scaler.transform(X_train)
X_test = scaler.transform(X_test)
```

In [40]:

```
print(X_test[27], '\n' '\n', y_test[27])
```

```
[1.         0.4        0.03930435 0.5        0.75       0.92857143
 0.         0.         1.         1.         1.         1.
 0.31406045 0.75       0.75       0.17475124 0.00410287 0.42918455
 0.38844847 1.         1.         1.         1.        ]

 1
```

Import and apply LinearSVC - results appear Encouraging

Attempted standard SVC with RBF kernel too but computationally exorbitant

In [41]:

```
from sklearn.svm import LinearSVC
```

In [42]:

```
svc = LinearSVC(dual="auto", random_state=0, tol=1e-5)
svc.fit(X_train, y_train)
```

Out[42]:

```
▾ LinearSVC
LinearSVC(dual='auto', random_state=0, tol=1e-05)
```

In [43]:

```
svc_predictions = svc.predict(X_test)
```

In [44]:

```
def plotCM(test, predictions):

    print("accuracy Score is: " + str(accuracy_score(test, predictions)))

    cm = confusion_matrix(test, predictions, normalize='true')
    fig, ax = plt.subplots(figsize=(2,2))
    ConfusionMatrixDisplay(cm).plot(ax=ax, colorbar=False)
    plt.title('Sens/Spec')
    plt.show()

    cm2 = confusion_matrix(test, predictions, normalize='pred')
    fig, ax = plt.subplots(figsize=(2,2))
    ConfusionMatrixDisplay(cm2).plot(ax=ax, colorbar=False)
    plt.title('NPV/PPV')
    plt.show()

    RocCurveDisplay.from_predictions(test, predictions)
    plt.show()
```

In [45]:

```
plotCM(y_test, svc_predictions)
```

```
accuracy Score is: 0.6877837224383917
```

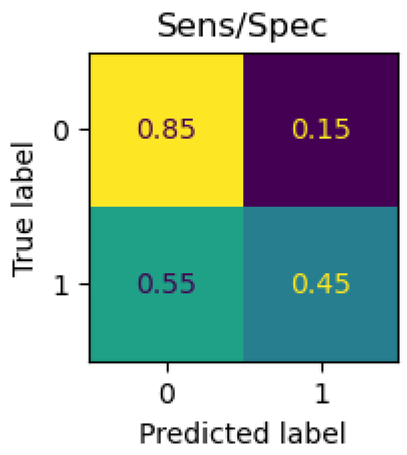

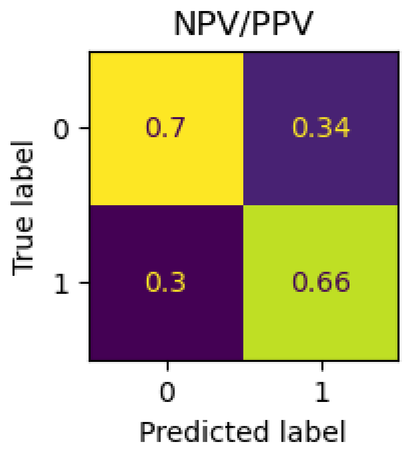

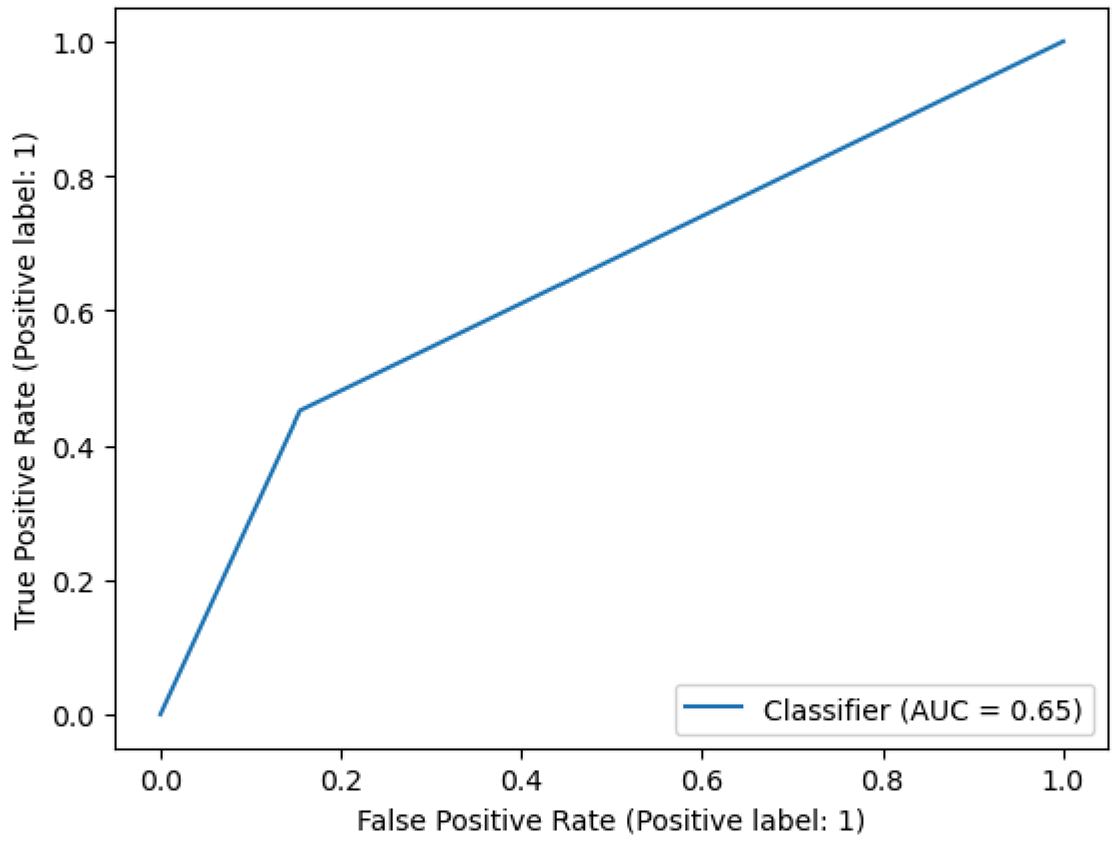

Import and apply Stochastic Gradient Descent Classification - results seen

In [46]:
```
from sklearn.linear_model import SGDClassifier
```

In [47]:
```
sgdc = SGDClassifier(loss="hinge", penalty="l2")
sgdc.fit(X_train, y_train)
```

Out[47]:
```
▾ SGDClassifier
SGDClassifier()
```

In [48]:
```
sgdc_predictions = sgdc.predict(X_test)
```

In [49]:
```
plotCM(y_test, sgdc_predictions)
```

```
accuracy Score is: 0.6802448119325551
```

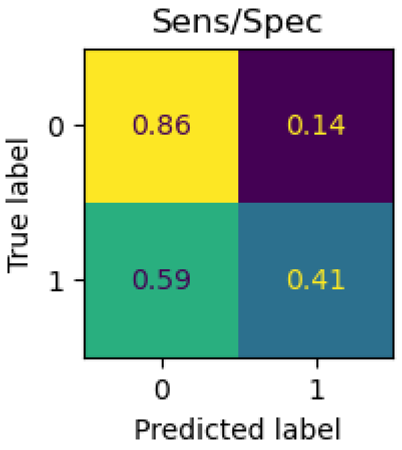

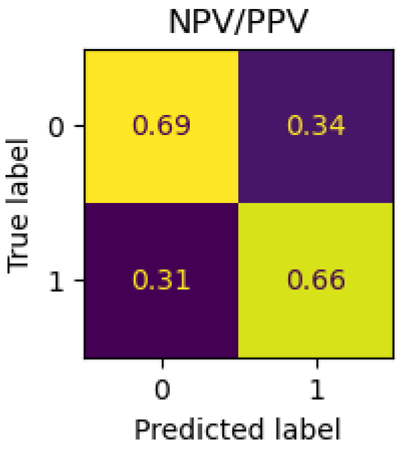

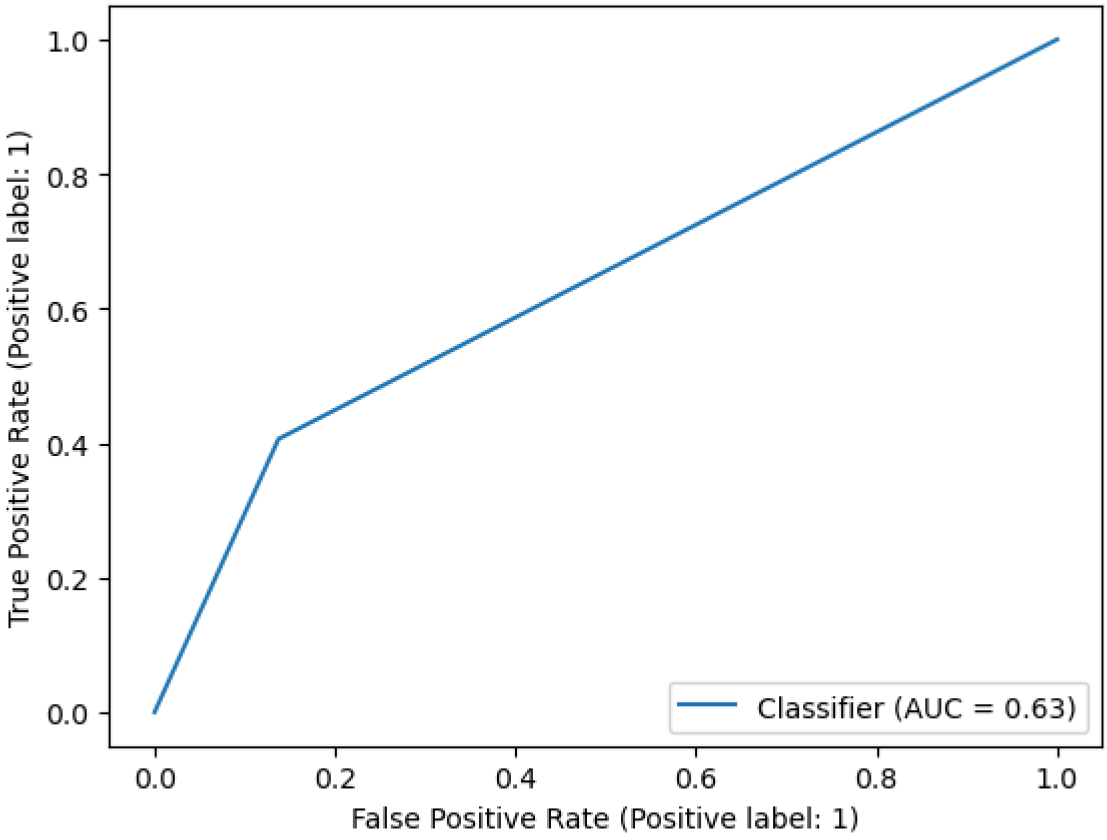

Import and apply KNN Classifier - results

In [50]:
```
from sklearn.neighbors import KNeighborsClassifier
```

In [51]:
```
knn = KNeighborsClassifier(n_neighbors=15)
knn.fit(X_train, y_train)
```

Out[51]:
```
▾ KNeighborsClassifier
KNeighborsClassifier(n_neighbors=15)
```

In [52]:
```
knn_predictions = knn.predict(X_test)
```

In [53]:
```
plotCM(y_test, knn_predictions)
```

```
accuracy Score is: 0.6976329442282749
```

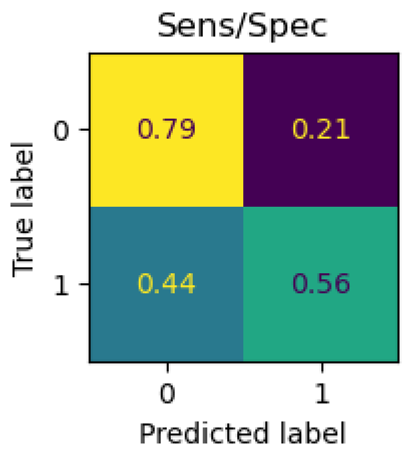

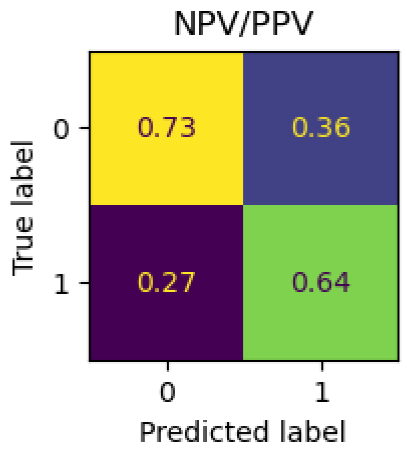

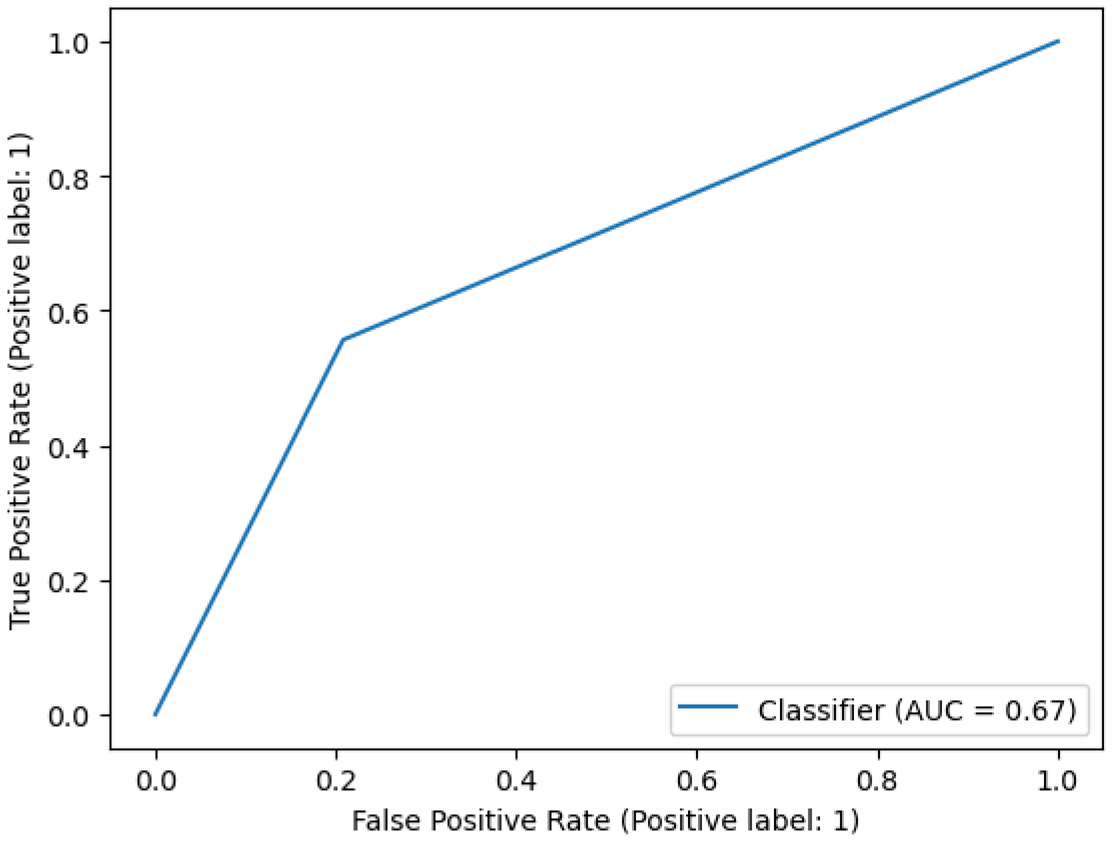

Import and apply Decision Tree Regression - results appear to be GARBAGE

```
In [54]: from sklearn.tree import DecisionTreeClassifier
```

```
In [55]: dtree = DecisionTreeClassifier(random_state=30)
         dtree.fit(X_train, y_train)
```

```
Out[55]: ▾ DecisionTreeClassifier
         DecisionTreeClassifier(random_state=30)
```

```
In [56]: dtree_predictions = dtree.predict(X_test)
```

```
In [57]: plotCM(y_test, dtree_predictions)
```

```
accuracy Score is: 0.6429961089494164
```

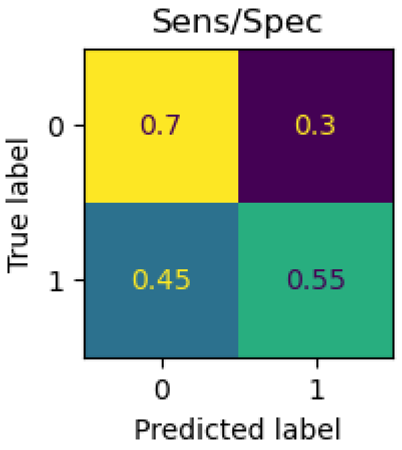

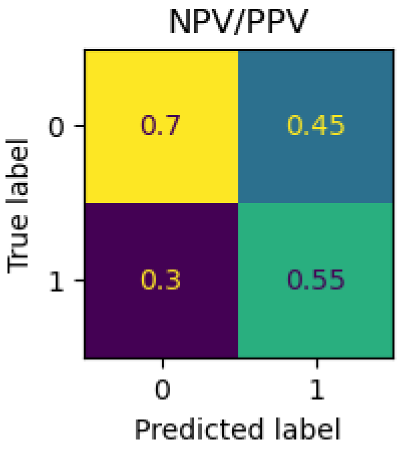

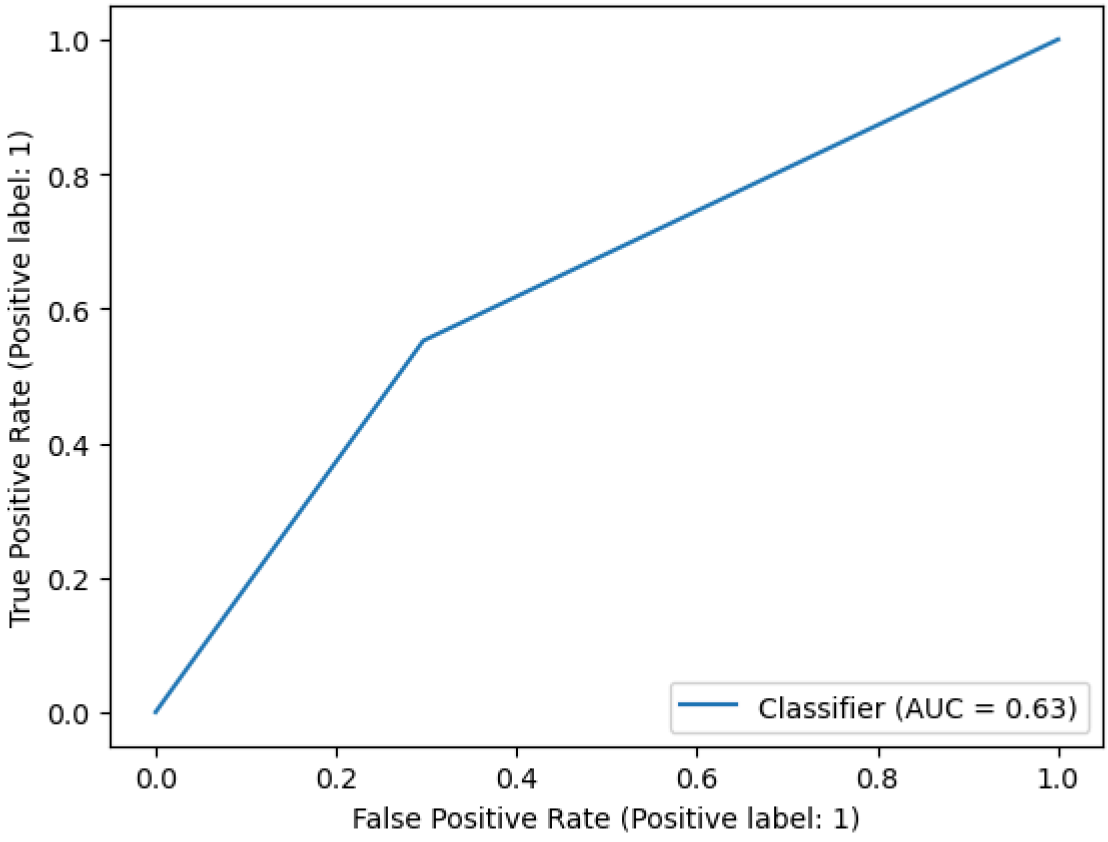

Attempt Gradient Boosting

In [58]:
```
from sklearn.ensemble import HistGradientBoostingClassifier
```

In [59]:
```
boost = HistGradientBoostingClassifier()
boost.fit(X_train, y_train)
```

Out[59]:
```
▾ HistGradientBoostingClassifier
HistGradientBoostingClassifier()
```

In [60]:
```
boost_predictions = boost.predict(X_test)
```

In [61]:
```
plotCM(y_test, boost_predictions)
```

```
accuracy Score is: 0.7314769779507133
```

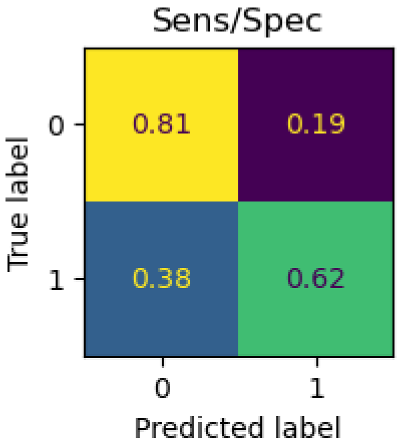

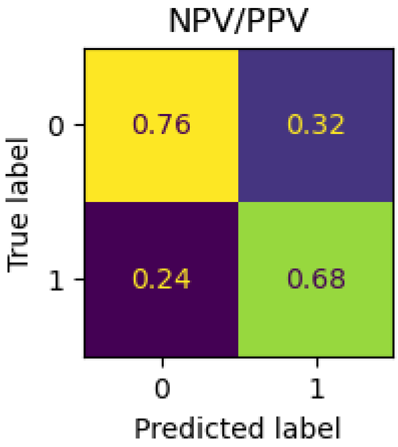

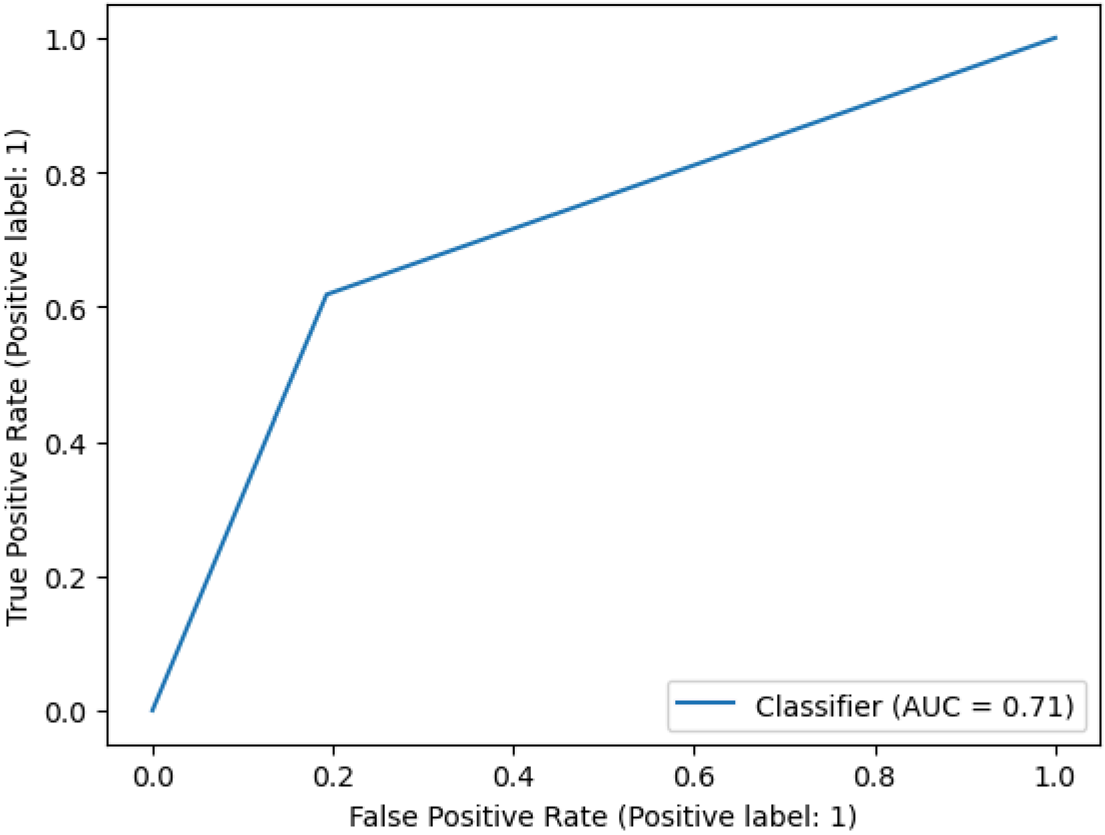

Attempt CatBoost

In [62]:
```
from catboost import CatBoostClassifier
import warnings
warnings.filterwarnings("ignore")
```

In [63]:
```
# Define the hyperparameters for the CatBoost algorithm
params = {'learning_rate': 0.1, 'depth': 6,
          'l2_leaf_reg': 3, 'iterations': 100}

# Initialize the CatBoostClassifier object
# with the defined hyperparameters and fit it on the training set
model = CatBoostClassifier(**params)
model.fit(X_train, y_train)
```

```
0:	learn: 0.6714921	total: 181ms	remaining: 18s
1:	learn: 0.6531966	total: 229ms	remaining: 11.2s
2:	learn: 0.6387709	total: 302ms	remaining: 9.77s
3:	learn: 0.6271011	total: 432ms	remaining: 10.4s
4:	learn: 0.6164765	total: 530ms	remaining: 10.1s
5:	learn: 0.6085883	total: 644ms	remaining: 10.1s
6:	learn: 0.6017119	total: 741ms	remaining: 9.85s
7:	learn: 0.5964953	total: 851ms	remaining: 9.79s
8:	learn: 0.5921643	total: 977ms	remaining: 9.88s
9:	learn: 0.5876747	total: 1.1s	remaining: 9.91s
10:	learn: 0.5841864	total: 1.22s	remaining: 9.85s
11:	learn: 0.5811722	total: 1.32s	remaining: 9.7s
12:	learn: 0.5782634	total: 1.42s	remaining: 9.51s
13:	learn: 0.5754080	total: 1.52s	remaining: 9.33s
14:	learn: 0.5733200	total: 1.6s	remaining: 9.06s
15:	learn: 0.5709679	total: 1.69s	remaining: 8.88s
16:	learn: 0.5694622	total: 1.79s	remaining: 8.73s
17:	learn: 0.5682512	total: 1.85s	remaining: 8.42s
18:	learn: 0.5670877	total: 1.91s	remaining: 8.13s
19:	learn: 0.5657159	total: 1.97s	remaining: 7.9s
20:	learn: 0.5647734	total: 2.09s	remaining: 7.86s
21:	learn: 0.5635593	total: 2.19s	remaining: 7.78s
22:	learn: 0.5624361	total: 2.27s	remaining: 7.59s
23:	learn: 0.5614503	total: 2.34s	remaining: 7.4s
24:	learn: 0.5604262	total: 2.4s	remaining: 7.19s
25:	learn: 0.5597100	total: 2.46s	remaining: 7.01s
26:	learn: 0.5590116	total: 2.56s	remaining: 6.92s
27:	learn: 0.5582476	total: 2.65s	remaining: 6.81s
28:	learn: 0.5575242	total: 2.72s	remaining: 6.67s
29:	learn: 0.5570968	total: 2.79s	remaining: 6.51s
30:	learn: 0.5567198	total: 2.89s	remaining: 6.44s
31:	learn: 0.5559320	total: 2.96s	remaining: 6.3s
32:	learn: 0.5551148	total: 3.06s	remaining: 6.22s
33:	learn: 0.5544594	total: 3.19s	remaining: 6.2s
34:	learn: 0.5540047	total: 3.31s	remaining: 6.15s
35:	learn: 0.5534586	total: 3.41s	remaining: 6.07s
36:	learn: 0.5530280	total: 3.51s	remaining: 5.97s
37:	learn: 0.5524786	total: 3.6s	remaining: 5.87s
38:	learn: 0.5520299	total: 3.67s	remaining: 5.74s
39:	learn: 0.5516633	total: 3.75s	remaining: 5.62s
40:	learn: 0.5512666	total: 3.8s	remaining: 5.47s
41:	learn: 0.5508704	total: 3.85s	remaining: 5.32s
42:	learn: 0.5504744	total: 3.93s	remaining: 5.21s
43:	learn: 0.5500400	total: 4s	remaining: 5.09s
44:	learn: 0.5496577	total: 4.09s	remaining: 4.99s
45:	learn: 0.5493962	total: 4.17s	remaining: 4.9s
46:	learn: 0.5490727	total: 4.27s	remaining: 4.81s
47:	learn: 0.5488596	total: 4.33s	remaining: 4.69s
48:	learn: 0.5486029	total: 4.41s	remaining: 4.59s
49:	learn: 0.5484013	total: 4.47s	remaining: 4.47s
50:	learn: 0.5480902	total: 4.53s	remaining: 4.35s
51:	learn: 0.5477076	total: 4.59s	remaining: 4.24s
52:	learn: 0.5473284	total: 4.64s	remaining: 4.12s
53:	learn: 0.5471493	total: 4.7s	remaining: 4.01s
54:	learn: 0.5467517	total: 4.75s	remaining: 3.89s
55:	learn: 0.5466024	total: 4.81s	remaining: 3.78s
56:	learn: 0.5464503	total: 4.88s	remaining: 3.68s
57:	learn: 0.5462642	total: 4.94s	remaining: 3.58s
58:	learn: 0.5461252	total: 5.01s	remaining: 3.48s
59:	learn: 0.5458766	total: 5.08s	remaining: 3.39s
```

```
60:	learn: 0.5457022	total: 5.16s	remaining: 3.3s
61:	learn: 0.5454549	total: 5.25s	remaining: 3.21s
62:	learn: 0.5452231	total: 5.32s	remaining: 3.13s
63:	learn: 0.5449763	total: 5.39s	remaining: 3.03s
64:	learn: 0.5447419	total: 5.47s	remaining: 2.94s
65:	learn: 0.5443393	total: 5.55s	remaining: 2.86s
66:	learn: 0.5441957	total: 5.61s	remaining: 2.76s
67:	learn: 0.5439737	total: 5.67s	remaining: 2.67s
68:	learn: 0.5435989	total: 5.73s	remaining: 2.57s
69:	learn: 0.5434676	total: 5.81s	remaining: 2.49s
70:	learn: 0.5432110	total: 5.89s	remaining: 2.4s
71:	learn: 0.5430272	total: 5.96s	remaining: 2.32s
72:	learn: 0.5427172	total: 6.03s	remaining: 2.23s
73:	learn: 0.5423935	total: 6.11s	remaining: 2.15s
74:	learn: 0.5422376	total: 6.2s	remaining: 2.07s
75:	learn: 0.5419674	total: 6.31s	remaining: 1.99s
76:	learn: 0.5418187	total: 6.4s	remaining: 1.91s
77:	learn: 0.5415827	total: 6.49s	remaining: 1.83s
78:	learn: 0.5414958	total: 6.57s	remaining: 1.75s
79:	learn: 0.5413635	total: 6.65s	remaining: 1.66s
80:	learn: 0.5412559	total: 6.72s	remaining: 1.58s
81:	learn: 0.5411024	total: 6.8s	remaining: 1.49s
82:	learn: 0.5409587	total: 6.86s	remaining: 1.41s
83:	learn: 0.5407624	total: 6.93s	remaining: 1.32s
84:	learn: 0.5406178	total: 7.03s	remaining: 1.24s
85:	learn: 0.5403179	total: 7.13s	remaining: 1.16s
86:	learn: 0.5401469	total: 7.19s	remaining: 1.07s
87:	learn: 0.5399055	total: 7.26s	remaining: 991ms
88:	learn: 0.5398279	total: 7.33s	remaining: 907ms
89:	learn: 0.5397034	total: 7.41s	remaining: 823ms
90:	learn: 0.5395608	total: 7.47s	remaining: 739ms
91:	learn: 0.5393493	total: 7.54s	remaining: 656ms
92:	learn: 0.5391551	total: 7.6s	remaining: 572ms
93:	learn: 0.5390606	total: 7.67s	remaining: 490ms
94:	learn: 0.5388749	total: 7.74s	remaining: 407ms
95:	learn: 0.5386474	total: 7.8s	remaining: 325ms
96:	learn: 0.5385329	total: 7.86s	remaining: 243ms
97:	learn: 0.5383121	total: 7.96s	remaining: 163ms
98:	learn: 0.5382245	total: 8.03s	remaining: 81.1ms
99:	learn: 0.5380336	total: 8.11s	remaining: 0us
```

Out[63]:

```
<catboost.core.CatBoostClassifier at 0x232f5905510>
```

In [64]:

```
y_pred = model.predict(X_test)
```

In [65]:

```
plotCM(y_test, y_pred)
```

```
accuracy Score is: 0.7262078469520103
```

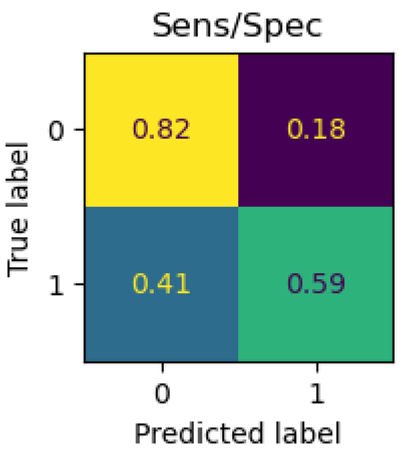

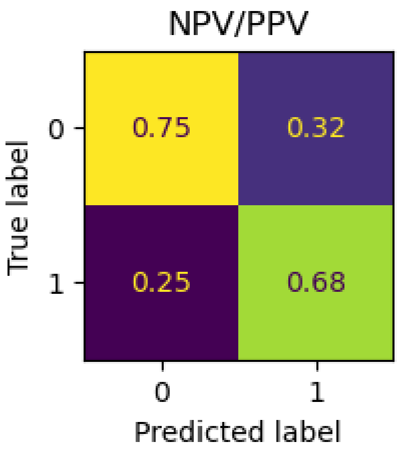

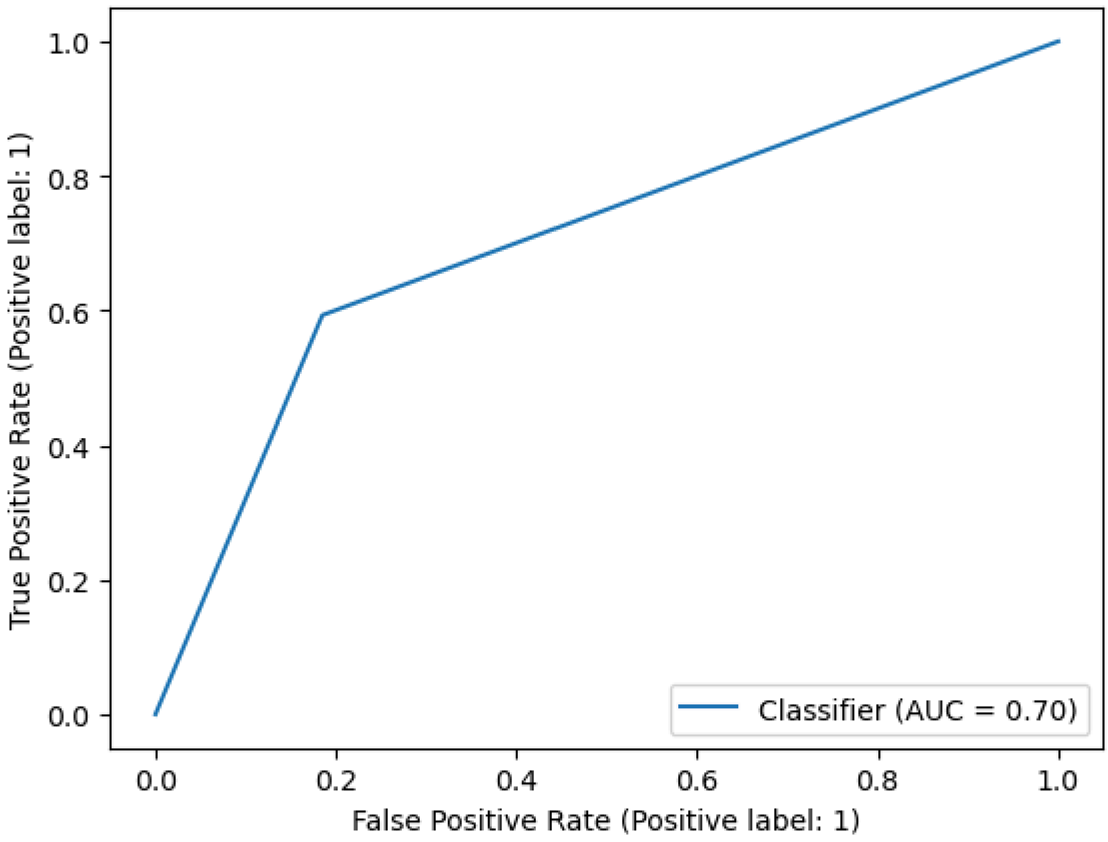

Neural Network

split Training data again in 75:25 ration to generate a total 60:20:20 split for Train:Validate:Test

In [66]:
```
X_train, X_val, y_train, y_val = train_test_split(X_train, y_train, test_size=0.25, ra
```

In [67]:
```
# Define function to plot Training Loss vs Validation Loss across epochs
def drawPlot():
    plt.plot(history.history['loss'])
    plt.plot(history.history['val_loss'])
    plt.title('model Loss')
    plt.ylabel('Loss')
    plt.xlabel('Epoch')
    #plt.xticks(np.arange(0, 21, 1.0))
    plt.legend(['train', 'val'], loc='upper left')
    plt.show()
```

In [73]:
```
drawPlot()
```

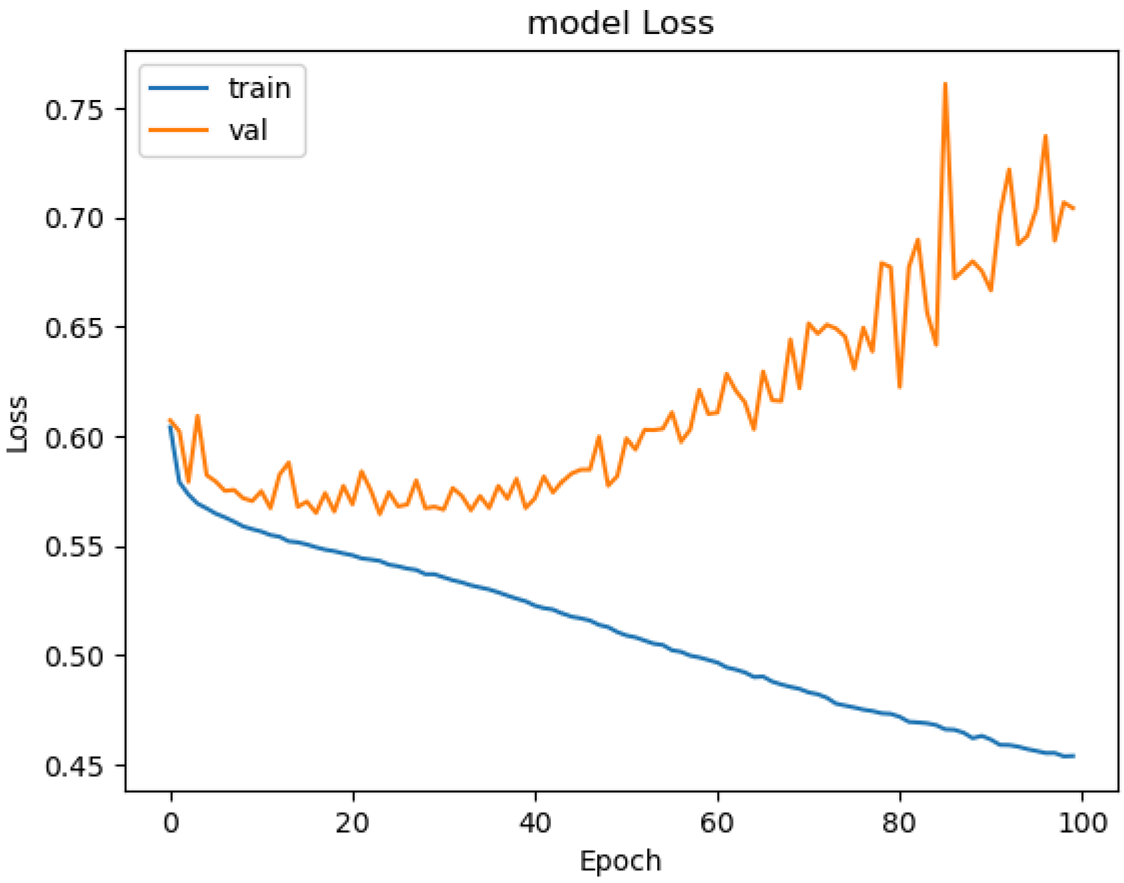

In [77]:
```
network = models.Sequential()
network.add(layers.Dense(512, activation='relu', input_shape=(23, )))
network.add(layers.Dense(256, activation='relu'))
network.add(layers.Dense(128, activation='relu'))
network.add(layers.Dense(64, activation='relu'))
network.add(layers.Dense(32, activation='relu'))
network.add(layers.Dense(16, activation='relu'))
network.add(layers.Dense(8, activation='relu'))
network.add(layers.Dense(4, activation='relu'))
network.add(layers.Dense(2, activation='relu'))
network.add(layers.Dense(1, activation='sigmoid'))
network.compile(optimizer=optimizers.RMSprop(),
```

```
                 loss='binary_crossentropy',
                 metrics=['accuracy'])
```

In [72]:
```
# fit model
history = network.fit(X_train, y_train,
                      batch_size=128, epochs=100,
                      validation_data = (X_val, y_val))
```

```
Epoch 1/100
579/579 [==============================] - 4s 5ms/step - loss: 0.6040 - accuracy: 0.6
808 - val_loss: 0.6071 - val_accuracy: 0.6706
Epoch 2/100
579/579 [==============================] - 3s 6ms/step - loss: 0.5791 - accuracy: 0.6
935 - val_loss: 0.6021 - val_accuracy: 0.6749
Epoch 3/100
579/579 [==============================] - 4s 6ms/step - loss: 0.5734 - accuracy: 0.6
984 - val_loss: 0.5792 - val_accuracy: 0.6944
Epoch 4/100
579/579 [==============================] - 4s 6ms/step - loss: 0.5692 - accuracy: 0.7
018 - val_loss: 0.6092 - val_accuracy: 0.6849
Epoch 5/100
579/579 [==============================] - 3s 6ms/step - loss: 0.5671 - accuracy: 0.7
033 - val_loss: 0.5823 - val_accuracy: 0.6906
Epoch 6/100
579/579 [==============================] - 3s 6ms/step - loss: 0.5647 - accuracy: 0.7
061 - val_loss: 0.5793 - val_accuracy: 0.6965
Epoch 7/100
579/579 [==============================] - 3s 6ms/step - loss: 0.5630 - accuracy: 0.7
072 - val_loss: 0.5751 - val_accuracy: 0.6984
Epoch 8/100
579/579 [==============================] - 4s 7ms/step - loss: 0.5610 - accuracy: 0.7
082 - val_loss: 0.5755 - val_accuracy: 0.7014
Epoch 9/100
579/579 [==============================] - 4s 6ms/step - loss: 0.5588 - accuracy: 0.7
093 - val_loss: 0.5717 - val_accuracy: 0.6995
Epoch 10/100
579/579 [==============================] - 3s 6ms/step - loss: 0.5575 - accuracy: 0.7
101 - val_loss: 0.5704 - val_accuracy: 0.6977
Epoch 11/100
579/579 [==============================] - 3s 5ms/step - loss: 0.5565 - accuracy: 0.7
113 - val_loss: 0.5749 - val_accuracy: 0.7008
Epoch 12/100
579/579 [==============================] - 3s 5ms/step - loss: 0.5549 - accuracy: 0.7
133 - val_loss: 0.5672 - val_accuracy: 0.7007
Epoch 13/100
579/579 [==============================] - 3s 6ms/step - loss: 0.5541 - accuracy: 0.7
141 - val_loss: 0.5825 - val_accuracy: 0.7018
Epoch 14/100
579/579 [==============================] - 3s 5ms/step - loss: 0.5520 - accuracy: 0.7
154 - val_loss: 0.5880 - val_accuracy: 0.6883
Epoch 15/100
579/579 [==============================] - 3s 5ms/step - loss: 0.5515 - accuracy: 0.7
160 - val_loss: 0.5678 - val_accuracy: 0.7002
Epoch 16/100
579/579 [==============================] - 3s 6ms/step - loss: 0.5505 - accuracy: 0.7
154 - val_loss: 0.5702 - val_accuracy: 0.7034
Epoch 17/100
579/579 [==============================] - 3s 6ms/step - loss: 0.5493 - accuracy: 0.7
178 - val_loss: 0.5650 - val_accuracy: 0.7049
Epoch 18/100
579/579 [==============================] - 3s 5ms/step - loss: 0.5482 - accuracy: 0.7
189 - val_loss: 0.5741 - val_accuracy: 0.7020
Epoch 19/100
579/579 [==============================] - 3s 5ms/step - loss: 0.5475 - accuracy: 0.7
182 - val_loss: 0.5656 - val_accuracy: 0.7040
Epoch 20/100
579/579 [==============================] - 3s 6ms/step - loss: 0.5465 - accuracy: 0.7
206 - val_loss: 0.5774 - val_accuracy: 0.7004
```

```
Epoch 21/100
579/579 [==============================] - 3s 6ms/step - loss: 0.5456 - accuracy: 0.7
201 - val_loss: 0.5689 - val_accuracy: 0.7082
Epoch 22/100
579/579 [==============================] - 4s 7ms/step - loss: 0.5442 - accuracy: 0.7
222 - val_loss: 0.5840 - val_accuracy: 0.6906
Epoch 23/100
579/579 [==============================] - 3s 6ms/step - loss: 0.5437 - accuracy: 0.7
227 - val_loss: 0.5754 - val_accuracy: 0.6963
Epoch 24/100
579/579 [==============================] - 3s 5ms/step - loss: 0.5430 - accuracy: 0.7
221 - val_loss: 0.5644 - val_accuracy: 0.7072
Epoch 25/100
579/579 [==============================] - 3s 5ms/step - loss: 0.5413 - accuracy: 0.7
249 - val_loss: 0.5745 - val_accuracy: 0.7067
Epoch 26/100
579/579 [==============================] - 3s 5ms/step - loss: 0.5406 - accuracy: 0.7
263 - val_loss: 0.5680 - val_accuracy: 0.7096
Epoch 27/100
579/579 [==============================] - 3s 5ms/step - loss: 0.5395 - accuracy: 0.7
251 - val_loss: 0.5688 - val_accuracy: 0.7076
Epoch 28/100
579/579 [==============================] - 3s 5ms/step - loss: 0.5389 - accuracy: 0.7
247 - val_loss: 0.5798 - val_accuracy: 0.7025
Epoch 29/100
579/579 [==============================] - 3s 6ms/step - loss: 0.5370 - accuracy: 0.7
273 - val_loss: 0.5672 - val_accuracy: 0.7060
Epoch 30/100
579/579 [==============================] - 3s 5ms/step - loss: 0.5369 - accuracy: 0.7
282 - val_loss: 0.5678 - val_accuracy: 0.7112
Epoch 31/100
579/579 [==============================] - 3s 5ms/step - loss: 0.5355 - accuracy: 0.7
297 - val_loss: 0.5665 - val_accuracy: 0.7076
Epoch 32/100
579/579 [==============================] - 3s 5ms/step - loss: 0.5342 - accuracy: 0.7
290 - val_loss: 0.5764 - val_accuracy: 0.7043
Epoch 33/100
579/579 [==============================] - 3s 5ms/step - loss: 0.5332 - accuracy: 0.7
290 - val_loss: 0.5728 - val_accuracy: 0.7051
Epoch 34/100
579/579 [==============================] - 3s 5ms/step - loss: 0.5318 - accuracy: 0.7
324 - val_loss: 0.5662 - val_accuracy: 0.7063
Epoch 35/100
579/579 [==============================] - 3s 6ms/step - loss: 0.5309 - accuracy: 0.7
324 - val_loss: 0.5727 - val_accuracy: 0.7025
Epoch 36/100
579/579 [==============================] - 3s 6ms/step - loss: 0.5299 - accuracy: 0.7
329 - val_loss: 0.5672 - val_accuracy: 0.7098
Epoch 37/100
579/579 [==============================] - 3s 6ms/step - loss: 0.5286 - accuracy: 0.7
337 - val_loss: 0.5773 - val_accuracy: 0.7031
Epoch 38/100
579/579 [==============================] - 3s 4ms/step - loss: 0.5271 - accuracy: 0.7
350 - val_loss: 0.5715 - val_accuracy: 0.7090
Epoch 39/100
579/579 [==============================] - 3s 5ms/step - loss: 0.5258 - accuracy: 0.7
371 - val_loss: 0.5806 - val_accuracy: 0.7030
Epoch 40/100
579/579 [==============================] - 3s 5ms/step - loss: 0.5245 - accuracy: 0.7
378 - val_loss: 0.5672 - val_accuracy: 0.7102
```

```
Epoch 41/100
579/579 [==============================] - 3s 4ms/step - loss: 0.5226 - accuracy: 0.7
382 - val_loss: 0.5716 - val_accuracy: 0.7023
Epoch 42/100
579/579 [==============================] - 3s 6ms/step - loss: 0.5214 - accuracy: 0.7
396 - val_loss: 0.5817 - val_accuracy: 0.7112
Epoch 43/100
579/579 [==============================] - 3s 6ms/step - loss: 0.5208 - accuracy: 0.7
404 - val_loss: 0.5744 - val_accuracy: 0.7109
Epoch 44/100
579/579 [==============================] - 3s 6ms/step - loss: 0.5189 - accuracy: 0.7
411 - val_loss: 0.5794 - val_accuracy: 0.7121
Epoch 45/100
579/579 [==============================] - 3s 5ms/step - loss: 0.5175 - accuracy: 0.7
416 - val_loss: 0.5830 - val_accuracy: 0.7095
Epoch 46/100
579/579 [==============================] - 3s 5ms/step - loss: 0.5167 - accuracy: 0.7
439 - val_loss: 0.5848 - val_accuracy: 0.7010
Epoch 47/100
579/579 [==============================] - 3s 5ms/step - loss: 0.5157 - accuracy: 0.7
440 - val_loss: 0.5848 - val_accuracy: 0.7067
Epoch 48/100
579/579 [==============================] - 3s 4ms/step - loss: 0.5137 - accuracy: 0.7
443 - val_loss: 0.5997 - val_accuracy: 0.6882
Epoch 49/100
579/579 [==============================] - 3s 5ms/step - loss: 0.5127 - accuracy: 0.7
458 - val_loss: 0.5774 - val_accuracy: 0.7073
Epoch 50/100
579/579 [==============================] - 3s 5ms/step - loss: 0.5105 - accuracy: 0.7
470 - val_loss: 0.5819 - val_accuracy: 0.7055
Epoch 51/100
579/579 [==============================] - 3s 5ms/step - loss: 0.5089 - accuracy: 0.7
473 - val_loss: 0.5989 - val_accuracy: 0.7016
Epoch 52/100
579/579 [==============================] - 3s 5ms/step - loss: 0.5080 - accuracy: 0.7
477 - val_loss: 0.5940 - val_accuracy: 0.7053
Epoch 53/100
579/579 [==============================] - 3s 5ms/step - loss: 0.5066 - accuracy: 0.7
494 - val_loss: 0.6028 - val_accuracy: 0.7051
Epoch 54/100
579/579 [==============================] - 3s 5ms/step - loss: 0.5051 - accuracy: 0.7
511 - val_loss: 0.6027 - val_accuracy: 0.7028
Epoch 55/100
579/579 [==============================] - 3s 5ms/step - loss: 0.5045 - accuracy: 0.7
506 - val_loss: 0.6033 - val_accuracy: 0.7086
Epoch 56/100
579/579 [==============================] - 3s 5ms/step - loss: 0.5021 - accuracy: 0.7
522 - val_loss: 0.6108 - val_accuracy: 0.7002
Epoch 57/100
579/579 [==============================] - 4s 6ms/step - loss: 0.5014 - accuracy: 0.7
523 - val_loss: 0.5974 - val_accuracy: 0.6984
Epoch 58/100
579/579 [==============================] - 3s 5ms/step - loss: 0.4997 - accuracy: 0.7
542 - val_loss: 0.6029 - val_accuracy: 0.6850
Epoch 59/100
579/579 [==============================] - 3s 6ms/step - loss: 0.4988 - accuracy: 0.7
546 - val_loss: 0.6210 - val_accuracy: 0.7060
Epoch 60/100
579/579 [==============================] - 3s 5ms/step - loss: 0.4977 - accuracy: 0.7
553 - val_loss: 0.6101 - val_accuracy: 0.7049
```

```
Epoch 61/100
579/579 [==============================] - 3s 5ms/step - loss: 0.4965 - accuracy: 0.7
548 - val_loss: 0.6107 - val_accuracy: 0.7060
Epoch 62/100
579/579 [==============================] - 3s 5ms/step - loss: 0.4943 - accuracy: 0.7
578 - val_loss: 0.6284 - val_accuracy: 0.6943
Epoch 63/100
579/579 [==============================] - 3s 6ms/step - loss: 0.4933 - accuracy: 0.7
578 - val_loss: 0.6208 - val_accuracy: 0.6958
Epoch 64/100
579/579 [==============================] - 3s 6ms/step - loss: 0.4920 - accuracy: 0.7
579 - val_loss: 0.6154 - val_accuracy: 0.6990
Epoch 65/100
579/579 [==============================] - 3s 6ms/step - loss: 0.4899 - accuracy: 0.7
609 - val_loss: 0.6030 - val_accuracy: 0.7027
Epoch 66/100
579/579 [==============================] - 3s 5ms/step - loss: 0.4901 - accuracy: 0.7
602 - val_loss: 0.6295 - val_accuracy: 0.6969
Epoch 67/100
579/579 [==============================] - 3s 5ms/step - loss: 0.4878 - accuracy: 0.7
626 - val_loss: 0.6163 - val_accuracy: 0.7017
Epoch 68/100
579/579 [==============================] - 3s 5ms/step - loss: 0.4865 - accuracy: 0.7
617 - val_loss: 0.6160 - val_accuracy: 0.6946
Epoch 69/100
579/579 [==============================] - 3s 4ms/step - loss: 0.4854 - accuracy: 0.7
639 - val_loss: 0.6442 - val_accuracy: 0.6877
Epoch 70/100
579/579 [==============================] - 3s 5ms/step - loss: 0.4844 - accuracy: 0.7
650 - val_loss: 0.6219 - val_accuracy: 0.6956
Epoch 71/100
579/579 [==============================] - 3s 5ms/step - loss: 0.4828 - accuracy: 0.7
648 - val_loss: 0.6515 - val_accuracy: 0.6870
Epoch 72/100
579/579 [==============================] - 3s 5ms/step - loss: 0.4820 - accuracy: 0.7
659 - val_loss: 0.6468 - val_accuracy: 0.7051
Epoch 73/100
579/579 [==============================] - 3s 5ms/step - loss: 0.4804 - accuracy: 0.7
661 - val_loss: 0.6509 - val_accuracy: 0.6969
Epoch 74/100
579/579 [==============================] - 3s 4ms/step - loss: 0.4777 - accuracy: 0.7
663 - val_loss: 0.6492 - val_accuracy: 0.6917
Epoch 75/100
579/579 [==============================] - 3s 5ms/step - loss: 0.4768 - accuracy: 0.7
689 - val_loss: 0.6454 - val_accuracy: 0.7009
Epoch 76/100
579/579 [==============================] - 3s 6ms/step - loss: 0.4759 - accuracy: 0.7
691 - val_loss: 0.6306 - val_accuracy: 0.7018
Epoch 77/100
579/579 [==============================] - 3s 5ms/step - loss: 0.4750 - accuracy: 0.7
697 - val_loss: 0.6496 - val_accuracy: 0.6962
Epoch 78/100
579/579 [==============================] - 3s 6ms/step - loss: 0.4743 - accuracy: 0.7
706 - val_loss: 0.6387 - val_accuracy: 0.7015
Epoch 79/100
579/579 [==============================] - 3s 5ms/step - loss: 0.4733 - accuracy: 0.7
694 - val_loss: 0.6791 - val_accuracy: 0.6965
Epoch 80/100
579/579 [==============================] - 3s 5ms/step - loss: 0.4730 - accuracy: 0.7
709 - val_loss: 0.6772 - val_accuracy: 0.6951
```

```
Epoch 81/100
579/579 [==============================] - 3s 5ms/step - loss: 0.4716 - accuracy: 0.7
714 - val_loss: 0.6224 - val_accuracy: 0.6985
Epoch 82/100
579/579 [==============================] - 3s 5ms/step - loss: 0.4693 - accuracy: 0.7
725 - val_loss: 0.6773 - val_accuracy: 0.7018
Epoch 83/100
579/579 [==============================] - 3s 5ms/step - loss: 0.4691 - accuracy: 0.7
731 - val_loss: 0.6899 - val_accuracy: 0.6926
Epoch 84/100
579/579 [==============================] - 3s 5ms/step - loss: 0.4687 - accuracy: 0.7
717 - val_loss: 0.6565 - val_accuracy: 0.7014
Epoch 85/100
579/579 [==============================] - 3s 5ms/step - loss: 0.4678 - accuracy: 0.7
740 - val_loss: 0.6417 - val_accuracy: 0.6964
Epoch 86/100
579/579 [==============================] - 3s 5ms/step - loss: 0.4659 - accuracy: 0.7
748 - val_loss: 0.7612 - val_accuracy: 0.6958
Epoch 87/100
579/579 [==============================] - 3s 5ms/step - loss: 0.4657 - accuracy: 0.7
754 - val_loss: 0.6721 - val_accuracy: 0.7023
Epoch 88/100
579/579 [==============================] - 3s 5ms/step - loss: 0.4644 - accuracy: 0.7
752 - val_loss: 0.6760 - val_accuracy: 0.6974
Epoch 89/100
579/579 [==============================] - 3s 5ms/step - loss: 0.4619 - accuracy: 0.7
781 - val_loss: 0.6799 - val_accuracy: 0.7026
Epoch 90/100
579/579 [==============================] - 3s 5ms/step - loss: 0.4629 - accuracy: 0.7
764 - val_loss: 0.6755 - val_accuracy: 0.6925
Epoch 91/100
579/579 [==============================] - 3s 5ms/step - loss: 0.4612 - accuracy: 0.7
793 - val_loss: 0.6666 - val_accuracy: 0.6976
Epoch 92/100
579/579 [==============================] - 4s 7ms/step - loss: 0.4588 - accuracy: 0.7
803 - val_loss: 0.7017 - val_accuracy: 0.6993
Epoch 93/100
579/579 [==============================] - 3s 6ms/step - loss: 0.4587 - accuracy: 0.7
806 - val_loss: 0.7220 - val_accuracy: 0.6962
Epoch 94/100
579/579 [==============================] - 3s 5ms/step - loss: 0.4580 - accuracy: 0.7
790 - val_loss: 0.6877 - val_accuracy: 0.6988
Epoch 95/100
579/579 [==============================] - 3s 5ms/step - loss: 0.4569 - accuracy: 0.7
806 - val_loss: 0.6916 - val_accuracy: 0.6956
Epoch 96/100
579/579 [==============================] - 3s 6ms/step - loss: 0.4560 - accuracy: 0.7
808 - val_loss: 0.7040 - val_accuracy: 0.6918
Epoch 97/100
579/579 [==============================] - 3s 5ms/step - loss: 0.4551 - accuracy: 0.7
823 - val_loss: 0.7373 - val_accuracy: 0.6976
Epoch 98/100
579/579 [==============================] - 4s 6ms/step - loss: 0.4551 - accuracy: 0.7
820 - val_loss: 0.6894 - val_accuracy: 0.6972
Epoch 99/100
579/579 [==============================] - 4s 7ms/step - loss: 0.4536 - accuracy: 0.7
823 - val_loss: 0.7069 - val_accuracy: 0.6988
Epoch 100/100
579/579 [==============================] - 4s 7ms/step - loss: 0.4537 - accuracy: 0.7
820 - val_loss: 0.7043 - val_accuracy: 0.6939
```

In [74]:
```
# Concatenate Train and Validation Data
X_training = np.concatenate((X_train, X_val), axis=0)
y_training = np.concatenate((y_train, y_val), axis=0)
```

In [78]:
```
# Re-train network on complete Training Data, with Epochs set at 25
network.fit(X_training, y_training,
            batch_size=128, epochs=40)
```

```
Epoch 1/40
771/771 [==============================] - 4s 4ms/step - loss: 0.6415 - accuracy: 0.6
785
Epoch 2/40
771/771 [==============================] - 3s 4ms/step - loss: 0.5987 - accuracy: 0.6
908
Epoch 3/40
771/771 [==============================] - 3s 4ms/step - loss: 0.5841 - accuracy: 0.6
953
Epoch 4/40
771/771 [==============================] - 3s 4ms/step - loss: 0.5773 - accuracy: 0.6
995
Epoch 5/40
771/771 [==============================] - 3s 4ms/step - loss: 0.5730 - accuracy: 0.7
007
Epoch 6/40
771/771 [==============================] - 3s 4ms/step - loss: 0.5701 - accuracy: 0.7
026
Epoch 7/40
771/771 [==============================] - 4s 5ms/step - loss: 0.5676 - accuracy: 0.7
035
Epoch 8/40
771/771 [==============================] - 3s 4ms/step - loss: 0.5650 - accuracy: 0.7
049
Epoch 9/40
771/771 [==============================] - 3s 4ms/step - loss: 0.5626 - accuracy: 0.7
089
Epoch 10/40
771/771 [==============================] - 4s 5ms/step - loss: 0.5605 - accuracy: 0.7
104
Epoch 11/40
771/771 [==============================] - 3s 4ms/step - loss: 0.5592 - accuracy: 0.7
105
Epoch 12/40
771/771 [==============================] - 3s 4ms/step - loss: 0.5575 - accuracy: 0.7
128
Epoch 13/40
771/771 [==============================] - 3s 4ms/step - loss: 0.5562 - accuracy: 0.7
132
Epoch 14/40
771/771 [==============================] - 4s 5ms/step - loss: 0.5551 - accuracy: 0.7
135
Epoch 15/40
771/771 [==============================] - 3s 4ms/step - loss: 0.5533 - accuracy: 0.7
162
Epoch 16/40
771/771 [==============================] - 3s 4ms/step - loss: 0.5529 - accuracy: 0.7
156
Epoch 17/40
771/771 [==============================] - 3s 4ms/step - loss: 0.5515 - accuracy: 0.7
177
Epoch 18/40
771/771 [==============================] - 3s 4ms/step - loss: 0.5509 - accuracy: 0.7
172
Epoch 19/40
771/771 [==============================] - 3s 4ms/step - loss: 0.5503 - accuracy: 0.7
187
Epoch 20/40
771/771 [==============================] - 3s 4ms/step - loss: 0.5499 - accuracy: 0.7
178
```

```
Epoch 21/40
771/771 [==============================] - 3s 4ms/step - loss: 0.5491 - accuracy: 0.7
188
Epoch 22/40
771/771 [==============================] - 3s 4ms/step - loss: 0.5479 - accuracy: 0.7
196
Epoch 23/40
771/771 [==============================] - 3s 4ms/step - loss: 0.5464 - accuracy: 0.7
211
Epoch 24/40
771/771 [==============================] - 4s 5ms/step - loss: 0.5463 - accuracy: 0.7
219
Epoch 25/40
771/771 [==============================] - 4s 5ms/step - loss: 0.5460 - accuracy: 0.7
211
Epoch 26/40
771/771 [==============================] - 4s 5ms/step - loss: 0.5452 - accuracy: 0.7
227
Epoch 27/40
771/771 [==============================] - 3s 4ms/step - loss: 0.5445 - accuracy: 0.7
226
Epoch 28/40
771/771 [==============================] - 3s 4ms/step - loss: 0.5436 - accuracy: 0.7
229
Epoch 29/40
771/771 [==============================] - 3s 4ms/step - loss: 0.5436 - accuracy: 0.7
225
Epoch 30/40
771/771 [==============================] - 3s 4ms/step - loss: 0.5431 - accuracy: 0.7
238
Epoch 31/40
771/771 [==============================] - 3s 4ms/step - loss: 0.5420 - accuracy: 0.7
251
Epoch 32/40
771/771 [==============================] - 3s 4ms/step - loss: 0.5414 - accuracy: 0.7
259
Epoch 33/40
771/771 [==============================] - 3s 4ms/step - loss: 0.5412 - accuracy: 0.7
252
Epoch 34/40
771/771 [==============================] - 3s 4ms/step - loss: 0.5406 - accuracy: 0.7
252
Epoch 35/40
771/771 [==============================] - 3s 4ms/step - loss: 0.5396 - accuracy: 0.7
258
Epoch 36/40
771/771 [==============================] - 3s 4ms/step - loss: 0.5392 - accuracy: 0.7
271
Epoch 37/40
771/771 [==============================] - 3s 4ms/step - loss: 0.5384 - accuracy: 0.7
277
Epoch 38/40
771/771 [==============================] - 3s 4ms/step - loss: 0.5380 - accuracy: 0.7
281
Epoch 39/40
771/771 [==============================] - 3s 4ms/step - loss: 0.5373 - accuracy: 0.7
280
Epoch 40/40
771/771 [==============================] - 3s 4ms/step - loss: 0.5369 - accuracy: 0.7
285
```

Out[78]:
```
<keras.src.callbacks.History at 0x2330eed9bd0>
```

In [79]:
```
y_prediction = network.predict(X_test)
```

```
771/771 [==============================] - 2s 2ms/step
```

In [80]:
```
network.evaluate(X_test, y_test)
```

```
771/771 [==============================] - 1s 2ms/step - loss: 0.5579 - accuracy: 0.7
140
```

Out[80]:
```
[0.5579021573066711, 0.7140077948570251]
```

In [81]:
```
y_prediction[y_prediction >= 0.5] = 1
y_prediction[y_prediction < 0.5] = 0
```

In [82]:
```
nnDF= pd.DataFrame(y_prediction)
```

In [83]:
```
nnDF.value_counts()
```

Out[83]:
```
0.0    15515
1.0     9157
Name: count, dtype: int64
```

In [84]:
```
plotCM(y_test, y_prediction)
```

```
accuracy Score is: 0.7140077821011673
```

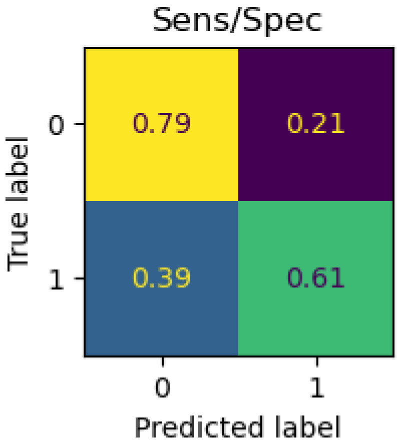

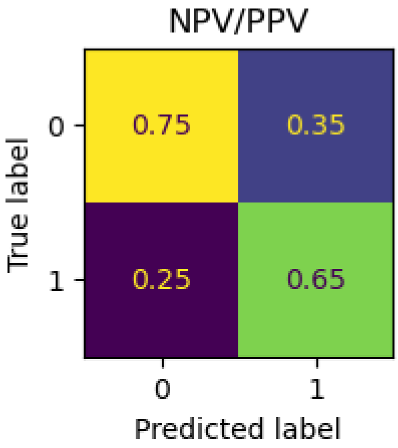

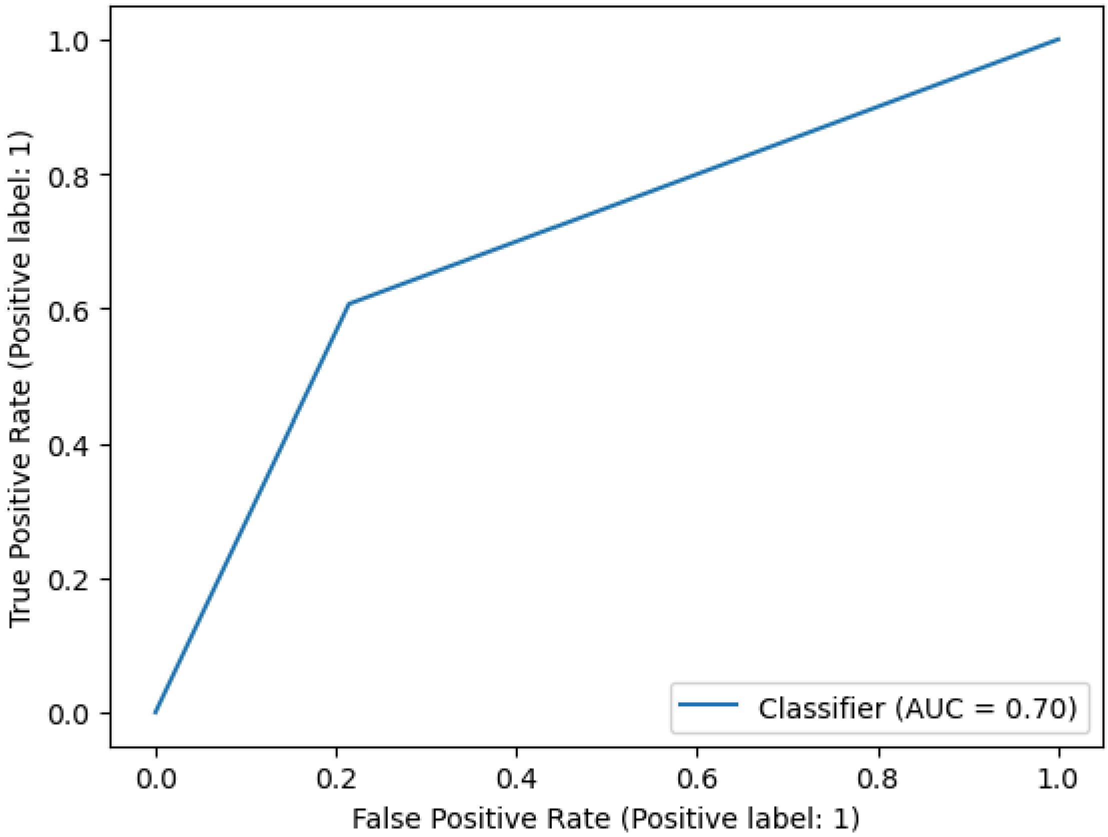

1.0
0.8
0.6
0.4
0.2
0.0
True Positive Rate (Positive label: 1)
Classifier (AUC = 0.70)
0.0
0.2
0.4
0.6
0.8
1.0
False Positive Rate (Positive label: 1)

Reimport files and re-process for 72 hour threshold modeling

In [5]:
```
df5 = pd.read_csv('/Users/ganatrh/Downloads/DataWithPrimaryDiagnosis2.csv')
df70 = pd.read_csv('/Users/ganatrh/Downloads/PIM.csv')
```

In [6]:
```
df5 = df5.merge(df70, on='Case Index Id', how='left' )
```

In [7]:
```
df5.info()
```

```
<class 'pandas.core.frame.DataFrame'>
RangeIndex: 608512 entries, 0 to 608511
Data columns (total 78 columns):
 #   Column                                          Non-Null Count   Dtype  
---  ------                                          --------------   -----  
 0   Unit Id                                         608512 non-null  int64  
 1   Case Id                                         608512 non-null  int64  
 2   Case Index Id                                   608512 non-null  object 
 3   Discharge Year                                  608512 non-null  int64  
 4   Is Readmission                                  608512 non-null  int64  
 5   Age                                             608512 non-null  object 
 6   Weight (kg)                                     608512 non-null  float64
 7   Flag - Age/Weight                               608512 non-null  int64  
 8   Collects height                                 608512 non-null  int64  
 9   Height (cm)                                     309701 non-null  float64
 10  Gender                                          608512 non-null  object 
 11  Collects Race                                   608512 non-null  int64  
 12  Race                                            550714 non-null  object 
 13  Patient Origin                                  608512 non-null  object 
 14  Trauma                                          608512 non-null  int64  
 15  Patient Type                                    608512 non-null  object 
 16  Collects Transport Team                         608512 non-null  int64  
 17  Transport Team                                  371934 non-null  object 
 18  Collects Transport Vehicle                      608512 non-null  int64  
 19  Transport Vehicle                               347485 non-null  object 
 20  Post Operative                                  608512 non-null  int64  
 21  Collects Baseline PCPC/POPC                     608512 non-null  int64  
 22  Baseline PCPC                                   127543 non-null  object 
 23  Baseline POPC                                   127464 non-null  object 
 24  Collects FSS                                    608512 non-null  int64  
 25  Baseline FSS                                    59273 non-null   float64
 26  Cardiac Patient                                 608512 non-null  int64  
 27  Cardiac Procedure directly prior to or during stay  608512 non-null  int64  
 28  Medical Length of Stay (days)                   584327 non-null  float64
 29  Physical Length of Stay (days)                  608512 non-null  float64
 30  Hospital LOS                                    606176 non-null  float64
 31  Outcome                                         608512 non-null  object 
 32  Disposition                                     608512 non-null  object 
 33  Collects Limitation on Care                     608512 non-null  int64  
 34  Limitation on Care                              523209 non-null  float64
 35  Collects Brain Dead                             608512 non-null  int64  
 36  Is Brain Dead                                   4922 non-null    float64
 37  Collects Altered Code                           608512 non-null  int64  
 38  Is Altered Code                                 523227 non-null  float64
 39  Collects Withdrawal of Support                  608512 non-null  int64  
 40  Withdrawal of Support                           497357 non-null  float64
 41  Collects Autopsy                                608512 non-null  int64  
 42  Was Autopsy Performed                           11602 non-null   object 
 43  Collects Organ Donation                         608512 non-null  int64  
 44  Is Organ Donor                                  2572 non-null    float64
 45  Collects Tissue Donation                        608512 non-null  int64  
 46  Is Tissue Donor                                 3443 non-null    float64
 47  Collects Donation after Cardiac Death           608512 non-null  int64  
 48  Donation after Cardiac Death                    3563 non-null    float64
 49  Discharge PCPC                                  123029 non-null  object 
 50  Discharge POPC                                  122965 non-null  object 
 51  Discharge FSS                                   58085 non-null   float64
 52  Hospital Outcome                                604949 non-null  object 
 53  Collects Diagnosis STS Codes                    608512 non-null  int64  
 54  Collects Diagnosis ICD-9 Codes                  608512 non-null  int64  
```

```
 55  PIM 3 Score                                            608512 non-null  float64
 56  PIM 3 Probability of Death                             608512 non-null  float64
 57  Mechanical Ventilation (First Hour)                    608511 non-null  float64
 58  Elective Admission to ICU                              608511 non-null  float64
 59  PIM 3 Recovery from Surgery                            608511 non-null  object
 60  Category                                               608475 non-null  object
 61  Systolic Blood Pressure                                588367 non-null  float64
 62  Systolic Blood Pressure Unknown                        608511 non-null  float64
 63  PaO2 (mmHg)                                            13722 non-null   float64
 64  PaO2 (kPa)                                             13722 non-null   float64
 65  PaO2 Unknown                                           608511 non-null  float64
 66  FiO2                                                   13722 non-null   float64
 67  FiO2 Unknown                                           608511 non-null  float64
 68  Base Excess                                            51063 non-null   float64
 69  Base Excess Unknown                                    608511 non-null  float64
 70  PIM 3 Pupillary Reaction                               608511 non-null  object
 71  PIM 3 Pupillary Reaction Unknown                       608511 non-null  float64
 72  Admitted following Cardiac Bypass - PIM2/PIM3          608511 non-null  float64
 73  Admitted following Cardiac Bypass - PIM3 Baseline      608511 non-null  float64
 74  Admitted following Cardiac Bypass - PIM3 PostIndex     166 non-null     float64
 75  PIM 3 - No Very High Risk Dx                           608512 non-null  int64
 76  PIM 3 - No High Risk Dx                                608512 non-null  int64
 77  PIM 3 - No Low Risk Dx                                 608512 non-null  int64
dtypes: float64(31), int64(28), object(19)
memory usage: 362.1+ MB
```

Remove rows that have flagged inaccurate weights

In [8]:

```
df6 = SmartDataframe(df5, config={"llm": llm})
df7 = df6.chat("Remove rows that have the value '1' in column 'Flag - Age/Weight' and
df7.info()
```

```
<class 'pandas.core.frame.DataFrame'>
RangeIndex: 607901 entries, 0 to 607900
Data columns (total 78 columns):
 #   Column                                          Non-Null Count   Dtype  
---  ------                                          --------------   -----  
 0   Unit Id                                         607901 non-null  int64  
 1   Case Id                                         607901 non-null  int64  
 2   Case Index Id                                   607901 non-null  object 
 3   Discharge Year                                  607901 non-null  int64  
 4   Is Readmission                                  607901 non-null  int64  
 5   Age                                             607901 non-null  object 
 6   Weight (kg)                                     607901 non-null  float64
 7   Flag - Age/Weight                               607901 non-null  int64  
 8   Collects height                                 607901 non-null  int64  
 9   Height (cm)                                     309407 non-null  float64
 10  Gender                                          607901 non-null  object 
 11  Collects Race                                   607901 non-null  int64  
 12  Race                                            550155 non-null  object 
 13  Patient Origin                                  607901 non-null  object 
 14  Trauma                                          607901 non-null  int64  
 15  Patient Type                                    607901 non-null  object 
 16  Collects Transport Team                         607901 non-null  int64  
 17  Transport Team                                  371523 non-null  object 
 18  Collects Transport Vehicle                      607901 non-null  int64  
 19  Transport Vehicle                               347101 non-null  object 
 20  Post Operative                                  607901 non-null  int64  
 21  Collects Baseline PCPC/POPC                     607901 non-null  int64  
 22  Baseline PCPC                                   127387 non-null  object 
 23  Baseline POPC                                   127309 non-null  object 
 24  Collects FSS                                    607901 non-null  int64  
 25  Baseline FSS                                    59184 non-null   float64
 26  Cardiac Patient                                 607901 non-null  int64  
 27  Cardiac Procedure directly prior to or during stay  607901 non-null  int64  
 28  Medical Length of Stay (days)                   583736 non-null  float64
 29  Physical Length of Stay (days)                  607901 non-null  float64
 30  Hospital LOS                                    605567 non-null  float64
 31  Outcome                                         607901 non-null  object 
 32  Disposition                                     607901 non-null  object 
 33  Collects Limitation on Care                     607901 non-null  int64  
 34  Limitation on Care                              522675 non-null  float64
 35  Collects Brain Dead                             607901 non-null  int64  
 36  Is Brain Dead                                   4918 non-null    float64
 37  Collects Altered Code                           607901 non-null  int64  
 38  Is Altered Code                                 522693 non-null  float64
 39  Collects Withdrawal of Support                  607901 non-null  int64  
 40  Withdrawal of Support                           496859 non-null  float64
 41  Collects Autopsy                                607901 non-null  int64  
 42  Was Autopsy Performed                           11595 non-null   object 
 43  Collects Organ Donation                         607901 non-null  int64  
 44  Is Organ Donor                                  2572 non-null    float64
 45  Collects Tissue Donation                        607901 non-null  int64  
 46  Is Tissue Donor                                 3442 non-null    float64
 47  Collects Donation after Cardiac Death           607901 non-null  int64  
 48  Donation after Cardiac Death                    3562 non-null    float64
 49  Discharge PCPC                                  122879 non-null  object 
 50  Discharge POPC                                  122815 non-null  object 
 51  Discharge FSS                                   58000 non-null   float64
 52  Hospital Outcome                                604344 non-null  object 
 53  Collects Diagnosis STS Codes                    607901 non-null  int64  
 54  Collects Diagnosis ICD-9 Codes                  607901 non-null  int64  
```

```
 55  PIM 3 Score                                         607901 non-null  float64
 56  PIM 3 Probability of Death                          607901 non-null  float64
 57  Mechanical Ventilation (First Hour)                 607900 non-null  float64
 58  Elective Admission to ICU                           607900 non-null  float64
 59  PIM 3 Recovery from Surgery                         607900 non-null  object
 60  Category                                            607864 non-null  object
 61  Systolic Blood Pressure                             587779 non-null  float64
 62  Systolic Blood Pressure Unknown                     607900 non-null  float64
 63  PaO2 (mmHg)                                         13708 non-null   float64
 64  PaO2 (kPa)                                          13708 non-null   float64
 65  PaO2 Unknown                                        607900 non-null  float64
 66  FiO2                                                13708 non-null   float64
 67  FiO2 Unknown                                        607900 non-null  float64
 68  Base Excess                                         50991 non-null   float64
 69  Base Excess Unknown                                 607900 non-null  float64
 70  PIM 3 Pupillary Reaction                            607900 non-null  object
 71  PIM 3 Pupillary Reaction Unknown                    607900 non-null  float64
 72  Admitted following Cardiac Bypass - PIM2/PIM3       607900 non-null  float64
 73  Admitted following Cardiac Bypass - PIM3 Baseline   607900 non-null  float64
 74  Admitted following Cardiac Bypass - PIM3 PostIndex  166 non-null     float64
 75  PIM 3 - No Very High Risk Dx                        607901 non-null  int64
 76  PIM 3 - No High Risk Dx                             607901 non-null  int64
 77  PIM 3 - No Low Risk Dx                              607901 non-null  int64
dtypes: float64(31), int64(28), object(19)
memory usage: 361.8+ MB
```

Create dataframe with Columns of interest

In [12]:
```
df8 = SmartDataframe(df7, config={"llm": llm})
df9 = df8.chat("Select the columns 'Case Index Id', 'Is Readmission', 'Age', 'Weight (
```

In [13]:
```
df9.info()
```

```
<class 'pandas.core.frame.DataFrame'>
RangeIndex: 607901 entries, 0 to 607900
Data columns (total 41 columns):
 #   Column                                               Non-Null Count   Dtype  
---  ------                                               --------------   -----  
 0   Case Index Id                                        607901 non-null  object 
 1   Is Readmission                                       607901 non-null  int64  
 2   Age                                                  607901 non-null  object 
 3   Weight (kg)                                          607901 non-null  float64
 4   Gender                                               607901 non-null  object 
 5   Race                                                 550155 non-null  object 
 6   Patient Origin                                       607901 non-null  object 
 7   Trauma                                               607901 non-null  int64  
 8   Patient Type                                         607901 non-null  object 
 9   Transport Team                                       371523 non-null  object 
 10  Transport Vehicle                                    347101 non-null  object 
 11  Post Operative                                       607901 non-null  int64  
 12  Baseline PCPC                                        127387 non-null  object 
 13  Baseline POPC                                        127309 non-null  object 
 14  Baseline FSS                                         59184 non-null   float64
 15  Cardiac Patient                                      607901 non-null  int64  
 16  Cardiac Procedure directly prior to or during stay  607901 non-null  int64  
 17  Medical Length of Stay (days)                        583736 non-null  float64
 18  Physical Length of Stay (days)                       607901 non-null  float64
 19  Hospital LOS                                         605567 non-null  float64
 20  Outcome                                              607901 non-null  object 
 21  Disposition                                          607901 non-null  object 
 22  Is Altered Code                                      522693 non-null  float64
 23  Discharge PCPC                                       122879 non-null  object 
 24  Discharge POPC                                       122815 non-null  object 
 25  Discharge FSS                                        58000 non-null   float64
 26  Hospital Outcome                                     604344 non-null  object 
 27  PIM 3 Score                                          607901 non-null  float64
 28  PIM 3 Probability of Death                           607901 non-null  float64
 29  Mechanical Ventilation (First Hour)                  607900 non-null  float64
 30  Elective Admission to ICU                            607900 non-null  float64
 31  PIM 3 Recovery from Surgery                          607900 non-null  object 
 32  Systolic Blood Pressure                              587779 non-null  float64
 33  PaO2 (mmHg)                                          13708 non-null   float64
 34  FiO2                                                 13708 non-null   float64
 35  Base Excess                                          50991 non-null   float64
 36  PIM 3 Pupillary Reaction                             607900 non-null  object 
 37  PIM 3 - No Very High Risk Dx                         607901 non-null  int64  
 38  PIM 3 - No High Risk Dx                              607901 non-null  int64  
 39  PIM 3 - No Low Risk Dx                               607901 non-null  int64  
 40  Category                                             607864 non-null  object 
dtypes: float64(15), int64(8), object(18)
memory usage: 190.2+ MB
```

Remove rows that have NaN for "elective ICU admission" and for "POPC/PCPC". This comcomitantly removes NaN for other variables too

In [14]:
```
df9.dropna(subset=['Elective Admission to ICU', 'Baseline POPC', 'Baseline PCPC', 'Sys
df9.info()
```

```
<class 'pandas.core.frame.DataFrame'>
RangeIndex: 123485 entries, 0 to 123484
Data columns (total 41 columns):
 #   Column                                              Non-Null Count   Dtype  
---  ------                                              --------------   -----  
 0   Case Index Id                                       123485 non-null  object 
 1   Is Readmission                                      123485 non-null  int64  
 2   Age                                                 123485 non-null  object 
 3   Weight (kg)                                         123485 non-null  float64
 4   Gender                                              123485 non-null  object 
 5   Race                                                121785 non-null  object 
 6   Patient Origin                                      123485 non-null  object 
 7   Trauma                                              123485 non-null  int64  
 8   Patient Type                                        123485 non-null  object 
 9   Transport Team                                      98498 non-null   object 
 10  Transport Vehicle                                   104581 non-null  object 
 11  Post Operative                                      123485 non-null  int64  
 12  Baseline PCPC                                       123485 non-null  object 
 13  Baseline POPC                                       123485 non-null  object 
 14  Baseline FSS                                        16690 non-null   float64
 15  Cardiac Patient                                     123485 non-null  int64  
 16  Cardiac Procedure directly prior to or during stay  123485 non-null  int64  
 17  Medical Length of Stay (days)                       118321 non-null  float64
 18  Physical Length of Stay (days)                      123485 non-null  float64
 19  Hospital LOS                                        123245 non-null  float64
 20  Outcome                                             123485 non-null  object 
 21  Disposition                                         123485 non-null  object 
 22  Is Altered Code                                     122991 non-null  float64
 23  Discharge PCPC                                      118721 non-null  object 
 24  Discharge POPC                                      118695 non-null  object 
 25  Discharge FSS                                       16383 non-null   float64
 26  Hospital Outcome                                    122734 non-null  object 
 27  PIM 3 Score                                         123485 non-null  float64
 28  PIM 3 Probability of Death                          123485 non-null  float64
 29  Mechanical Ventilation (First Hour)                 123485 non-null  float64
 30  Elective Admission to ICU                           123485 non-null  float64
 31  PIM 3 Recovery from Surgery                         123485 non-null  object 
 32  Systolic Blood Pressure                             123485 non-null  float64
 33  PaO2 (mmHg)                                         3205 non-null    float64
 34  FiO2                                                3205 non-null    float64
 35  Base Excess                                         12155 non-null   float64
 36  PIM 3 Pupillary Reaction                            123485 non-null  object 
 37  PIM 3 - No Very High Risk Dx                        123485 non-null  int64  
 38  PIM 3 - No High Risk Dx                             123485 non-null  int64  
 39  PIM 3 - No Low Risk Dx                              123485 non-null  int64  
 40  Category                                            123485 non-null  object 
dtypes: float64(15), int64(8), object(18)
memory usage: 38.6+ MB
```

In [15]:

```
df9["Base Excess"] = df9["Base Excess"].replace(np.nan, 0)
df9["Base Excess"].value_counts()
```

Out[15]:

```
Base Excess
 0.0      111514
-3.0         358
-4.0         342
-5.0         329
-2.0         327
           ...  
-16.5          1
 9.7           1
 23.3          1
-34.1          1
 11.3          1
Name: count, Length: 472, dtype: int64
```

The cells below consolidate all patients > 18 into one group

In [16]:

```
df9["Age"].value_counts()
```

Out[16]:

```
Age
Infant 29 days to < 2 years                    36705
Adolescent 12 years to < 18 years              32266
Child 2 years to < 6 years                     23946
Child 6 years to < 12 years                    22954
Adolescent (late) 18 years to < 21 years        4241
Neonate Birth to 29 days                        2355
Adult 21 years and up                           1018
Name: count, dtype: int64
```

In [17]:

```
df10 = df9.copy()
df10.Age.replace(['Adult 21 years and up','Adolescent (late) 18 years to < 21 years'],
```

In [18]:

```
df10["Age"].value_counts()
```

Out[18]:

```
Age
Infant 29 days to < 2 years          36705
Adolescent 12 years to < 18 years    32266
Child 2 years to < 6 years           23946
Child 6 years to < 12 years          22954
Adult 18 years and up                 5259
Neonate Birth to 29 days              2355
Name: count, dtype: int64
```

The cells below consolidate "Patient origin" into more discrete categories

In [19]:

```
df10["Patient Origin"].value_counts()
```

```
Out[19]: Patient Origin
Emergency Department                                                        47563
Another Hospital's Emergency Department                                     21884
General Care Floor                                                          18033
Operating Room (Direct to ICU)                                              17792
Recovery Room (PACU)                                                         9248
Another Hospital's ICU                                                       1796
Another Hospital's General Care Floor                                        1753
Step-Down Unit/Intermediate Care Unit                                        1374
Home                                                                         1120
Physician's Office/Clinic                                                     959
Inpatient Procedure Suite (not cath lab)                                      558
Outpatient Procedure Suite                                                    255
NICU (in this hospital)                                                       229
Another Hospital's OR                                                         205
Other                                                                         151
Another ICU in this hospital (except NICU)                                    129
Transitional Care/Skilled Nursing/Chronic Care Facility                       122
Dedicated technology dependent unit (transitional/progressive care unit)      104
Physical Rehab Center                                                          64
Cath lab                                                                       57
Another hospital's cath lab                                                    17
Another hospital's Step-Down Unit/Intermediate Care Unit                       16
Psychiatric/Substance Abuse/Chemical Dependence Rehab Center                   13
Another Hospital's dedicated home ventilator unit                              13
Delivery Room (including delivery room in another hospital)                    12
Telemetry Unit                                                                 10
Pulmonary Rehab Center                                                          7
Another hospital's Telemetry Unit                                               1
Name: count, dtype: int64
```

```python
In [20]: df11 = df10.copy()
df11["Patient Origin"].replace(["Another Hospital's General Care Floor", "Transitional
df11["Patient Origin"].replace(["Another Hospital's ICU", "Another Hospital's OR", "Ar
df11["Patient Origin"].replace(["Home", "Physician's Office/Clinic"], "Home/Clinic",
df11["Patient Origin"].replace(["Inpatient Procedure Suite (not cath lab)", "Outpatier
df11["Patient Origin"].replace(["Step-Down Unit/Intermediate Care Unit", "Telemetry Ur
```

```python
In [21]: df11["Patient Origin"].value_counts()
```

```
Out[21]: Patient Origin
Emergency Department                                           47563
Another Hospital's Emergency Department                        21884
General Care Floor                                             18033
Operating Room (Direct to ICU)                                 17792
Recovery Room (PACU)                                            9248
Home/Clinic                                                     2079
Another Hospital ICU/OR/Cath                                    2018
Another Hospital Non-ED Non-ICU unit                            1989
Step down/Intermediate/Telemetry                                1488
Procedure Suite                                                  813
NICU (in this hospital)                                          229
Other                                                            151
Another ICU in this hospital (except NICU)                       129
Cath lab                                                          57
Delivery Room (including delivery room in another hospital)       12
Name: count, dtype: int64
```

Remove rows with PIM3 recovery from surgery showing a recovery from cardiac surgery

In [22]:
```
df11["PIM 3 Recovery from Surgery"].value_counts()
```

Out[22]:
```
PIM 3 Recovery from Surgery
No                                                  99362
Yes, Recovery from non-cardiac procedure            23992
Yes, Recovery from non-bypass cardiac procedure        98
Yes, Recovery from bypass cardiac procedure            33
Name: count, dtype: int64
```

In [23]:
```
df12 = df11.copy()
df12 = df12.drop(df12[df12['PIM 3 Recovery from Surgery'] == 'Yes, Recovery from non-b
df12 = df12.drop(df12[df12['PIM 3 Recovery from Surgery'] == 'Yes, Recovery from bypas
df12 = df12.reset_index(drop=True)
```

In [24]:
```
df12["PIM 3 Recovery from Surgery"].value_counts()
```

Out[24]:
```
PIM 3 Recovery from Surgery
No                                          99362
Yes, Recovery from non-cardiac procedure    23992
Name: count, dtype: int64
```

Fill Race NaN with "Unspecified"

In [25]:
```
df12.Race.fillna('Unspecified', inplace=True)
df12.Race.unique()
```

Out[25]:
```
array(['White', 'Other/Mixed', 'Hispanic or Latino',
       'Black or African American', 'Asian/Indian/Pacific Islander',
       'Unspecified', 'Asian', 'American Indian or Alaska Native',
       'Native Hawaiian or Other Pacific Islander'], dtype=object)
```

Consolidate Pupillary reactions to Fixed>3mm and others/unknown

In [26]:
```
df12["PIM 3 Pupillary Reaction"].value_counts()
```

Out[26]:
```
PIM 3 Pupillary Reaction
Other                                   116524
Unknown                                   5040
>3mm and both fixed                        927
Pupilary Assessment Invalid - Drugs        651
Pupilary Assessment Invalid - Injury       141
Pupilary Assessment Invalid - Toxins        71
Name: count, dtype: int64
```

In [34]:
```
df13 = df12.copy()
df13["PIM 3 Pupillary Reaction"].replace(["Other", "Unknown", "Pupilary Assessment Inv
df13["PIM 3 Pupillary Reaction"].value_counts()
```

Out[34]:
```
PIM 3 Pupillary Reaction
Other/Unknown          122427
>3mm and both fixed       927
Name: count, dtype: int64
```

In [35]:
```
chatBot = SmartDataframe(df13, config={"llm": llm})
```

In [36]:
```
chatBot.chat("For the column 'Physical Length of Stay (days)', what percentage of rows
```
Out[36]:
```
"The percentage of rows with 'Physical Length of Stay (days)' more than 3 is: 27.75%"
```
In [57]:
```
df14 = chatBot.chat("Create a new dataframe with column 'LOS', and if the value in col
df14.LOS.value_counts()
```
Out[57]:
```
LOS
0    89123
1    34231
Name: count, dtype: int64
```
In [58]:
```
df14.info()
```

```
<class 'pandas.core.frame.DataFrame'>
RangeIndex: 123354 entries, 0 to 123353
Data columns (total 42 columns):
 #   Column                                               Non-Null Count   Dtype  
---  ------                                               --------------   -----  
 0   Case Index Id                                        123354 non-null  object 
 1   Is Readmission                                       123354 non-null  int64  
 2   Age                                                  123354 non-null  object 
 3   Weight (kg)                                          123354 non-null  float64
 4   Gender                                               123354 non-null  object 
 5   Race                                                 123354 non-null  object 
 6   Patient Origin                                       123354 non-null  object 
 7   Trauma                                               123354 non-null  int64  
 8   Patient Type                                         123354 non-null  object 
 9   Transport Team                                       98391 non-null   object 
 10  Transport Vehicle                                    104468 non-null  object 
 11  Post Operative                                       123354 non-null  int64  
 12  Baseline PCPC                                        123354 non-null  object 
 13  Baseline POPC                                        123354 non-null  object 
 14  Baseline FSS                                         16666 non-null   float64
 15  Cardiac Patient                                      123354 non-null  int64  
 16  Cardiac Procedure directly prior to or during stay   123354 non-null  int64  
 17  Medical Length of Stay (days)                        118191 non-null  float64
 18  Physical Length of Stay (days)                       123354 non-null  float64
 19  Hospital LOS                                         123115 non-null  float64
 20  Outcome                                              123354 non-null  object 
 21  Disposition                                          123354 non-null  object 
 22  Is Altered Code                                      122862 non-null  float64
 23  Discharge PCPC                                       118592 non-null  object 
 24  Discharge POPC                                       118566 non-null  object 
 25  Discharge FSS                                        16359 non-null   float64
 26  Hospital Outcome                                     122609 non-null  object 
 27  PIM 3 Score                                          123354 non-null  float64
 28  PIM 3 Probability of Death                           123354 non-null  float64
 29  Mechanical Ventilation (First Hour)                  123354 non-null  float64
 30  Elective Admission to ICU                            123354 non-null  float64
 31  PIM 3 Recovery from Surgery                          123354 non-null  object 
 32  Systolic Blood Pressure                              123354 non-null  float64
 33  PaO2 (mmHg)                                          3179 non-null    float64
 34  FiO2                                                 3179 non-null    float64
 35  Base Excess                                          123354 non-null  float64
 36  PIM 3 Pupillary Reaction                             123354 non-null  object 
 37  PIM 3 - No Very High Risk Dx                         123354 non-null  int64  
 38  PIM 3 - No High Risk Dx                              123354 non-null  int64  
 39  PIM 3 - No Low Risk Dx                               123354 non-null  int64  
 40  Category                                             123354 non-null  object 
 41  LOS                                                  123354 non-null  int64  
dtypes: float64(15), int64(9), object(18)
memory usage: 39.5+ MB
```

Apply Ordinal Encoder to convert Object datatype to Integers

In [61]:
```
toEncode = df14[['Is Readmission', 'Age', 'Gender', 'Race', 'Patient Origin', 'Trauma'
                'Post Operative', 'Mechanical Ventilation (First Hour)', 'Elective Adm
                'PIM 3 Recovery from Surgery', 'Category', 'Baseline PCPC', 'Baseline 
                 'PIM 3 Pupillary Reaction']].copy()
columnNames = list(toEncode.columns)

enc = OrdinalEncoder()
```

```
df15 = pd.DataFrame(enc.fit_transform(toEncode), columns= columnNames)

for i in range(len(columnNames)):
    print (columnNames[i] , enc.categories_[i])
```

```
Is Readmission [0 1]
Age ['Adolescent 12 years to < 18 years' 'Adult 18 years and up'
 'Child 2 years to < 6 years' 'Child 6 years to < 12 years'
 'Infant 29 days to < 2 years' 'Neonate Birth to 29 days']
Gender ['Ambiguous' 'Female' 'Male']
Race ['American Indian or Alaska Native' 'Asian'
 'Asian/Indian/Pacific Islander' 'Black or African American'
 'Hispanic or Latino' 'Native Hawaiian or Other Pacific Islander'
 'Other/Mixed' 'Unspecified' 'White']
Patient Origin ['Another Hospital ICU/OR/Cath' 'Another Hospital Non-ED Non-ICU unit'
 "Another Hospital's Emergency Department"
 'Another ICU in this hospital (except NICU)' 'Cath lab'
 'Delivery Room (including delivery room in another hospital)'
 'Emergency Department' 'General Care Floor' 'Home/Clinic'
 'NICU (in this hospital)' 'Operating Room (Direct to ICU)' 'Other'
 'Procedure Suite' 'Recovery Room (PACU)'
 'Step down/Intermediate/Telemetry']
Trauma [0 1]
Patient Type ['Scheduled (> or = 12 Hours in Advance)' 'Unscheduled']
Post Operative [0 1]
Mechanical Ventilation (First Hour) [0. 1.]
Elective Admission to ICU [0. 1.]
PIM 3 Recovery from Surgery ['No' 'Yes, Recovery from non-cardiac procedure']
Category ['Cardiovascular' 'Dermatologic' 'Endocrine' 'Factors Influencing Health'
 'Gastrointestinal' 'Genetic' 'Gynecologic' 'Hematologic' 'Immunologic'
 'Infectious' 'Injury/Poisoning/Adverse Effects' 'Metabolic' 'Neurologic'
 'Newborn/Perinatal' 'Oncologic' 'Ophthalmologic' 'Orthopedic'
 'Psychiatric' 'Renal/Genitourinary' 'Respiratory' 'Respiratory/ENT'
 'Rheumatologic' 'Symptoms' 'Transplant' 'Ungroupable']
Baseline PCPC ['1 - Normal' '2 - Mild disability' '3 - Moderate disability'
 '4 - Severe disability' '5 - Coma or vegetative state']
Baseline POPC ['1 - Good overall performance' '2 - Mild overall disability'
 '3 - Moderate overall disability' '4 - Severe overall disability'
 '5 - Coma or vegetative state']
PIM 3 Pupillary Reaction ['>3mm and both fixed' 'Other/Unknown']
```

In [62]:
```
df15.info()
```

```
<class 'pandas.core.frame.DataFrame'>
RangeIndex: 123354 entries, 0 to 123353
Data columns (total 15 columns):
 #   Column                              Non-Null Count   Dtype  
---  ------                              --------------   -----  
 0   Is Readmission                      123354 non-null  float64
 1   Age                                 123354 non-null  float64
 2   Gender                              123354 non-null  float64
 3   Race                                123354 non-null  float64
 4   Patient Origin                      123354 non-null  float64
 5   Trauma                              123354 non-null  float64
 6   Patient Type                        123354 non-null  float64
 7   Post Operative                      123354 non-null  float64
 8   Mechanical Ventilation (First Hour) 123354 non-null  float64
 9   Elective Admission to ICU           123354 non-null  float64
 10  PIM 3 Recovery from Surgery         123354 non-null  float64
 11  Category                            123354 non-null  float64
 12  Baseline PCPC                       123354 non-null  float64
 13  Baseline POPC                       123354 non-null  float64
 14  PIM 3 Pupillary Reaction            123354 non-null  float64
dtypes: float64(15)
memory usage: 14.1 MB
```

Add "weight" and "PIM3" Score into the dataframe

In [63]:

```
df16 = df15.copy()

df16.insert(2, "Weight", 0)
df16.insert(15, "PIM3", 0)
df16.insert(16, "PIM 3 Probability of Death", 0)
df16.insert(17, "Systolic Blood Pressure", 0)
df16.insert(18, "Base Excess", 0)
df16.insert(20, "PIM 3 - No Very High Risk Dx", 0)
df16.insert(21, "PIM 3 - No High Risk Dx", 0)
df16.insert(22, "PIM 3 - No Low Risk Dx", 0)




df16.Weight = df14["Weight (kg)"].copy()
df16.PIM3 = df14["PIM 3 Score"].copy()
df16["PIM 3 Probability of Death"] = df14["PIM 3 Probability of Death"].copy()
df16["Systolic Blood Pressure"] = df14["Systolic Blood Pressure"].copy()
df16["Base Excess"] = df14["Base Excess"].copy()
df16["PIM 3 - No Very High Risk Dx"] = df14["PIM 3 - No Very High Risk Dx"].copy()
df16["PIM 3 - No High Risk Dx"] = df14["PIM 3 - No High Risk Dx"].copy()
df16["PIM 3 - No Low Risk Dx"] = df14["PIM 3 - No Low Risk Dx"].copy()


df16.info()
```

```
<class 'pandas.core.frame.DataFrame'>
RangeIndex: 123354 entries, 0 to 123353
Data columns (total 23 columns):
 #   Column                              Non-Null Count   Dtype  
---  ------                              --------------   -----  
 0   Is Readmission                      123354 non-null  float64
 1   Age                                 123354 non-null  float64
 2   Weight                              123354 non-null  float64
 3   Gender                              123354 non-null  float64
 4   Race                                123354 non-null  float64
 5   Patient Origin                      123354 non-null  float64
 6   Trauma                              123354 non-null  float64
 7   Patient Type                        123354 non-null  float64
 8   Post Operative                      123354 non-null  float64
 9   Mechanical Ventilation (First Hour) 123354 non-null  float64
 10  Elective Admission to ICU           123354 non-null  float64
 11  PIM 3 Recovery from Surgery         123354 non-null  float64
 12  Category                            123354 non-null  float64
 13  Baseline PCPC                       123354 non-null  float64
 14  Baseline POPC                       123354 non-null  float64
 15  PIM3                                123354 non-null  float64
 16  PIM 3 Probability of Death          123354 non-null  float64
 17  Systolic Blood Pressure             123354 non-null  float64
 18  Base Excess                         123354 non-null  float64
 19  PIM 3 Pupillary Reaction            123354 non-null  float64
 20  PIM 3 - No Very High Risk Dx        123354 non-null  int64  
 21  PIM 3 - No High Risk Dx             123354 non-null  int64  
 22  PIM 3 - No Low Risk Dx              123354 non-null  int64  
dtypes: float64(20), int64(3)
memory usage: 21.6 MB
```

Add "LOS" into the dataframe and generate HEATMAP

In [64]:
```
df17 = df16.copy()

df17.insert(23, "LOS", 0)
df17.LOS = df14.LOS.copy()
```

In [65]:
```
df17.info()
```

```
<class 'pandas.core.frame.DataFrame'>
RangeIndex: 123354 entries, 0 to 123353
Data columns (total 24 columns):
 #   Column                               Non-Null Count   Dtype  
---  ------                               --------------   -----  
 0   Is Readmission                       123354 non-null  float64
 1   Age                                  123354 non-null  float64
 2   Weight                               123354 non-null  float64
 3   Gender                               123354 non-null  float64
 4   Race                                 123354 non-null  float64
 5   Patient Origin                       123354 non-null  float64
 6   Trauma                               123354 non-null  float64
 7   Patient Type                         123354 non-null  float64
 8   Post Operative                       123354 non-null  float64
 9   Mechanical Ventilation (First Hour)  123354 non-null  float64
 10  Elective Admission to ICU            123354 non-null  float64
 11  PIM 3 Recovery from Surgery          123354 non-null  float64
 12  Category                             123354 non-null  float64
 13  Baseline PCPC                        123354 non-null  float64
 14  Baseline POPC                        123354 non-null  float64
 15  PIM3                                 123354 non-null  float64
 16  PIM 3 Probability of Death           123354 non-null  float64
 17  Systolic Blood Pressure              123354 non-null  float64
 18  Base Excess                          123354 non-null  float64
 19  PIM 3 Pupillary Reaction             123354 non-null  float64
 20  PIM 3 - No Very High Risk Dx         123354 non-null  int64  
 21  PIM 3 - No High Risk Dx              123354 non-null  int64  
 22  PIM 3 - No Low Risk Dx               123354 non-null  int64  
 23  LOS                                  123354 non-null  int64  
dtypes: float64(20), int64(4)
memory usage: 22.6 MB
```

In [66]:

```
def heatMap(list, fontSize):
    corr = list.corr()
    corr= corr.round(decimals=2)
    plt.figure(figsize=(20, 8))
    sns.set(font_scale = 1.2)
    sns.heatmap(corr, cmap='RdBu', vmin=-1, vmax=1, annot=True, annot_kws={'fontsize':
```

In [67]:

```
heatMap(df17, 12)
```

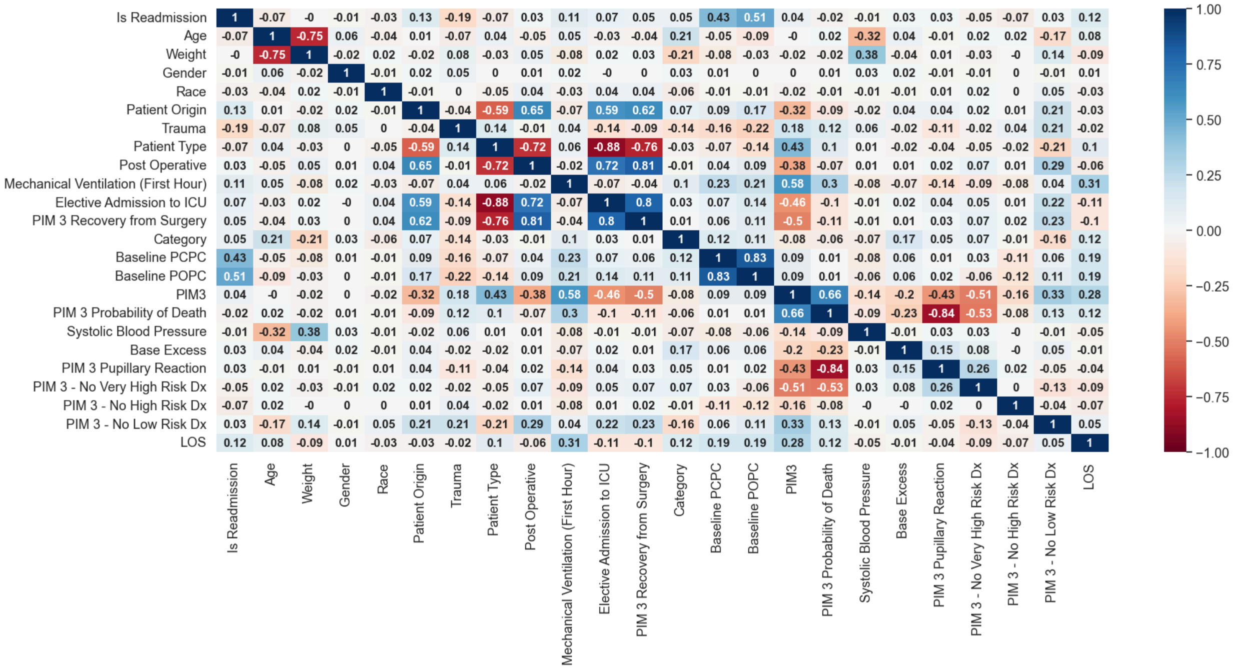

Create INPUT and OUTPUT NP arrays

In [35]:
```
input = df16.copy().to_numpy()
output = df17["LOS"].copy().to_numpy()

#input.head()
print('Input sample 25: \n' , input[27] ,  "\n \n Output sample 25: " , output[27])
```

```
Input sample 25: 
 [  0.      4.     10.8     2.      7.      1.      0.      1.      1.      1.
   0.      0.    625.      1.      2.     -1.06   25.68   89.      0.      1.
   0.      1.      1.   ]

 Output sample 25:  1
```

Apply SKLearn train/test split to Input and Output for 80:20 split

In [36]:
```
X_train, X_test, y_train, y_test = train_test_split(input, output, test_size=0.2, ranc
```

In [37]:
```
print(X_train.shape, "\t", y_train.shape, "\n")
print(X_test.shape, "\t", y_test.shape, "\n")
```

```
(98685, 23)      (98685,)

(24672, 23)      (24672,)
```

Fit MinMaxScaler on Training data, and then apply transformation to training and test set

In [38]:
```
scaler = MinMaxScaler()
scaler.fit(X_train)
```

Out[38]:

```
▾ MinMaxScaler
MinMaxScaler()
```

In [39]:

```
X_train = scaler.transform(X_train)
X_test = scaler.transform(X_test)
```

In [40]:

```
print(X_test[27], '\n' '\n', y_test[27])
```

```
[1.         0.4        0.03930435 0.5        0.75       0.92857143
 0.         0.         1.         1.         1.         1.
 0.31406045 0.75       0.75       0.17475124 0.00410287 0.42918455
 0.38844847 1.         1.         1.         1.        ]

 1
```

Import and apply LinearSVC - results appear Encouraging

Attempted standard SVC with RBF kernel too but computationally exorbitant

In [41]:

```
from sklearn.svm import LinearSVC
```

In [42]:

```
svc = LinearSVC(dual="auto", random_state=0, tol=1e-5)
svc.fit(X_train, y_train)
```

Out[42]:

```
▾ LinearSVC
LinearSVC(dual='auto', random_state=0, tol=1e-05)
```

In [43]:

```
svc_predictions = svc.predict(X_test)
```

In [44]:

```
def plotCM(test, predictions):

    print("accuracy Score is: " + str(accuracy_score(test, predictions)))

    cm = confusion_matrix(test, predictions, normalize='true')
    fig, ax = plt.subplots(figsize=(2,2))
    ConfusionMatrixDisplay(cm).plot(ax=ax, colorbar=False)
    plt.title('Sens/Spec')
    plt.show()

    cm2 = confusion_matrix(test, predictions, normalize='pred')
    fig, ax = plt.subplots(figsize=(2,2))
    ConfusionMatrixDisplay(cm2).plot(ax=ax, colorbar=False)
    plt.title('NPV/PPV')
    plt.show()

    RocCurveDisplay.from_predictions(test, predictions)
    plt.show()
```

In [45]:

```
plotCM(y_test, svc_predictions)
```

```
accuracy Score is: 0.7521481841763943
```

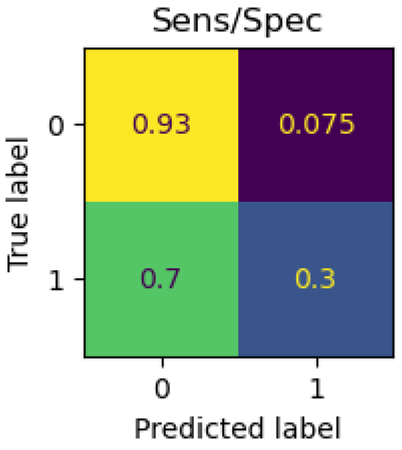

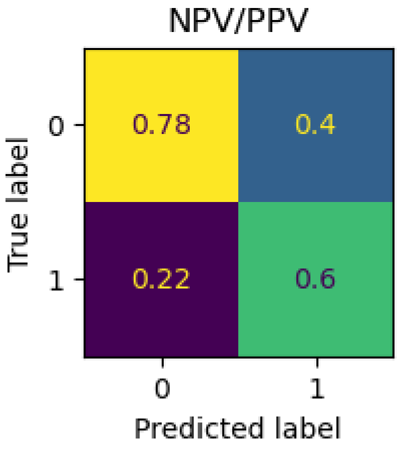

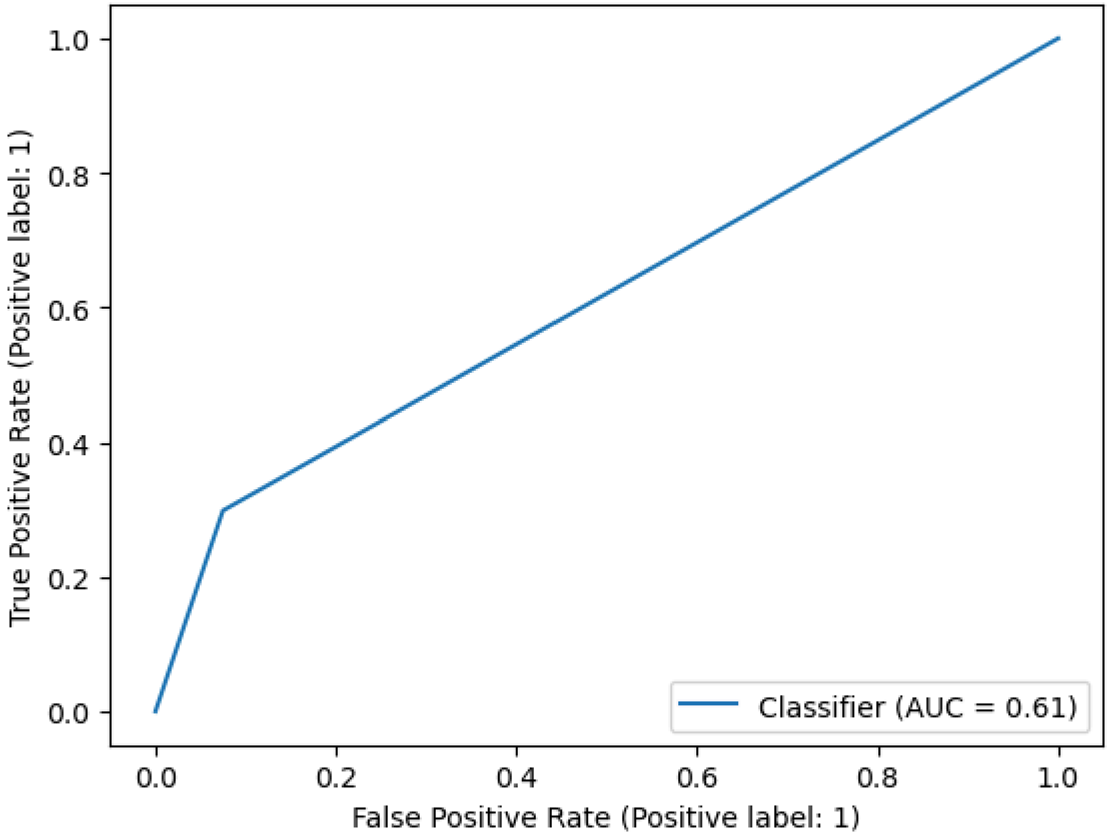

Import and apply Stochastic Gradient Descent Classification - results seen

In [46]:
```python
from sklearn.linear_model import SGDClassifier
```

In [47]:
```python
sgdc = SGDClassifier(loss="hinge", penalty="l2")
sgdc.fit(X_train, y_train)
```

Out[47]:
```
▾ SGDClassifier
SGDClassifier()
```

In [48]:
```python
sgdc_predictions = sgdc.predict(X_test)
```

In [49]:
```python
plotCM(y_test, sgdc_predictions)
```

```
accuracy Score is: 0.7361381322957199
```

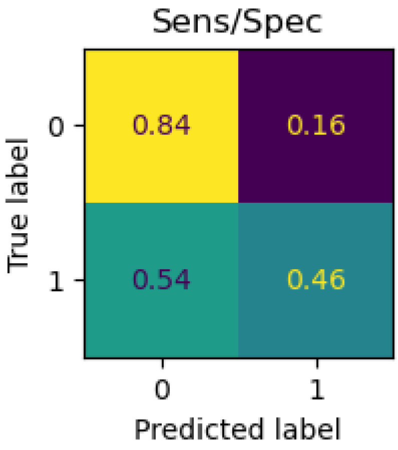

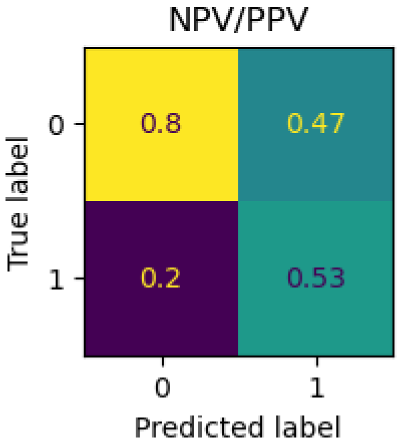

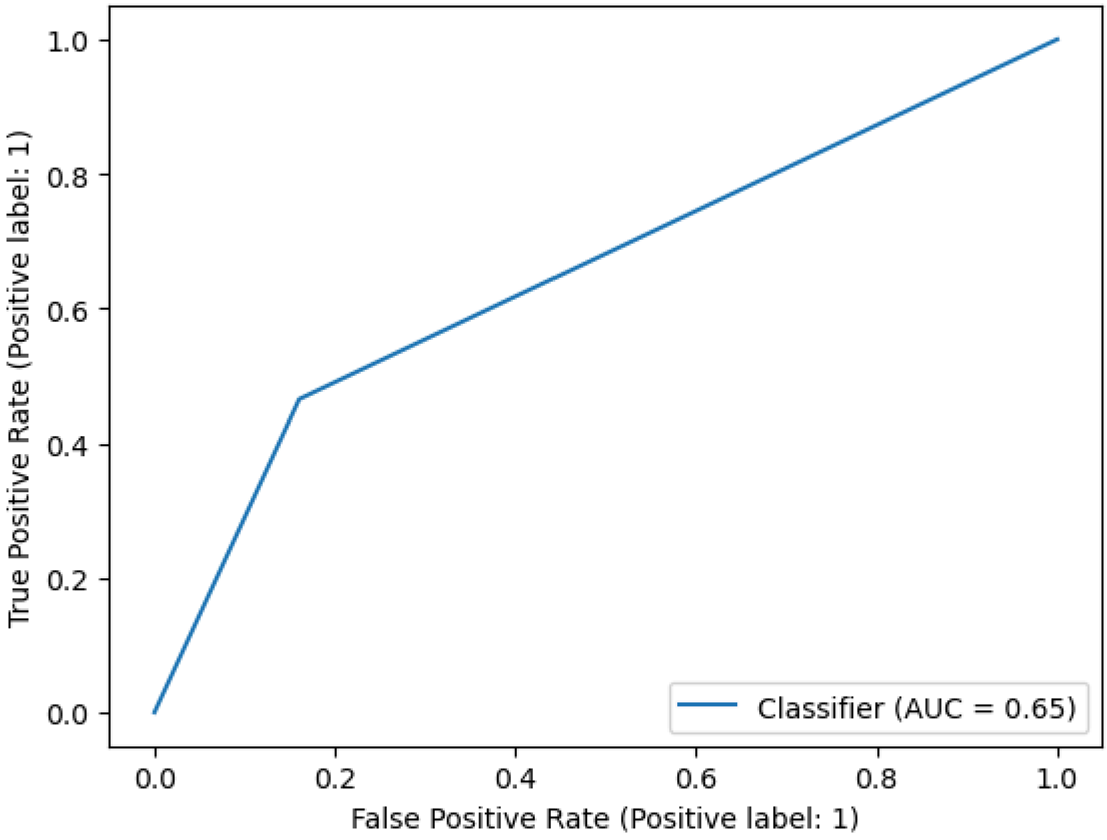

Import and apply KNN Classifier - results

In [50]:
```
from sklearn.neighbors import KNeighborsClassifier
```

In [51]:
```
knn = KNeighborsClassifier(n_neighbors=15)
knn.fit(X_train, y_train)
```

Out[51]:
```
▾ KNeighborsClassifier
KNeighborsClassifier(n_neighbors=15)
```

In [52]:
```
knn_predictions = knn.predict(X_test)
```

In [53]:
```
plotCM(y_test, knn_predictions)
```

```
accuracy Score is: 0.7505674448767834
```

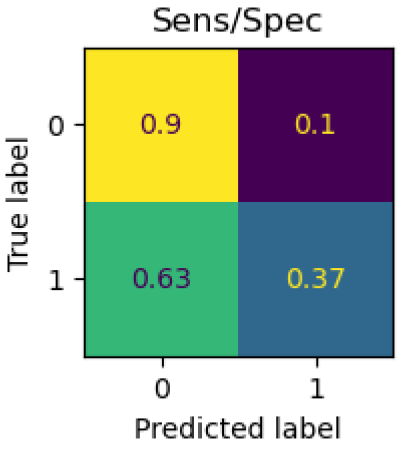

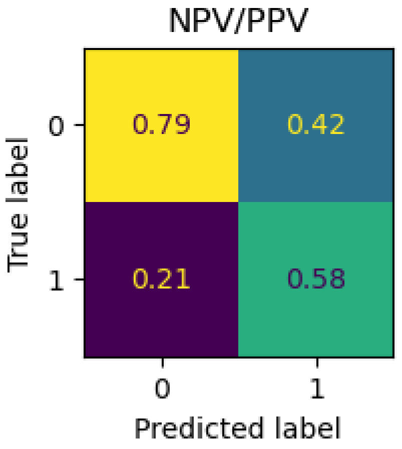

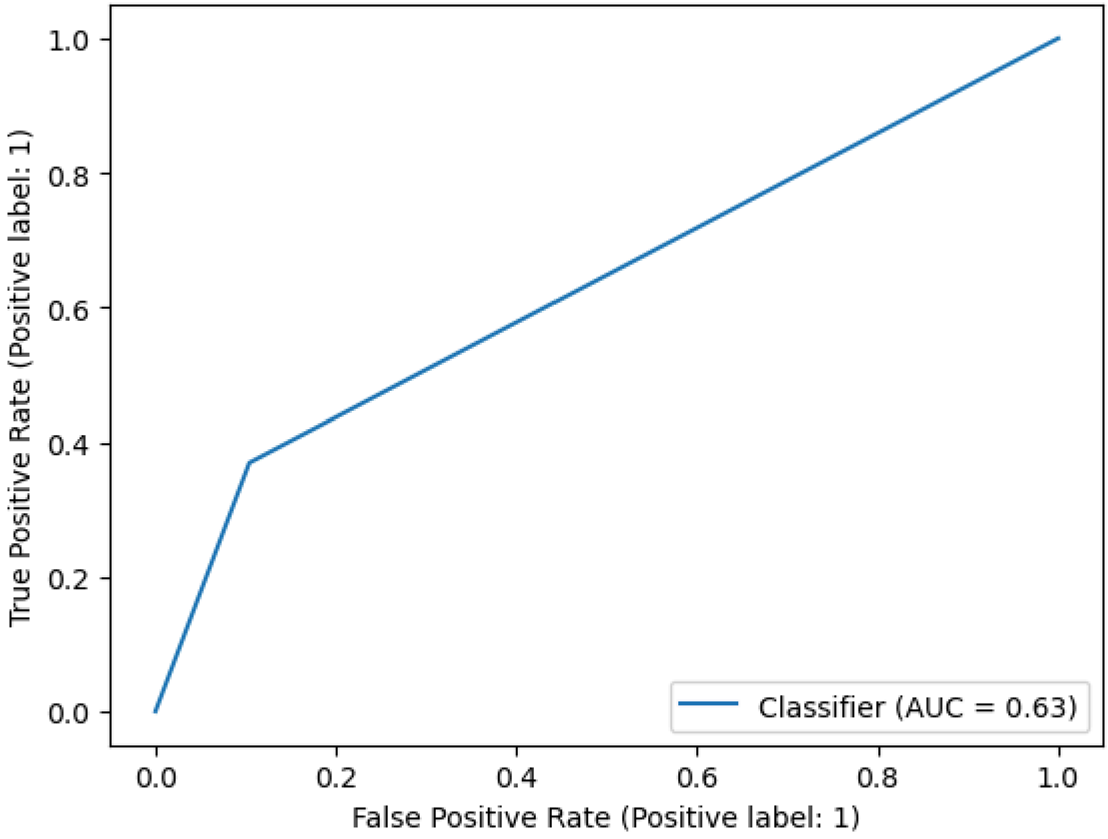

Import and apply Decision Tree Regression - results appear to be GARBAGE

In [54]:
```
from sklearn.tree import DecisionTreeClassifier
```

In [55]:
```
dtree = DecisionTreeClassifier(random_state=30)
dtree.fit(X_train, y_train)
```

Out[55]:
```
▾ DecisionTreeClassifier
DecisionTreeClassifier(random_state=30)
```

In [56]:
```
dtree_predictions = dtree.predict(X_test)
```

In [57]:
```
plotCM(y_test, dtree_predictions)
```

```
accuracy Score is: 0.6963764591439688
```

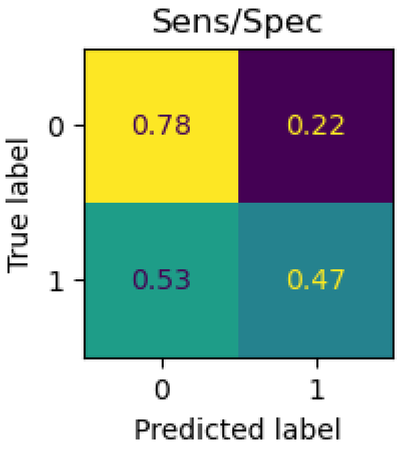

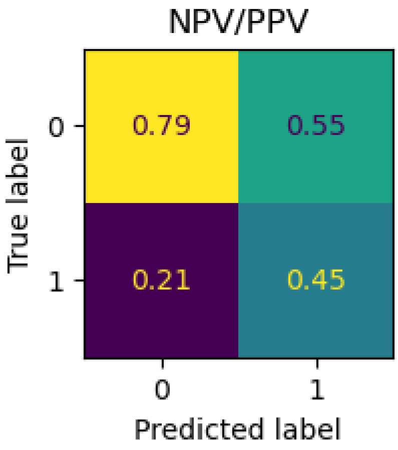

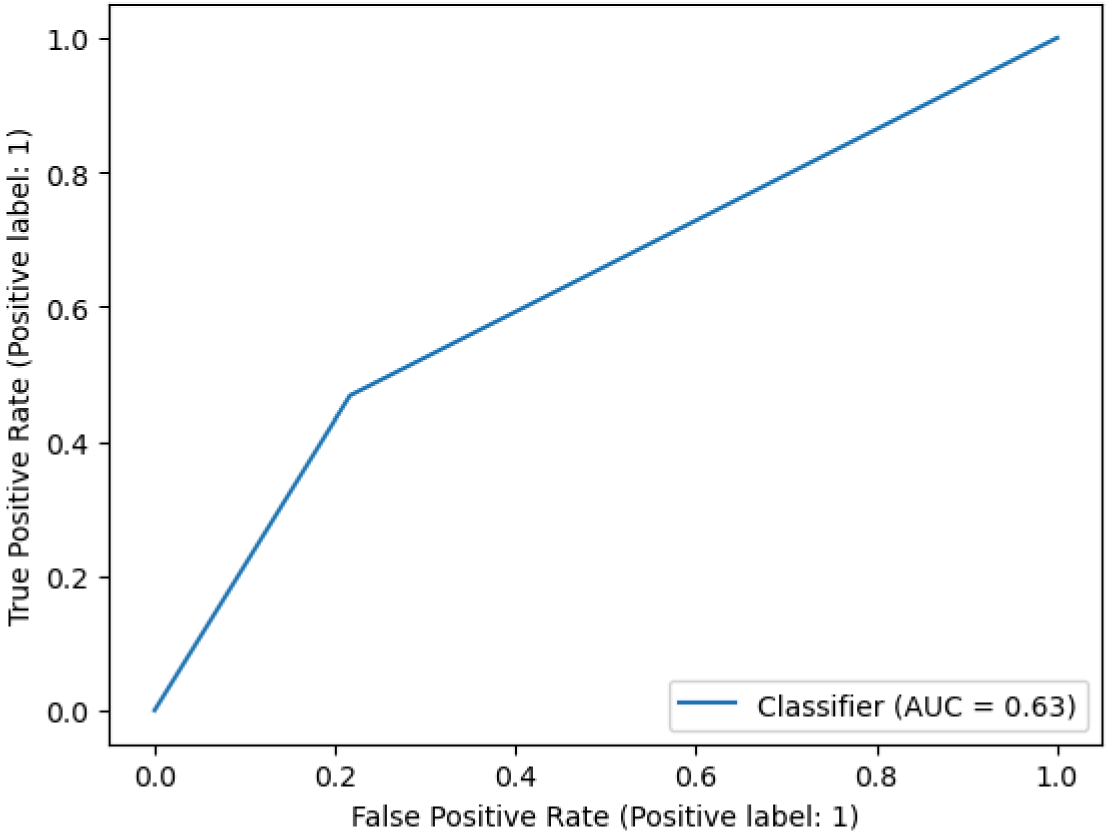

Attempt Gradient Boosting

In [58]:
```
from sklearn.ensemble import HistGradientBoostingClassifier
```

In [59]:
```
boost = HistGradientBoostingClassifier()
boost.fit(X_train, y_train)
```

Out[59]:
```
▾ HistGradientBoostingClassifier
HistGradientBoostingClassifier()
```

In [60]:
```
boost_predictions = boost.predict(X_test)
```

In [61]:
```
plotCM(y_test, boost_predictions)
```

```
accuracy Score is: 0.7781695849546044
```

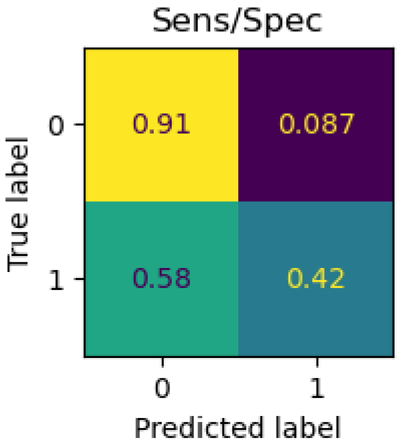

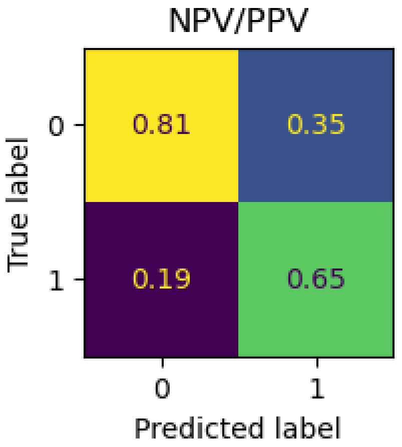

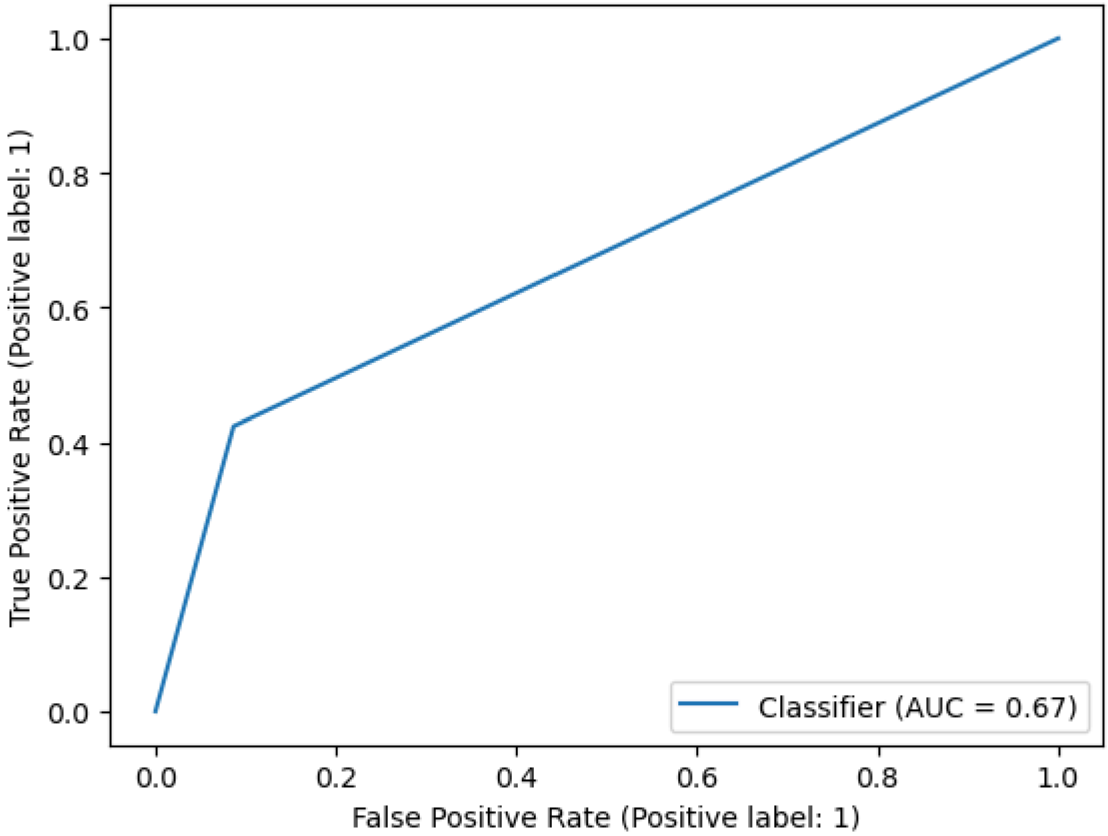

Attempt CatBoost

In [62]:
```
from catboost import CatBoostClassifier
import warnings
warnings.filterwarnings("ignore")
```

In [63]:
```
# Define the hyperparameters for the CatBoost algorithm
params = {'learning_rate': 0.1, 'depth': 6,
          'l2_leaf_reg': 3, 'iterations': 100}

# Initialize the CatBoostClassifier object
# with the defined hyperparameters and fit it on the training set
model = CatBoostClassifier(**params)
model.fit(X_train, y_train)
```

```
0:	learn: 0.6537430	total: 223ms	remaining: 22.1s
1:	learn: 0.6213722	total: 273ms	remaining: 13.4s
2:	learn: 0.5959228	total: 305ms	remaining: 9.85s
3:	learn: 0.5771781	total: 346ms	remaining: 8.31s
4:	learn: 0.5607714	total: 380ms	remaining: 7.23s
5:	learn: 0.5476268	total: 413ms	remaining: 6.48s
6:	learn: 0.5379610	total: 469ms	remaining: 6.22s
7:	learn: 0.5301161	total: 505ms	remaining: 5.81s
8:	learn: 0.5236230	total: 550ms	remaining: 5.56s
9:	learn: 0.5184433	total: 592ms	remaining: 5.33s
10:	learn: 0.5133633	total: 648ms	remaining: 5.24s
11:	learn: 0.5096727	total: 710ms	remaining: 5.21s
12:	learn: 0.5061846	total: 757ms	remaining: 5.07s
13:	learn: 0.5031466	total: 789ms	remaining: 4.84s
14:	learn: 0.5003469	total: 827ms	remaining: 4.68s
15:	learn: 0.4977360	total: 861ms	remaining: 4.52s
16:	learn: 0.4959181	total: 890ms	remaining: 4.35s
17:	learn: 0.4941595	total: 921ms	remaining: 4.2s
18:	learn: 0.4930826	total: 950ms	remaining: 4.05s
19:	learn: 0.4919306	total: 974ms	remaining: 3.9s
20:	learn: 0.4905689	total: 998ms	remaining: 3.75s
21:	learn: 0.4894222	total: 1.02s	remaining: 3.63s
22:	learn: 0.4881210	total: 1.05s	remaining: 3.51s
23:	learn: 0.4868730	total: 1.07s	remaining: 3.4s
24:	learn: 0.4858173	total: 1.1s	remaining: 3.29s
25:	learn: 0.4849546	total: 1.12s	remaining: 3.19s
26:	learn: 0.4840698	total: 1.15s	remaining: 3.1s
27:	learn: 0.4833453	total: 1.17s	remaining: 3.01s
28:	learn: 0.4828975	total: 1.19s	remaining: 2.92s
29:	learn: 0.4824367	total: 1.21s	remaining: 2.83s
30:	learn: 0.4818337	total: 1.24s	remaining: 2.75s
31:	learn: 0.4811630	total: 1.26s	remaining: 2.67s
32:	learn: 0.4805654	total: 1.28s	remaining: 2.61s
33:	learn: 0.4799814	total: 1.31s	remaining: 2.54s
34:	learn: 0.4794950	total: 1.33s	remaining: 2.47s
35:	learn: 0.4790256	total: 1.35s	remaining: 2.41s
36:	learn: 0.4786289	total: 1.39s	remaining: 2.37s
37:	learn: 0.4781916	total: 1.42s	remaining: 2.32s
38:	learn: 0.4779381	total: 1.45s	remaining: 2.27s
39:	learn: 0.4774470	total: 1.47s	remaining: 2.21s
40:	learn: 0.4771962	total: 1.5s	remaining: 2.15s
41:	learn: 0.4769796	total: 1.52s	remaining: 2.1s
42:	learn: 0.4766436	total: 1.54s	remaining: 2.04s
43:	learn: 0.4762937	total: 1.57s	remaining: 2s
44:	learn: 0.4758800	total: 1.6s	remaining: 1.95s
45:	learn: 0.4756426	total: 1.63s	remaining: 1.91s
46:	learn: 0.4752276	total: 1.66s	remaining: 1.87s
47:	learn: 0.4746998	total: 1.68s	remaining: 1.82s
48:	learn: 0.4744771	total: 1.71s	remaining: 1.78s
49:	learn: 0.4742948	total: 1.74s	remaining: 1.74s
50:	learn: 0.4739748	total: 1.76s	remaining: 1.7s
51:	learn: 0.4736635	total: 1.79s	remaining: 1.65s
52:	learn: 0.4735408	total: 1.82s	remaining: 1.61s
53:	learn: 0.4732556	total: 1.84s	remaining: 1.57s
54:	learn: 0.4730195	total: 1.87s	remaining: 1.53s
55:	learn: 0.4726302	total: 1.9s	remaining: 1.49s
56:	learn: 0.4723989	total: 1.92s	remaining: 1.45s
57:	learn: 0.4721940	total: 1.95s	remaining: 1.41s
58:	learn: 0.4720574	total: 1.97s	remaining: 1.37s
59:	learn: 0.4718501	total: 2.01s	remaining: 1.34s
```

```
60:	learn: 0.4717436	total: 2.04s	remaining: 1.3s
61:	learn: 0.4714665	total: 2.06s	remaining: 1.26s
62:	learn: 0.4712631	total: 2.08s	remaining: 1.22s
63:	learn: 0.4711060	total: 2.11s	remaining: 1.19s
64:	learn: 0.4709403	total: 2.13s	remaining: 1.15s
65:	learn: 0.4708251	total: 2.16s	remaining: 1.11s
66:	learn: 0.4705953	total: 2.18s	remaining: 1.07s
67:	learn: 0.4703029	total: 2.2s	remaining: 1.04s
68:	learn: 0.4701218	total: 2.23s	remaining: 1000ms
69:	learn: 0.4699797	total: 2.25s	remaining: 963ms
70:	learn: 0.4697769	total: 2.27s	remaining: 928ms
71:	learn: 0.4695907	total: 2.29s	remaining: 893ms
72:	learn: 0.4694843	total: 2.31s	remaining: 857ms
73:	learn: 0.4693154	total: 2.34s	remaining: 821ms
74:	learn: 0.4691394	total: 2.36s	remaining: 786ms
75:	learn: 0.4690337	total: 2.38s	remaining: 752ms
76:	learn: 0.4687706	total: 2.4s	remaining: 718ms
77:	learn: 0.4686706	total: 2.42s	remaining: 684ms
78:	learn: 0.4683915	total: 2.45s	remaining: 651ms
79:	learn: 0.4681463	total: 2.48s	remaining: 619ms
80:	learn: 0.4679283	total: 2.5s	remaining: 587ms
81:	learn: 0.4677904	total: 2.53s	remaining: 555ms
82:	learn: 0.4676816	total: 2.55s	remaining: 523ms
83:	learn: 0.4674883	total: 2.59s	remaining: 493ms
84:	learn: 0.4672445	total: 2.61s	remaining: 461ms
85:	learn: 0.4671368	total: 2.65s	remaining: 431ms
86:	learn: 0.4670317	total: 2.69s	remaining: 402ms
87:	learn: 0.4669530	total: 2.71s	remaining: 370ms
88:	learn: 0.4668567	total: 2.74s	remaining: 338ms
89:	learn: 0.4666439	total: 2.76s	remaining: 307ms
90:	learn: 0.4664647	total: 2.79s	remaining: 276ms
91:	learn: 0.4663810	total: 2.81s	remaining: 244ms
92:	learn: 0.4662763	total: 2.83s	remaining: 213ms
93:	learn: 0.4661452	total: 2.86s	remaining: 183ms
94:	learn: 0.4660469	total: 2.89s	remaining: 152ms
95:	learn: 0.4657948	total: 2.91s	remaining: 121ms
96:	learn: 0.4656752	total: 2.94s	remaining: 90.8ms
97:	learn: 0.4655953	total: 2.96s	remaining: 60.5ms
98:	learn: 0.4654612	total: 2.99s	remaining: 30.2ms
99:	learn: 0.4653632	total: 3.01s	remaining: 0us
```

Out[63]:

```
<catboost.core.CatBoostClassifier at 0x17c1d2d6a10>
```

In [64]:

```python
y_pred = model.predict(X_test)
```

In [65]:

```python
plotCM(y_test, y_pred)
```

```
accuracy Score is: 0.7731841763942932
```

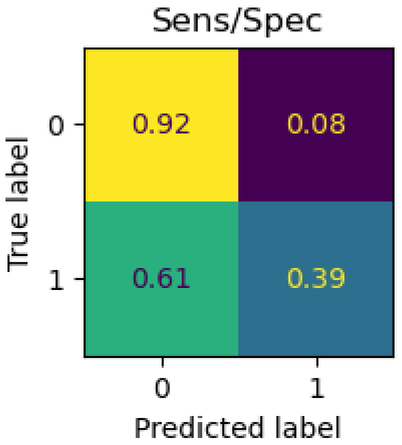

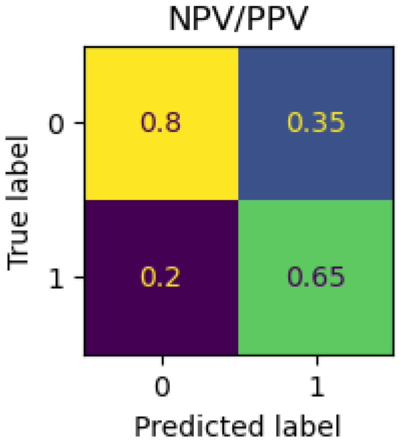

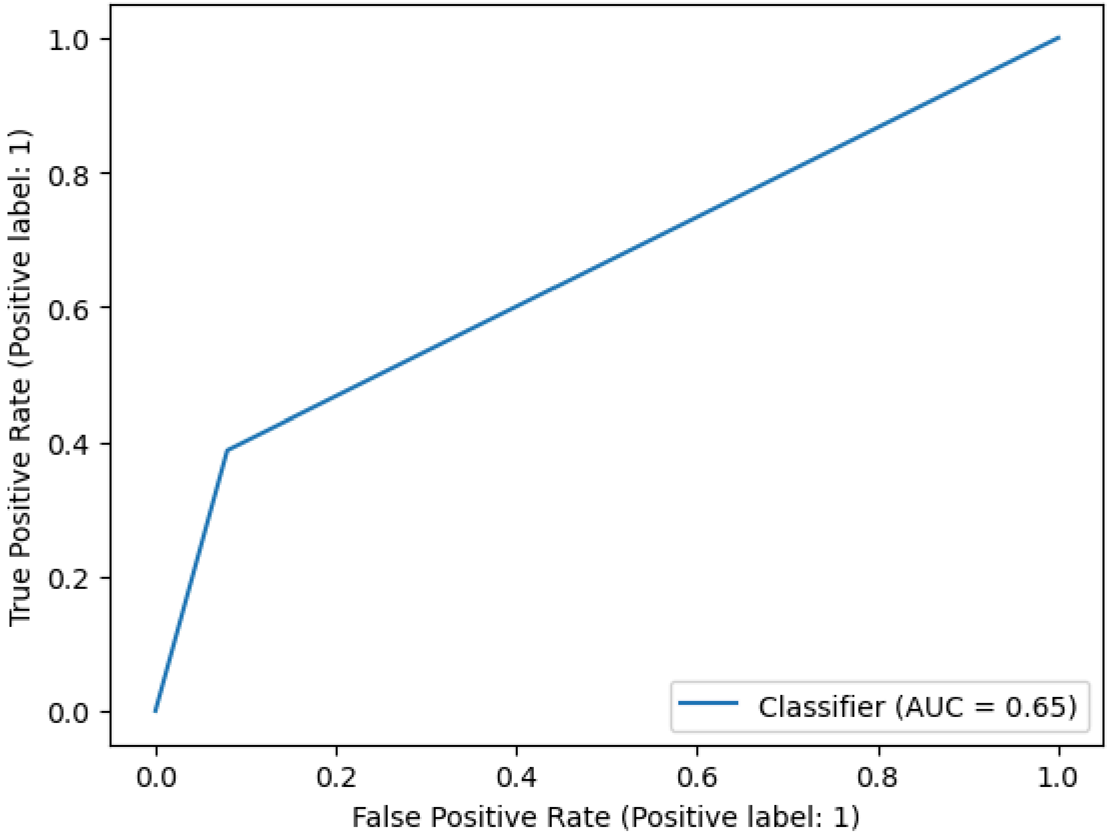

Neural Network

split Training data again in 75:25 ration to generate a total 60:20:20 split for Train:Validate:Test

In [66]:
```
X_train, X_val, y_train, y_val = train_test_split(X_train, y_train, test_size=0.25, ra
```

In [67]:
```
# Define function to plot Training Loss vs Validation Loss across epochs
def drawPlot():
    plt.plot(history.history['loss'])
    plt.plot(history.history['val_loss'])
    plt.title('model Loss')
    plt.ylabel('Loss')
    plt.xlabel('Epoch')
    #plt.xticks(np.arange(0, 21, 1.0))
    plt.legend(['train', 'val'], loc='upper left')
    plt.show()
```

In [74]:
```
drawPlot()
```

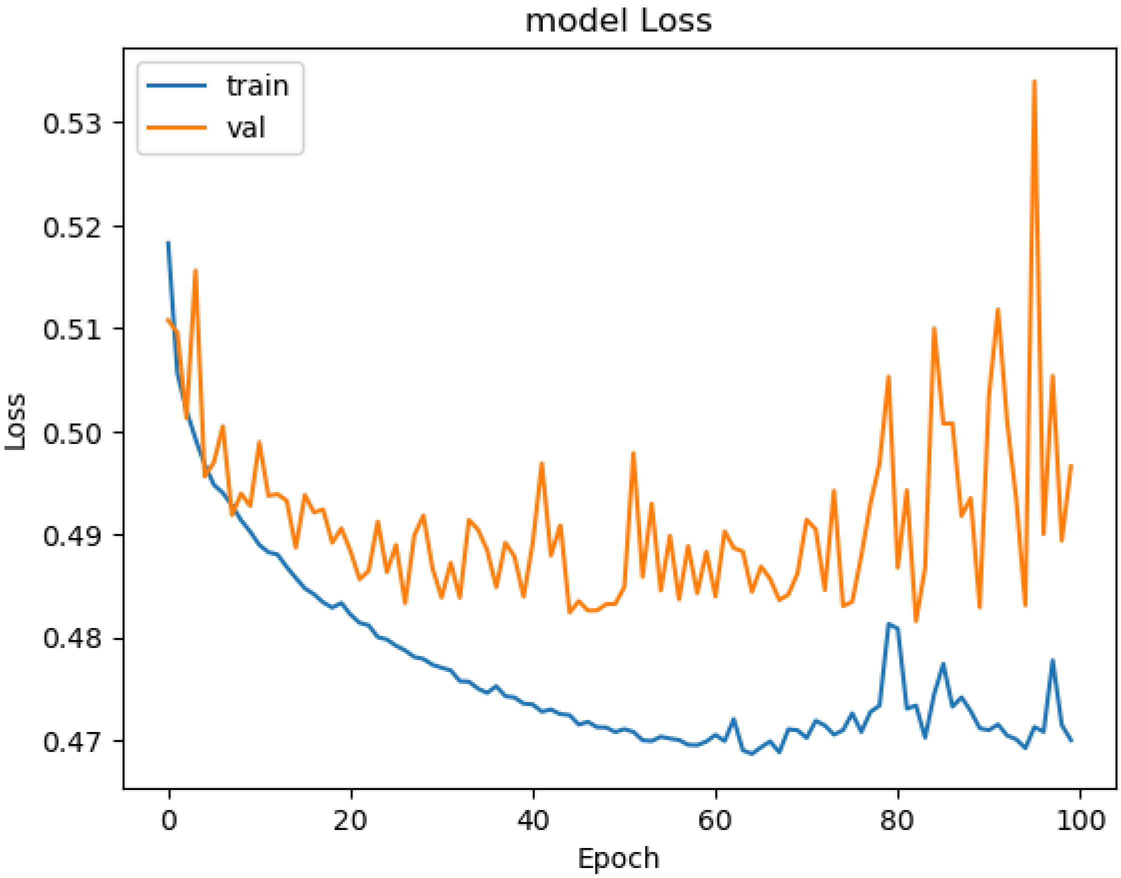

In [76]:
```
network = models.Sequential()
network.add(layers.Dense(512, activation='relu', input_shape=(23, )))
network.add(layers.Dense(256, activation='relu'))
network.add(layers.Dense(128, activation='relu'))
network.add(layers.Dense(64, activation='relu'))
network.add(layers.Dense(32, activation='relu'))
network.add(layers.Dense(16, activation='relu'))
network.add(layers.Dense(8, activation='relu'))
network.add(layers.Dense(4, activation='relu'))
network.add(layers.Dense(2, activation='relu'))
network.add(layers.Dense(1, activation='sigmoid'))
network.compile(optimizer=optimizers.RMSprop(),
```

```
                 loss='binary_crossentropy',
                 metrics=['accuracy'])
```

In [73]:
```
# fit model
history = network.fit(X_train, y_train,
                      batch_size=64, epochs=100,
                      validation_data = (X_val, y_val))
```

```
Epoch 1/100
1157/1157 [==============================] - 6s 4ms/step - loss: 0.5182 - accuracy:
0.7457 - val_loss: 0.5108 - val_accuracy: 0.7510
Epoch 2/100
1157/1157 [==============================] - 5s 4ms/step - loss: 0.5057 - accuracy:
0.7525 - val_loss: 0.5096 - val_accuracy: 0.7469
Epoch 3/100
1157/1157 [==============================] - 5s 4ms/step - loss: 0.5020 - accuracy:
0.7547 - val_loss: 0.5013 - val_accuracy: 0.7550
Epoch 4/100
1157/1157 [==============================] - 5s 4ms/step - loss: 0.4992 - accuracy:
0.7566 - val_loss: 0.5156 - val_accuracy: 0.7537
Epoch 5/100
1157/1157 [==============================] - 5s 4ms/step - loss: 0.4968 - accuracy:
0.7583 - val_loss: 0.4956 - val_accuracy: 0.7607
Epoch 6/100
1157/1157 [==============================] - 5s 4ms/step - loss: 0.4948 - accuracy:
0.7600 - val_loss: 0.4970 - val_accuracy: 0.7567
Epoch 7/100
1157/1157 [==============================] - 5s 4ms/step - loss: 0.4940 - accuracy:
0.7613 - val_loss: 0.5005 - val_accuracy: 0.7584
Epoch 8/100
1157/1157 [==============================] - 6s 5ms/step - loss: 0.4928 - accuracy:
0.7611 - val_loss: 0.4919 - val_accuracy: 0.7632
Epoch 9/100
1157/1157 [==============================] - 4s 4ms/step - loss: 0.4914 - accuracy:
0.7626 - val_loss: 0.4940 - val_accuracy: 0.7616
Epoch 10/100
1157/1157 [==============================] - 4s 4ms/step - loss: 0.4903 - accuracy:
0.7615 - val_loss: 0.4928 - val_accuracy: 0.7598
Epoch 11/100
1157/1157 [==============================] - 5s 4ms/step - loss: 0.4890 - accuracy:
0.7645 - val_loss: 0.4989 - val_accuracy: 0.7579
Epoch 12/100
1157/1157 [==============================] - 5s 4ms/step - loss: 0.4883 - accuracy:
0.7646 - val_loss: 0.4938 - val_accuracy: 0.7573
Epoch 13/100
1157/1157 [==============================] - 5s 4ms/step - loss: 0.4881 - accuracy:
0.7636 - val_loss: 0.4939 - val_accuracy: 0.7621
Epoch 14/100
1157/1157 [==============================] - 5s 5ms/step - loss: 0.4869 - accuracy:
0.7650 - val_loss: 0.4933 - val_accuracy: 0.7598
Epoch 15/100
1157/1157 [==============================] - 5s 4ms/step - loss: 0.4858 - accuracy:
0.7666 - val_loss: 0.4887 - val_accuracy: 0.7624
Epoch 16/100
1157/1157 [==============================] - 5s 5ms/step - loss: 0.4848 - accuracy:
0.7665 - val_loss: 0.4939 - val_accuracy: 0.7580
Epoch 17/100
1157/1157 [==============================] - 5s 4ms/step - loss: 0.4842 - accuracy:
0.7672 - val_loss: 0.4921 - val_accuracy: 0.7618
Epoch 18/100
1157/1157 [==============================] - 5s 4ms/step - loss: 0.4834 - accuracy:
0.7668 - val_loss: 0.4924 - val_accuracy: 0.7643
Epoch 19/100
1157/1157 [==============================] - 5s 4ms/step - loss: 0.4829 - accuracy:
0.7673 - val_loss: 0.4892 - val_accuracy: 0.7630
Epoch 20/100
1157/1157 [==============================] - 5s 4ms/step - loss: 0.4834 - accuracy:
0.7680 - val_loss: 0.4906 - val_accuracy: 0.7601
```

```
Epoch 21/100
1157/1157 [==============================] - 4s 4ms/step - loss: 0.4822 - accuracy: 0.7683 - val_loss: 0.4884 - val_accuracy: 0.7646
Epoch 22/100
1157/1157 [==============================] - 5s 4ms/step - loss: 0.4814 - accuracy: 0.7694 - val_loss: 0.4856 - val_accuracy: 0.7655
Epoch 23/100
1157/1157 [==============================] - 5s 4ms/step - loss: 0.4812 - accuracy: 0.7689 - val_loss: 0.4865 - val_accuracy: 0.7663
Epoch 24/100
1157/1157 [==============================] - 5s 4ms/step - loss: 0.4800 - accuracy: 0.7688 - val_loss: 0.4913 - val_accuracy: 0.7628
Epoch 25/100
1157/1157 [==============================] - 5s 4ms/step - loss: 0.4798 - accuracy: 0.7694 - val_loss: 0.4864 - val_accuracy: 0.7666
Epoch 26/100
1157/1157 [==============================] - 5s 4ms/step - loss: 0.4792 - accuracy: 0.7694 - val_loss: 0.4890 - val_accuracy: 0.7639
Epoch 27/100
1157/1157 [==============================] - 5s 5ms/step - loss: 0.4787 - accuracy: 0.7702 - val_loss: 0.4833 - val_accuracy: 0.7678
Epoch 28/100
1157/1157 [==============================] - 5s 4ms/step - loss: 0.4781 - accuracy: 0.7709 - val_loss: 0.4899 - val_accuracy: 0.7665
Epoch 29/100
1157/1157 [==============================] - 5s 4ms/step - loss: 0.4779 - accuracy: 0.7709 - val_loss: 0.4919 - val_accuracy: 0.7661
Epoch 30/100
1157/1157 [==============================] - 5s 4ms/step - loss: 0.4774 - accuracy: 0.7710 - val_loss: 0.4867 - val_accuracy: 0.7652
Epoch 31/100
1157/1157 [==============================] - 6s 5ms/step - loss: 0.4771 - accuracy: 0.7714 - val_loss: 0.4839 - val_accuracy: 0.7675
Epoch 32/100
1157/1157 [==============================] - 5s 4ms/step - loss: 0.4768 - accuracy: 0.7718 - val_loss: 0.4873 - val_accuracy: 0.7658
Epoch 33/100
1157/1157 [==============================] - 4s 4ms/step - loss: 0.4758 - accuracy: 0.7717 - val_loss: 0.4839 - val_accuracy: 0.7656
Epoch 34/100
1157/1157 [==============================] - 6s 5ms/step - loss: 0.4757 - accuracy: 0.7717 - val_loss: 0.4914 - val_accuracy: 0.7643
Epoch 35/100
1157/1157 [==============================] - 5s 5ms/step - loss: 0.4750 - accuracy: 0.7729 - val_loss: 0.4905 - val_accuracy: 0.7612
Epoch 36/100
1157/1157 [==============================] - 5s 4ms/step - loss: 0.4746 - accuracy: 0.7734 - val_loss: 0.4885 - val_accuracy: 0.7660
Epoch 37/100
1157/1157 [==============================] - 6s 5ms/step - loss: 0.4753 - accuracy: 0.7719 - val_loss: 0.4849 - val_accuracy: 0.7670
Epoch 38/100
1157/1157 [==============================] - 5s 4ms/step - loss: 0.4743 - accuracy: 0.7732 - val_loss: 0.4892 - val_accuracy: 0.7578
Epoch 39/100
1157/1157 [==============================] - 6s 5ms/step - loss: 0.4742 - accuracy: 0.7727 - val_loss: 0.4879 - val_accuracy: 0.7628
Epoch 40/100
1157/1157 [==============================] - 7s 6ms/step - loss: 0.4736 - accuracy: 0.7734 - val_loss: 0.4840 - val_accuracy: 0.7667
```

```
Epoch 41/100
1157/1157 [==============================] - 5s 5ms/step - loss: 0.4735 - accuracy: 0.7733 - val_loss: 0.4892 - val_accuracy: 0.7633
Epoch 42/100
1157/1157 [==============================] - 6s 5ms/step - loss: 0.4728 - accuracy: 0.7741 - val_loss: 0.4969 - val_accuracy: 0.7622
Epoch 43/100
1157/1157 [==============================] - 5s 5ms/step - loss: 0.4730 - accuracy: 0.7737 - val_loss: 0.4880 - val_accuracy: 0.7622
Epoch 44/100
1157/1157 [==============================] - 5s 5ms/step - loss: 0.4726 - accuracy: 0.7742 - val_loss: 0.4909 - val_accuracy: 0.7632
Epoch 45/100
1157/1157 [==============================] - 5s 4ms/step - loss: 0.4725 - accuracy: 0.7742 - val_loss: 0.4824 - val_accuracy: 0.7650
Epoch 46/100
1157/1157 [==============================] - 5s 5ms/step - loss: 0.4715 - accuracy: 0.7749 - val_loss: 0.4835 - val_accuracy: 0.7663
Epoch 47/100
1157/1157 [==============================] - 5s 4ms/step - loss: 0.4718 - accuracy: 0.7756 - val_loss: 0.4826 - val_accuracy: 0.7664
Epoch 48/100
1157/1157 [==============================] - 5s 4ms/step - loss: 0.4713 - accuracy: 0.7746 - val_loss: 0.4826 - val_accuracy: 0.7674
Epoch 49/100
1157/1157 [==============================] - 5s 4ms/step - loss: 0.4713 - accuracy: 0.7750 - val_loss: 0.4832 - val_accuracy: 0.7654
Epoch 50/100
1157/1157 [==============================] - 5s 5ms/step - loss: 0.4708 - accuracy: 0.7755 - val_loss: 0.4833 - val_accuracy: 0.7671
Epoch 51/100
1157/1157 [==============================] - 4s 4ms/step - loss: 0.4711 - accuracy: 0.7738 - val_loss: 0.4849 - val_accuracy: 0.7655
Epoch 52/100
1157/1157 [==============================] - 5s 4ms/step - loss: 0.4708 - accuracy: 0.7754 - val_loss: 0.4979 - val_accuracy: 0.7384
Epoch 53/100
1157/1157 [==============================] - 5s 4ms/step - loss: 0.4700 - accuracy: 0.7750 - val_loss: 0.4859 - val_accuracy: 0.7682
Epoch 54/100
1157/1157 [==============================] - 5s 4ms/step - loss: 0.4700 - accuracy: 0.7749 - val_loss: 0.4930 - val_accuracy: 0.7673
Epoch 55/100
1157/1157 [==============================] - 6s 5ms/step - loss: 0.4704 - accuracy: 0.7746 - val_loss: 0.4846 - val_accuracy: 0.7660
Epoch 56/100
1157/1157 [==============================] - 5s 5ms/step - loss: 0.4702 - accuracy: 0.7756 - val_loss: 0.4899 - val_accuracy: 0.7605
Epoch 57/100
1157/1157 [==============================] - 5s 4ms/step - loss: 0.4700 - accuracy: 0.7744 - val_loss: 0.4837 - val_accuracy: 0.7663
Epoch 58/100
1157/1157 [==============================] - 5s 4ms/step - loss: 0.4696 - accuracy: 0.7756 - val_loss: 0.4889 - val_accuracy: 0.7652
Epoch 59/100
1157/1157 [==============================] - 5s 5ms/step - loss: 0.4696 - accuracy: 0.7768 - val_loss: 0.4843 - val_accuracy: 0.7647
Epoch 60/100
1157/1157 [==============================] - 5s 4ms/step - loss: 0.4699 - accuracy: 0.7751 - val_loss: 0.4883 - val_accuracy: 0.7581
```

```
Epoch 61/100
1157/1157 [==============================] - 5s 4ms/step - loss: 0.4705 - accuracy:
0.7733 - val_loss: 0.4840 - val_accuracy: 0.7629
Epoch 62/100
1157/1157 [==============================] - 6s 5ms/step - loss: 0.4699 - accuracy:
0.7762 - val_loss: 0.4903 - val_accuracy: 0.7661
Epoch 63/100
1157/1157 [==============================] - 5s 4ms/step - loss: 0.4721 - accuracy:
0.7751 - val_loss: 0.4887 - val_accuracy: 0.7648
Epoch 64/100
1157/1157 [==============================] - 4s 4ms/step - loss: 0.4691 - accuracy:
0.7764 - val_loss: 0.4884 - val_accuracy: 0.7671
Epoch 65/100
1157/1157 [==============================] - 4s 4ms/step - loss: 0.4687 - accuracy:
0.7773 - val_loss: 0.4844 - val_accuracy: 0.7671
Epoch 66/100
1157/1157 [==============================] - 4s 4ms/step - loss: 0.4694 - accuracy:
0.7754 - val_loss: 0.4869 - val_accuracy: 0.7660
Epoch 67/100
1157/1157 [==============================] - 5s 4ms/step - loss: 0.4699 - accuracy:
0.7748 - val_loss: 0.4857 - val_accuracy: 0.7654
Epoch 68/100
1157/1157 [==============================] - 4s 4ms/step - loss: 0.4689 - accuracy:
0.7757 - val_loss: 0.4836 - val_accuracy: 0.7670
Epoch 69/100
1157/1157 [==============================] - 4s 4ms/step - loss: 0.4711 - accuracy:
0.7748 - val_loss: 0.4842 - val_accuracy: 0.7655
Epoch 70/100
1157/1157 [==============================] - 4s 3ms/step - loss: 0.4710 - accuracy:
0.7738 - val_loss: 0.4862 - val_accuracy: 0.7558
Epoch 71/100
1157/1157 [==============================] - 4s 4ms/step - loss: 0.4703 - accuracy:
0.7753 - val_loss: 0.4914 - val_accuracy: 0.7586
Epoch 72/100
1157/1157 [==============================] - 4s 4ms/step - loss: 0.4719 - accuracy:
0.7733 - val_loss: 0.4905 - val_accuracy: 0.7628
Epoch 73/100
1157/1157 [==============================] - 4s 4ms/step - loss: 0.4715 - accuracy:
0.7719 - val_loss: 0.4846 - val_accuracy: 0.7649
Epoch 74/100
1157/1157 [==============================] - 5s 4ms/step - loss: 0.4706 - accuracy:
0.7748 - val_loss: 0.4943 - val_accuracy: 0.7639
Epoch 75/100
1157/1157 [==============================] - 5s 4ms/step - loss: 0.4710 - accuracy:
0.7736 - val_loss: 0.4831 - val_accuracy: 0.7701
Epoch 76/100
1157/1157 [==============================] - 5s 4ms/step - loss: 0.4726 - accuracy:
0.7736 - val_loss: 0.4835 - val_accuracy: 0.7682
Epoch 77/100
1157/1157 [==============================] - 5s 4ms/step - loss: 0.4708 - accuracy:
0.7725 - val_loss: 0.4879 - val_accuracy: 0.7633
Epoch 78/100
1157/1157 [==============================] - 4s 4ms/step - loss: 0.4728 - accuracy:
0.7727 - val_loss: 0.4930 - val_accuracy: 0.7621
Epoch 79/100
1157/1157 [==============================] - 6s 5ms/step - loss: 0.4734 - accuracy:
0.7715 - val_loss: 0.4969 - val_accuracy: 0.7663
Epoch 80/100
1157/1157 [==============================] - 5s 4ms/step - loss: 0.4813 - accuracy:
0.7673 - val_loss: 0.5053 - val_accuracy: 0.7646
```

```
Epoch 81/100
1157/1157 [==============================] - 5s 4ms/step - loss: 0.4809 - accuracy: 
0.7702 - val_loss: 0.4868 - val_accuracy: 0.7672
Epoch 82/100
1157/1157 [==============================] - 4s 4ms/step - loss: 0.4731 - accuracy: 
0.7749 - val_loss: 0.4943 - val_accuracy: 0.7642
Epoch 83/100
1157/1157 [==============================] - 4s 4ms/step - loss: 0.4734 - accuracy: 
0.7741 - val_loss: 0.4816 - val_accuracy: 0.7691
Epoch 84/100
1157/1157 [==============================] - 4s 4ms/step - loss: 0.4703 - accuracy: 
0.7756 - val_loss: 0.4868 - val_accuracy: 0.7665
Epoch 85/100
1157/1157 [==============================] - 4s 4ms/step - loss: 0.4745 - accuracy: 
0.7721 - val_loss: 0.5100 - val_accuracy: 0.7468
Epoch 86/100
1157/1157 [==============================] - 5s 4ms/step - loss: 0.4774 - accuracy: 
0.7715 - val_loss: 0.5007 - val_accuracy: 0.7697
Epoch 87/100
1157/1157 [==============================] - 5s 4ms/step - loss: 0.4733 - accuracy: 
0.7758 - val_loss: 0.5008 - val_accuracy: 0.7665
Epoch 88/100
1157/1157 [==============================] - 5s 4ms/step - loss: 0.4742 - accuracy: 
0.7751 - val_loss: 0.4918 - val_accuracy: 0.7686
Epoch 89/100
1157/1157 [==============================] - 5s 4ms/step - loss: 0.4729 - accuracy: 
0.7752 - val_loss: 0.4936 - val_accuracy: 0.7693
Epoch 90/100
1157/1157 [==============================] - 5s 4ms/step - loss: 0.4712 - accuracy: 
0.7743 - val_loss: 0.4829 - val_accuracy: 0.7661
Epoch 91/100
1157/1157 [==============================] - 6s 5ms/step - loss: 0.4710 - accuracy: 
0.7756 - val_loss: 0.5034 - val_accuracy: 0.7643
Epoch 92/100
1157/1157 [==============================] - 5s 5ms/step - loss: 0.4716 - accuracy: 
0.7747 - val_loss: 0.5118 - val_accuracy: 0.7687
Epoch 93/100
1157/1157 [==============================] - 5s 4ms/step - loss: 0.4705 - accuracy: 
0.7755 - val_loss: 0.5010 - val_accuracy: 0.7652
Epoch 94/100
1157/1157 [==============================] - 5s 4ms/step - loss: 0.4701 - accuracy: 
0.7742 - val_loss: 0.4935 - val_accuracy: 0.7643
Epoch 95/100
1157/1157 [==============================] - 4s 4ms/step - loss: 0.4693 - accuracy: 
0.7741 - val_loss: 0.4831 - val_accuracy: 0.7667
Epoch 96/100
1157/1157 [==============================] - 4s 3ms/step - loss: 0.4713 - accuracy: 
0.7753 - val_loss: 0.5340 - val_accuracy: 0.7684
Epoch 97/100
1157/1157 [==============================] - 4s 3ms/step - loss: 0.4708 - accuracy: 
0.7756 - val_loss: 0.4900 - val_accuracy: 0.7622
Epoch 98/100
1157/1157 [==============================] - 4s 4ms/step - loss: 0.4778 - accuracy: 
0.7696 - val_loss: 0.5054 - val_accuracy: 0.7658
Epoch 99/100
1157/1157 [==============================] - 5s 4ms/step - loss: 0.4715 - accuracy: 
0.7735 - val_loss: 0.4894 - val_accuracy: 0.7663
Epoch 100/100
1157/1157 [==============================] - 4s 4ms/step - loss: 0.4700 - accuracy: 
0.7757 - val_loss: 0.4966 - val_accuracy: 0.7612
```

In [75]:
```
# Concatenate Train and Validation Data
X_training = np.concatenate((X_train, X_val), axis=0)
y_training = np.concatenate((y_train, y_val), axis=0)
```

In [77]:
```
# Re-train network on complete Training Data, with Epochs set at 25
network.fit(X_training, y_training,
            batch_size=64, epochs=60)
```

```
Epoch 1/60
1542/1542 [==============================] - 7s 4ms/step - loss: 0.5168 - accuracy:
0.7472
Epoch 2/60
1542/1542 [==============================] - 5s 3ms/step - loss: 0.5041 - accuracy:
0.7541
Epoch 3/60
1542/1542 [==============================] - 6s 4ms/step - loss: 0.5000 - accuracy:
0.7568
Epoch 4/60
1542/1542 [==============================] - 6s 4ms/step - loss: 0.4974 - accuracy:
0.7585
Epoch 5/60
1542/1542 [==============================] - 6s 4ms/step - loss: 0.4954 - accuracy:
0.7602
Epoch 6/60
1542/1542 [==============================] - 6s 4ms/step - loss: 0.4936 - accuracy:
0.7612
Epoch 7/60
1542/1542 [==============================] - 7s 4ms/step - loss: 0.4916 - accuracy:
0.7622
Epoch 8/60
1542/1542 [==============================] - 5s 3ms/step - loss: 0.4896 - accuracy:
0.7637
Epoch 9/60
1542/1542 [==============================] - 5s 3ms/step - loss: 0.4883 - accuracy:
0.7642
Epoch 10/60
1542/1542 [==============================] - 5s 3ms/step - loss: 0.4872 - accuracy:
0.7650
Epoch 11/60
1542/1542 [==============================] - 5s 3ms/step - loss: 0.4859 - accuracy:
0.7665
Epoch 12/60
1542/1542 [==============================] - 8s 5ms/step - loss: 0.4850 - accuracy:
0.7661
Epoch 13/60
1542/1542 [==============================] - 8s 5ms/step - loss: 0.4840 - accuracy:
0.7675
Epoch 14/60
1542/1542 [==============================] - 10s 6ms/step - loss: 0.4835 - accuracy:
0.7681
Epoch 15/60
1542/1542 [==============================] - 7s 5ms/step - loss: 0.4826 - accuracy:
0.7671
Epoch 16/60
1542/1542 [==============================] - 7s 4ms/step - loss: 0.4819 - accuracy:
0.7685
Epoch 17/60
1542/1542 [==============================] - 6s 4ms/step - loss: 0.4815 - accuracy:
0.7688
Epoch 18/60
1542/1542 [==============================] - 7s 5ms/step - loss: 0.4808 - accuracy:
0.7695
Epoch 19/60
1542/1542 [==============================] - 6s 4ms/step - loss: 0.4800 - accuracy:
0.7697
Epoch 20/60
1542/1542 [==============================] - 5s 3ms/step - loss: 0.4793 - accuracy:
0.7705
```

```
Epoch 21/60
1542/1542 [==============================] - 5s 3ms/step - loss: 0.4792 - accuracy:
0.7704
Epoch 22/60
1542/1542 [==============================] - 5s 3ms/step - loss: 0.4785 - accuracy:
0.7707
Epoch 23/60
1542/1542 [==============================] - 5s 3ms/step - loss: 0.4782 - accuracy:
0.7713
Epoch 24/60
1542/1542 [==============================] - 4s 3ms/step - loss: 0.4777 - accuracy:
0.7708
Epoch 25/60
1542/1542 [==============================] - 5s 3ms/step - loss: 0.4770 - accuracy:
0.7725
Epoch 26/60
1542/1542 [==============================] - 6s 4ms/step - loss: 0.4769 - accuracy:
0.7724
Epoch 27/60
1542/1542 [==============================] - 5s 3ms/step - loss: 0.4764 - accuracy:
0.7720
Epoch 28/60
1542/1542 [==============================] - 6s 4ms/step - loss: 0.4760 - accuracy:
0.7721
Epoch 29/60
1542/1542 [==============================] - 5s 4ms/step - loss: 0.4757 - accuracy:
0.7717
Epoch 30/60
1542/1542 [==============================] - 6s 4ms/step - loss: 0.4752 - accuracy:
0.7729
Epoch 31/60
1542/1542 [==============================] - 6s 4ms/step - loss: 0.4753 - accuracy:
0.7734
Epoch 32/60
1542/1542 [==============================] - 6s 4ms/step - loss: 0.4746 - accuracy:
0.7737
Epoch 33/60
1542/1542 [==============================] - 5s 4ms/step - loss: 0.4742 - accuracy:
0.7741
Epoch 34/60
1542/1542 [==============================] - 6s 4ms/step - loss: 0.4744 - accuracy:
0.7732
Epoch 35/60
1542/1542 [==============================] - 7s 4ms/step - loss: 0.4742 - accuracy:
0.7732
Epoch 36/60
1542/1542 [==============================] - 6s 4ms/step - loss: 0.4735 - accuracy:
0.7747
Epoch 37/60
1542/1542 [==============================] - 6s 4ms/step - loss: 0.4733 - accuracy:
0.7742
Epoch 38/60
1542/1542 [==============================] - 6s 4ms/step - loss: 0.4729 - accuracy:
0.7745
Epoch 39/60
1542/1542 [==============================] - 6s 4ms/step - loss: 0.4721 - accuracy:
0.7753
Epoch 40/60
1542/1542 [==============================] - 6s 4ms/step - loss: 0.4721 - accuracy:
0.7749
```

```
Epoch 41/60
1542/1542 [==============================] - 6s 4ms/step - loss: 0.4723 - accuracy:
0.7755
Epoch 42/60
1542/1542 [==============================] - 7s 4ms/step - loss: 0.4720 - accuracy:
0.7752
Epoch 43/60
1542/1542 [==============================] - 7s 5ms/step - loss: 0.4718 - accuracy:
0.7754
Epoch 44/60
1542/1542 [==============================] - 7s 4ms/step - loss: 0.4714 - accuracy:
0.7755
Epoch 45/60
1542/1542 [==============================] - 7s 5ms/step - loss: 0.4710 - accuracy:
0.7761
Epoch 46/60
1542/1542 [==============================] - 8s 5ms/step - loss: 0.4706 - accuracy:
0.7758
Epoch 47/60
1542/1542 [==============================] - 8s 5ms/step - loss: 0.4702 - accuracy:
0.7762
Epoch 48/60
1542/1542 [==============================] - 8s 5ms/step - loss: 0.4698 - accuracy:
0.7769
Epoch 49/60
1542/1542 [==============================] - 8s 5ms/step - loss: 0.4699 - accuracy:
0.7767
Epoch 50/60
1542/1542 [==============================] - 7s 5ms/step - loss: 0.4695 - accuracy:
0.7767
Epoch 51/60
1542/1542 [==============================] - 8s 5ms/step - loss: 0.4691 - accuracy:
0.7777
Epoch 52/60
1542/1542 [==============================] - 8s 5ms/step - loss: 0.4693 - accuracy:
0.7771
Epoch 53/60
1542/1542 [==============================] - 8s 5ms/step - loss: 0.4688 - accuracy:
0.7785
Epoch 54/60
1542/1542 [==============================] - 7s 4ms/step - loss: 0.4686 - accuracy:
0.7770
Epoch 55/60
1542/1542 [==============================] - 6s 4ms/step - loss: 0.4686 - accuracy:
0.7779
Epoch 56/60
1542/1542 [==============================] - 5s 4ms/step - loss: 0.4683 - accuracy:
0.7779
Epoch 57/60
1542/1542 [==============================] - 5s 3ms/step - loss: 0.4676 - accuracy:
0.7790
Epoch 58/60
1542/1542 [==============================] - 5s 3ms/step - loss: 0.4678 - accuracy:
0.7783
Epoch 59/60
1542/1542 [==============================] - 6s 4ms/step - loss: 0.4676 - accuracy:
0.7791
Epoch 60/60
1542/1542 [==============================] - 5s 3ms/step - loss: 0.4675 - accuracy:
0.7783
```

Out[77]: <keras.src.callbacks.History at 0x17c350e3850>

In [78]:
```
y_prediction = network.predict(X_test)
```

771/771 [==============================] - 2s 2ms/step

In [79]:
```
network.evaluate(X_test, y_test)
```

771/771 [==============================] - 3s 3ms/step - loss: 0.4822 - accuracy: 0.7
667

Out[79]: [0.48215046525001526, 0.7667396068572998]

In [80]:
```
y_prediction[y_prediction >= 0.5] = 1
y_prediction[y_prediction < 0.5] = 0
```

In [81]:
```
nnDF= pd.DataFrame(y_prediction)
```

In [82]:
```
nnDF.value_counts()
```

Out[82]:
```
0.0    19858
1.0     4814
Name: count, dtype: int64
```

In [83]:
```
plotCM(y_test, y_prediction)
```

accuracy Score is: 0.7667396238651103

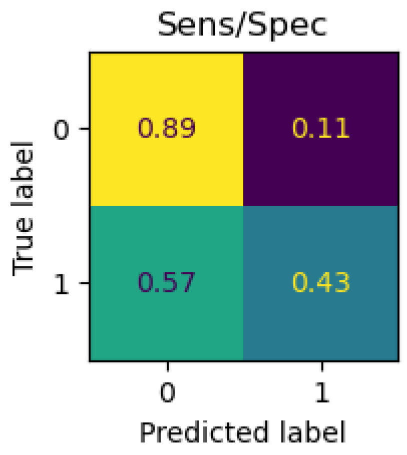

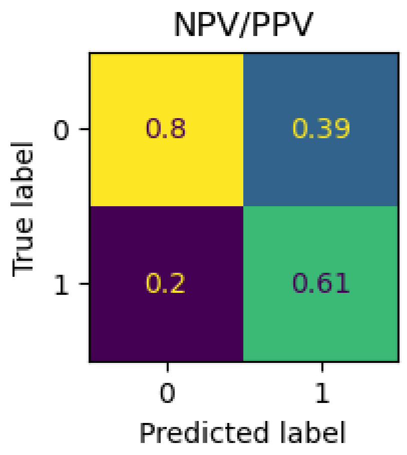

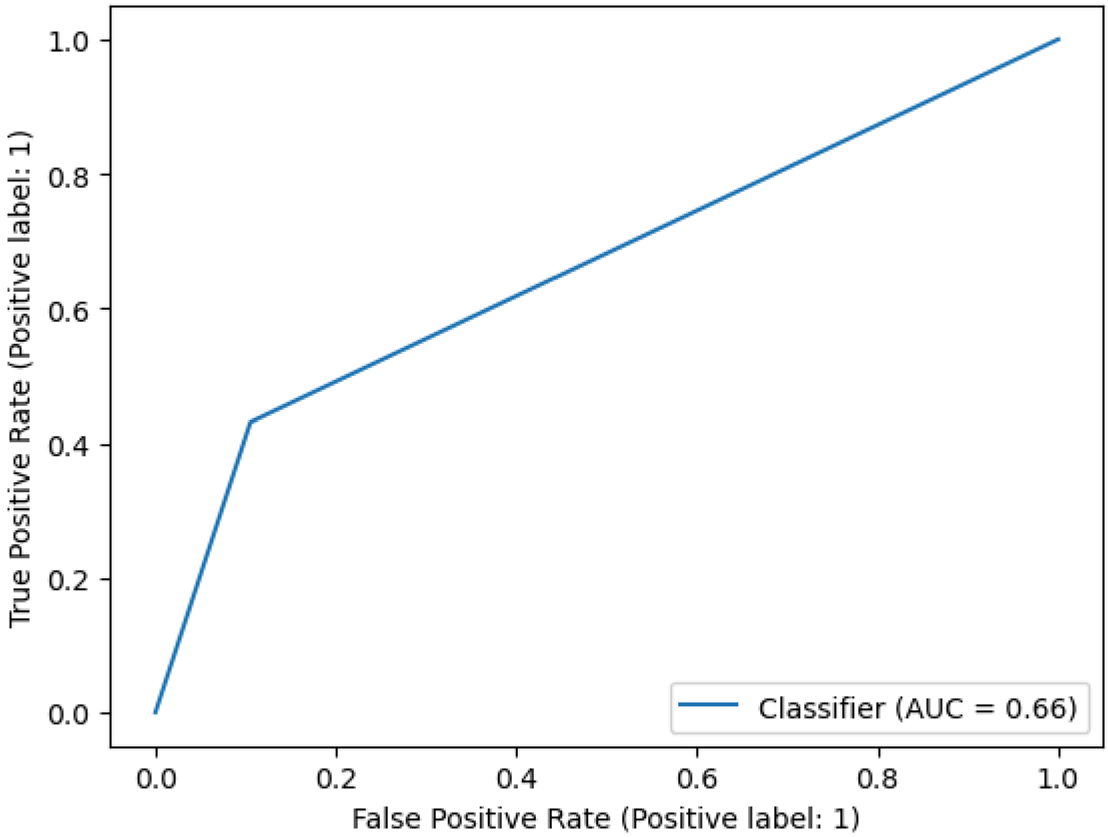
1.0
0.8
0.6
0.4
0.2
0.0
True Positive Rate (Positive label: 1)
Classifier (AUC = 0.66)
0.0
0.2
0.4
0.6
0.8
1.0
False Positive Rate (Positive label: 1)

Reimport files and re-process for 5 day threshold modeling

In [4]:
```
df5 = pd.read_csv('/Users/ganatrh/Downloads/DataWithPrimaryDiagnosis2.csv')
df70 = pd.read_csv('/Users/ganatrh/Downloads/PIM.csv')
```

In [5]:
```
df5 = df5.merge(df70, on='Case Index Id', how='left' )
```

In [6]:
```
df5.info()
```

```
<class 'pandas.core.frame.DataFrame'>
RangeIndex: 608512 entries, 0 to 608511
Data columns (total 78 columns):
 #   Column                                         Non-Null Count   Dtype
---  ------                                         --------------   -----
 0   Unit Id                                        608512 non-null  int64
 1   Case Id                                        608512 non-null  int64
 2   Case Index Id                                  608512 non-null  object
 3   Discharge Year                                 608512 non-null  int64
 4   Is Readmission                                 608512 non-null  int64
 5   Age                                            608512 non-null  object
 6   Weight (kg)                                    608512 non-null  float64
 7   Flag - Age/Weight                              608512 non-null  int64
 8   Collects height                                608512 non-null  int64
 9   Height (cm)                                    309701 non-null  float64
 10  Gender                                         608512 non-null  object
 11  Collects Race                                  608512 non-null  int64
 12  Race                                           550714 non-null  object
 13  Patient Origin                                 608512 non-null  object
 14  Trauma                                         608512 non-null  int64
 15  Patient Type                                   608512 non-null  object
 16  Collects Transport Team                        608512 non-null  int64
 17  Transport Team                                 371934 non-null  object
 18  Collects Transport Vehicle                     608512 non-null  int64
 19  Transport Vehicle                              347485 non-null  object
 20  Post Operative                                 608512 non-null  int64
 21  Collects Baseline PCPC/POPC                    608512 non-null  int64
 22  Baseline PCPC                                  127543 non-null  object
 23  Baseline POPC                                  127464 non-null  object
 24  Collects FSS                                   608512 non-null  int64
 25  Baseline FSS                                   59273 non-null   float64
 26  Cardiac Patient                                608512 non-null  int64
 27  Cardiac Procedure directly prior to or during stay  608512 non-null  int64
 28  Medical Length of Stay (days)                  584327 non-null  float64
 29  Physical Length of Stay (days)                 608512 non-null  float64
 30  Hospital LOS                                   606176 non-null  float64
 31  Outcome                                        608512 non-null  object
 32  Disposition                                    608512 non-null  object
 33  Collects Limitation on Care                    608512 non-null  int64
 34  Limitation on Care                             523209 non-null  float64
 35  Collects Brain Dead                            608512 non-null  int64
 36  Is Brain Dead                                  4922 non-null    float64
 37  Collects Altered Code                          608512 non-null  int64
 38  Is Altered Code                                523227 non-null  float64
 39  Collects Withdrawal of Support                 608512 non-null  int64
 40  Withdrawal of Support                          497357 non-null  float64
 41  Collects Autopsy                               608512 non-null  int64
 42  Was Autopsy Performed                          11602 non-null   object
 43  Collects Organ Donation                        608512 non-null  int64
 44  Is Organ Donor                                 2572 non-null    float64
 45  Collects Tissue Donation                       608512 non-null  int64
 46  Is Tissue Donor                                3443 non-null    float64
 47  Collects Donation after Cardiac Death          608512 non-null  int64
 48  Donation after Cardiac Death                   3563 non-null    float64
 49  Discharge PCPC                                 123029 non-null  object
 50  Discharge POPC                                 122965 non-null  object
 51  Discharge FSS                                  58085 non-null   float64
 52  Hospital Outcome                               604949 non-null  object
 53  Collects Diagnosis STS Codes                   608512 non-null  int64
 54  Collects Diagnosis ICD-9 Codes                 608512 non-null  int64
```

```
 55  PIM 3 Score                                      608512 non-null  float64
 56  PIM 3 Probability of Death                       608512 non-null  float64
 57  Mechanical Ventilation (First Hour)              608511 non-null  float64
 58  Elective Admission to ICU                        608511 non-null  float64
 59  PIM 3 Recovery from Surgery                      608511 non-null  object
 60  Category                                         608475 non-null  object
 61  Systolic Blood Pressure                          588367 non-null  float64
 62  Systolic Blood Pressure Unknown                  608511 non-null  float64
 63  PaO2 (mmHg)                                      13722 non-null   float64
 64  PaO2 (kPa)                                       13722 non-null   float64
 65  PaO2 Unknown                                     608511 non-null  float64
 66  FiO2                                             13722 non-null   float64
 67  FiO2 Unknown                                     608511 non-null  float64
 68  Base Excess                                      51063 non-null   float64
 69  Base Excess Unknown                              608511 non-null  float64
 70  PIM 3 Pupillary Reaction                         608511 non-null  object
 71  PIM 3 Pupillary Reaction Unknown                 608511 non-null  float64
 72  Admitted following Cardiac Bypass - PIM2/PIM3    608511 non-null  float64
 73  Admitted following Cardiac Bypass - PIM3 Baseline 608511 non-null  float64
 74  Admitted following Cardiac Bypass - PIM3 PostIndex 166 non-null     float64
 75  PIM 3 - No Very High Risk Dx                     608512 non-null  int64
 76  PIM 3 - No High Risk Dx                          608512 non-null  int64
 77  PIM 3 - No Low Risk Dx                           608512 non-null  int64
dtypes: float64(31), int64(28), object(19)
memory usage: 362.1+ MB
```

Remove rows that have flagged inaccurate weights

In [7]:
```
df6 = SmartDataframe(df5, config={"llm": llm})
df7 = df6.chat("Remove rows that have the value '1' in column 'Flag - Age/Weight' and
df7.info()
```

```
<class 'pandas.core.frame.DataFrame'>
RangeIndex: 607901 entries, 0 to 607900
Data columns (total 78 columns):
 #   Column                                           Non-Null Count   Dtype  
---  ------                                           --------------   -----  
 0   Unit Id                                          607901 non-null  int64  
 1   Case Id                                          607901 non-null  int64  
 2   Case Index Id                                    607901 non-null  object 
 3   Discharge Year                                   607901 non-null  int64  
 4   Is Readmission                                   607901 non-null  int64  
 5   Age                                              607901 non-null  object 
 6   Weight (kg)                                      607901 non-null  float64
 7   Flag - Age/Weight                                607901 non-null  int64  
 8   Collects height                                  607901 non-null  int64  
 9   Height (cm)                                      309407 non-null  float64
 10  Gender                                           607901 non-null  object 
 11  Collects Race                                    607901 non-null  int64  
 12  Race                                             550155 non-null  object 
 13  Patient Origin                                   607901 non-null  object 
 14  Trauma                                           607901 non-null  int64  
 15  Patient Type                                     607901 non-null  object 
 16  Collects Transport Team                          607901 non-null  int64  
 17  Transport Team                                   371523 non-null  object 
 18  Collects Transport Vehicle                       607901 non-null  int64  
 19  Transport Vehicle                                347101 non-null  object 
 20  Post Operative                                   607901 non-null  int64  
 21  Collects Baseline PCPC/POPC                      607901 non-null  int64  
 22  Baseline PCPC                                    127387 non-null  object 
 23  Baseline POPC                                    127309 non-null  object 
 24  Collects FSS                                     607901 non-null  int64  
 25  Baseline FSS                                     59184 non-null   float64
 26  Cardiac Patient                                  607901 non-null  int64  
 27  Cardiac Procedure directly prior to or during stay  607901 non-null  int64  
 28  Medical Length of Stay (days)                    583736 non-null  float64
 29  Physical Length of Stay (days)                   607901 non-null  float64
 30  Hospital LOS                                     605567 non-null  float64
 31  Outcome                                          607901 non-null  object 
 32  Disposition                                      607901 non-null  object 
 33  Collects Limitation on Care                      607901 non-null  int64  
 34  Limitation on Care                               522675 non-null  float64
 35  Collects Brain Dead                              607901 non-null  int64  
 36  Is Brain Dead                                    4918 non-null    float64
 37  Collects Altered Code                            607901 non-null  int64  
 38  Is Altered Code                                  522693 non-null  float64
 39  Collects Withdrawal of Support                   607901 non-null  int64  
 40  Withdrawal of Support                            496859 non-null  float64
 41  Collects Autopsy                                 607901 non-null  int64  
 42  Was Autopsy Performed                            11595 non-null   object 
 43  Collects Organ Donation                          607901 non-null  int64  
 44  Is Organ Donor                                   2572 non-null    float64
 45  Collects Tissue Donation                         607901 non-null  int64  
 46  Is Tissue Donor                                  3442 non-null    float64
 47  Collects Donation after Cardiac Death            607901 non-null  int64  
 48  Donation after Cardiac Death                     3562 non-null    float64
 49  Discharge PCPC                                   122879 non-null  object 
 50  Discharge POPC                                   122815 non-null  object 
 51  Discharge FSS                                    58000 non-null   float64
 52  Hospital Outcome                                 604344 non-null  object 
 53  Collects Diagnosis STS Codes                     607901 non-null  int64  
 54  Collects Diagnosis ICD-9 Codes                   607901 non-null  int64  
```

```
 55  PIM 3 Score                                        607901 non-null  float64
 56  PIM 3 Probability of Death                         607901 non-null  float64
 57  Mechanical Ventilation (First Hour)                607900 non-null  float64
 58  Elective Admission to ICU                          607900 non-null  float64
 59  PIM 3 Recovery from Surgery                        607900 non-null  object
 60  Category                                           607864 non-null  object
 61  Systolic Blood Pressure                            587779 non-null  float64
 62  Systolic Blood Pressure Unknown                    607900 non-null  float64
 63  PaO2 (mmHg)                                        13708 non-null   float64
 64  PaO2 (kPa)                                         13708 non-null   float64
 65  PaO2 Unknown                                       607900 non-null  float64
 66  FiO2                                               13708 non-null   float64
 67  FiO2 Unknown                                       607900 non-null  float64
 68  Base Excess                                        50991 non-null   float64
 69  Base Excess Unknown                                607900 non-null  float64
 70  PIM 3 Pupillary Reaction                           607900 non-null  object
 71  PIM 3 Pupillary Reaction Unknown                   607900 non-null  float64
 72  Admitted following Cardiac Bypass - PIM2/PIM3      607900 non-null  float64
 73  Admitted following Cardiac Bypass - PIM3 Baseline  607900 non-null  float64
 74  Admitted following Cardiac Bypass - PIM3 PostIndex 166 non-null     float64
 75  PIM 3 - No Very High Risk Dx                       607901 non-null  int64
 76  PIM 3 - No High Risk Dx                            607901 non-null  int64
 77  PIM 3 - No Low Risk Dx                             607901 non-null  int64
dtypes: float64(31), int64(28), object(19)
memory usage: 361.8+ MB
```

Create dataframe with Columns of interest

In [8]:

```
df8 = SmartDataframe(df7, config={"llm": llm})
df9 = df8.chat("Select the columns 'Case Index Id', 'Is Readmission', 'Age', 'Weight (
```

In [9]:

```
df9.info()
```

```
<class 'pandas.core.frame.DataFrame'>
RangeIndex: 607901 entries, 0 to 607900
Data columns (total 41 columns):
 #   Column                                           Non-Null Count   Dtype  
---  ------                                           --------------   -----  
 0   Case Index Id                                    607901 non-null  object 
 1   Is Readmission                                   607901 non-null  int64  
 2   Age                                              607901 non-null  object 
 3   Weight (kg)                                      607901 non-null  float64
 4   Gender                                           607901 non-null  object 
 5   Race                                             550155 non-null  object 
 6   Patient Origin                                   607901 non-null  object 
 7   Trauma                                           607901 non-null  int64  
 8   Patient Type                                     607901 non-null  object 
 9   Transport Team                                   371523 non-null  object 
 10  Transport Vehicle                                347101 non-null  object 
 11  Post Operative                                   607901 non-null  int64  
 12  Baseline PCPC                                    127387 non-null  object 
 13  Baseline POPC                                    127309 non-null  object 
 14  Baseline FSS                                     59184 non-null   float64
 15  Cardiac Patient                                  607901 non-null  int64  
 16  Cardiac Procedure directly prior to or during stay  607901 non-null  int64  
 17  Medical Length of Stay (days)                    583736 non-null  float64
 18  Physical Length of Stay (days)                   607901 non-null  float64
 19  Hospital LOS                                     605567 non-null  float64
 20  Outcome                                          607901 non-null  object 
 21  Disposition                                      607901 non-null  object 
 22  Is Altered Code                                  522693 non-null  float64
 23  Discharge PCPC                                   122879 non-null  object 
 24  Discharge POPC                                   122815 non-null  object 
 25  Discharge FSS                                    58000 non-null   float64
 26  Hospital Outcome                                 604344 non-null  object 
 27  PIM 3 Score                                      607901 non-null  float64
 28  PIM 3 Probability of Death                       607901 non-null  float64
 29  Mechanical Ventilation (First Hour)              607900 non-null  float64
 30  Elective Admission to ICU                        607900 non-null  float64
 31  PIM 3 Recovery from Surgery                      607900 non-null  object 
 32  Systolic Blood Pressure                          587779 non-null  float64
 33  PaO2 (mmHg)                                      13708 non-null   float64
 34  FiO2                                             13708 non-null   float64
 35  Base Excess                                      50991 non-null   float64
 36  PIM 3 Pupillary Reaction                         607900 non-null  object 
 37  PIM 3 - No Very High Risk Dx                     607901 non-null  int64  
 38  PIM 3 - No High Risk Dx                          607901 non-null  int64  
 39  PIM 3 - No Low Risk Dx                           607901 non-null  int64  
 40  Category                                         607864 non-null  object 
dtypes: float64(15), int64(8), object(18)
memory usage: 190.2+ MB
```

Remove rows that have NaN for "elective ICU admission" and for "POPC/PCPC". This comcomitantly removes NaN for other variables too

In [10]:
```
df9.dropna(subset=['Elective Admission to ICU', 'Baseline POPC', 'Baseline PCPC', 'Sys
df9.info()
```

```
<class 'pandas.core.frame.DataFrame'>
RangeIndex: 123485 entries, 0 to 123484
Data columns (total 41 columns):
 #   Column                                              Non-Null Count   Dtype  
---  ------                                              --------------   -----  
 0   Case Index Id                                       123485 non-null  object 
 1   Is Readmission                                      123485 non-null  int64  
 2   Age                                                 123485 non-null  object 
 3   Weight (kg)                                         123485 non-null  float64
 4   Gender                                              123485 non-null  object 
 5   Race                                                121785 non-null  object 
 6   Patient Origin                                      123485 non-null  object 
 7   Trauma                                              123485 non-null  int64  
 8   Patient Type                                        123485 non-null  object 
 9   Transport Team                                      98498 non-null   object 
 10  Transport Vehicle                                   104581 non-null  object 
 11  Post Operative                                      123485 non-null  int64  
 12  Baseline PCPC                                       123485 non-null  object 
 13  Baseline POPC                                       123485 non-null  object 
 14  Baseline FSS                                        16690 non-null   float64
 15  Cardiac Patient                                     123485 non-null  int64  
 16  Cardiac Procedure directly prior to or during stay  123485 non-null  int64  
 17  Medical Length of Stay (days)                       118321 non-null  float64
 18  Physical Length of Stay (days)                      123485 non-null  float64
 19  Hospital LOS                                        123245 non-null  float64
 20  Outcome                                             123485 non-null  object 
 21  Disposition                                         123485 non-null  object 
 22  Is Altered Code                                     122991 non-null  float64
 23  Discharge PCPC                                      118721 non-null  object 
 24  Discharge POPC                                      118695 non-null  object 
 25  Discharge FSS                                       16383 non-null   float64
 26  Hospital Outcome                                    122734 non-null  object 
 27  PIM 3 Score                                         123485 non-null  float64
 28  PIM 3 Probability of Death                          123485 non-null  float64
 29  Mechanical Ventilation (First Hour)                 123485 non-null  float64
 30  Elective Admission to ICU                           123485 non-null  float64
 31  PIM 3 Recovery from Surgery                         123485 non-null  object 
 32  Systolic Blood Pressure                             123485 non-null  float64
 33  PaO2 (mmHg)                                         3205 non-null    float64
 34  FiO2                                                3205 non-null    float64
 35  Base Excess                                         12155 non-null   float64
 36  PIM 3 Pupillary Reaction                            123485 non-null  object 
 37  PIM 3 - No Very High Risk Dx                        123485 non-null  int64  
 38  PIM 3 - No High Risk Dx                             123485 non-null  int64  
 39  PIM 3 - No Low Risk Dx                              123485 non-null  int64  
 40  Category                                            123485 non-null  object 
dtypes: float64(15), int64(8), object(18)
memory usage: 38.6+ MB
```

In [11]:
```
df9["Base Excess"] = df9["Base Excess"].replace(np.nan, 0)
df9["Base Excess"].value_counts()
```

Out[11]:
```
Base Excess
 0.0     111514
-3.0        358
-4.0        342
-5.0        329
-2.0        327
          ...
-16.5         1
 9.7          1
 23.3         1
-34.1         1
 11.3         1
Name: count, Length: 472, dtype: int64
```

The cells below consolidate all patients > 18 into one group

In [12]:
```
df9["Age"].value_counts()
```

Out[12]:
```
Age
Infant 29 days to < 2 years                     36705
Adolescent 12 years to < 18 years               32266
Child 2 years to < 6 years                      23946
Child 6 years to < 12 years                     22954
Adolescent (late) 18 years to < 21 years         4241
Neonate Birth to 29 days                         2355
Adult 21 years and up                            1018
Name: count, dtype: int64
```

In [13]:
```
df10 = df9.copy()
df10.Age.replace(['Adult 21 years and up','Adolescent (late) 18 years to < 21 years'],
```

In [14]:
```
df10["Age"].value_counts()
```

Out[14]:
```
Age
Infant 29 days to < 2 years          36705
Adolescent 12 years to < 18 years    32266
Child 2 years to < 6 years           23946
Child 6 years to < 12 years          22954
Adult 18 years and up                 5259
Neonate Birth to 29 days              2355
Name: count, dtype: int64
```

The cells below consolidate "Patient origin" into more discrete categories

In [15]:
```
df10["Patient Origin"].value_counts()
```

Out[15]:
```
Patient Origin
Emergency Department                                                      47563
Another Hospital's Emergency Department                                   21884
General Care Floor                                                        18033
Operating Room (Direct to ICU)                                            17792
Recovery Room (PACU)                                                       9248
Another Hospital's ICU                                                     1796
Another Hospital's General Care Floor                                      1753
Step-Down Unit/Intermediate Care Unit                                      1374
Home                                                                       1120
Physician's Office/Clinic                                                   959
Inpatient Procedure Suite (not cath lab)                                    558
Outpatient Procedure Suite                                                  255
NICU (in this hospital)                                                     229
Another Hospital's OR                                                       205
Other                                                                       151
Another ICU in this hospital (except NICU)                                  129
Transitional Care/Skilled Nursing/Chronic Care Facility                     122
Dedicated technology dependent unit (transitional/progressive care unit)    104
Physical Rehab Center                                                        64
Cath lab                                                                     57
Another hospital's cath lab                                                  17
Another hospital's Step-Down Unit/Intermediate Care Unit                     16
Psychiatric/Substance Abuse/Chemical Dependence Rehab Center                 13
Another Hospital's dedicated home ventilator unit                            13
Delivery Room (including delivery room in another hospital)                  12
Telemetry Unit                                                               10
Pulmonary Rehab Center                                                        7
Another hospital's Telemetry Unit                                             1
Name: count, dtype: int64
```

In [16]:
```
df11 = df10.copy()
df11["Patient Origin"].replace(["Another Hospital's General Care Floor", "Transitional
df11["Patient Origin"].replace(["Another Hospital's ICU", "Another Hospital's OR", "Ar
df11["Patient Origin"].replace(["Home", "Physician's Office/Clinic"], "Home/Clinic",
df11["Patient Origin"].replace(["Inpatient Procedure Suite (not cath lab)", "Outpatier
df11["Patient Origin"].replace(["Step-Down Unit/Intermediate Care Unit", "Telemetry Ur
```

In [17]:
```
df11["Patient Origin"].value_counts()
```

Out[17]:
```
Patient Origin
Emergency Department                                           47563
Another Hospital's Emergency Department                        21884
General Care Floor                                             18033
Operating Room (Direct to ICU)                                 17792
Recovery Room (PACU)                                            9248
Home/Clinic                                                     2079
Another Hospital ICU/OR/Cath                                    2018
Another Hospital Non-ED Non-ICU unit                            1989
Step down/Intermediate/Telemetry                                1488
Procedure Suite                                                  813
NICU (in this hospital)                                          229
Other                                                            151
Another ICU in this hospital (except NICU)                       129
Cath lab                                                          57
Delivery Room (including delivery room in another hospital)       12
Name: count, dtype: int64
```

Remove rows with PIM3 recovery from surgery showing a recovery from cardiac surgery

In [18]:
```
df11["PIM 3 Recovery from Surgery"].value_counts()
```

Out[18]:
```
PIM 3 Recovery from Surgery
No                                              99362
Yes, Recovery from non-cardiac procedure        23992
Yes, Recovery from non-bypass cardiac procedure    98
Yes, Recovery from bypass cardiac procedure        33
Name: count, dtype: int64
```

In [19]:
```
df12 = df11.copy()
df12 = df12.drop(df12[df12['PIM 3 Recovery from Surgery'] == 'Yes, Recovery from non-b
df12 = df12.drop(df12[df12['PIM 3 Recovery from Surgery'] == 'Yes, Recovery from bypas
df12 = df12.reset_index(drop=True)
```

In [20]:
```
df12["PIM 3 Recovery from Surgery"].value_counts()
```

Out[20]:
```
PIM 3 Recovery from Surgery
No                                          99362
Yes, Recovery from non-cardiac procedure    23992
Name: count, dtype: int64
```

Fill Race NaN with "Unspecified"

In [21]:
```
df12.Race.fillna('Unspecified', inplace=True)
df12.Race.unique()
```

Out[21]:
```
array(['White', 'Other/Mixed', 'Hispanic or Latino',
       'Black or African American', 'Asian/Indian/Pacific Islander',
       'Unspecified', 'Asian', 'American Indian or Alaska Native',
       'Native Hawaiian or Other Pacific Islander'], dtype=object)
```

Consolidate Pupillary reactions to Fixed>3mm and others/unknown

In [22]:
```
df12["PIM 3 Pupillary Reaction"].value_counts()
```

Out[22]:
```
PIM 3 Pupillary Reaction
Other                                   116524
Unknown                                   5040
>3mm and both fixed                        927
Pupilary Assessment Invalid - Drugs        651
Pupilary Assessment Invalid - Injury       141
Pupilary Assessment Invalid - Toxins        71
Name: count, dtype: int64
```

In [23]:
```
df13 = df12.copy()
df13["PIM 3 Pupillary Reaction"].replace(["Other", "Unknown", "Pupilary Assessment Inv
df13["PIM 3 Pupillary Reaction"].value_counts()
```

Out[23]:
```
PIM 3 Pupillary Reaction
Other/Unknown          122427
>3mm and both fixed       927
Name: count, dtype: int64
```

In [25]:
```
chatBot = SmartDataframe(df13, config={"llm": llm})
```

In [26]:
```
chatBot.chat("For the column 'Physical Length of Stay (days)', what percentage of rows
```
Out[26]:
```
"The percentage of rows with 'Physical Length of Stay (days)' more than 5 is: 16.43%"
```
In [29]:
```
df14 = chatBot.chat("Create a new dataframe called 'LOS' with column 'LOS', and if the
df14.info()
```
```
<class 'pandas.core.frame.DataFrame'>
RangeIndex: 123354 entries, 0 to 123353
Data columns (total 42 columns):
 #   Column                                          Non-Null Count   Dtype
---  ------                                          --------------   -----
 0   Case Index Id                                   123354 non-null  object
 1   Is Readmission                                  123354 non-null  int64
 2   Age                                             123354 non-null  object
 3   Weight (kg)                                     123354 non-null  float64
 4   Gender                                          123354 non-null  object
 5   Race                                            123354 non-null  object
 6   Patient Origin                                  123354 non-null  object
 7   Trauma                                          123354 non-null  int64
 8   Patient Type                                    123354 non-null  object
 9   Transport Team                                  98391 non-null   object
 10  Transport Vehicle                               104468 non-null  object
 11  Post Operative                                  123354 non-null  int64
 12  Baseline PCPC                                   123354 non-null  object
 13  Baseline POPC                                   123354 non-null  object
 14  Baseline FSS                                    16666 non-null   float64
 15  Cardiac Patient                                 123354 non-null  int64
 16  Cardiac Procedure directly prior to or during stay  123354 non-null  int64
 17  Medical Length of Stay (days)                   118191 non-null  float64
 18  Physical Length of Stay (days)                  123354 non-null  float64
 19  Hospital LOS                                    123115 non-null  float64
 20  Outcome                                         123354 non-null  object
 21  Disposition                                     123354 non-null  object
 22  Is Altered Code                                 122862 non-null  float64
 23  Discharge PCPC                                  118592 non-null  object
 24  Discharge POPC                                  118566 non-null  object
 25  Discharge FSS                                   16359 non-null   float64
 26  Hospital Outcome                                122609 non-null  object
 27  PIM 3 Score                                     123354 non-null  float64
 28  PIM 3 Probability of Death                      123354 non-null  float64
 29  Mechanical Ventilation (First Hour)             123354 non-null  float64
 30  Elective Admission to ICU                       123354 non-null  float64
 31  PIM 3 Recovery from Surgery                     123354 non-null  object
 32  Systolic Blood Pressure                         123354 non-null  float64
 33  PaO2 (mmHg)                                     3179 non-null    float64
 34  FiO2                                            3179 non-null    float64
 35  Base Excess                                     123354 non-null  float64
 36  PIM 3 Pupillary Reaction                        123354 non-null  object
 37  PIM 3 - No Very High Risk Dx                    123354 non-null  int64
 38  PIM 3 - No High Risk Dx                         123354 non-null  int64
 39  PIM 3 - No Low Risk Dx                          123354 non-null  int64
 40  Category                                        123354 non-null  object
 41  LOS                                             123354 non-null  int32
dtypes: float64(15), int32(1), int64(8), object(18)
memory usage: 39.1+ MB
```

Apply Ordinal Encoder to convert Object datatype to Integers

```
In [31]: toEncode = df14[['Is Readmission', 'Age', 'Gender', 'Race', 'Patient Origin', 'Trauma'
                  'Post Operative', 'Mechanical Ventilation (First Hour)', 'Elective Adm
                  'PIM 3 Recovery from Surgery', 'Category', 'Baseline PCPC', 'Baseline
                   'PIM 3 Pupillary Reaction']].copy()
         columnNames = list(toEncode.columns)

         enc = OrdinalEncoder()

         df15 = pd.DataFrame(enc.fit_transform(toEncode), columns= columnNames)

         for i in range(len(columnNames)):
             print (columnNames[i] , enc.categories_[i])
```

```
Is Readmission [0 1]
Age ['Adolescent 12 years to < 18 years' 'Adult 18 years and up'
 'Child 2 years to < 6 years' 'Child 6 years to < 12 years'
 'Infant 29 days to < 2 years' 'Neonate Birth to 29 days']
Gender ['Ambiguous' 'Female' 'Male']
Race ['American Indian or Alaska Native' 'Asian'
 'Asian/Indian/Pacific Islander' 'Black or African American'
 'Hispanic or Latino' 'Native Hawaiian or Other Pacific Islander'
 'Other/Mixed' 'Unspecified' 'White']
Patient Origin ['Another Hospital ICU/OR/Cath' 'Another Hospital Non-ED Non-ICU unit'
 "Another Hospital's Emergency Department"
 'Another ICU in this hospital (except NICU)' 'Cath lab'
 'Delivery Room (including delivery room in another hospital)'
 'Emergency Department' 'General Care Floor' 'Home/Clinic'
 'NICU (in this hospital)' 'Operating Room (Direct to ICU)' 'Other'
 'Procedure Suite' 'Recovery Room (PACU)'
 'Step down/Intermediate/Telemetry']
Trauma [0 1]
Patient Type ['Scheduled (> or = 12 Hours in Advance)' 'Unscheduled']
Post Operative [0 1]
Mechanical Ventilation (First Hour) [0. 1.]
Elective Admission to ICU [0. 1.]
PIM 3 Recovery from Surgery ['No' 'Yes, Recovery from non-cardiac procedure']
Category ['Cardiovascular' 'Dermatologic' 'Endocrine' 'Factors Influencing Health'
 'Gastrointestinal' 'Genetic' 'Gynecologic' 'Hematologic' 'Immunologic'
 'Infectious' 'Injury/Poisoning/Adverse Effects' 'Metabolic' 'Neurologic'
 'Newborn/Perinatal' 'Oncologic' 'Ophthalmologic' 'Orthopedic'
 'Psychiatric' 'Renal/Genitourinary' 'Respiratory' 'Respiratory/ENT'
 'Rheumatologic' 'Symptoms' 'Transplant' 'Ungroupable']
Baseline PCPC ['1 - Normal' '2 - Mild disability' '3 - Moderate disability'
 '4 - Severe disability' '5 - Coma or vegetative state']
Baseline POPC ['1 - Good overall performance' '2 - Mild overall disability'
 '3 - Moderate overall disability' '4 - Severe overall disability'
 '5 - Coma or vegetative state']
PIM 3 Pupillary Reaction ['>3mm and both fixed' 'Other/Unknown']
```

```
In [32]: df15.info()
```

```
<class 'pandas.core.frame.DataFrame'>
RangeIndex: 123354 entries, 0 to 123353
Data columns (total 15 columns):
 #   Column                              Non-Null Count   Dtype  
---  ------                              --------------   -----  
 0   Is Readmission                      123354 non-null  float64
 1   Age                                 123354 non-null  float64
 2   Gender                              123354 non-null  float64
 3   Race                                123354 non-null  float64
 4   Patient Origin                      123354 non-null  float64
 5   Trauma                              123354 non-null  float64
 6   Patient Type                        123354 non-null  float64
 7   Post Operative                      123354 non-null  float64
 8   Mechanical Ventilation (First Hour) 123354 non-null  float64
 9   Elective Admission to ICU           123354 non-null  float64
 10  PIM 3 Recovery from Surgery         123354 non-null  float64
 11  Category                            123354 non-null  float64
 12  Baseline PCPC                       123354 non-null  float64
 13  Baseline POPC                       123354 non-null  float64
 14  PIM 3 Pupillary Reaction            123354 non-null  float64
dtypes: float64(15)
memory usage: 14.1 MB
```

Add "weight" and "PIM3" Score into the dataframe

In [33]:

```
df16 = df15.copy()

df16.insert(2, "Weight", 0)
df16.insert(15, "PIM3", 0)
df16.insert(16, "PIM 3 Probability of Death", 0)
df16.insert(17, "Systolic Blood Pressure", 0)
df16.insert(18, "Base Excess", 0)
df16.insert(20, "PIM 3 - No Very High Risk Dx", 0)
df16.insert(21, "PIM 3 - No High Risk Dx", 0)
df16.insert(22, "PIM 3 - No Low Risk Dx", 0)




df16.Weight = df14["Weight (kg)"].copy()
df16.PIM3 = df14["PIM 3 Score"].copy()
df16["PIM 3 Probability of Death"] = df14["PIM 3 Probability of Death"].copy()
df16["Systolic Blood Pressure"] = df14["Systolic Blood Pressure"].copy()
df16["Base Excess"] = df14["Base Excess"].copy()
df16["PIM 3 - No Very High Risk Dx"] = df14["PIM 3 - No Very High Risk Dx"].copy()
df16["PIM 3 - No High Risk Dx"] = df14["PIM 3 - No High Risk Dx"].copy()
df16["PIM 3 - No Low Risk Dx"] = df14["PIM 3 - No Low Risk Dx"].copy()


df16.info()
```

```
<class 'pandas.core.frame.DataFrame'>
RangeIndex: 123354 entries, 0 to 123353
Data columns (total 23 columns):
 #   Column                               Non-Null Count   Dtype  
---  ------                               --------------   -----  
 0   Is Readmission                       123354 non-null  float64
 1   Age                                  123354 non-null  float64
 2   Weight                               123354 non-null  float64
 3   Gender                               123354 non-null  float64
 4   Race                                 123354 non-null  float64
 5   Patient Origin                       123354 non-null  float64
 6   Trauma                               123354 non-null  float64
 7   Patient Type                         123354 non-null  float64
 8   Post Operative                       123354 non-null  float64
 9   Mechanical Ventilation (First Hour)  123354 non-null  float64
 10  Elective Admission to ICU            123354 non-null  float64
 11  PIM 3 Recovery from Surgery          123354 non-null  float64
 12  Category                             123354 non-null  float64
 13  Baseline PCPC                        123354 non-null  float64
 14  Baseline POPC                        123354 non-null  float64
 15  PIM3                                 123354 non-null  float64
 16  PIM 3 Probability of Death           123354 non-null  float64
 17  Systolic Blood Pressure              123354 non-null  float64
 18  Base Excess                          123354 non-null  float64
 19  PIM 3 Pupillary Reaction             123354 non-null  float64
 20  PIM 3 - No Very High Risk Dx         123354 non-null  int64  
 21  PIM 3 - No High Risk Dx              123354 non-null  int64  
 22  PIM 3 - No Low Risk Dx               123354 non-null  int64  
dtypes: float64(20), int64(3)
memory usage: 21.6 MB
```

Add "LOS" into the dataframe and generate HEATMAP

In [34]:
```
df17 = df16.copy()

df17.insert(23, "LOS", 0)
df17.LOS = df14.LOS.copy()
```

In [35]:
```
df17.info()
```

```
<class 'pandas.core.frame.DataFrame'>
RangeIndex: 123354 entries, 0 to 123353
Data columns (total 24 columns):
 #   Column                              Non-Null Count   Dtype  
---  ------                              --------------   -----  
 0   Is Readmission                      123354 non-null  float64
 1   Age                                 123354 non-null  float64
 2   Weight                              123354 non-null  float64
 3   Gender                              123354 non-null  float64
 4   Race                                123354 non-null  float64
 5   Patient Origin                      123354 non-null  float64
 6   Trauma                              123354 non-null  float64
 7   Patient Type                        123354 non-null  float64
 8   Post Operative                      123354 non-null  float64
 9   Mechanical Ventilation (First Hour) 123354 non-null  float64
 10  Elective Admission to ICU           123354 non-null  float64
 11  PIM 3 Recovery from Surgery         123354 non-null  float64
 12  Category                            123354 non-null  float64
 13  Baseline PCPC                       123354 non-null  float64
 14  Baseline POPC                       123354 non-null  float64
 15  PIM3                                123354 non-null  float64
 16  PIM 3 Probability of Death          123354 non-null  float64
 17  Systolic Blood Pressure             123354 non-null  float64
 18  Base Excess                         123354 non-null  float64
 19  PIM 3 Pupillary Reaction            123354 non-null  float64
 20  PIM 3 - No Very High Risk Dx        123354 non-null  int64  
 21  PIM 3 - No High Risk Dx             123354 non-null  int64  
 22  PIM 3 - No Low Risk Dx              123354 non-null  int64  
 23  LOS                                 123354 non-null  int32  
dtypes: float64(20), int32(1), int64(3)
memory usage: 22.1 MB
```

In [36]:

```
def heatMap(list, fontSize):
    corr = list.corr()
    corr= corr.round(decimals=2)
    plt.figure(figsize=(20, 8))
    sns.set(font_scale = 1.2)
    sns.heatmap(corr, cmap='RdBu', vmin=-1, vmax=1, annot=True, annot_kws={'fontsize':
```

In [38]:

```
heatMap(df17, 12)
```

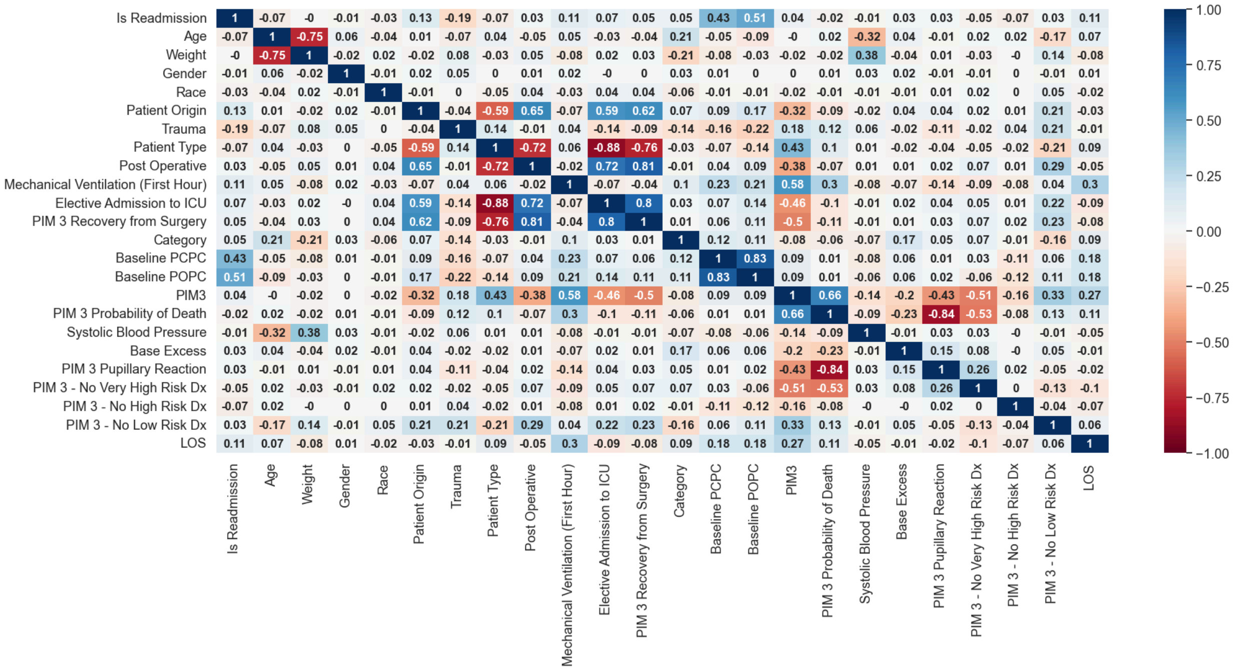

Create INPUT and OUTPUT NP arrays

In [36]:

```
input = df16.copy().to_numpy()
output = df17["LOS"].copy().to_numpy()

#input.head()
print('Input sample 25: \n' , input[27] ,  "\n \n Output sample 25: " , output[27])
```

```
Input sample 25: 
 [  0.     4.    10.8    2.     7.     1.     0.     1.     1.     1.
   0.     0.   625.     1.     2.    -1.06  25.68  89.     0.     1.
   0.     1.     1.  ]

 Output sample 25:  0
```

Apply SKLearn train/test split to Input and Output for 80:20 split

In [37]:

```
X_train, X_test, y_train, y_test = train_test_split(input, output, test_size=0.2, ranc
```

In [38]:

```
print(X_train.shape, "\t", y_train.shape, "\n")
print(X_test.shape, "\t", y_test.shape, "\n")
```

```
(98685, 23)      (98685,)

(24672, 23)      (24672,)
```

Fit MinMaxScaler on Training data, and then apply transformation to training and test set

In [39]:

```
scaler = MinMaxScaler()
scaler.fit(X_train)
```

Out[39]:

```
▾ MinMaxScaler
MinMaxScaler()
```

In [40]:

```
X_train = scaler.transform(X_train)
X_test = scaler.transform(X_test)
```

In [41]:

```
print(X_test[27], '\n' '\n', y_test[27])
```

```
[1.         0.4        0.03930435 0.5        0.75       0.92857143
 0.         0.         1.         1.         1.         1.
 0.31406045 0.75       0.75       0.17475124 0.00410287 0.42918455
 0.38844847 1.         1.         1.         1.        ]

 1
```

Import and apply LinearSVC - results appear Encouraging

Attempted standard SVC with RBF kernel too but computationally exorbitant

In [42]:

```
from sklearn.svm import LinearSVC
```

In [43]:

```
svc = LinearSVC(dual="auto", random_state=0, tol=1e-5)
svc.fit(X_train, y_train)
```

Out[43]:

```
▾ LinearSVC
LinearSVC(dual='auto', random_state=0, tol=1e-05)
```

In [44]:

```
svc_predictions = svc.predict(X_test)
```

In [45]:

```
def plotCM(test, predictions):

    print("accuracy Score is: " + str(accuracy_score(test, predictions)))

    cm = confusion_matrix(test, predictions, normalize='true')
    fig, ax = plt.subplots(figsize=(2,2))
    ConfusionMatrixDisplay(cm).plot(ax=ax, colorbar=False)
    plt.title('Sens/Spec')
    plt.show()

    cm2 = confusion_matrix(test, predictions, normalize='pred')
    fig, ax = plt.subplots(figsize=(2,2))
    ConfusionMatrixDisplay(cm2).plot(ax=ax, colorbar=False)
    plt.title('NPV/PPV')
    plt.show()

    RocCurveDisplay.from_predictions(test, predictions)
    plt.show()
```

In [46]:

```
plotCM(y_test, svc_predictions)
```

```
accuracy Score is: 0.8359273670557718
```

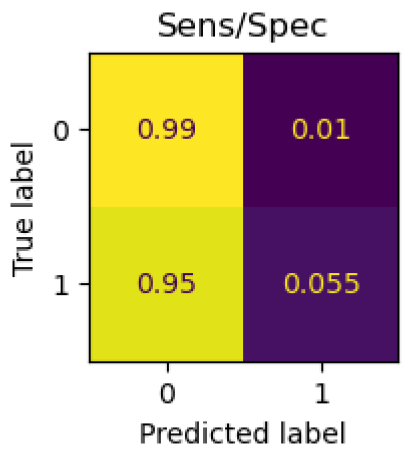

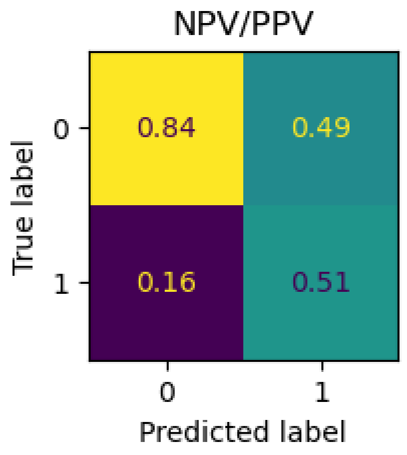

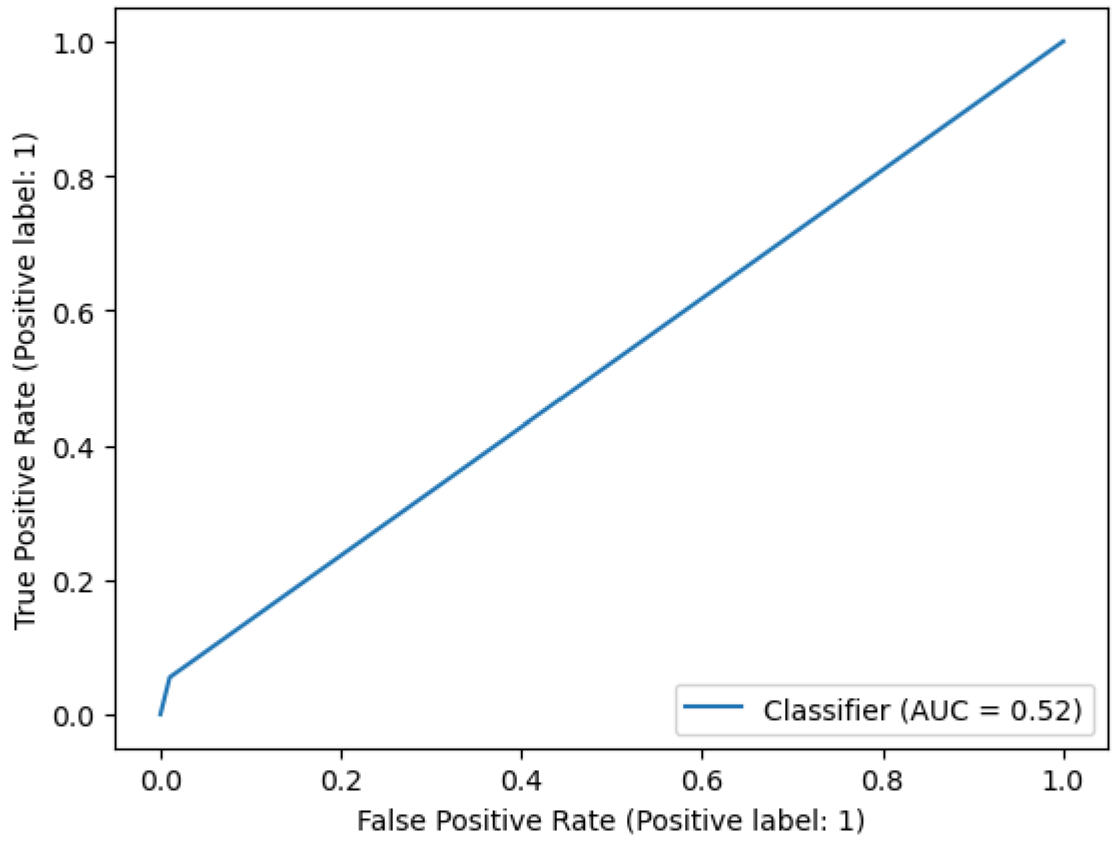

Import and apply Stochastic Gradient Descent Classification - results seen

In [47]:
```
from sklearn.linear_model import SGDClassifier
```

In [48]:
```
sgdc = SGDClassifier(loss="hinge", penalty="l2")
sgdc.fit(X_train, y_train)
```

Out[48]:
```
▾ SGDClassifier
SGDClassifier()
```

In [49]:
```
sgdc_predictions = sgdc.predict(X_test)
```

In [50]:
```
plotCM(y_test, sgdc_predictions)
```

```
accuracy Score is: 0.8354409857328146
```

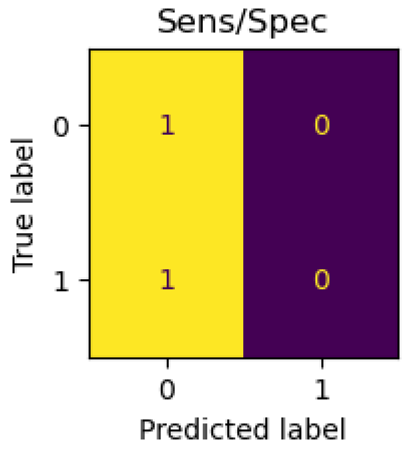

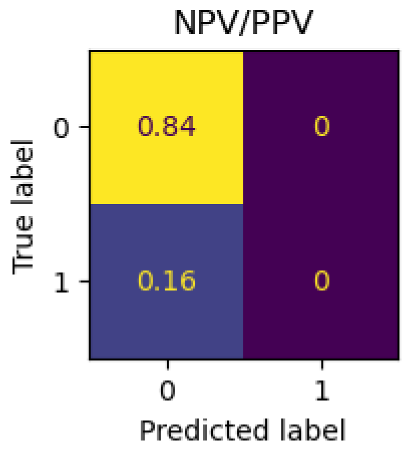

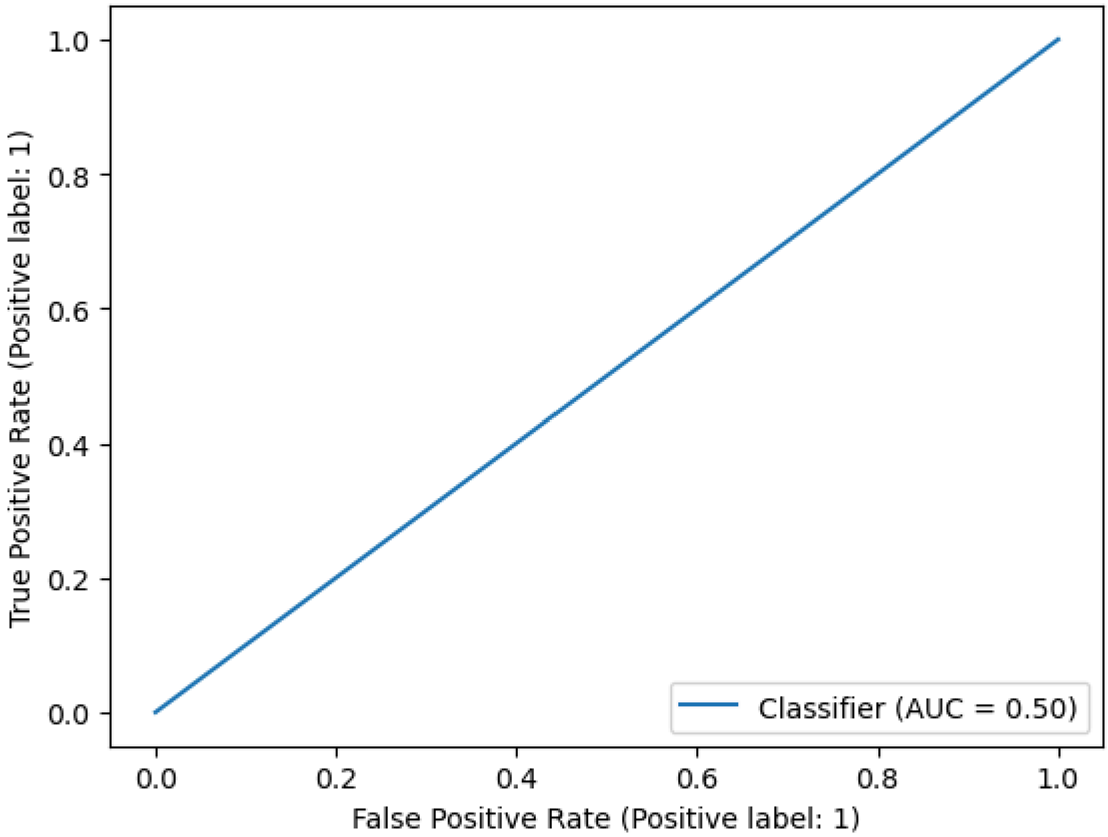

Import and apply KNN Classifier - results

In [51]:
```
from sklearn.neighbors import KNeighborsClassifier
```

In [52]:
```
knn = KNeighborsClassifier(n_neighbors=15)
knn.fit(X_train, y_train)
```

Out[52]:
```
▾ KNeighborsClassifier
KNeighborsClassifier(n_neighbors=15)
```

In [53]:
```
knn_predictions = knn.predict(X_test)
```

In [54]:
```
plotCM(y_test, knn_predictions)
```

```
accuracy Score is: 0.8344276913099871
```

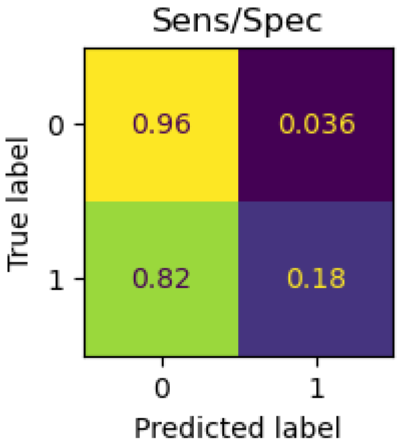

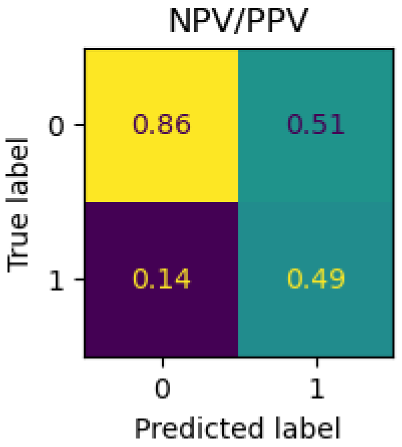

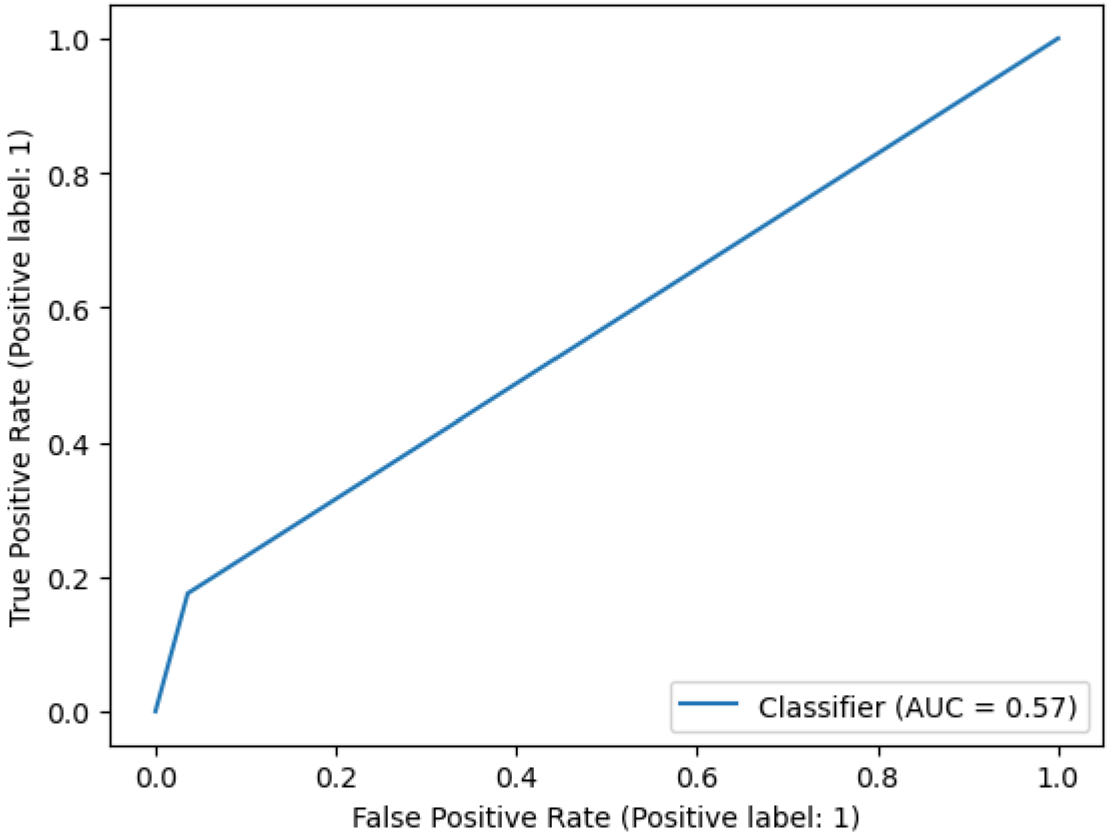

Import and apply Decision Tree Regression - results appear to be GARBAGE

In [55]:
```
from sklearn.tree import DecisionTreeClassifier
```

In [56]:
```
dtree = DecisionTreeClassifier(random_state=30)
dtree.fit(X_train, y_train)
```

Out[56]:
```
DecisionTreeClassifier
DecisionTreeClassifier(random_state=30)
```

In [57]:
```
dtree_predictions = dtree.predict(X_test)
```

In [58]:
```
plotCM(y_test, dtree_predictions)
```

```
accuracy Score is: 0.7813715953307393
```

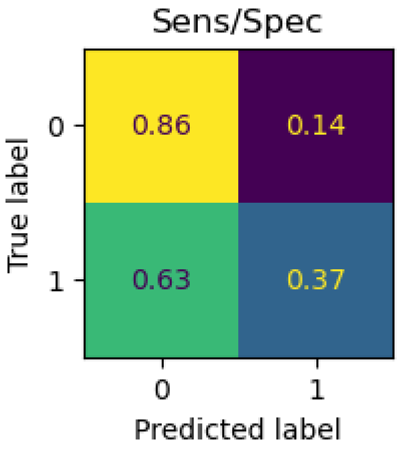

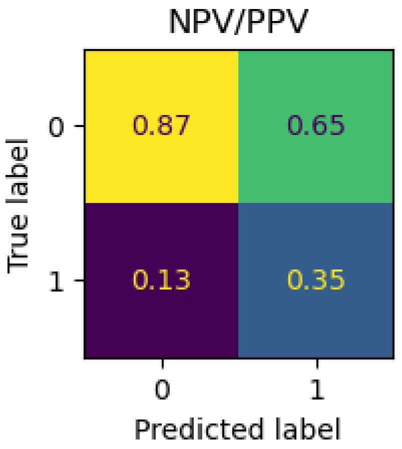

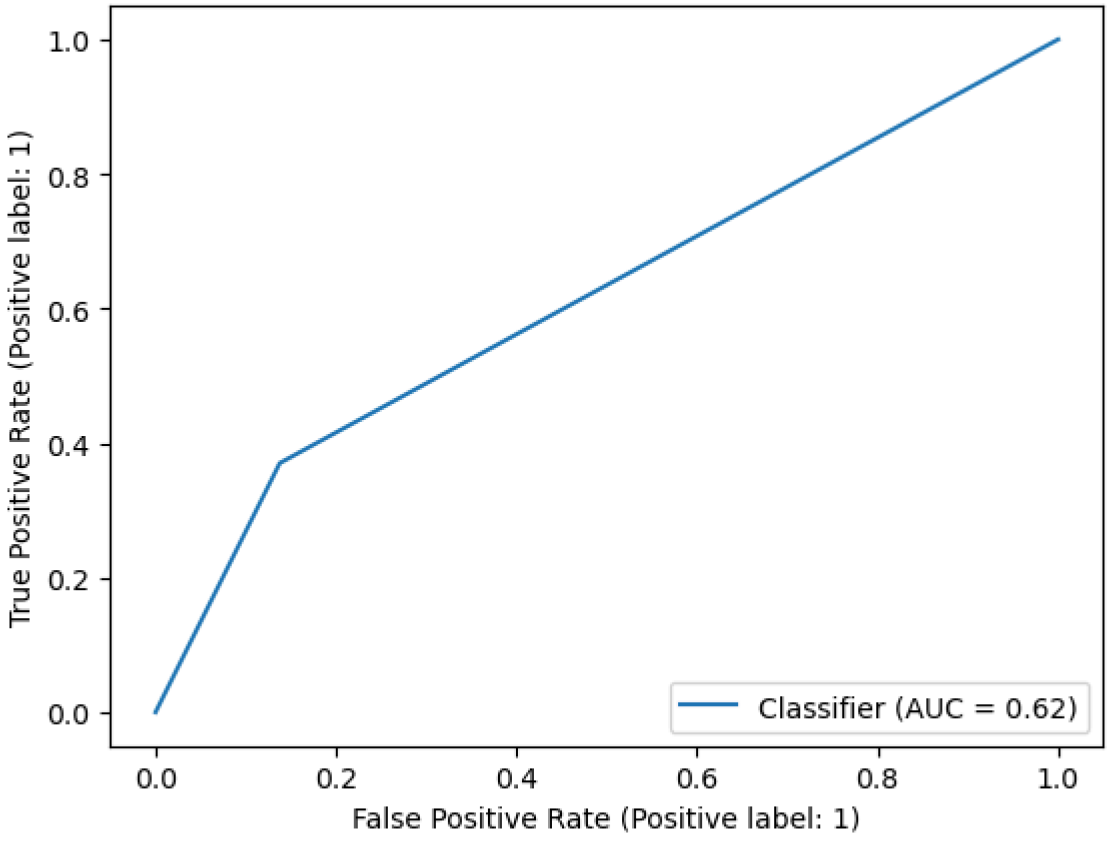

Attempt Gradient Boosting

In [59]:
```
from sklearn.ensemble import HistGradientBoostingClassifier
```

In [60]:
```
boost = HistGradientBoostingClassifier()
boost.fit(X_train, y_train)
```

Out[60]:
```
▾ HistGradientBoostingClassifier
HistGradientBoostingClassifier()
```

In [61]:
```
boost_predictions = boost.predict(X_test)
```

In [62]:
```
plotCM(y_test, boost_predictions)
```

```
accuracy Score is: 0.8465466926070039
```

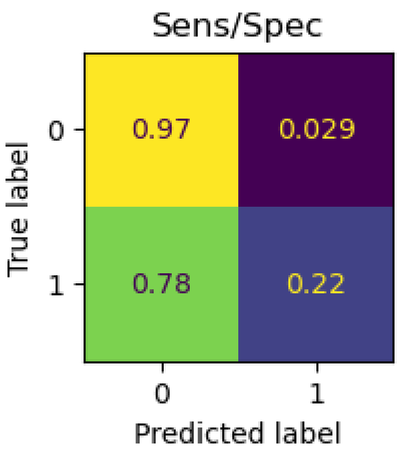

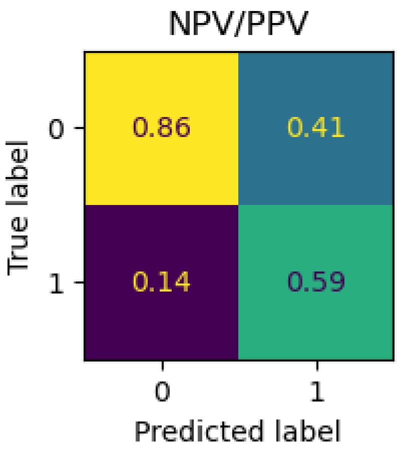

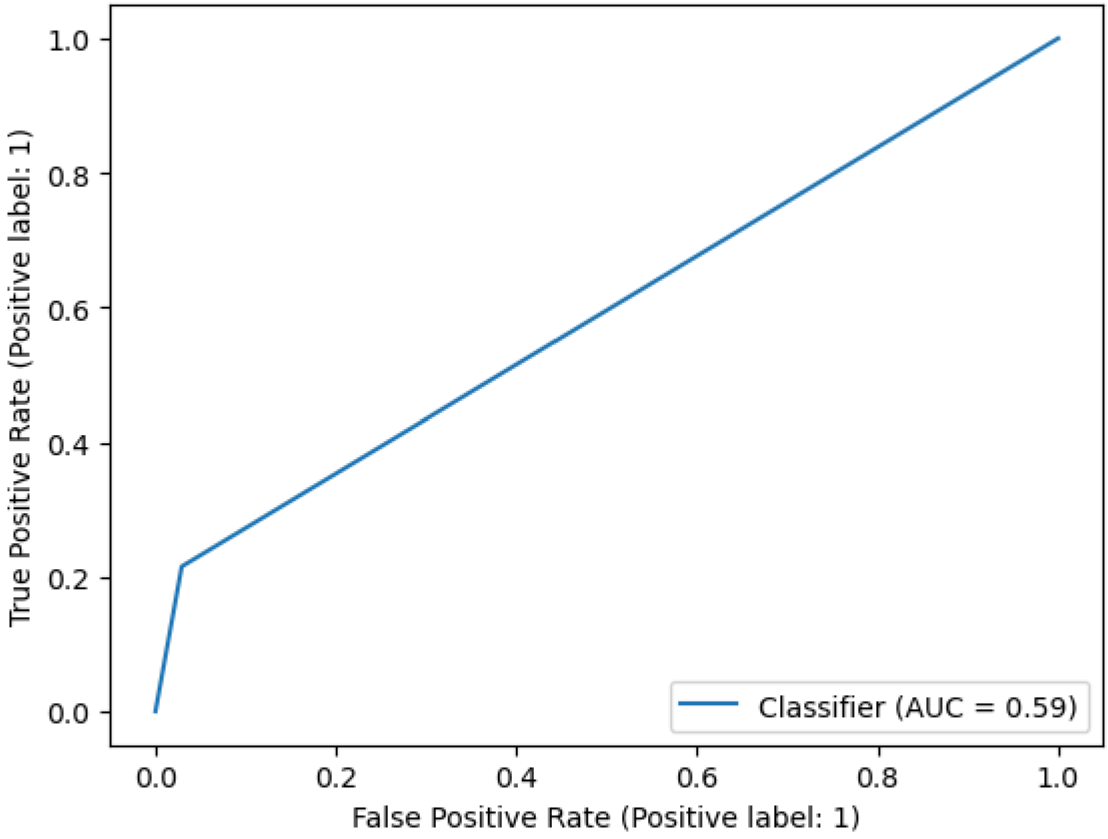

Attempt CatBoost

In [63]:
```
from catboost import CatBoostClassifier
import warnings
warnings.filterwarnings("ignore")
```

In [64]:
```
# Define the hyperparameters for the CatBoost algorithm
params = {'learning_rate': 0.1, 'depth': 6,
          'l2_leaf_reg': 3, 'iterations': 100}

# Initialize the CatBoostClassifier object
# with the defined hyperparameters and fit it on the training set
model = CatBoostClassifier(**params)
model.fit(X_train, y_train)
```

```
0:	learn: 0.6213331	total: 193ms	remaining: 19.1s
1:	learn: 0.5661020	total: 221ms	remaining: 10.8s
2:	learn: 0.5248400	total: 249ms	remaining: 8.04s
3:	learn: 0.4941306	total: 277ms	remaining: 6.64s
4:	learn: 0.4699486	total: 300ms	remaining: 5.71s
5:	learn: 0.4490647	total: 335ms	remaining: 5.24s
6:	learn: 0.4346216	total: 368ms	remaining: 4.89s
7:	learn: 0.4202236	total: 398ms	remaining: 4.58s
8:	learn: 0.4119304	total: 429ms	remaining: 4.34s
9:	learn: 0.4046446	total: 460ms	remaining: 4.14s
10:	learn: 0.3971835	total: 491ms	remaining: 3.97s
11:	learn: 0.3919723	total: 531ms	remaining: 3.89s
12:	learn: 0.3871260	total: 572ms	remaining: 3.83s
13:	learn: 0.3829057	total: 610ms	remaining: 3.75s
14:	learn: 0.3796432	total: 646ms	remaining: 3.66s
15:	learn: 0.3766262	total: 676ms	remaining: 3.55s
16:	learn: 0.3748177	total: 706ms	remaining: 3.45s
17:	learn: 0.3731045	total: 734ms	remaining: 3.34s
18:	learn: 0.3711549	total: 766ms	remaining: 3.27s
19:	learn: 0.3694947	total: 795ms	remaining: 3.18s
20:	learn: 0.3681312	total: 824ms	remaining: 3.1s
21:	learn: 0.3673827	total: 856ms	remaining: 3.04s
22:	learn: 0.3663446	total: 884ms	remaining: 2.96s
23:	learn: 0.3652260	total: 912ms	remaining: 2.89s
24:	learn: 0.3644059	total: 939ms	remaining: 2.81s
25:	learn: 0.3637218	total: 963ms	remaining: 2.74s
26:	learn: 0.3629712	total: 988ms	remaining: 2.67s
27:	learn: 0.3623214	total: 1.01s	remaining: 2.6s
28:	learn: 0.3614301	total: 1.04s	remaining: 2.54s
29:	learn: 0.3611060	total: 1.06s	remaining: 2.49s
30:	learn: 0.3607358	total: 1.1s	remaining: 2.44s
31:	learn: 0.3600547	total: 1.13s	remaining: 2.4s
32:	learn: 0.3595070	total: 1.16s	remaining: 2.35s
33:	learn: 0.3591999	total: 1.19s	remaining: 2.31s
34:	learn: 0.3586340	total: 1.22s	remaining: 2.26s
35:	learn: 0.3579563	total: 1.25s	remaining: 2.22s
36:	learn: 0.3575179	total: 1.27s	remaining: 2.17s
37:	learn: 0.3570541	total: 1.32s	remaining: 2.16s
38:	learn: 0.3568114	total: 1.37s	remaining: 2.15s
39:	learn: 0.3565871	total: 1.4s	remaining: 2.11s
40:	learn: 0.3561628	total: 1.44s	remaining: 2.07s
41:	learn: 0.3559181	total: 1.46s	remaining: 2.02s
42:	learn: 0.3555723	total: 1.51s	remaining: 2s
43:	learn: 0.3552724	total: 1.54s	remaining: 1.96s
44:	learn: 0.3548687	total: 1.57s	remaining: 1.92s
45:	learn: 0.3546232	total: 1.6s	remaining: 1.87s
46:	learn: 0.3544807	total: 1.62s	remaining: 1.83s
47:	learn: 0.3543354	total: 1.65s	remaining: 1.79s
48:	learn: 0.3540870	total: 1.68s	remaining: 1.75s
49:	learn: 0.3538538	total: 1.71s	remaining: 1.71s
50:	learn: 0.3536348	total: 1.74s	remaining: 1.67s
51:	learn: 0.3534117	total: 1.77s	remaining: 1.63s
52:	learn: 0.3530301	total: 1.8s	remaining: 1.6s
53:	learn: 0.3528174	total: 1.86s	remaining: 1.59s
54:	learn: 0.3525803	total: 1.9s	remaining: 1.55s
55:	learn: 0.3524711	total: 1.93s	remaining: 1.51s
56:	learn: 0.3522823	total: 1.96s	remaining: 1.48s
57:	learn: 0.3520195	total: 1.98s	remaining: 1.43s
58:	learn: 0.3518749	total: 2.01s	remaining: 1.4s
59:	learn: 0.3517790	total: 2.04s	remaining: 1.36s
```

```
60:	learn: 0.3516380	total: 2.08s	remaining: 1.33s
61:	learn: 0.3514807	total: 2.12s	remaining: 1.3s
62:	learn: 0.3512838	total: 2.16s	remaining: 1.27s
63:	learn: 0.3509284	total: 2.19s	remaining: 1.23s
64:	learn: 0.3506593	total: 2.22s	remaining: 1.2s
65:	learn: 0.3504531	total: 2.26s	remaining: 1.16s
66:	learn: 0.3502639	total: 2.29s	remaining: 1.13s
67:	learn: 0.3501451	total: 2.32s	remaining: 1.09s
68:	learn: 0.3499415	total: 2.36s	remaining: 1.06s
69:	learn: 0.3498178	total: 2.39s	remaining: 1.02s
70:	learn: 0.3496760	total: 2.42s	remaining: 989ms
71:	learn: 0.3495625	total: 2.45s	remaining: 953ms
72:	learn: 0.3494089	total: 2.48s	remaining: 918ms
73:	learn: 0.3493361	total: 2.51s	remaining: 881ms
74:	learn: 0.3491656	total: 2.54s	remaining: 846ms
75:	learn: 0.3489835	total: 2.57s	remaining: 811ms
76:	learn: 0.3487780	total: 2.6s	remaining: 776ms
77:	learn: 0.3486806	total: 2.62s	remaining: 739ms
78:	learn: 0.3484595	total: 2.65s	remaining: 704ms
79:	learn: 0.3481831	total: 2.68s	remaining: 670ms
80:	learn: 0.3480664	total: 2.71s	remaining: 636ms
81:	learn: 0.3479335	total: 2.74s	remaining: 601ms
82:	learn: 0.3478637	total: 2.76s	remaining: 566ms
83:	learn: 0.3477409	total: 2.79s	remaining: 531ms
84:	learn: 0.3476461	total: 2.81s	remaining: 497ms
85:	learn: 0.3475719	total: 2.84s	remaining: 462ms
86:	learn: 0.3473896	total: 2.87s	remaining: 429ms
87:	learn: 0.3472338	total: 2.9s	remaining: 396ms
88:	learn: 0.3471590	total: 2.93s	remaining: 363ms
89:	learn: 0.3470808	total: 2.96s	remaining: 329ms
90:	learn: 0.3469651	total: 2.99s	remaining: 295ms
91:	learn: 0.3468970	total: 3.02s	remaining: 262ms
92:	learn: 0.3467860	total: 3.04s	remaining: 229ms
93:	learn: 0.3466331	total: 3.07s	remaining: 196ms
94:	learn: 0.3464497	total: 3.1s	remaining: 163ms
95:	learn: 0.3463327	total: 3.13s	remaining: 130ms
96:	learn: 0.3462343	total: 3.16s	remaining: 97.8ms
97:	learn: 0.3460796	total: 3.19s	remaining: 65.1ms
98:	learn: 0.3459135	total: 3.22s	remaining: 32.6ms
99:	learn: 0.3458115	total: 3.25s	remaining: 0us
```

Out[64]:

```
<catboost.core.CatBoostClassifier at 0x1fbd9aca290>
```

In [65]:

```
y_pred = model.predict(X_test)
```

In [66]:

```
plotCM(y_test, y_pred)
```

```
accuracy Score is: 0.846587224383917
```

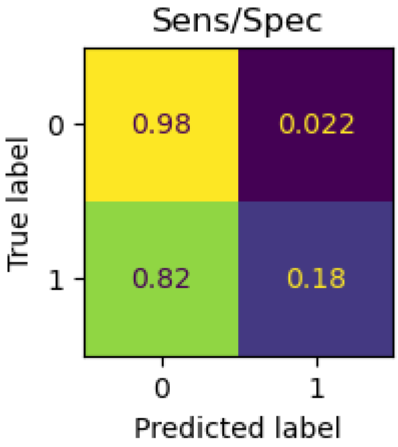

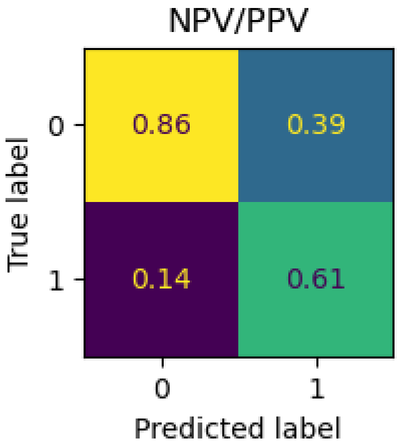

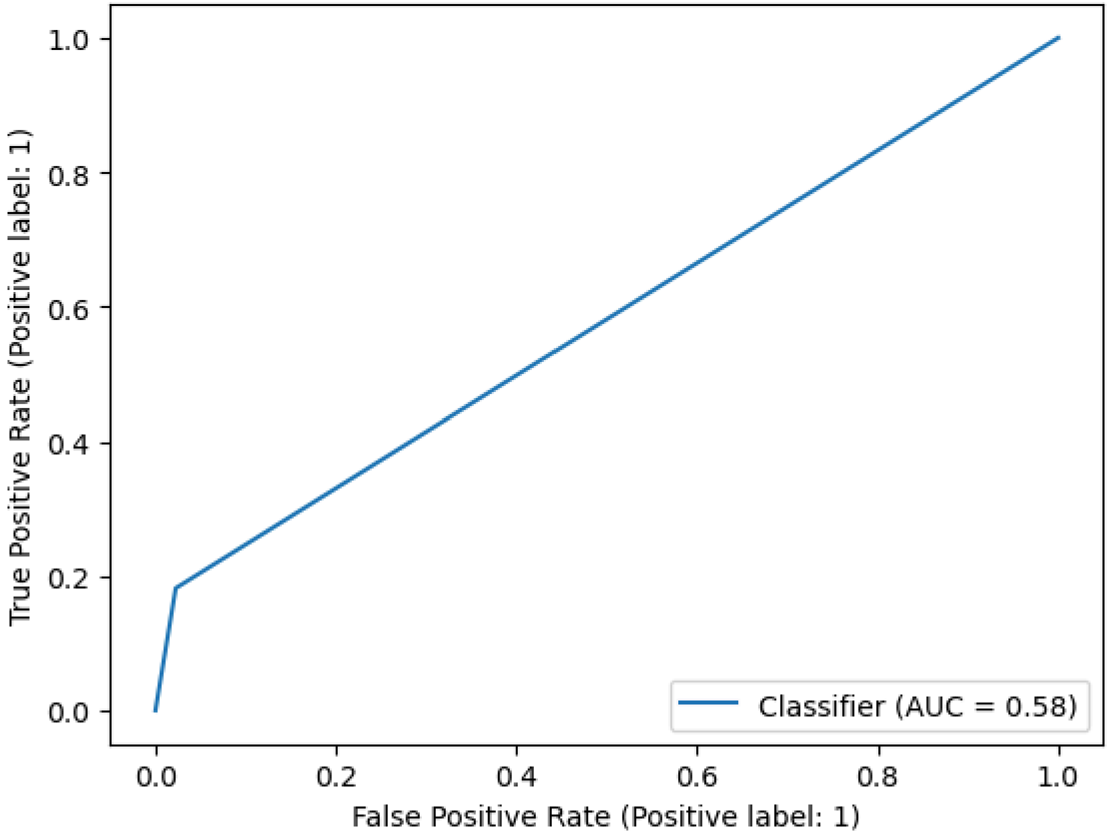

Neural Network

split Training data again in 75:25 ration to generate a total 60:20:20 split for Train:Validate:Test

In [67]:
```
X_train, X_val, y_train, y_val = train_test_split(X_train, y_train, test_size=0.25, ra
```

In [68]:
```
# Define function to plot Training Loss vs Validation Loss across epochs
def drawPlot():
    plt.plot(history.history['loss'])
    plt.plot(history.history['val_loss'])
    plt.title('model Loss')
    plt.ylabel('Loss')
    plt.xlabel('Epoch')
    #plt.xticks(np.arange(0, 21, 1.0))
    plt.legend(['train', 'val'], loc='upper left')
    plt.show()
```

In [71]:
```
drawPlot()
```

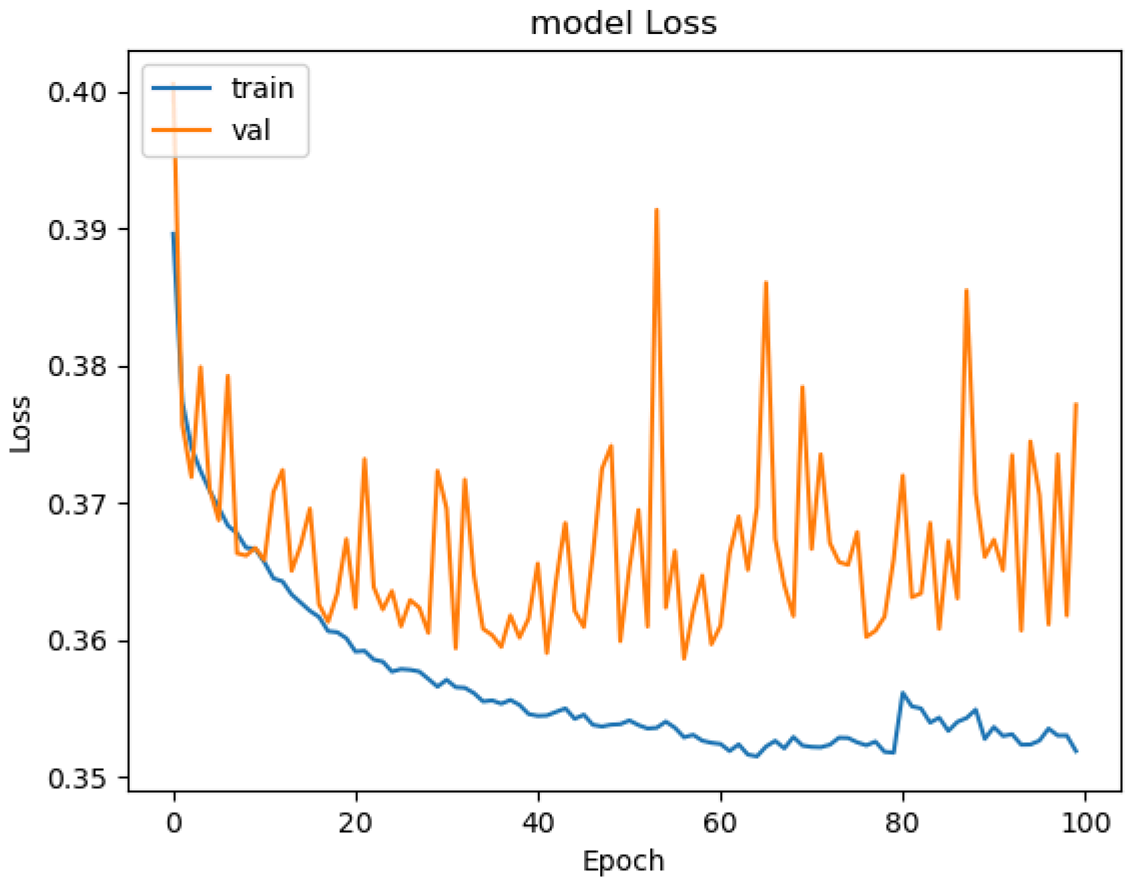

In [73]:
```
network = models.Sequential()
network.add(layers.Dense(512, activation='relu', input_shape=(23, )))
network.add(layers.Dense(256, activation='relu'))
network.add(layers.Dense(128, activation='relu'))
network.add(layers.Dense(64, activation='relu'))
network.add(layers.Dense(32, activation='relu'))
network.add(layers.Dense(16, activation='relu'))
network.add(layers.Dense(8, activation='relu'))
network.add(layers.Dense(4, activation='relu'))
network.add(layers.Dense(2, activation='relu'))
network.add(layers.Dense(1, activation='sigmoid'))
network.compile(optimizer=optimizers.RMSprop(),
```

```
                loss='binary_crossentropy',
                metrics=['accuracy'])
```

In [70]:
```
# fit model
history = network.fit(X_train, y_train,
                      batch_size=64, epochs=100,
                      validation_data = (X_val, y_val))
```

```
Epoch 1/100
1157/1157 [==============================] - 8s 5ms/step - loss: 0.3896 - accuracy:
0.8344 - val_loss: 0.4006 - val_accuracy: 0.8369
Epoch 2/100
1157/1157 [==============================] - 6s 5ms/step - loss: 0.3773 - accuracy:
0.8354 - val_loss: 0.3757 - val_accuracy: 0.8369
Epoch 3/100
1157/1157 [==============================] - 5s 4ms/step - loss: 0.3740 - accuracy:
0.8363 - val_loss: 0.3719 - val_accuracy: 0.8384
Epoch 4/100
1157/1157 [==============================] - 6s 5ms/step - loss: 0.3723 - accuracy:
0.8382 - val_loss: 0.3799 - val_accuracy: 0.8395
Epoch 5/100
1157/1157 [==============================] - 5s 5ms/step - loss: 0.3709 - accuracy:
0.8382 - val_loss: 0.3710 - val_accuracy: 0.8402
Epoch 6/100
1157/1157 [==============================] - 5s 4ms/step - loss: 0.3696 - accuracy:
0.8387 - val_loss: 0.3687 - val_accuracy: 0.8401
Epoch 7/100
1157/1157 [==============================] - 6s 5ms/step - loss: 0.3683 - accuracy:
0.8402 - val_loss: 0.3792 - val_accuracy: 0.8417
Epoch 8/100
1157/1157 [==============================] - 5s 5ms/step - loss: 0.3678 - accuracy:
0.8400 - val_loss: 0.3663 - val_accuracy: 0.8403
Epoch 9/100
1157/1157 [==============================] - 5s 5ms/step - loss: 0.3667 - accuracy:
0.8413 - val_loss: 0.3661 - val_accuracy: 0.8400
Epoch 10/100
1157/1157 [==============================] - 5s 5ms/step - loss: 0.3667 - accuracy:
0.8411 - val_loss: 0.3667 - val_accuracy: 0.8415
Epoch 11/100
1157/1157 [==============================] - 5s 5ms/step - loss: 0.3657 - accuracy:
0.8410 - val_loss: 0.3658 - val_accuracy: 0.8413
Epoch 12/100
1157/1157 [==============================] - 5s 4ms/step - loss: 0.3645 - accuracy:
0.8418 - val_loss: 0.3708 - val_accuracy: 0.8417
Epoch 13/100
1157/1157 [==============================] - 6s 5ms/step - loss: 0.3643 - accuracy:
0.8411 - val_loss: 0.3724 - val_accuracy: 0.8408
Epoch 14/100
1157/1157 [==============================] - 6s 5ms/step - loss: 0.3633 - accuracy:
0.8424 - val_loss: 0.3650 - val_accuracy: 0.8424
Epoch 15/100
1157/1157 [==============================] - 7s 6ms/step - loss: 0.3627 - accuracy:
0.8414 - val_loss: 0.3669 - val_accuracy: 0.8418
Epoch 16/100
1157/1157 [==============================] - 6s 6ms/step - loss: 0.3621 - accuracy:
0.8427 - val_loss: 0.3696 - val_accuracy: 0.8414
Epoch 17/100
1157/1157 [==============================] - 7s 6ms/step - loss: 0.3617 - accuracy:
0.8424 - val_loss: 0.3626 - val_accuracy: 0.8430
Epoch 18/100
1157/1157 [==============================] - 6s 5ms/step - loss: 0.3606 - accuracy:
0.8429 - val_loss: 0.3613 - val_accuracy: 0.8435
Epoch 19/100
1157/1157 [==============================] - 5s 4ms/step - loss: 0.3605 - accuracy:
0.8424 - val_loss: 0.3634 - val_accuracy: 0.8427
Epoch 20/100
1157/1157 [==============================] - 5s 4ms/step - loss: 0.3601 - accuracy:
0.8438 - val_loss: 0.3674 - val_accuracy: 0.8435
```

```
Epoch 21/100
1157/1157 [==============================] - 5s 4ms/step - loss: 0.3591 - accuracy:
0.8432 - val_loss: 0.3623 - val_accuracy: 0.8417
Epoch 22/100
1157/1157 [==============================] - 5s 5ms/step - loss: 0.3592 - accuracy:
0.8440 - val_loss: 0.3732 - val_accuracy: 0.8421
Epoch 23/100
1157/1157 [==============================] - 6s 5ms/step - loss: 0.3586 - accuracy:
0.8436 - val_loss: 0.3639 - val_accuracy: 0.8430
Epoch 24/100
1157/1157 [==============================] - 5s 4ms/step - loss: 0.3584 - accuracy:
0.8434 - val_loss: 0.3622 - val_accuracy: 0.8425
Epoch 25/100
1157/1157 [==============================] - 5s 4ms/step - loss: 0.3577 - accuracy:
0.8447 - val_loss: 0.3636 - val_accuracy: 0.8423
Epoch 26/100
1157/1157 [==============================] - 4s 4ms/step - loss: 0.3579 - accuracy:
0.8442 - val_loss: 0.3610 - val_accuracy: 0.8425
Epoch 27/100
1157/1157 [==============================] - 5s 5ms/step - loss: 0.3578 - accuracy:
0.8444 - val_loss: 0.3629 - val_accuracy: 0.8423
Epoch 28/100
1157/1157 [==============================] - 5s 5ms/step - loss: 0.3577 - accuracy:
0.8449 - val_loss: 0.3623 - val_accuracy: 0.8434
Epoch 29/100
1157/1157 [==============================] - 5s 4ms/step - loss: 0.3571 - accuracy:
0.8443 - val_loss: 0.3605 - val_accuracy: 0.8414
Epoch 30/100
1157/1157 [==============================] - 5s 4ms/step - loss: 0.3566 - accuracy:
0.8445 - val_loss: 0.3723 - val_accuracy: 0.8410
Epoch 31/100
1157/1157 [==============================] - 6s 5ms/step - loss: 0.3571 - accuracy:
0.8438 - val_loss: 0.3696 - val_accuracy: 0.8433
Epoch 32/100
1157/1157 [==============================] - 6s 5ms/step - loss: 0.3565 - accuracy:
0.8441 - val_loss: 0.3594 - val_accuracy: 0.8420
Epoch 33/100
1157/1157 [==============================] - 5s 5ms/step - loss: 0.3565 - accuracy:
0.8447 - val_loss: 0.3717 - val_accuracy: 0.8427
Epoch 34/100
1157/1157 [==============================] - 5s 5ms/step - loss: 0.3561 - accuracy:
0.8449 - val_loss: 0.3647 - val_accuracy: 0.8433
Epoch 35/100
1157/1157 [==============================] - 5s 4ms/step - loss: 0.3555 - accuracy:
0.8449 - val_loss: 0.3608 - val_accuracy: 0.8414
Epoch 36/100
1157/1157 [==============================] - 5s 4ms/step - loss: 0.3556 - accuracy:
0.8446 - val_loss: 0.3603 - val_accuracy: 0.8425
Epoch 37/100
1157/1157 [==============================] - 4s 4ms/step - loss: 0.3553 - accuracy:
0.8448 - val_loss: 0.3595 - val_accuracy: 0.8427
Epoch 38/100
1157/1157 [==============================] - 5s 4ms/step - loss: 0.3556 - accuracy:
0.8448 - val_loss: 0.3618 - val_accuracy: 0.8432
Epoch 39/100
1157/1157 [==============================] - 4s 4ms/step - loss: 0.3552 - accuracy:
0.8447 - val_loss: 0.3602 - val_accuracy: 0.8437
Epoch 40/100
1157/1157 [==============================] - 5s 4ms/step - loss: 0.3546 - accuracy:
0.8454 - val_loss: 0.3616 - val_accuracy: 0.8408
```

```
Epoch 41/100
1157/1157 [==============================] - 5s 5ms/step - loss: 0.3544 - accuracy:
0.8461 - val_loss: 0.3656 - val_accuracy: 0.8415
Epoch 42/100
1157/1157 [==============================] - 5s 4ms/step - loss: 0.3545 - accuracy:
0.8451 - val_loss: 0.3590 - val_accuracy: 0.8431
Epoch 43/100
1157/1157 [==============================] - 5s 4ms/step - loss: 0.3547 - accuracy:
0.8442 - val_loss: 0.3642 - val_accuracy: 0.8410
Epoch 44/100
1157/1157 [==============================] - 5s 4ms/step - loss: 0.3550 - accuracy:
0.8449 - val_loss: 0.3685 - val_accuracy: 0.8425
Epoch 45/100
1157/1157 [==============================] - 5s 4ms/step - loss: 0.3542 - accuracy:
0.8454 - val_loss: 0.3621 - val_accuracy: 0.8421
Epoch 46/100
1157/1157 [==============================] - 6s 5ms/step - loss: 0.3545 - accuracy:
0.8448 - val_loss: 0.3609 - val_accuracy: 0.8427
Epoch 47/100
1157/1157 [==============================] - 5s 5ms/step - loss: 0.3538 - accuracy:
0.8453 - val_loss: 0.3663 - val_accuracy: 0.8422
Epoch 48/100
1157/1157 [==============================] - 5s 4ms/step - loss: 0.3537 - accuracy:
0.8457 - val_loss: 0.3725 - val_accuracy: 0.8420
Epoch 49/100
1157/1157 [==============================] - 4s 4ms/step - loss: 0.3538 - accuracy:
0.8447 - val_loss: 0.3742 - val_accuracy: 0.8425
Epoch 50/100
1157/1157 [==============================] - 5s 4ms/step - loss: 0.3538 - accuracy:
0.8452 - val_loss: 0.3599 - val_accuracy: 0.8426
Epoch 51/100
1157/1157 [==============================] - 5s 4ms/step - loss: 0.3541 - accuracy:
0.8447 - val_loss: 0.3652 - val_accuracy: 0.8428
Epoch 52/100
1157/1157 [==============================] - 5s 4ms/step - loss: 0.3538 - accuracy:
0.8455 - val_loss: 0.3695 - val_accuracy: 0.8437
Epoch 53/100
1157/1157 [==============================] - 5s 5ms/step - loss: 0.3535 - accuracy:
0.8459 - val_loss: 0.3610 - val_accuracy: 0.8431
Epoch 54/100
1157/1157 [==============================] - 5s 5ms/step - loss: 0.3536 - accuracy:
0.8458 - val_loss: 0.3914 - val_accuracy: 0.8336
Epoch 55/100
1157/1157 [==============================] - 6s 5ms/step - loss: 0.3540 - accuracy:
0.8456 - val_loss: 0.3624 - val_accuracy: 0.8423
Epoch 56/100
1157/1157 [==============================] - 5s 4ms/step - loss: 0.3536 - accuracy:
0.8457 - val_loss: 0.3665 - val_accuracy: 0.8381
Epoch 57/100
1157/1157 [==============================] - 4s 4ms/step - loss: 0.3529 - accuracy:
0.8456 - val_loss: 0.3586 - val_accuracy: 0.8444
Epoch 58/100
1157/1157 [==============================] - 5s 4ms/step - loss: 0.3531 - accuracy:
0.8453 - val_loss: 0.3621 - val_accuracy: 0.8438
Epoch 59/100
1157/1157 [==============================] - 5s 4ms/step - loss: 0.3526 - accuracy:
0.8463 - val_loss: 0.3647 - val_accuracy: 0.8421
Epoch 60/100
1157/1157 [==============================] - 4s 4ms/step - loss: 0.3525 - accuracy:
0.8458 - val_loss: 0.3597 - val_accuracy: 0.8433
```

```
Epoch 61/100
1157/1157 [==============================] - 4s 4ms/step - loss: 0.3524 - accuracy:
0.8448 - val_loss: 0.3610 - val_accuracy: 0.8418
Epoch 62/100
1157/1157 [==============================] - 5s 4ms/step - loss: 0.3519 - accuracy:
0.8464 - val_loss: 0.3663 - val_accuracy: 0.8438
Epoch 63/100
1157/1157 [==============================] - 4s 4ms/step - loss: 0.3524 - accuracy:
0.8456 - val_loss: 0.3690 - val_accuracy: 0.8441
Epoch 64/100
1157/1157 [==============================] - 4s 4ms/step - loss: 0.3516 - accuracy:
0.8457 - val_loss: 0.3651 - val_accuracy: 0.8417
Epoch 65/100
1157/1157 [==============================] - 4s 4ms/step - loss: 0.3515 - accuracy:
0.8457 - val_loss: 0.3697 - val_accuracy: 0.8431
Epoch 66/100
1157/1157 [==============================] - 4s 4ms/step - loss: 0.3522 - accuracy:
0.8447 - val_loss: 0.3860 - val_accuracy: 0.8413
Epoch 67/100
1157/1157 [==============================] - 5s 4ms/step - loss: 0.3526 - accuracy:
0.8449 - val_loss: 0.3674 - val_accuracy: 0.8419
Epoch 68/100
1157/1157 [==============================] - 6s 5ms/step - loss: 0.3521 - accuracy:
0.8466 - val_loss: 0.3640 - val_accuracy: 0.8433
Epoch 69/100
1157/1157 [==============================] - 4s 4ms/step - loss: 0.3529 - accuracy:
0.8450 - val_loss: 0.3617 - val_accuracy: 0.8421
Epoch 70/100
1157/1157 [==============================] - 4s 4ms/step - loss: 0.3523 - accuracy:
0.8445 - val_loss: 0.3784 - val_accuracy: 0.8443
Epoch 71/100
1157/1157 [==============================] - 4s 3ms/step - loss: 0.3522 - accuracy:
0.8451 - val_loss: 0.3666 - val_accuracy: 0.8426
Epoch 72/100
1157/1157 [==============================] - 4s 4ms/step - loss: 0.3522 - accuracy:
0.8455 - val_loss: 0.3735 - val_accuracy: 0.8396
Epoch 73/100
1157/1157 [==============================] - 5s 4ms/step - loss: 0.3523 - accuracy:
0.8445 - val_loss: 0.3671 - val_accuracy: 0.8436
Epoch 74/100
1157/1157 [==============================] - 4s 4ms/step - loss: 0.3528 - accuracy:
0.8446 - val_loss: 0.3657 - val_accuracy: 0.8398
Epoch 75/100
1157/1157 [==============================] - 5s 5ms/step - loss: 0.3528 - accuracy:
0.8446 - val_loss: 0.3655 - val_accuracy: 0.8432
Epoch 76/100
1157/1157 [==============================] - 7s 6ms/step - loss: 0.3525 - accuracy:
0.8449 - val_loss: 0.3679 - val_accuracy: 0.8427
Epoch 77/100
1157/1157 [==============================] - 8s 6ms/step - loss: 0.3523 - accuracy:
0.8453 - val_loss: 0.3602 - val_accuracy: 0.8434
Epoch 78/100
1157/1157 [==============================] - 7s 6ms/step - loss: 0.3526 - accuracy:
0.8458 - val_loss: 0.3607 - val_accuracy: 0.8429
Epoch 79/100
1157/1157 [==============================] - 5s 4ms/step - loss: 0.3518 - accuracy:
0.8456 - val_loss: 0.3617 - val_accuracy: 0.8430
Epoch 80/100
1157/1157 [==============================] - 4s 4ms/step - loss: 0.3518 - accuracy:
0.8460 - val_loss: 0.3659 - val_accuracy: 0.8348
```

```
Epoch 81/100
1157/1157 [==============================] - 4s 4ms/step - loss: 0.3561 - accuracy:
0.8435 - val_loss: 0.3720 - val_accuracy: 0.8432
Epoch 82/100
1157/1157 [==============================] - 4s 4ms/step - loss: 0.3551 - accuracy:
0.8445 - val_loss: 0.3631 - val_accuracy: 0.8427
Epoch 83/100
1157/1157 [==============================] - 5s 4ms/step - loss: 0.3550 - accuracy:
0.8435 - val_loss: 0.3634 - val_accuracy: 0.8363
Epoch 84/100
1157/1157 [==============================] - 5s 4ms/step - loss: 0.3540 - accuracy:
0.8446 - val_loss: 0.3685 - val_accuracy: 0.8431
Epoch 85/100
1157/1157 [==============================] - 5s 4ms/step - loss: 0.3543 - accuracy:
0.8446 - val_loss: 0.3608 - val_accuracy: 0.8434
Epoch 86/100
1157/1157 [==============================] - 5s 4ms/step - loss: 0.3533 - accuracy:
0.8440 - val_loss: 0.3672 - val_accuracy: 0.8411
Epoch 87/100
1157/1157 [==============================] - 4s 4ms/step - loss: 0.3540 - accuracy:
0.8441 - val_loss: 0.3630 - val_accuracy: 0.8432
Epoch 88/100
1157/1157 [==============================] - 4s 4ms/step - loss: 0.3543 - accuracy:
0.8425 - val_loss: 0.3855 - val_accuracy: 0.8411
Epoch 89/100
1157/1157 [==============================] - 5s 4ms/step - loss: 0.3549 - accuracy:
0.8437 - val_loss: 0.3707 - val_accuracy: 0.8402
Epoch 90/100
1157/1157 [==============================] - 4s 4ms/step - loss: 0.3528 - accuracy:
0.8445 - val_loss: 0.3660 - val_accuracy: 0.8432
Epoch 91/100
1157/1157 [==============================] - 5s 4ms/step - loss: 0.3536 - accuracy:
0.8431 - val_loss: 0.3673 - val_accuracy: 0.8419
Epoch 92/100
1157/1157 [==============================] - 5s 4ms/step - loss: 0.3530 - accuracy:
0.8441 - val_loss: 0.3651 - val_accuracy: 0.8411
Epoch 93/100
1157/1157 [==============================] - 4s 4ms/step - loss: 0.3531 - accuracy:
0.8448 - val_loss: 0.3735 - val_accuracy: 0.8423
Epoch 94/100
1157/1157 [==============================] - 4s 4ms/step - loss: 0.3523 - accuracy:
0.8447 - val_loss: 0.3607 - val_accuracy: 0.8409
Epoch 95/100
1157/1157 [==============================] - 4s 4ms/step - loss: 0.3523 - accuracy:
0.8455 - val_loss: 0.3745 - val_accuracy: 0.8407
Epoch 96/100
1157/1157 [==============================] - 5s 4ms/step - loss: 0.3526 - accuracy:
0.8440 - val_loss: 0.3706 - val_accuracy: 0.8400
Epoch 97/100
1157/1157 [==============================] - 7s 6ms/step - loss: 0.3535 - accuracy:
0.8442 - val_loss: 0.3611 - val_accuracy: 0.8438
Epoch 98/100
1157/1157 [==============================] - 6s 5ms/step - loss: 0.3530 - accuracy:
0.8445 - val_loss: 0.3735 - val_accuracy: 0.8429
Epoch 99/100
1157/1157 [==============================] - 6s 5ms/step - loss: 0.3530 - accuracy:
0.8448 - val_loss: 0.3618 - val_accuracy: 0.8426
Epoch 100/100
1157/1157 [==============================] - 6s 6ms/step - loss: 0.3519 - accuracy:
0.8451 - val_loss: 0.3771 - val_accuracy: 0.8424
```

In [72]:
```
# Concatenate Train and Validation Data
X_training = np.concatenate((X_train, X_val), axis=0)
y_training = np.concatenate((y_train, y_val), axis=0)
```

In [74]:
```
# Re-train network on complete Training Data, with Epochs set at 25
network.fit(X_training, y_training,
            batch_size=64, epochs=40)
```

```
Epoch 1/40
1542/1542 [==============================] - 7s 3ms/step - loss: 0.3857 - accuracy:
0.8358
Epoch 2/40
1542/1542 [==============================] - 5s 3ms/step - loss: 0.3761 - accuracy:
0.8358
Epoch 3/40
1542/1542 [==============================] - 5s 3ms/step - loss: 0.3732 - accuracy:
0.8358
Epoch 4/40
1542/1542 [==============================] - 6s 4ms/step - loss: 0.3711 - accuracy:
0.8359
Epoch 5/40
1542/1542 [==============================] - 6s 4ms/step - loss: 0.3702 - accuracy:
0.8395
Epoch 6/40
1542/1542 [==============================] - 6s 4ms/step - loss: 0.3689 - accuracy:
0.8395
Epoch 7/40
1542/1542 [==============================] - 6s 4ms/step - loss: 0.3678 - accuracy:
0.8402
Epoch 8/40
1542/1542 [==============================] - 5s 3ms/step - loss: 0.3670 - accuracy:
0.8408
Epoch 9/40
1542/1542 [==============================] - 5s 3ms/step - loss: 0.3660 - accuracy:
0.8414
Epoch 10/40
1542/1542 [==============================] - 5s 3ms/step - loss: 0.3652 - accuracy:
0.8415
Epoch 11/40
1542/1542 [==============================] - 6s 4ms/step - loss: 0.3645 - accuracy:
0.8419
Epoch 12/40
1542/1542 [==============================] - 6s 4ms/step - loss: 0.3634 - accuracy:
0.8429
Epoch 13/40
1542/1542 [==============================] - 5s 3ms/step - loss: 0.3627 - accuracy:
0.8429
Epoch 14/40
1542/1542 [==============================] - 6s 4ms/step - loss: 0.3615 - accuracy:
0.8433
Epoch 15/40
1542/1542 [==============================] - 6s 4ms/step - loss: 0.3613 - accuracy:
0.8435
Epoch 16/40
1542/1542 [==============================] - 6s 4ms/step - loss: 0.3604 - accuracy:
0.8432
Epoch 17/40
1542/1542 [==============================] - 5s 3ms/step - loss: 0.3599 - accuracy:
0.8442
Epoch 18/40
1542/1542 [==============================] - 5s 3ms/step - loss: 0.3598 - accuracy:
0.8435
Epoch 19/40
1542/1542 [==============================] - 5s 3ms/step - loss: 0.3589 - accuracy:
0.8445
Epoch 20/40
1542/1542 [==============================] - 6s 4ms/step - loss: 0.3586 - accuracy:
0.8446
```

```
Epoch 21/40
1542/1542 [==============================] - 7s 5ms/step - loss: 0.3579 - accuracy:
0.8444
Epoch 22/40
1542/1542 [==============================] - 6s 4ms/step - loss: 0.3576 - accuracy:
0.8452
Epoch 23/40
1542/1542 [==============================] - 5s 3ms/step - loss: 0.3571 - accuracy:
0.8453
Epoch 24/40
1542/1542 [==============================] - 5s 3ms/step - loss: 0.3570 - accuracy:
0.8454
Epoch 25/40
1542/1542 [==============================] - 6s 4ms/step - loss: 0.3559 - accuracy:
0.8458
Epoch 26/40
1542/1542 [==============================] - 6s 4ms/step - loss: 0.3561 - accuracy:
0.8457
Epoch 27/40
1542/1542 [==============================] - 6s 4ms/step - loss: 0.3559 - accuracy:
0.8450
Epoch 28/40
1542/1542 [==============================] - 6s 4ms/step - loss: 0.3552 - accuracy:
0.8462
Epoch 29/40
1542/1542 [==============================] - 6s 4ms/step - loss: 0.3547 - accuracy:
0.8455
Epoch 30/40
1542/1542 [==============================] - 6s 4ms/step - loss: 0.3547 - accuracy:
0.8460
Epoch 31/40
1542/1542 [==============================] - 5s 3ms/step - loss: 0.3540 - accuracy:
0.8469
Epoch 32/40
1542/1542 [==============================] - 5s 3ms/step - loss: 0.3542 - accuracy:
0.8467
Epoch 33/40
1542/1542 [==============================] - 5s 3ms/step - loss: 0.3536 - accuracy:
0.8460
Epoch 34/40
1542/1542 [==============================] - 6s 4ms/step - loss: 0.3540 - accuracy:
0.8464
Epoch 35/40
1542/1542 [==============================] - 6s 4ms/step - loss: 0.3539 - accuracy:
0.8465
Epoch 36/40
1542/1542 [==============================] - 5s 4ms/step - loss: 0.3529 - accuracy:
0.8466
Epoch 37/40
1542/1542 [==============================] - 6s 4ms/step - loss: 0.3528 - accuracy:
0.8471
Epoch 38/40
1542/1542 [==============================] - 7s 5ms/step - loss: 0.3526 - accuracy:
0.8466
Epoch 39/40
1542/1542 [==============================] - 7s 4ms/step - loss: 0.3524 - accuracy:
0.8466
Epoch 40/40
1542/1542 [==============================] - 6s 4ms/step - loss: 0.3517 - accuracy:
0.8466
```

```
Out[74]: <keras.src.callbacks.History at 0x1fbe89e7950>
```

```
In [75]: y_prediction = network.predict(X_test)
```

```
771/771 [==============================] - 2s 2ms/step
```

```
In [76]: network.evaluate(X_test, y_test)
```

```
771/771 [==============================] - 2s 2ms/step - loss: 0.3652 - accuracy: 0.8
412
Out[76]: [0.3651596009731293, 0.8411964774131775]
```

```
In [77]: y_prediction[y_prediction >= 0.5] = 1
         y_prediction[y_prediction < 0.5] = 0
```

```
In [78]: nnDF= pd.DataFrame(y_prediction)
```

```
In [79]: nnDF.value_counts()
```

```
Out[79]: 0.0    23632
         1.0     1040
         Name: count, dtype: int64
```

```
In [80]: plotCM(y_test, y_prediction)
```

```
accuracy Score is: 0.8411964980544747
```

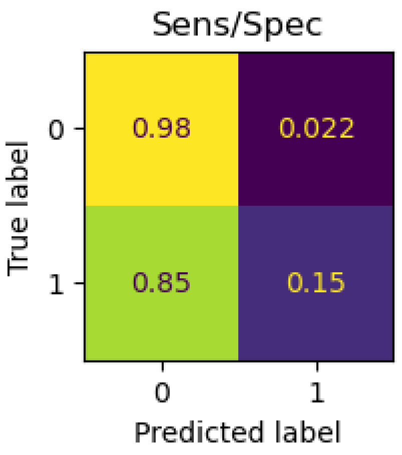

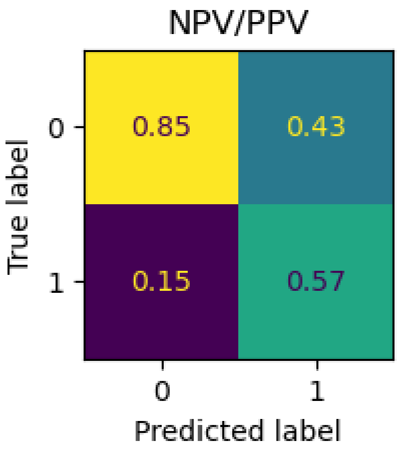

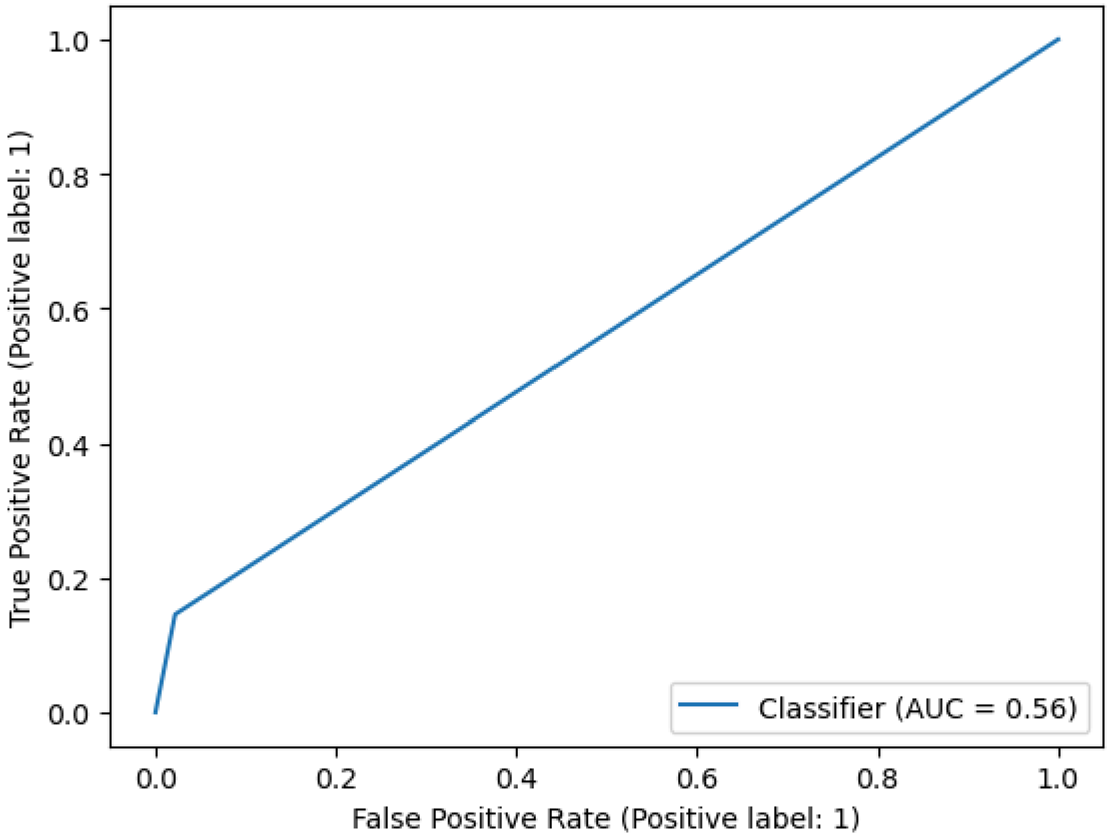
1.0
0.8
0.6
0.4
0.2
0.0
True Positive Rate (Positive label: 1)
Classifier (AUC = 0.56)
0.0
0.2
0.4
0.6
0.8
1.0
False Positive Rate (Positive label: 1)

Reimport files and re-process for 7 day threshold modeling

In [4]:
```
df5 = pd.read_csv('/Users/ganatrh/Downloads/DataWithPrimaryDiagnosis2.csv')
df70 = pd.read_csv('/Users/ganatrh/Downloads/PIM.csv')
```

In [5]:
```
df5 = df5.merge(df70, on='Case Index Id', how='left' )
```

In [6]:
```
df5.info()
```

```
<class 'pandas.core.frame.DataFrame'>
RangeIndex: 608512 entries, 0 to 608511
Data columns (total 78 columns):
 #   Column                                          Non-Null Count   Dtype  
---  ------                                          --------------   -----  
 0   Unit Id                                         608512 non-null  int64  
 1   Case Id                                         608512 non-null  int64  
 2   Case Index Id                                   608512 non-null  object 
 3   Discharge Year                                  608512 non-null  int64  
 4   Is Readmission                                  608512 non-null  int64  
 5   Age                                             608512 non-null  object 
 6   Weight (kg)                                     608512 non-null  float64
 7   Flag - Age/Weight                               608512 non-null  int64  
 8   Collects height                                 608512 non-null  int64  
 9   Height (cm)                                     309701 non-null  float64
 10  Gender                                          608512 non-null  object 
 11  Collects Race                                   608512 non-null  int64  
 12  Race                                            550714 non-null  object 
 13  Patient Origin                                  608512 non-null  object 
 14  Trauma                                          608512 non-null  int64  
 15  Patient Type                                    608512 non-null  object 
 16  Collects Transport Team                         608512 non-null  int64  
 17  Transport Team                                  371934 non-null  object 
 18  Collects Transport Vehicle                      608512 non-null  int64  
 19  Transport Vehicle                               347485 non-null  object 
 20  Post Operative                                  608512 non-null  int64  
 21  Collects Baseline PCPC/POPC                     608512 non-null  int64  
 22  Baseline PCPC                                   127543 non-null  object 
 23  Baseline POPC                                   127464 non-null  object 
 24  Collects FSS                                    608512 non-null  int64  
 25  Baseline FSS                                    59273 non-null   float64
 26  Cardiac Patient                                 608512 non-null  int64  
 27  Cardiac Procedure directly prior to or during stay  608512 non-null  int64  
 28  Medical Length of Stay (days)                   584327 non-null  float64
 29  Physical Length of Stay (days)                  608512 non-null  float64
 30  Hospital LOS                                    606176 non-null  float64
 31  Outcome                                         608512 non-null  object 
 32  Disposition                                     608512 non-null  object 
 33  Collects Limitation on Care                     608512 non-null  int64  
 34  Limitation on Care                              523209 non-null  float64
 35  Collects Brain Dead                             608512 non-null  int64  
 36  Is Brain Dead                                   4922 non-null    float64
 37  Collects Altered Code                           608512 non-null  int64  
 38  Is Altered Code                                 523227 non-null  float64
 39  Collects Withdrawal of Support                  608512 non-null  int64  
 40  Withdrawal of Support                           497357 non-null  float64
 41  Collects Autopsy                                608512 non-null  int64  
 42  Was Autopsy Performed                           11602 non-null   object 
 43  Collects Organ Donation                         608512 non-null  int64  
 44  Is Organ Donor                                  2572 non-null    float64
 45  Collects Tissue Donation                        608512 non-null  int64  
 46  Is Tissue Donor                                 3443 non-null    float64
 47  Collects Donation after Cardiac Death           608512 non-null  int64  
 48  Donation after Cardiac Death                    3563 non-null    float64
 49  Discharge PCPC                                  123029 non-null  object 
 50  Discharge POPC                                  122965 non-null  object 
 51  Discharge FSS                                   58085 non-null   float64
 52  Hospital Outcome                                604949 non-null  object 
 53  Collects Diagnosis STS Codes                    608512 non-null  int64  
 54  Collects Diagnosis ICD-9 Codes                  608512 non-null  int64  
```

```
 55  PIM 3 Score                                         608512 non-null  float64
 56  PIM 3 Probability of Death                          608512 non-null  float64
 57  Mechanical Ventilation (First Hour)                 608511 non-null  float64
 58  Elective Admission to ICU                           608511 non-null  float64
 59  PIM 3 Recovery from Surgery                         608511 non-null  object
 60  Category                                            608475 non-null  object
 61  Systolic Blood Pressure                             588367 non-null  float64
 62  Systolic Blood Pressure Unknown                     608511 non-null  float64
 63  PaO2 (mmHg)                                         13722 non-null   float64
 64  PaO2 (kPa)                                          13722 non-null   float64
 65  PaO2 Unknown                                        608511 non-null  float64
 66  FiO2                                                13722 non-null   float64
 67  FiO2 Unknown                                        608511 non-null  float64
 68  Base Excess                                         51063 non-null   float64
 69  Base Excess Unknown                                 608511 non-null  float64
 70  PIM 3 Pupillary Reaction                            608511 non-null  object
 71  PIM 3 Pupillary Reaction Unknown                    608511 non-null  float64
 72  Admitted following Cardiac Bypass - PIM2/PIM3       608511 non-null  float64
 73  Admitted following Cardiac Bypass - PIM3 Baseline   608511 non-null  float64
 74  Admitted following Cardiac Bypass - PIM3 PostIndex  166 non-null     float64
 75  PIM 3 - No Very High Risk Dx                        608512 non-null  int64
 76  PIM 3 - No High Risk Dx                             608512 non-null  int64
 77  PIM 3 - No Low Risk Dx                              608512 non-null  int64
dtypes: float64(31), int64(28), object(19)
memory usage: 362.1+ MB
```

Remove rows that have flagged inaccurate weights

In [7]:
```
df6 = SmartDataframe(df5, config={"llm": llm})
df7 = df6.chat("Remove rows that have the value '1' in column 'Flag - Age/Weight' and
df7.info()
```

```
<class 'pandas.core.frame.DataFrame'>
RangeIndex: 607901 entries, 0 to 607900
Data columns (total 78 columns):
 #   Column                                           Non-Null Count   Dtype
---  ------                                           --------------   -----
 0   Unit Id                                          607901 non-null  int64
 1   Case Id                                          607901 non-null  int64
 2   Case Index Id                                    607901 non-null  object
 3   Discharge Year                                   607901 non-null  int64
 4   Is Readmission                                   607901 non-null  int64
 5   Age                                              607901 non-null  object
 6   Weight (kg)                                      607901 non-null  float64
 7   Flag - Age/Weight                                607901 non-null  int64
 8   Collects height                                  607901 non-null  int64
 9   Height (cm)                                      309407 non-null  float64
 10  Gender                                           607901 non-null  object
 11  Collects Race                                    607901 non-null  int64
 12  Race                                             550155 non-null  object
 13  Patient Origin                                   607901 non-null  object
 14  Trauma                                           607901 non-null  int64
 15  Patient Type                                     607901 non-null  object
 16  Collects Transport Team                          607901 non-null  int64
 17  Transport Team                                   371523 non-null  object
 18  Collects Transport Vehicle                       607901 non-null  int64
 19  Transport Vehicle                                347101 non-null  object
 20  Post Operative                                   607901 non-null  int64
 21  Collects Baseline PCPC/POPC                      607901 non-null  int64
 22  Baseline PCPC                                    127387 non-null  object
 23  Baseline POPC                                    127309 non-null  object
 24  Collects FSS                                     607901 non-null  int64
 25  Baseline FSS                                     59184 non-null   float64
 26  Cardiac Patient                                  607901 non-null  int64
 27  Cardiac Procedure directly prior to or during stay  607901 non-null  int64
 28  Medical Length of Stay (days)                    583736 non-null  float64
 29  Physical Length of Stay (days)                   607901 non-null  float64
 30  Hospital LOS                                     605567 non-null  float64
 31  Outcome                                          607901 non-null  object
 32  Disposition                                      607901 non-null  object
 33  Collects Limitation on Care                      607901 non-null  int64
 34  Limitation on Care                               522675 non-null  float64
 35  Collects Brain Dead                              607901 non-null  int64
 36  Is Brain Dead                                    4918 non-null    float64
 37  Collects Altered Code                            607901 non-null  int64
 38  Is Altered Code                                  522693 non-null  float64
 39  Collects Withdrawal of Support                   607901 non-null  int64
 40  Withdrawal of Support                            496859 non-null  float64
 41  Collects Autopsy                                 607901 non-null  int64
 42  Was Autopsy Performed                            11595 non-null   object
 43  Collects Organ Donation                          607901 non-null  int64
 44  Is Organ Donor                                   2572 non-null    float64
 45  Collects Tissue Donation                         607901 non-null  int64
 46  Is Tissue Donor                                  3442 non-null    float64
 47  Collects Donation after Cardiac Death            607901 non-null  int64
 48  Donation after Cardiac Death                     3562 non-null    float64
 49  Discharge PCPC                                   122879 non-null  object
 50  Discharge POPC                                   122815 non-null  object
 51  Discharge FSS                                    58000 non-null   float64
 52  Hospital Outcome                                 604344 non-null  object
 53  Collects Diagnosis STS Codes                     607901 non-null  int64
 54  Collects Diagnosis ICD-9 Codes                   607901 non-null  int64
```

```
 55  PIM 3 Score                                           607901 non-null  float64
 56  PIM 3 Probability of Death                            607901 non-null  float64
 57  Mechanical Ventilation (First Hour)                   607900 non-null  float64
 58  Elective Admission to ICU                             607900 non-null  float64
 59  PIM 3 Recovery from Surgery                           607900 non-null  object
 60  Category                                              607864 non-null  object
 61  Systolic Blood Pressure                               587779 non-null  float64
 62  Systolic Blood Pressure Unknown                       607900 non-null  float64
 63  PaO2 (mmHg)                                           13708 non-null   float64
 64  PaO2 (kPa)                                            13708 non-null   float64
 65  PaO2 Unknown                                          607900 non-null  float64
 66  FiO2                                                  13708 non-null   float64
 67  FiO2 Unknown                                          607900 non-null  float64
 68  Base Excess                                           50991 non-null   float64
 69  Base Excess Unknown                                   607900 non-null  float64
 70  PIM 3 Pupillary Reaction                              607900 non-null  object
 71  PIM 3 Pupillary Reaction Unknown                      607900 non-null  float64
 72  Admitted following Cardiac Bypass - PIM2/PIM3         607900 non-null  float64
 73  Admitted following Cardiac Bypass - PIM3 Baseline     607900 non-null  float64
 74  Admitted following Cardiac Bypass - PIM3 PostIndex    166 non-null     float64
 75  PIM 3 - No Very High Risk Dx                          607901 non-null  int64
 76  PIM 3 - No High Risk Dx                               607901 non-null  int64
 77  PIM 3 - No Low Risk Dx                                607901 non-null  int64
dtypes: float64(31), int64(28), object(19)
memory usage: 361.8+ MB
```

Create dataframe with Columns of interest

In [8]:
```
df8 = SmartDataframe(df7, config={"llm": llm})
df9 = df8.chat("Select the columns 'Case Index Id', 'Is Readmission', 'Age', 'Weight (
```

In [9]:
```
df9.info()
```

```
<class 'pandas.core.frame.DataFrame'>
RangeIndex: 607901 entries, 0 to 607900
Data columns (total 41 columns):
 #   Column                                                Non-Null Count   Dtype  
---  ------                                                --------------   -----  
 0   Case Index Id                                         607901 non-null  object 
 1   Is Readmission                                        607901 non-null  int64  
 2   Age                                                   607901 non-null  object 
 3   Weight (kg)                                           607901 non-null  float64
 4   Gender                                                607901 non-null  object 
 5   Race                                                  550155 non-null  object 
 6   Patient Origin                                        607901 non-null  object 
 7   Trauma                                                607901 non-null  int64  
 8   Patient Type                                          607901 non-null  object 
 9   Transport Team                                        371523 non-null  object 
 10  Transport Vehicle                                     347101 non-null  object 
 11  Post Operative                                        607901 non-null  int64  
 12  Baseline PCPC                                         127387 non-null  object 
 13  Baseline POPC                                         127309 non-null  object 
 14  Baseline FSS                                          59184 non-null   float64
 15  Cardiac Patient                                       607901 non-null  int64  
 16  Cardiac Procedure directly prior to or during stay   607901 non-null  int64  
 17  Medical Length of Stay (days)                         583736 non-null  float64
 18  Physical Length of Stay (days)                        607901 non-null  float64
 19  Hospital LOS                                          605567 non-null  float64
 20  Outcome                                               607901 non-null  object 
 21  Disposition                                           607901 non-null  object 
 22  Is Altered Code                                       522693 non-null  float64
 23  Discharge PCPC                                        122879 non-null  object 
 24  Discharge POPC                                        122815 non-null  object 
 25  Discharge FSS                                         58000 non-null   float64
 26  Hospital Outcome                                      604344 non-null  object 
 27  PIM 3 Score                                           607901 non-null  float64
 28  PIM 3 Probability of Death                            607901 non-null  float64
 29  Mechanical Ventilation (First Hour)                   607900 non-null  float64
 30  Elective Admission to ICU                             607900 non-null  float64
 31  PIM 3 Recovery from Surgery                           607900 non-null  object 
 32  Systolic Blood Pressure                               587779 non-null  float64
 33  PaO2 (mmHg)                                           13708 non-null   float64
 34  FiO2                                                  13708 non-null   float64
 35  Base Excess                                           50991 non-null   float64
 36  PIM 3 Pupillary Reaction                              607900 non-null  object 
 37  PIM 3 - No Very High Risk Dx                          607901 non-null  int64  
 38  PIM 3 - No High Risk Dx                               607901 non-null  int64  
 39  PIM 3 - No Low Risk Dx                                607901 non-null  int64  
 40  Category                                              607864 non-null  object 
dtypes: float64(15), int64(8), object(18)
memory usage: 190.2+ MB
```

Remove rows that have NaN for "elective ICU admission" and for "POPC/PCPC". This comcomitantly removes NaN for other variables too

In [10]:

```python
df9.dropna(subset=['Elective Admission to ICU', 'Baseline POPC', 'Baseline PCPC', 'Sys
df9.info()
```

```
<class 'pandas.core.frame.DataFrame'>
RangeIndex: 123485 entries, 0 to 123484
Data columns (total 41 columns):
 #   Column                                                Non-Null Count   Dtype  
---  ------                                                --------------   -----  
 0   Case Index Id                                         123485 non-null  object 
 1   Is Readmission                                        123485 non-null  int64  
 2   Age                                                   123485 non-null  object 
 3   Weight (kg)                                           123485 non-null  float64
 4   Gender                                                123485 non-null  object 
 5   Race                                                  121785 non-null  object 
 6   Patient Origin                                        123485 non-null  object 
 7   Trauma                                                123485 non-null  int64  
 8   Patient Type                                          123485 non-null  object 
 9   Transport Team                                        98498 non-null   object 
 10  Transport Vehicle                                     104581 non-null  object 
 11  Post Operative                                        123485 non-null  int64  
 12  Baseline PCPC                                         123485 non-null  object 
 13  Baseline POPC                                         123485 non-null  object 
 14  Baseline FSS                                          16690 non-null   float64
 15  Cardiac Patient                                       123485 non-null  int64  
 16  Cardiac Procedure directly prior to or during stay   123485 non-null  int64  
 17  Medical Length of Stay (days)                         118321 non-null  float64
 18  Physical Length of Stay (days)                        123485 non-null  float64
 19  Hospital LOS                                          123245 non-null  float64
 20  Outcome                                               123485 non-null  object 
 21  Disposition                                           123485 non-null  object 
 22  Is Altered Code                                       122991 non-null  float64
 23  Discharge PCPC                                        118721 non-null  object 
 24  Discharge POPC                                        118695 non-null  object 
 25  Discharge FSS                                         16383 non-null   float64
 26  Hospital Outcome                                      122734 non-null  object 
 27  PIM 3 Score                                           123485 non-null  float64
 28  PIM 3 Probability of Death                            123485 non-null  float64
 29  Mechanical Ventilation (First Hour)                   123485 non-null  float64
 30  Elective Admission to ICU                             123485 non-null  float64
 31  PIM 3 Recovery from Surgery                           123485 non-null  object 
 32  Systolic Blood Pressure                               123485 non-null  float64
 33  PaO2 (mmHg)                                           3205 non-null    float64
 34  FiO2                                                  3205 non-null    float64
 35  Base Excess                                           12155 non-null   float64
 36  PIM 3 Pupillary Reaction                              123485 non-null  object 
 37  PIM 3 - No Very High Risk Dx                          123485 non-null  int64  
 38  PIM 3 - No High Risk Dx                               123485 non-null  int64  
 39  PIM 3 - No Low Risk Dx                                123485 non-null  int64  
 40  Category                                              123485 non-null  object 
dtypes: float64(15), int64(8), object(18)
memory usage: 38.6+ MB
```

In [11]:
```
df9["Base Excess"] = df9["Base Excess"].replace(np.nan, 0)
df9["Base Excess"].value_counts()
```

Out[11]:
```
Base Excess
 0.0     111514
-3.0        358
-4.0        342
-5.0        329
-2.0        327
          ...
-16.5         1
 9.7          1
 23.3         1
-34.1         1
 11.3         1
Name: count, Length: 472, dtype: int64
```

The cells below consolidate all patients > 18 into one group

In [12]:
```
df9["Age"].value_counts()
```

Out[12]:
```
Age
Infant 29 days to < 2 years                  36705
Adolescent 12 years to < 18 years            32266
Child 2 years to < 6 years                   23946
Child 6 years to < 12 years                  22954
Adolescent (late) 18 years to < 21 years      4241
Neonate Birth to 29 days                      2355
Adult 21 years and up                         1018
Name: count, dtype: int64
```

In [13]:
```
df10 = df9.copy()
df10.Age.replace(['Adult 21 years and up','Adolescent (late) 18 years to < 21 years'],
```

In [14]:
```
df10["Age"].value_counts()
```

Out[14]:
```
Age
Infant 29 days to < 2 years          36705
Adolescent 12 years to < 18 years    32266
Child 2 years to < 6 years           23946
Child 6 years to < 12 years          22954
Adult 18 years and up                 5259
Neonate Birth to 29 days              2355
Name: count, dtype: int64
```

The cells below consolidate "Patient origin" into more discrete categories

In [15]:
```
df10["Patient Origin"].value_counts()
```

Out[15]:
```
Patient Origin
Emergency Department                                                  47563
Another Hospital's Emergency Department                               21884
General Care Floor                                                    18033
Operating Room (Direct to ICU)                                        17792
Recovery Room (PACU)                                                   9248
Another Hospital's ICU                                                 1796
Another Hospital's General Care Floor                                  1753
Step-Down Unit/Intermediate Care Unit                                  1374
Home                                                                   1120
Physician's Office/Clinic                                               959
Inpatient Procedure Suite (not cath lab)                                558
Outpatient Procedure Suite                                              255
NICU (in this hospital)                                                 229
Another Hospital's OR                                                   205
Other                                                                   151
Another ICU in this hospital (except NICU)                              129
Transitional Care/Skilled Nursing/Chronic Care Facility                 122
Dedicated technology dependent unit (transitional/progressive care unit)   104
Physical Rehab Center                                                    64
Cath lab                                                                 57
Another hospital's cath lab                                              17
Another hospital's Step-Down Unit/Intermediate Care Unit                 16
Psychiatric/Substance Abuse/Chemical Dependence Rehab Center             13
Another Hospital's dedicated home ventilator unit                        13
Delivery Room (including delivery room in another hospital)              12
Telemetry Unit                                                           10
Pulmonary Rehab Center                                                    7
Another hospital's Telemetry Unit                                         1
Name: count, dtype: int64
```

In [16]:
```
df11 = df10.copy()
df11["Patient Origin"].replace(["Another Hospital's General Care Floor", "Transitional
df11["Patient Origin"].replace(["Another Hospital's ICU", "Another Hospital's OR", "Ar
df11["Patient Origin"].replace(["Home", "Physician's Office/Clinic"], "Home/Clinic",
df11["Patient Origin"].replace(["Inpatient Procedure Suite (not cath lab)", "Outpatier
df11["Patient Origin"].replace(["Step-Down Unit/Intermediate Care Unit", "Telemetry Ur
```

In [17]:
```
df11["Patient Origin"].value_counts()
```

Out[17]:
```
Patient Origin
Emergency Department                                           47563
Another Hospital's Emergency Department                        21884
General Care Floor                                             18033
Operating Room (Direct to ICU)                                 17792
Recovery Room (PACU)                                            9248
Home/Clinic                                                     2079
Another Hospital ICU/OR/Cath                                    2018
Another Hospital Non-ED Non-ICU unit                            1989
Step down/Intermediate/Telemetry                                1488
Procedure Suite                                                  813
NICU (in this hospital)                                          229
Other                                                            151
Another ICU in this hospital (except NICU)                       129
Cath lab                                                          57
Delivery Room (including delivery room in another hospital)       12
Name: count, dtype: int64
```

Remove rows with PIM3 recovery from surgery showing a recovery from cardiac surgery

In [18]:
```
df11["PIM 3 Recovery from Surgery"].value_counts()
```

Out[18]:
```
PIM 3 Recovery from Surgery
No                                              99362
Yes, Recovery from non-cardiac procedure        23992
Yes, Recovery from non-bypass cardiac procedure    98
Yes, Recovery from bypass cardiac procedure        33
Name: count, dtype: int64
```

In [19]:
```
df12 = df11.copy()
df12 = df12.drop(df12[df12['PIM 3 Recovery from Surgery'] == 'Yes, Recovery from non-b
df12 = df12.drop(df12[df12['PIM 3 Recovery from Surgery'] == 'Yes, Recovery from bypas
df12 = df12.reset_index(drop=True)
```

In [20]:
```
df12["PIM 3 Recovery from Surgery"].value_counts()
```

Out[20]:
```
PIM 3 Recovery from Surgery
No                                          99362
Yes, Recovery from non-cardiac procedure    23992
Name: count, dtype: int64
```

Fill Race NaN with "Unspecified"

In [21]:
```
df12.Race.fillna('Unspecified', inplace=True)
df12.Race.unique()
```

Out[21]:
```
array(['White', 'Other/Mixed', 'Hispanic or Latino',
       'Black or African American', 'Asian/Indian/Pacific Islander',
       'Unspecified', 'Asian', 'American Indian or Alaska Native',
       'Native Hawaiian or Other Pacific Islander'], dtype=object)
```

Consolidate Pupillary reactions to Fixed>3mm and others/unknown

In [22]:
```
df12["PIM 3 Pupillary Reaction"].value_counts()
```

Out[22]:
```
PIM 3 Pupillary Reaction
Other                                     116524
Unknown                                     5040
>3mm and both fixed                          927
Pupilary Assessment Invalid - Drugs          651
Pupilary Assessment Invalid - Injury         141
Pupilary Assessment Invalid - Toxins          71
Name: count, dtype: int64
```

In [23]:
```
df13 = df12.copy()
df13["PIM 3 Pupillary Reaction"].replace(["Other", "Unknown", "Pupilary Assessment Inv
df13["PIM 3 Pupillary Reaction"].value_counts()
```

Out[23]:
```
PIM 3 Pupillary Reaction
Other/Unknown          122427
>3mm and both fixed       927
Name: count, dtype: int64
```

In [24]:
```
chatBot = SmartDataframe(df13, config={"llm": llm})
```

```
In [25]: chatBot.chat("For the column 'Physical Length of Stay (days)', what percentage of rows

Out[25]: 11.28

In [27]: df14 = chatBot.chat("Create a new dataframe with column 'LOS', and if the value in col
         df14.LOS.value_counts()

Out[27]: LOS
         0    109439
         1     13915
         Name: count, dtype: int64

In [28]: df14.info()
```

```
<class 'pandas.core.frame.DataFrame'>
RangeIndex: 123354 entries, 0 to 123353
Data columns (total 42 columns):
 #   Column                                          Non-Null Count   Dtype  
---  ------                                          --------------   -----  
 0   Case Index Id                                   123354 non-null  object 
 1   Is Readmission                                  123354 non-null  int64  
 2   Age                                             123354 non-null  object 
 3   Weight (kg)                                     123354 non-null  float64
 4   Gender                                          123354 non-null  object 
 5   Race                                            123354 non-null  object 
 6   Patient Origin                                  123354 non-null  object 
 7   Trauma                                          123354 non-null  int64  
 8   Patient Type                                    123354 non-null  object 
 9   Transport Team                                  98391 non-null   object 
 10  Transport Vehicle                               104468 non-null  object 
 11  Post Operative                                  123354 non-null  int64  
 12  Baseline PCPC                                   123354 non-null  object 
 13  Baseline POPC                                   123354 non-null  object 
 14  Baseline FSS                                    16666 non-null   float64
 15  Cardiac Patient                                 123354 non-null  int64  
 16  Cardiac Procedure directly prior to or during stay 123354 non-null  int64  
 17  Medical Length of Stay (days)                   118191 non-null  float64
 18  Physical Length of Stay (days)                  123354 non-null  float64
 19  Hospital LOS                                    123115 non-null  float64
 20  Outcome                                         123354 non-null  object 
 21  Disposition                                     123354 non-null  object 
 22  Is Altered Code                                 122862 non-null  float64
 23  Discharge PCPC                                  118592 non-null  object 
 24  Discharge POPC                                  118566 non-null  object 
 25  Discharge FSS                                   16359 non-null   float64
 26  Hospital Outcome                                122609 non-null  object 
 27  PIM 3 Score                                     123354 non-null  float64
 28  PIM 3 Probability of Death                      123354 non-null  float64
 29  Mechanical Ventilation (First Hour)             123354 non-null  float64
 30  Elective Admission to ICU                       123354 non-null  float64
 31  PIM 3 Recovery from Surgery                     123354 non-null  object 
 32  Systolic Blood Pressure                         123354 non-null  float64
 33  PaO2 (mmHg)                                     3179 non-null    float64
 34  FiO2                                            3179 non-null    float64
 35  Base Excess                                     123354 non-null  float64
 36  PIM 3 Pupillary Reaction                        123354 non-null  object 
 37  PIM 3 - No Very High Risk Dx                    123354 non-null  int64  
 38  PIM 3 - No High Risk Dx                         123354 non-null  int64  
 39  PIM 3 - No Low Risk Dx                          123354 non-null  int64  
 40  Category                                        123354 non-null  object 
 41  LOS                                             123354 non-null  int64  
dtypes: float64(15), int64(9), object(18)
memory usage: 39.5+ MB
```

Apply Ordinal Encoder to convert Object datatype to Integers

In [29]:

```
toEncode = df14[['Is Readmission', 'Age', 'Gender', 'Race', 'Patient Origin', 'Trauma'
                'Post Operative', 'Mechanical Ventilation (First Hour)', 'Elective Adm
                'PIM 3 Recovery from Surgery', 'Category', 'Baseline PCPC', 'Baseline 
                 'PIM 3 Pupillary Reaction']].copy()
columnNames = list(toEncode.columns)

enc = OrdinalEncoder()
```

```
df15 = pd.DataFrame(enc.fit_transform(toEncode), columns= columnNames)

for i in range(len(columnNames)):
    print (columnNames[i] , enc.categories_[i])
```

```
Is Readmission [0 1]
Age ['Adolescent 12 years to < 18 years' 'Adult 18 years and up'
 'Child 2 years to < 6 years' 'Child 6 years to < 12 years'
 'Infant 29 days to < 2 years' 'Neonate Birth to 29 days']
Gender ['Ambiguous' 'Female' 'Male']
Race ['American Indian or Alaska Native' 'Asian'
 'Asian/Indian/Pacific Islander' 'Black or African American'
 'Hispanic or Latino' 'Native Hawaiian or Other Pacific Islander'
 'Other/Mixed' 'Unspecified' 'White']
Patient Origin ['Another Hospital ICU/OR/Cath' 'Another Hospital Non-ED Non-ICU unit'
 "Another Hospital's Emergency Department"
 'Another ICU in this hospital (except NICU)' 'Cath lab'
 'Delivery Room (including delivery room in another hospital)'
 'Emergency Department' 'General Care Floor' 'Home/Clinic'
 'NICU (in this hospital)' 'Operating Room (Direct to ICU)' 'Other'
 'Procedure Suite' 'Recovery Room (PACU)'
 'Step down/Intermediate/Telemetry']
Trauma [0 1]
Patient Type ['Scheduled (> or = 12 Hours in Advance)' 'Unscheduled']
Post Operative [0 1]
Mechanical Ventilation (First Hour) [0. 1.]
Elective Admission to ICU [0. 1.]
PIM 3 Recovery from Surgery ['No' 'Yes, Recovery from non-cardiac procedure']
Category ['Cardiovascular' 'Dermatologic' 'Endocrine' 'Factors Influencing Health'
 'Gastrointestinal' 'Genetic' 'Gynecologic' 'Hematologic' 'Immunologic'
 'Infectious' 'Injury/Poisoning/Adverse Effects' 'Metabolic' 'Neurologic'
 'Newborn/Perinatal' 'Oncologic' 'Ophthalmologic' 'Orthopedic'
 'Psychiatric' 'Renal/Genitourinary' 'Respiratory' 'Respiratory/ENT'
 'Rheumatologic' 'Symptoms' 'Transplant' 'Ungroupable']
Baseline PCPC ['1 - Normal' '2 - Mild disability' '3 - Moderate disability'
 '4 - Severe disability' '5 - Coma or vegetative state']
Baseline POPC ['1 - Good overall performance' '2 - Mild overall disability'
 '3 - Moderate overall disability' '4 - Severe overall disability'
 '5 - Coma or vegetative state']
PIM 3 Pupillary Reaction ['>3mm and both fixed' 'Other/Unknown']
```

In [30]:
```
df15.info()
```

```
<class 'pandas.core.frame.DataFrame'>
RangeIndex: 123354 entries, 0 to 123353
Data columns (total 15 columns):
 #   Column                              Non-Null Count   Dtype  
---  ------                              --------------   -----  
 0   Is Readmission                      123354 non-null  float64
 1   Age                                 123354 non-null  float64
 2   Gender                              123354 non-null  float64
 3   Race                                123354 non-null  float64
 4   Patient Origin                      123354 non-null  float64
 5   Trauma                              123354 non-null  float64
 6   Patient Type                        123354 non-null  float64
 7   Post Operative                      123354 non-null  float64
 8   Mechanical Ventilation (First Hour) 123354 non-null  float64
 9   Elective Admission to ICU           123354 non-null  float64
 10  PIM 3 Recovery from Surgery         123354 non-null  float64
 11  Category                            123354 non-null  float64
 12  Baseline PCPC                       123354 non-null  float64
 13  Baseline POPC                       123354 non-null  float64
 14  PIM 3 Pupillary Reaction            123354 non-null  float64
dtypes: float64(15)
memory usage: 14.1 MB
```

Add "weight" and "PIM3" Score into the dataframe

In [31]:

```
df16 = df15.copy()

df16.insert(2, "Weight", 0)
df16.insert(15, "PIM3", 0)
df16.insert(16, "PIM 3 Probability of Death", 0)
df16.insert(17, "Systolic Blood Pressure", 0)
df16.insert(18, "Base Excess", 0)
df16.insert(20, "PIM 3 - No Very High Risk Dx", 0)
df16.insert(21, "PIM 3 - No High Risk Dx", 0)
df16.insert(22, "PIM 3 - No Low Risk Dx", 0)




df16.Weight = df14["Weight (kg)"].copy()
df16.PIM3 = df14["PIM 3 Score"].copy()
df16["PIM 3 Probability of Death"] = df14["PIM 3 Probability of Death"].copy()
df16["Systolic Blood Pressure"] = df14["Systolic Blood Pressure"].copy()
df16["Base Excess"] = df14["Base Excess"].copy()
df16["PIM 3 - No Very High Risk Dx"] = df14["PIM 3 - No Very High Risk Dx"].copy()
df16["PIM 3 - No High Risk Dx"] = df14["PIM 3 - No High Risk Dx"].copy()
df16["PIM 3 - No Low Risk Dx"] = df14["PIM 3 - No Low Risk Dx"].copy()


df16.info()
```

```
<class 'pandas.core.frame.DataFrame'>
RangeIndex: 123354 entries, 0 to 123353
Data columns (total 23 columns):
 #   Column                               Non-Null Count   Dtype
---  ------                               --------------   -----
 0   Is Readmission                       123354 non-null  float64
 1   Age                                  123354 non-null  float64
 2   Weight                               123354 non-null  float64
 3   Gender                               123354 non-null  float64
 4   Race                                 123354 non-null  float64
 5   Patient Origin                       123354 non-null  float64
 6   Trauma                               123354 non-null  float64
 7   Patient Type                         123354 non-null  float64
 8   Post Operative                       123354 non-null  float64
 9   Mechanical Ventilation (First Hour)  123354 non-null  float64
 10  Elective Admission to ICU            123354 non-null  float64
 11  PIM 3 Recovery from Surgery          123354 non-null  float64
 12  Category                             123354 non-null  float64
 13  Baseline PCPC                        123354 non-null  float64
 14  Baseline POPC                        123354 non-null  float64
 15  PIM3                                 123354 non-null  float64
 16  PIM 3 Probability of Death           123354 non-null  float64
 17  Systolic Blood Pressure              123354 non-null  float64
 18  Base Excess                          123354 non-null  float64
 19  PIM 3 Pupillary Reaction             123354 non-null  float64
 20  PIM 3 - No Very High Risk Dx         123354 non-null  int64
 21  PIM 3 - No High Risk Dx              123354 non-null  int64
 22  PIM 3 - No Low Risk Dx               123354 non-null  int64
dtypes: float64(20), int64(3)
memory usage: 21.6 MB
```

Add "LOS" into the dataframe and generate HEATMAP

In [32]:
```
df17 = df16.copy()

df17.insert(23, "LOS", 0)
df17.LOS = df14.LOS.copy()
```

In [33]:
```
df17.info()
```

```
<class 'pandas.core.frame.DataFrame'>
RangeIndex: 123354 entries, 0 to 123353
Data columns (total 24 columns):
 #   Column                              Non-Null Count   Dtype  
---  ------                              --------------   -----  
 0   Is Readmission                      123354 non-null  float64
 1   Age                                 123354 non-null  float64
 2   Weight                              123354 non-null  float64
 3   Gender                              123354 non-null  float64
 4   Race                                123354 non-null  float64
 5   Patient Origin                      123354 non-null  float64
 6   Trauma                              123354 non-null  float64
 7   Patient Type                        123354 non-null  float64
 8   Post Operative                      123354 non-null  float64
 9   Mechanical Ventilation (First Hour) 123354 non-null  float64
 10  Elective Admission to ICU           123354 non-null  float64
 11  PIM 3 Recovery from Surgery         123354 non-null  float64
 12  Category                            123354 non-null  float64
 13  Baseline PCPC                       123354 non-null  float64
 14  Baseline POPC                       123354 non-null  float64
 15  PIM3                                123354 non-null  float64
 16  PIM 3 Probability of Death          123354 non-null  float64
 17  Systolic Blood Pressure             123354 non-null  float64
 18  Base Excess                         123354 non-null  float64
 19  PIM 3 Pupillary Reaction            123354 non-null  float64
 20  PIM 3 - No Very High Risk Dx        123354 non-null  int64  
 21  PIM 3 - No High Risk Dx             123354 non-null  int64  
 22  PIM 3 - No Low Risk Dx              123354 non-null  int64  
 23  LOS                                 123354 non-null  int64  
dtypes: float64(20), int64(4)
memory usage: 22.6 MB
```

In [34]:

```
def heatMap(list, fontSize):
    corr = list.corr()
    corr= corr.round(decimals=2)
    plt.figure(figsize=(20, 8))
    sns.set(font_scale = 1.2)
    sns.heatmap(corr, cmap='RdBu', vmin=-1, vmax=1, annot=True, annot_kws={'fontsize':
```

In [35]:

```
heatMap(df17, 12)
```

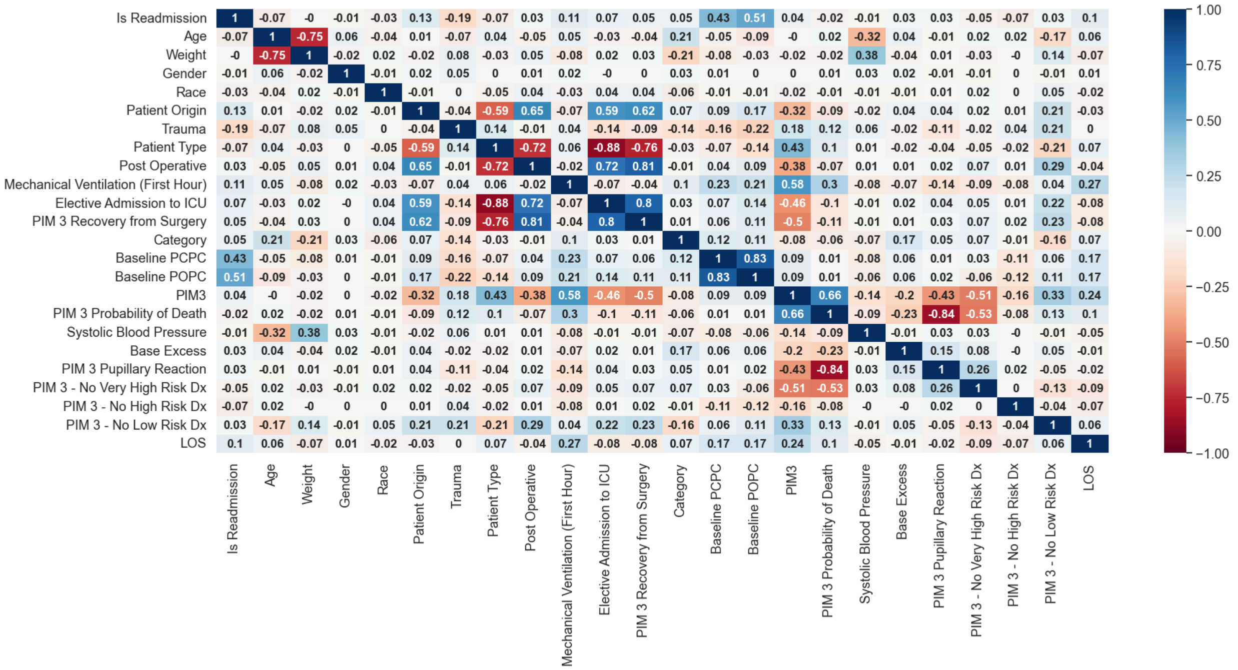

Create INPUT and OUTPUT NP arrays

In [36]:

```
input = df16.copy().to_numpy()
output = df17["LOS"].copy().to_numpy()

#input.head()
print('Input sample 25: \n' , input[27] ,  "\n \n Output sample 25: " , output[27])
```

```
Input sample 25: 
 [  0.      4.     10.8     2.      7.      1.      0.      1.      1.      1.
   0.      0.    625.      1.      2.     -1.06   25.68   89.      0.      1.
   0.      1.      1.   ]

 Output sample 25:  0
```

Apply SKLearn train/test split to Input and Output for 80:20 split

In [37]:

```
X_train, X_test, y_train, y_test = train_test_split(input, output, test_size=0.2, ranc
```

In [38]:

```
print(X_train.shape, "\t", y_train.shape, "\n")
print(X_test.shape, "\t", y_test.shape, "\n")
```

```
(98685, 23)      (98685,)

(24672, 23)      (24672,)
```

Fit MinMaxScaler on Training data, and then apply transformation to training and test set

In [39]:

```
scaler = MinMaxScaler()
scaler.fit(X_train)
```

Out[39]:

```
▾ MinMaxScaler
MinMaxScaler()
```

In [40]:

```
X_train = scaler.transform(X_train)
X_test = scaler.transform(X_test)
```

In [41]:

```
print(X_test[27], '\n' '\n', y_test[27])
```

```
[1.         0.4        0.03930435 0.5        0.75       0.92857143
 0.         0.         1.         1.         1.         1.
 0.31406045 0.75       0.75       0.17475124 0.00410287 0.42918455
 0.38844847 1.         1.         1.         1.        ]

 1
```

Import and apply LinearSVC - results appear Encouraging

Attempted standard SVC with RBF kernel too but computationally exorbitant

In [42]:

```
from sklearn.svm import LinearSVC
```

In [43]:

```
svc = LinearSVC(dual="auto", random_state=0, tol=1e-5)
svc.fit(X_train, y_train)
```

Out[43]:

```
▾ LinearSVC
LinearSVC(dual='auto', random_state=0, tol=1e-05)
```

In [44]:

```
svc_predictions = svc.predict(X_test)
```

In [45]:

```
def plotCM(test, predictions):

    print("accuracy Score is: " + str(accuracy_score(test, predictions)))

    cm = confusion_matrix(test, predictions, normalize='true')
    fig, ax = plt.subplots(figsize=(2,2))
    ConfusionMatrixDisplay(cm).plot(ax=ax, colorbar=False)
    plt.title('Sens/Spec')
    plt.show()

    cm2 = confusion_matrix(test, predictions, normalize='pred')
    fig, ax = plt.subplots(figsize=(2,2))
    ConfusionMatrixDisplay(cm2).plot(ax=ax, colorbar=False)
    plt.title('NPV/PPV')
    plt.show()

    RocCurveDisplay.from_predictions(test, predictions)
    plt.show()
```

In [46]:

```
plotCM(y_test, svc_predictions)
```

```
accuracy Score is: 0.8853761348897535
```

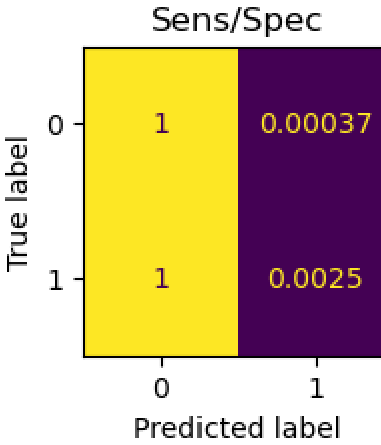

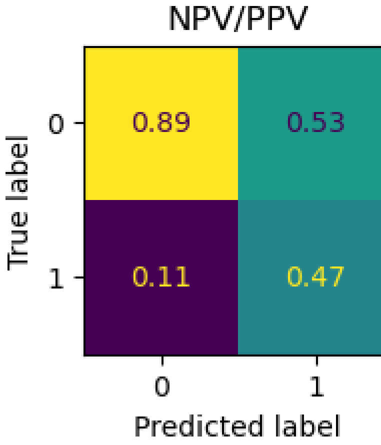

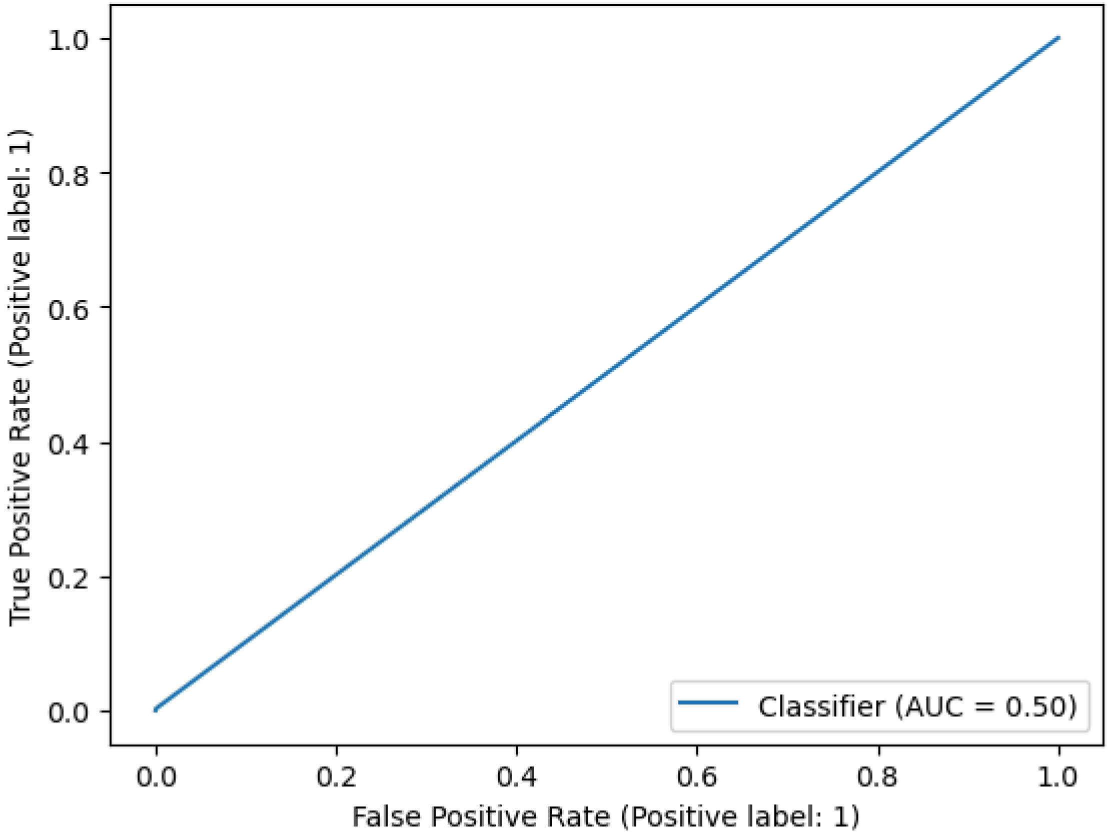

Import and apply Stochastic Gradient Descent Classification - results seen

In [47]:
```python
from sklearn.linear_model import SGDClassifier
```

In [48]:
```python
sgdc = SGDClassifier(loss="hinge", penalty="l2")
sgdc.fit(X_train, y_train)
```

Out[48]:
```
▾ SGDClassifier
SGDClassifier()
```

In [49]:
```python
sgdc_predictions = sgdc.predict(X_test)
```

In [50]:
```python
plotCM(y_test, sgdc_predictions)
```

```
accuracy Score is: 0.8854166666666666
```

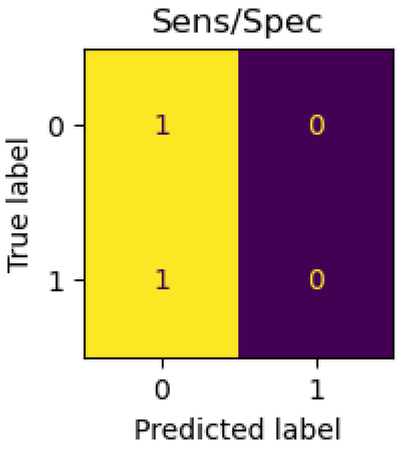

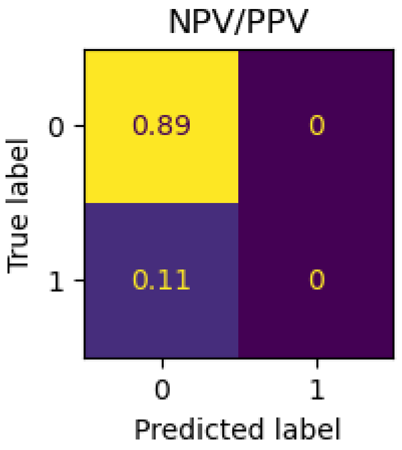

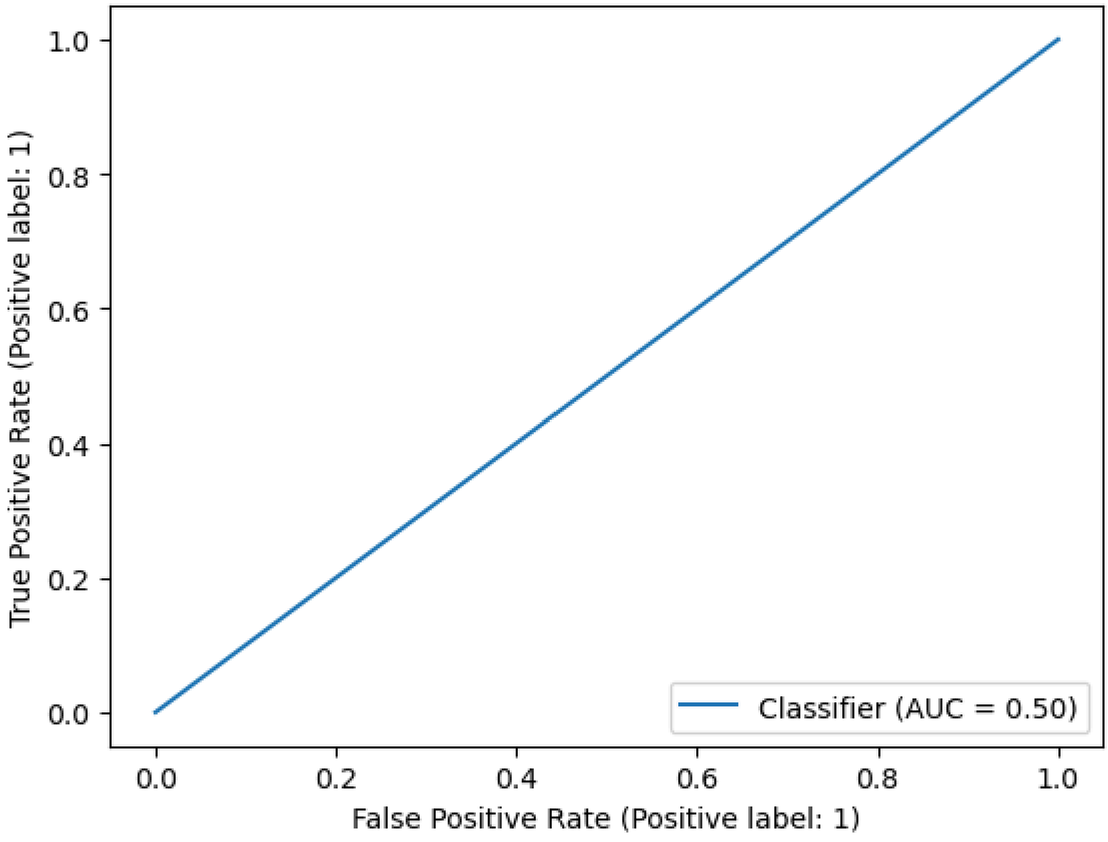

Import and apply KNN Classifier - results

In [51]:
```
from sklearn.neighbors import KNeighborsClassifier
```

In [52]:
```
knn = KNeighborsClassifier(n_neighbors=15)
knn.fit(X_train, y_train)
```

Out[52]:
```
▾ KNeighborsClassifier
KNeighborsClassifier(n_neighbors=15)
```

In [53]:
```
knn_predictions = knn.predict(X_test)
```

In [54]:
```
plotCM(y_test, knn_predictions)
```

```
accuracy Score is: 0.8826199740596627
```

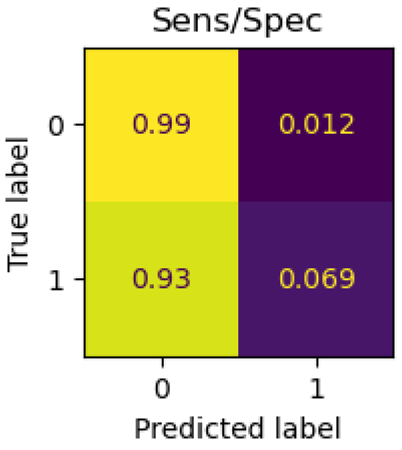

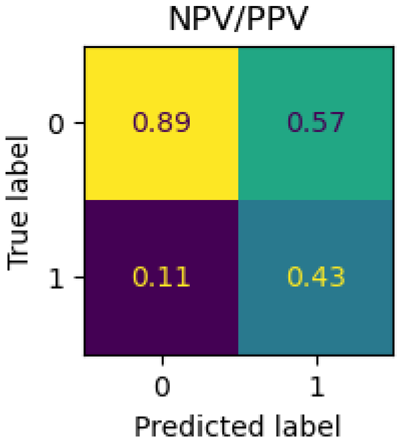

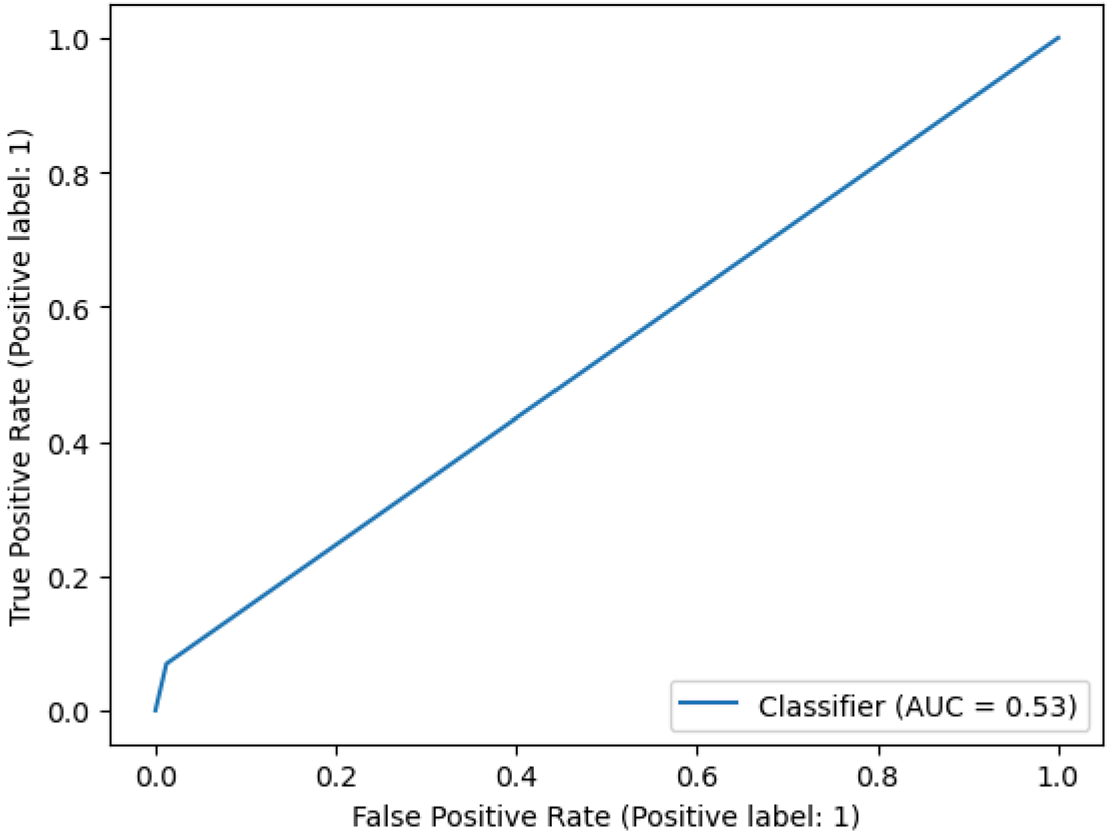

Import and apply Decision Tree Regression - results appear to be GARBAGE

In [55]:
```
from sklearn.tree import DecisionTreeClassifier
```

In [56]:
```
dtree = DecisionTreeClassifier(random_state=30)
dtree.fit(X_train, y_train)
```

Out[56]:
```
▾          DecisionTreeClassifier
DecisionTreeClassifier(random_state=30)
```

In [57]:
```
dtree_predictions = dtree.predict(X_test)
```

In [88]:
```
plotCM(y_test, dtree_predictions)
```

```
accuracy Score is: 0.8274562256809338
```

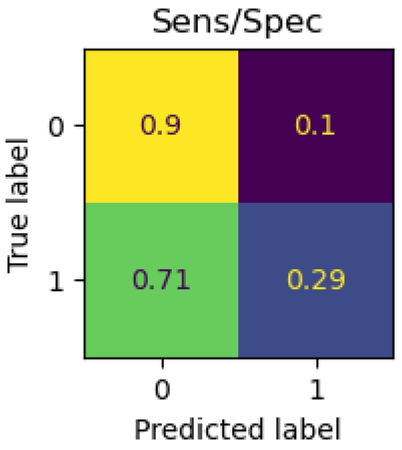

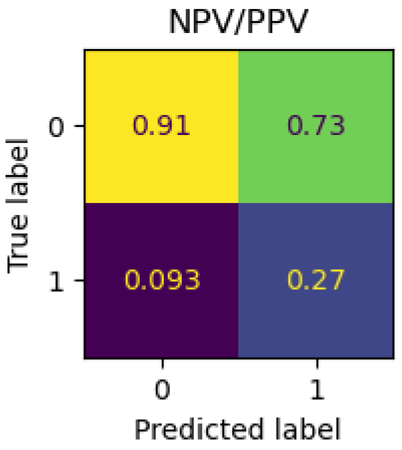

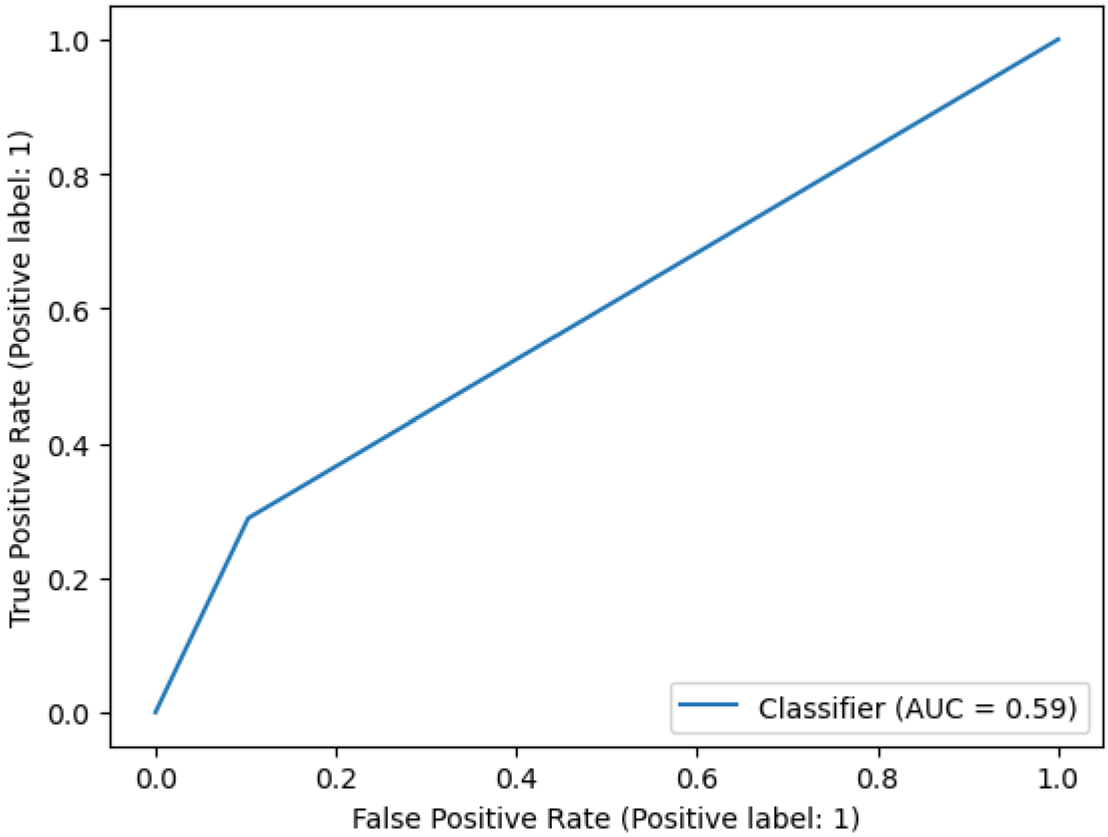

Attempt Gradient Boosting

In [59]:
```
from sklearn.ensemble import HistGradientBoostingClassifier
```

In [60]:
```
boost = HistGradientBoostingClassifier()
boost.fit(X_train, y_train)
```

Out[60]:
```
▾ HistGradientBoostingClassifier
HistGradientBoostingClassifier()
```

In [61]:
```
boost_predictions = boost.predict(X_test)
```

In [62]:
```
plotCM(y_test, boost_predictions)
```

```
accuracy Score is: 0.8879296368352788
```

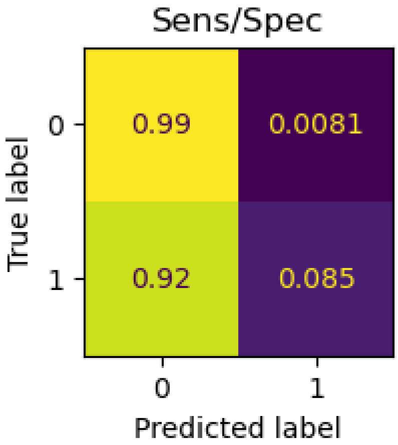

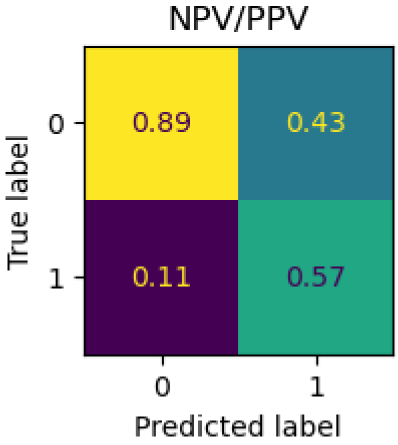

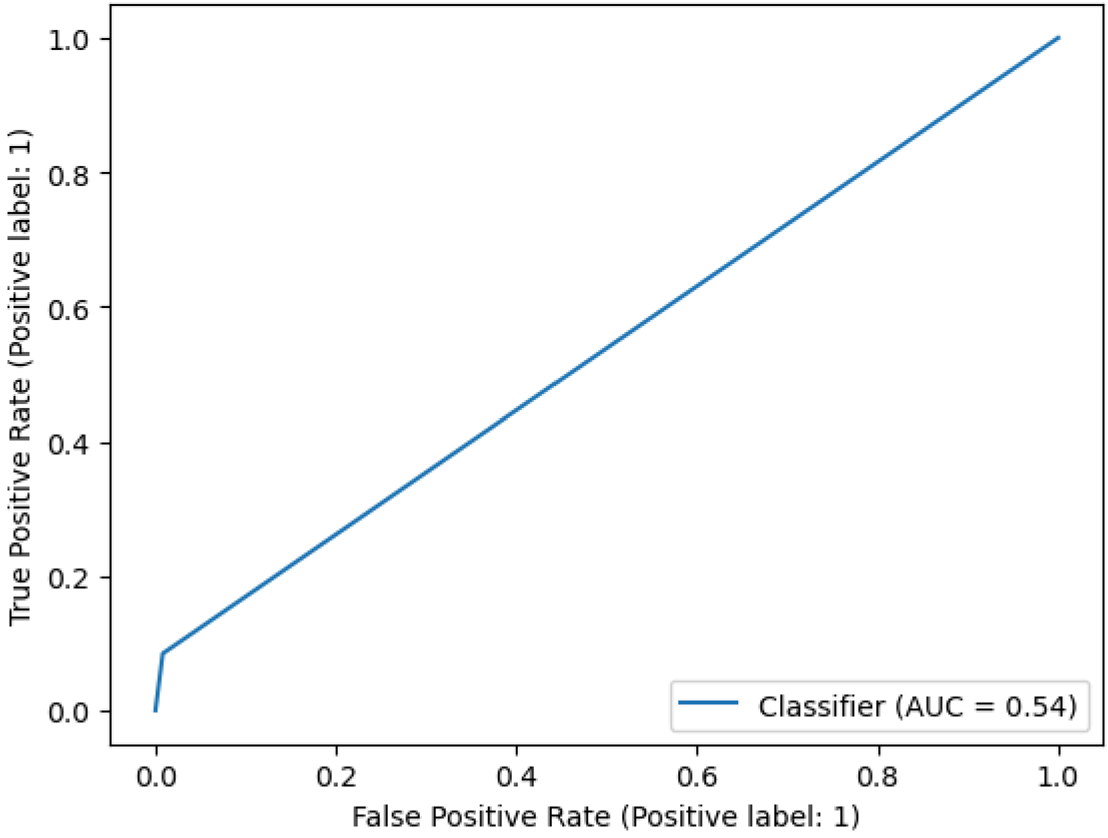

Attempt CatBoost

In [63]:
```
from catboost import CatBoostClassifier
import warnings
warnings.filterwarnings("ignore")
```

In [64]:
```
# Define the hyperparameters for the CatBoost algorithm
params = {'learning_rate': 0.1, 'depth': 6,
          'l2_leaf_reg': 3, 'iterations': 100}

# Initialize the CatBoostClassifier object
# with the defined hyperparameters and fit it on the training set
model = CatBoostClassifier(**params)
model.fit(X_train, y_train)
```

```
0:      learn: 0.5967375        total: 156ms    remaining: 15.5s
1:      learn: 0.5253791        total: 176ms    remaining: 8.63s
2:      learn: 0.4743676        total: 195ms    remaining: 6.32s
3:      learn: 0.4335223        total: 220ms    remaining: 5.27s
4:      learn: 0.4030726        total: 245ms    remaining: 4.66s
5:      learn: 0.3814371        total: 272ms    remaining: 4.26s
6:      learn: 0.3607224        total: 303ms    remaining: 4.03s
7:      learn: 0.3469929        total: 333ms    remaining: 3.83s
8:      learn: 0.3357863        total: 360ms    remaining: 3.64s
9:      learn: 0.3279067        total: 393ms    remaining: 3.54s
10:     learn: 0.3208617        total: 420ms    remaining: 3.4s
11:     learn: 0.3145188        total: 455ms    remaining: 3.33s
12:     learn: 0.3098107        total: 490ms    remaining: 3.28s
13:     learn: 0.3064641        total: 525ms    remaining: 3.22s
14:     learn: 0.3033767        total: 558ms    remaining: 3.16s
15:     learn: 0.3012548        total: 591ms    remaining: 3.1s
16:     learn: 0.2993233        total: 630ms    remaining: 3.08s
17:     learn: 0.2976984        total: 659ms    remaining: 3s
18:     learn: 0.2957648        total: 688ms    remaining: 2.93s
19:     learn: 0.2944674        total: 718ms    remaining: 2.87s
20:     learn: 0.2930493        total: 753ms    remaining: 2.83s
21:     learn: 0.2919909        total: 782ms    remaining: 2.77s
22:     learn: 0.2906454        total: 813ms    remaining: 2.72s
23:     learn: 0.2896667        total: 845ms    remaining: 2.68s
24:     learn: 0.2885502        total: 890ms    remaining: 2.67s
25:     learn: 0.2878487        total: 918ms    remaining: 2.61s
26:     learn: 0.2871039        total: 946ms    remaining: 2.56s
27:     learn: 0.2866188        total: 971ms    remaining: 2.5s
28:     learn: 0.2861816        total: 996ms    remaining: 2.44s
29:     learn: 0.2856275        total: 1.02s    remaining: 2.38s
30:     learn: 0.2850844        total: 1.04s    remaining: 2.32s
31:     learn: 0.2845909        total: 1.07s    remaining: 2.27s
32:     learn: 0.2843174        total: 1.09s    remaining: 2.22s
33:     learn: 0.2836853        total: 1.12s    remaining: 2.17s
34:     learn: 0.2834595        total: 1.14s    remaining: 2.11s
35:     learn: 0.2830645        total: 1.16s    remaining: 2.06s
36:     learn: 0.2828703        total: 1.18s    remaining: 2.01s
37:     learn: 0.2824900        total: 1.21s    remaining: 1.97s
38:     learn: 0.2821955        total: 1.24s    remaining: 1.94s
39:     learn: 0.2817881        total: 1.26s    remaining: 1.9s
40:     learn: 0.2814499        total: 1.31s    remaining: 1.88s
41:     learn: 0.2811289        total: 1.34s    remaining: 1.85s
42:     learn: 0.2809882        total: 1.37s    remaining: 1.81s
43:     learn: 0.2806514        total: 1.4s     remaining: 1.78s
44:     learn: 0.2804941        total: 1.42s    remaining: 1.74s
45:     learn: 0.2802918        total: 1.45s    remaining: 1.7s
46:     learn: 0.2800271        total: 1.47s    remaining: 1.65s
47:     learn: 0.2797289        total: 1.49s    remaining: 1.61s
48:     learn: 0.2795479        total: 1.51s    remaining: 1.58s
49:     learn: 0.2793708        total: 1.54s    remaining: 1.54s
50:     learn: 0.2792852        total: 1.56s    remaining: 1.5s
51:     learn: 0.2789950        total: 1.59s    remaining: 1.46s
52:     learn: 0.2788498        total: 1.61s    remaining: 1.43s
53:     learn: 0.2786824        total: 1.64s    remaining: 1.39s
54:     learn: 0.2785277        total: 1.66s    remaining: 1.36s
55:     learn: 0.2783967        total: 1.69s    remaining: 1.32s
56:     learn: 0.2781121        total: 1.71s    remaining: 1.29s
57:     learn: 0.2779190        total: 1.74s    remaining: 1.26s
58:     learn: 0.2777846        total: 1.77s    remaining: 1.23s
59:     learn: 0.2776680        total: 1.8s     remaining: 1.2s
```

```
60:	learn: 0.2775703	total: 1.83s	remaining: 1.17s
61:	learn: 0.2773207	total: 1.86s	remaining: 1.14s
62:	learn: 0.2770597	total: 1.88s	remaining: 1.11s
63:	learn: 0.2769684	total: 1.91s	remaining: 1.07s
64:	learn: 0.2767745	total: 1.94s	remaining: 1.04s
65:	learn: 0.2766835	total: 1.97s	remaining: 1.01s
66:	learn: 0.2764688	total: 2s	remaining: 984ms
67:	learn: 0.2763408	total: 2.03s	remaining: 957ms
68:	learn: 0.2761892	total: 2.06s	remaining: 928ms
69:	learn: 0.2760955	total: 2.09s	remaining: 897ms
70:	learn: 0.2759226	total: 2.12s	remaining: 867ms
71:	learn: 0.2758471	total: 2.15s	remaining: 838ms
72:	learn: 0.2756439	total: 2.19s	remaining: 810ms
73:	learn: 0.2755128	total: 2.23s	remaining: 782ms
74:	learn: 0.2753848	total: 2.26s	remaining: 752ms
75:	learn: 0.2753083	total: 2.28s	remaining: 721ms
76:	learn: 0.2750391	total: 2.31s	remaining: 691ms
77:	learn: 0.2748980	total: 2.34s	remaining: 659ms
78:	learn: 0.2748152	total: 2.37s	remaining: 629ms
79:	learn: 0.2746296	total: 2.39s	remaining: 598ms
80:	learn: 0.2744412	total: 2.42s	remaining: 568ms
81:	learn: 0.2743703	total: 2.45s	remaining: 538ms
82:	learn: 0.2742816	total: 2.48s	remaining: 508ms
83:	learn: 0.2741451	total: 2.51s	remaining: 478ms
84:	learn: 0.2739683	total: 2.54s	remaining: 448ms
85:	learn: 0.2738292	total: 2.56s	remaining: 418ms
86:	learn: 0.2737726	total: 2.6s	remaining: 388ms
87:	learn: 0.2737060	total: 2.62s	remaining: 358ms
88:	learn: 0.2736240	total: 2.65s	remaining: 328ms
89:	learn: 0.2735133	total: 2.69s	remaining: 298ms
90:	learn: 0.2733832	total: 2.71s	remaining: 268ms
91:	learn: 0.2732694	total: 2.75s	remaining: 239ms
92:	learn: 0.2731797	total: 2.78s	remaining: 209ms
93:	learn: 0.2730555	total: 2.81s	remaining: 179ms
94:	learn: 0.2729270	total: 2.84s	remaining: 150ms
95:	learn: 0.2728182	total: 2.88s	remaining: 120ms
96:	learn: 0.2726756	total: 2.91s	remaining: 90.1ms
97:	learn: 0.2725469	total: 2.95s	remaining: 60.2ms
98:	learn: 0.2724813	total: 2.98s	remaining: 30.1ms
99:	learn: 0.2723096	total: 3.02s	remaining: 0us
```

Out[64]:
```
<catboost.core.CatBoostClassifier at 0x22f1e16a190>
```

In [65]:
```python
y_pred = model.predict(X_test)
```

In [66]:
```python
plotCM(y_test, y_pred)
```

```
accuracy Score is: 0.8882538910505836
```

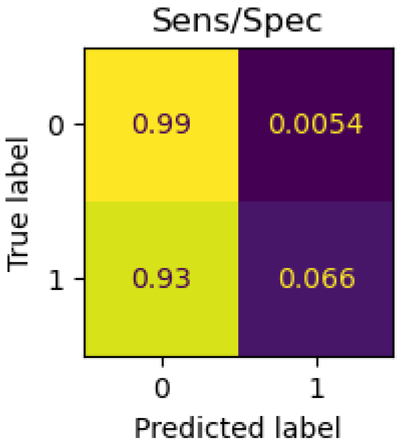

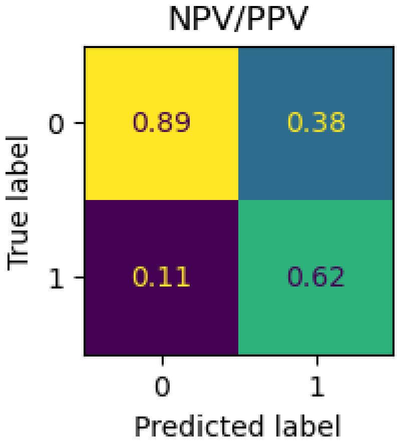

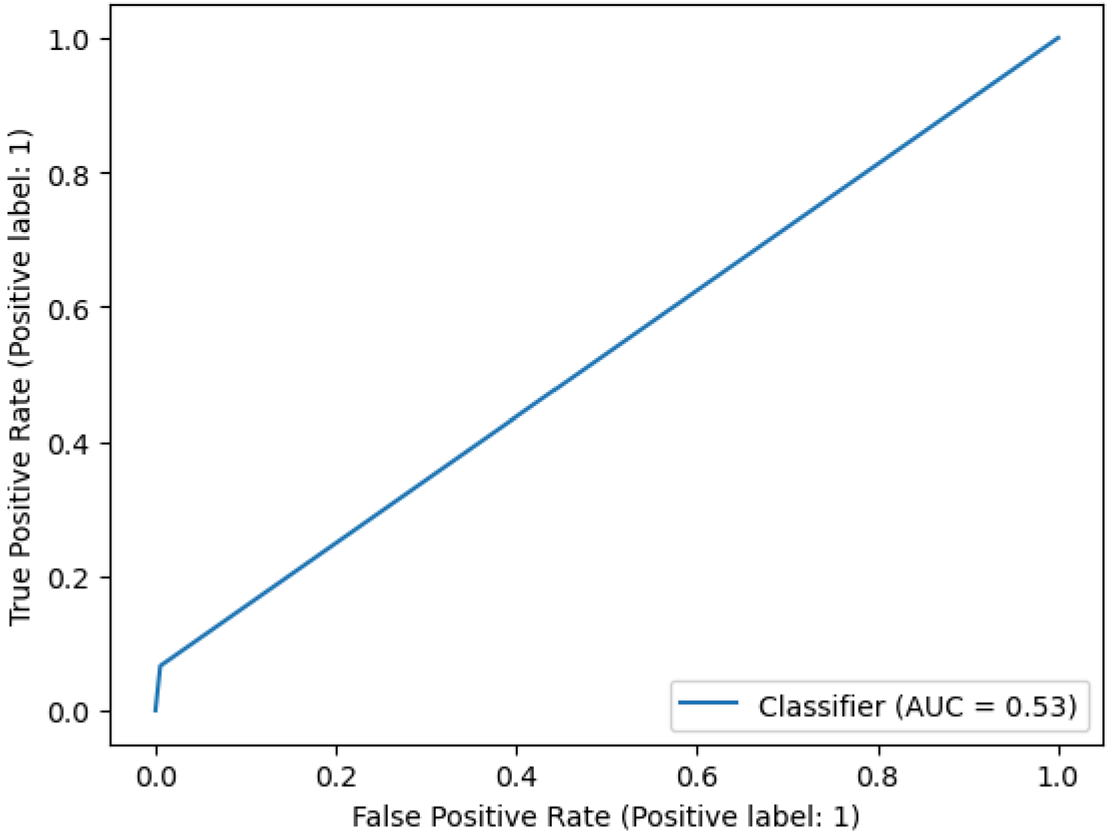

Neural Network

split Training data again in 75:25 ration to generate a total 60:20:20 split for Train:Validate:Test

In [67]:
```
X_train, X_val, y_train, y_val = train_test_split(X_train, y_train, test_size=0.25, ra
```

In [68]:
```
# Define function to plot Training Loss vs Validation Loss across epochs
def drawPlot():
    plt.plot(history.history['loss'])
    plt.plot(history.history['val_loss'])
    plt.title('model Loss')
    plt.ylabel('Loss')
    plt.xlabel('Epoch')
    #plt.xticks(np.arange(0, 21, 1.0))
    plt.legend(['train', 'val'], loc='upper left')
    plt.show()
```

In [71]:
```
drawPlot()
```

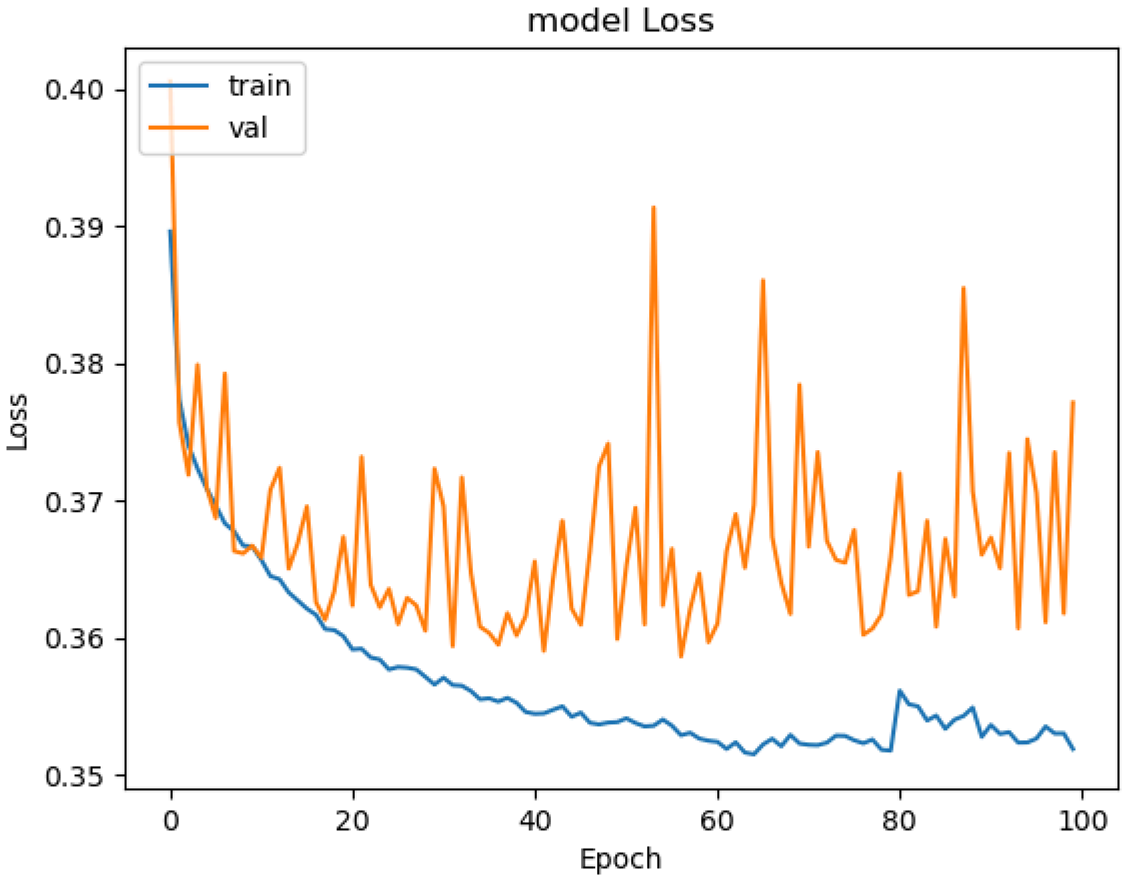

In [90]:
```
network = models.Sequential()
network.add(layers.Dense(512, activation='relu', input_shape=(23, )))
network.add(layers.Dense(256, activation='relu'))
network.add(layers.Dense(128, activation='relu'))
network.add(layers.Dense(64, activation='relu'))
network.add(layers.Dense(32, activation='relu'))
network.add(layers.Dense(16, activation='relu'))
network.add(layers.Dense(8, activation='relu'))
network.add(layers.Dense(4, activation='relu'))
network.add(layers.Dense(2, activation='relu'))
network.add(layers.Dense(1, activation='sigmoid'))
network.compile(optimizer=optimizers.RMSprop(),
```

```
                 loss='binary_crossentropy',
                 metrics=['accuracy'])
```

In [87]:
```
# fit model
history = network.fit(X_train, y_train,
                      batch_size=128, epochs=10,
                      validation_data = (X_val, y_val))
```

```
Epoch 1/10
579/579 [==============================] - 6s 8ms/step - loss: 0.5932 - accuracy: 0.8
875 - val_loss: 0.5099 - val_accuracy: 0.8874
Epoch 2/10
579/579 [==============================] - 4s 7ms/step - loss: 0.4510 - accuracy: 0.8
877 - val_loss: 0.4050 - val_accuracy: 0.8874
Epoch 3/10
579/579 [==============================] - 4s 7ms/step - loss: 0.3779 - accuracy: 0.8
877 - val_loss: 0.3601 - val_accuracy: 0.8874
Epoch 4/10
579/579 [==============================] - 4s 7ms/step - loss: 0.3538 - accuracy: 0.8
877 - val_loss: 0.3522 - val_accuracy: 0.8874
Epoch 5/10
579/579 [==============================] - 5s 8ms/step - loss: 0.3514 - accuracy: 0.8
877 - val_loss: 0.3520 - val_accuracy: 0.8874
Epoch 6/10
579/579 [==============================] - 4s 8ms/step - loss: 0.3513 - accuracy: 0.8
877 - val_loss: 0.3520 - val_accuracy: 0.8874
Epoch 7/10
579/579 [==============================] - 4s 7ms/step - loss: 0.3513 - accuracy: 0.8
877 - val_loss: 0.3520 - val_accuracy: 0.8874
Epoch 8/10
579/579 [==============================] - 4s 8ms/step - loss: 0.3513 - accuracy: 0.8
877 - val_loss: 0.3520 - val_accuracy: 0.8874
Epoch 9/10
579/579 [==============================] - 4s 7ms/step - loss: 0.3513 - accuracy: 0.8
877 - val_loss: 0.3520 - val_accuracy: 0.8874
Epoch 10/10
579/579 [==============================] - 5s 8ms/step - loss: 0.3513 - accuracy: 0.8
877 - val_loss: 0.3520 - val_accuracy: 0.8874
```

In [89]:
```
# Concatenate Train and Validation Data
X_training = np.concatenate((X_train, X_val), axis=0)
y_training = np.concatenate((y_train, y_val), axis=0)
```

In [91]:
```
# Re-train network on complete Training Data, with Epochs set at 25
network.fit(X_training, y_training,
            batch_size=128, epochs=10)
```

```
Epoch 1/10
771/771 [==============================] - 9s 9ms/step - loss: 0.3299 - accuracy: 0.8
865
Epoch 2/10
771/771 [==============================] - 7s 9ms/step - loss: 0.2972 - accuracy: 0.8
876
Epoch 3/10
771/771 [==============================] - 7s 9ms/step - loss: 0.2947 - accuracy: 0.8
876
Epoch 4/10
771/771 [==============================] - 7s 10ms/step - loss: 0.2932 - accuracy: 0.
8876
Epoch 5/10
771/771 [==============================] - 7s 9ms/step - loss: 0.2924 - accuracy: 0.8
877
Epoch 6/10
771/771 [==============================] - 7s 9ms/step - loss: 0.2915 - accuracy: 0.8
881
Epoch 7/10
771/771 [==============================] - 7s 9ms/step - loss: 0.2903 - accuracy: 0.8
886
Epoch 8/10
771/771 [==============================] - 7s 8ms/step - loss: 0.2895 - accuracy: 0.8
890
Epoch 9/10
771/771 [==============================] - 7s 9ms/step - loss: 0.2892 - accuracy: 0.8
890
Epoch 10/10
771/771 [==============================] - 7s 9ms/step - loss: 0.2884 - accuracy: 0.8
892
```

Out[91]:

```
<keras.src.callbacks.History at 0x22f42fb0c50>
```

In [92]:

```
y_prediction = network.predict(X_test)
```

```
771/771 [==============================] - 3s 3ms/step
```

In [93]:

```
network.evaluate(X_test, y_test)
```

```
771/771 [==============================] - 3s 4ms/step - loss: 0.2940 - accuracy: 0.8
862
```

Out[93]:

```
[0.294021338224411, 0.8862273097038269]
```

In [94]:

```
y_prediction[y_prediction >= 0.5] = 1
y_prediction[y_prediction < 0.5] = 0
```

In [95]:

```
nnDF= pd.DataFrame(y_prediction)
```

In [96]:

```
nnDF.value_counts()
```

Out[96]:

```
0.0    24430
1.0      242
Name: count, dtype: int64
```

In [97]:

```
plotCM(y_test, y_prediction)
```

```
accuracy Score is: 0.8862273022049286
```

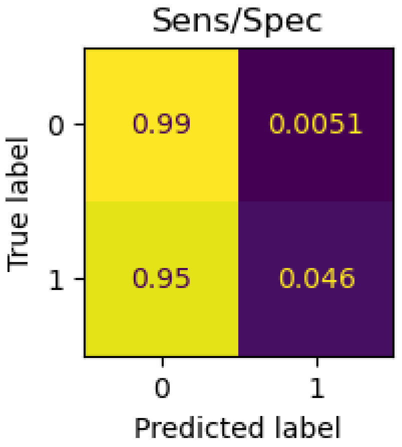
Sens/Spec
0.99
0.0051
0.95
0.046
True label
Predicted label

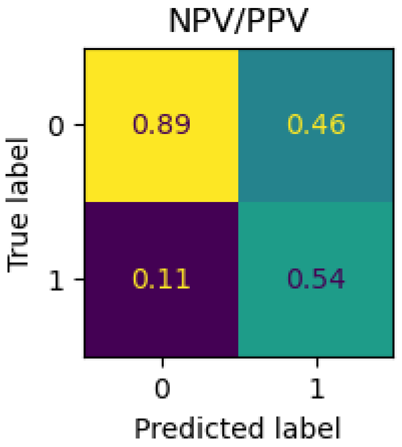
NPV/PPV
0.89
0.46
0.11
0.54
True label
Predicted label

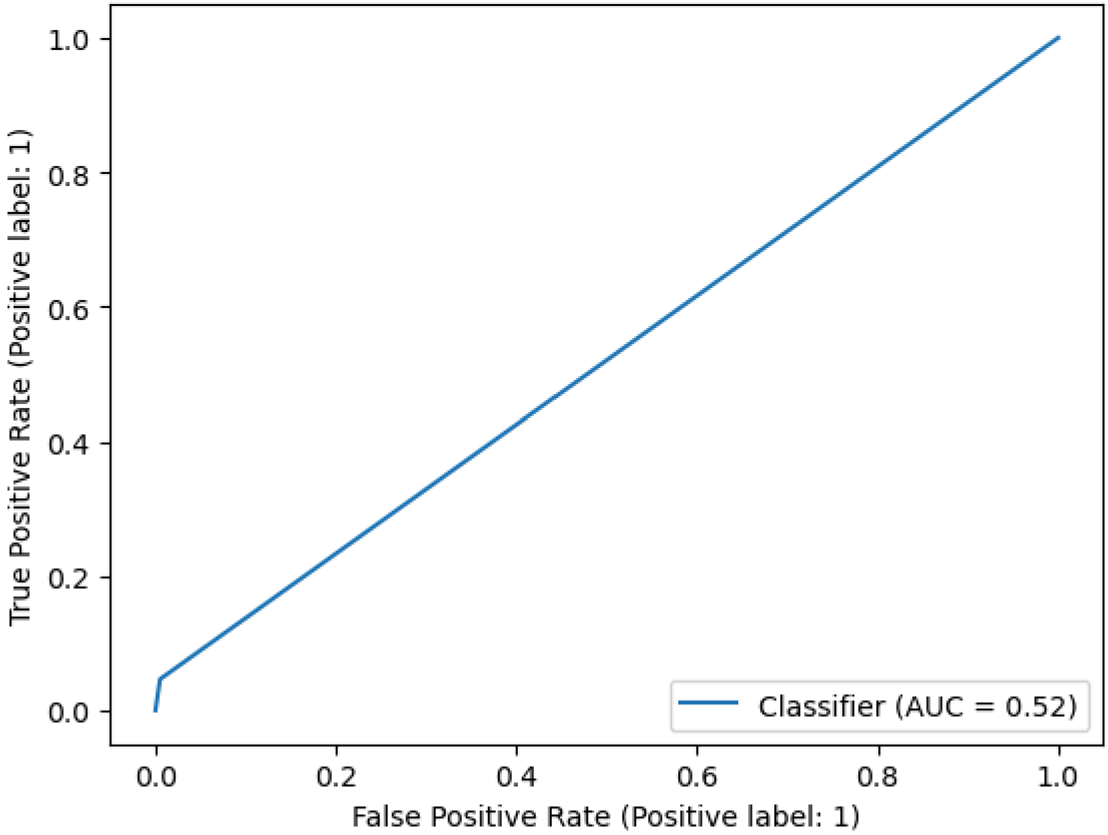
True Positive Rate (Positive label: 1)
False Positive Rate (Positive label: 1)
Classifier (AUC = 0.52)
